# Integrated *N*- and *O*-Glycomics of Acute Myeloid Leukemia (AML) Cell Lines

**DOI:** 10.3390/cells10113058

**Published:** 2021-11-06

**Authors:** Constantin Blöchl, Di Wang, Katarina Madunić, Guinevere S. M. Lageveen-Kammeijer, Christian G. Huber, Manfred Wuhrer, Tao Zhang

**Affiliations:** 1 Center for Proteomics and Metabolomics, Leiden University Medical Center, Albinusdreef 2, 2333 ZA Leiden, The Netherlands; constantin.bloechl@sbg.ac.at (C.B.); d.wang@lumc.nl (D.W.); k.madunic@lumc.nl (K.M.); g.s.m.kammeijer@lumc.nl (G.S.M.L.-K.); 2 Department of Biosciences, University of Salzburg, Hellbrunnerstrasse 34, 5020 Salzburg, Austria; 3 Cancer Cluster Salzburg, Department of Biosciences, University of Salzburg, Hellbrunnerstrasse 34, 5020 Salzburg, Austria

**Keywords:** PGC nano-LC-MS^2^, *N*-glycosylation, *O*-glycosylation, tumor microenvironment, sialyl Lewis x/a, α-2,8 sialylation, glycosyltransferases, hematopoietic transcription factors

## Abstract

Acute myeloid leukemia (AML) is characterized by a dysregulated expansion of poorly differentiated myeloid cells. Although patients are usually treated effectively by chemotherapy, a high rate of relapsed or refractory disease poses a major hurdle in its treatment. Recently, several studies have proposed implications of protein glycosylation in the pathobiology of AML including chemoresistance. Accordingly, associations have been found between specific glycan epitopes and the outcome of the disease. To advance this poorly studied field, we performed an exploratory glycomics study characterizing 21 widely used AML cell lines. Exploiting the benefits of porous graphitized carbon chromatography coupled to tandem mass spectrometry (PGC nano-LC-MS^2^), we qualitatively and quantitatively profiled *N*- and *O*-linked glycans. AML cell lines exhibited distinct glycan fingerprints differing in relevant glycan traits correlating with their cellular phenotype as classified by the FAB system. By implementing transcriptomics data, specific glycosyltransferases and hematopoietic transcription factors were identified, which are candidate drivers of the glycan phenotype of these cells. In conclusion, we report the varying expression of glycan structures across a high number of AML cell lines, including those associated with poor prognosis, identified underlying glycosyltransferases and transcription factors, and provide insights into the regulation of the AML glycan repertoire.

## 1. Introduction

Acute myeloid leukemia (AML) is a genetically heterogeneous disease characterized by clonal expansion of irregularly differentiated cells of the myeloid lineage termed blasts [1]. It is the most common type of acute leukemia with a median age of 68 years at diagnosis [2,3]. Due to its heterogeneity, the classification of AML is of high importance for risk assessment, choice of treatment, and general stratification of the disease [4]. Traditionally, AML has been categorized according to the French-American-British (FAB) classification, which is predominantly based on the morphological appearance of leukemic blasts as well as their cytochemical characteristics [5]. This classification system defines eight major subtypes specified as M0 to M7. In 2001, the World Health Organization (WHO) introduced a novel classification system for AML [6], and in its latest version [7], six major groups were defined based on clinical, morphological, genetic, and immunophenotypic features. The group with recurring genetic abnormalities can be further classified based on specific chromosomal aberrations revealing 11 subtypes, e.g., AML with mutated *NPM1* or the *PML-RARA* fusion gene. However, if the AML subtype is not specified otherwise, morphological and phenotypic criteria similar to the FAB classification are still considered.

Despite the ongoing advances in cancer therapy and the general understanding of AML biology, the predominant treatment regimens remained largely unchanged for the past decades [8]. For those who are eligible, it generally consists of strong induction chemotherapy followed by consolidation therapy to avoid relapse of the disease [4]. Whilst induction therapy leads to clinical remission in a substantial number of patients, relapsed or refractory disease remains one of the principal difficulties in AML with an overall survival of only around 10% in those patients [9].

In the last decade, it has been increasingly recognized that AML blasts that reside in the bone marrow create a hostile pro-tumoral microenvironment, which may be responsible for the dysregulation of normal hematopoiesis as well as chemoresistance, immune evasion, and eventually, relapse of the disease [10,11,12]. This niche is established through a complex interplay of soluble biomolecules, extracellular vesicle-mediated signaling, and direct cellular interaction and is, to date, still not fully understood [13]. In this regard, glycosylation of leukemic cells may add to the complex picture. Cancer-associated changes in glycosylation that may modulate these malignant properties include increased sialylation as well as high expression of Lewis (Le) structures and truncated glycans, e.g., Tn or T antigens [14,15]. In hematological malignancies, several of these aberrant protein glycosylation features have been described and may involve both *N*-glycans (linked to Asn residues) and *O*-glycans (linked to Ser or Thr residues), as reviewed by Pang et al. [16].

Especially, the role of the sialyl Lewis x/a (sLe^x/a^) antigen expression in AML and its direct interaction with E-selectin in the bone marrow niche is intriguing. Reported implications of this interplay include altered homing of leukemic cells as well as regulation of their cellular proliferation and quiescence, respectively [17,18,19]. In a recent report, Barbier et al. showed that the interaction between sLe^x/a^ and E-selectin is a major determinant of chemoresistance in AML [20]. The glycomimetic drug Uproleselan/GMI-1271, a novel E-selectin antagonist, disrupts this interplay to diminish resistance to chemotherapeutics in AML among other cancer entities [20,21]. This therapeutic agent is currently investigated in clinical trials including a phase 3 trial in relapsed or refractory AML [22,23].

Attachment of AML blasts to cells of the bone marrow microenvironment may be additionally governed by the membrane glycoprotein CD82, which organizes *N*-cadherin on the cellular surface of blasts [24]. The activity of CD82 is in turn strongly modulated by its three extracellular *N*-glycosylation sites [25]. Interestingly, the involvement of CD82 in the chemoresistance of AML has been recently proposed [26]. In this regard, glycomics studies on adriamycin-resistant AML cell lines have also reported the involvement of α-2,6 and α-2,8 sialylation on *N*-glycans in chemoresistance [27,28]. More precisely, by a stepwise addition of the chemotherapeutic adriamycin, which is widely used in the treatment of several solid tumors and acute leukemias [29], a multi drug resistance (MDR) phenotype was induced [27]. Aberrant sialylation identified in these resistant cells mediated a change in the phosphoinositide-3 kinase (PI3K)/Akt signaling pathway accompanied by altered expression of P-glycoprotein and MDR-related protein 1, both of which are strongly associated with chemoresistance [27].

In addition to altered glycosylation patterns of proteins presented at the cellular surface, protein glycosylation is also crucial for intrinsic leukemic pathways. For instance, the activity of the receptor tyrosine kinases FLT3, which is frequently mutated in AML and usually associated with a worse outcome, may be altered by both its *N*- and *O*-glycosylation [30]. Intriguingly, both hyper- and hypo-glycosylation of FLT3 induced by potential therapeutic agents may be modes of action to target malignant FLT3-signaling [30,31,32,33].

While substantial evidence has been gathered that points towards an essential role of protein glycosylation in AML, e.g., in niche formation and chemoresistance, an exploratory glycomics study identifying and characterizing relevant glycan structures has not been conducted to date. Moreover, associations of AML classes as specified by FAB or WHO and their glycomic fingerprint were hitherto not investigated. In turn, this may provide potential benefits to the further stratification of the disease. Therefore, we set out to thoroughly characterize the *N*- and *O*-glycome of 21 widely used cell lines reflecting most of the genetic and phenotypic variability of AML in an integrated manner. Relying on a robust 96-well plate sample preparation strategy [34] and state-of-the-art glycomics techniques, i.e., porous graphitized carbon nano-liquid chromatography coupled to tandem mass spectrometry (PGC nano-LC-MS^2^), more than 90 distinct *N*- and *O*-glycan structures could be structurally characterized and relatively quantified. We report a comprehensive library of glycans present in common AML cell lines and identify the associated antigens, e.g., T antigen, sLe^x/a^, and α-2,8 sialylation, as a valuable tool for future research. Based on a principal component analysis (PCA), we identified a strong association between the glycomic fingerprint of AML cells and their phenotypic and cytochemical characteristics as classified by the FAB system. In addition, we linked acquired glycomics information to the available transcriptomics data to identify the involved glycosyltransferases (GSTs) and, eventually, gathered evidence for the upstream involvement of key hematopoietic transcription factors (TFs) in AML protein glycosylation.

## 2. Materials and Methods

### 2.1. Cell Culture

AML cell lines were obtained from the Department of Hematology (Leiden University Medical Center, Leiden, The Netherlands), Department of Immunopathology—Sanquin Research (Sanquin, Amsterdam, The Netherlands), and the Department of Biosciences (University of Salzburg, Salzburg, Austria). An overview of used cell lines is listed in Supplementary Table S1. All of the cell lines were cultured in Iscove’s Modified Dulbecco’s Medium (IMDM) (Gibco, Thermo Fisher Scientific, Waltham, MA, USA) containing 1% penicillin-streptomycin (Invitrogen, Thermo Fisher Scientific) at 37 °C, under normoxic conditions, and 5% CO_2_. Cell lines KG-1, KG-1a, HL-60, PLB985, NB-4, ML-1, OCI-AML2, OCI-AML3, EOL-1, MOLM-13, MOLM-14, MV4-11, THP-1, U937, HEL, HEL 92.1.7, TF-1, and M-07e were cultured in media with 10% FBS (fetal bovine serum), whereas Kasumi-1 and ME-1 were grown in media with 20% FBS and AML193 with 5% FBS. Media for TF-1 and M-07e additionally contained 20 ng·mL^−1^ granulocyte-macrophage colony-stimulating factor (GM-CSF; Cellgenix, Freiburg, Germany). Cells were washed thoroughly with phosphate-buffered saline before conducting the glycomics analysis.

### 2.2. Sample Preparation

*N*- and *O*-glycans were analyzed based on polyvinylidene difluoride (PVDF; Millipore, Amsterdam, The Netherlands) membrane-based glycan release workflow using a 96-well plate format, as previously described [34]. Briefly, 500,000 cells were lysed by sonication in water, followed by protein denaturation upon addition of dithiothreitol (Sigma-Aldrich, Steinheim, Germany) to 5.0 mmol·L^−1^, guanidine hydrochloride (Thermo Fisher Scientific) to 5.8 mol·L^−1^, and incubation at 60 °C for 30 min. Subsequently, proteins were washed with water before applying PNGase F (Roche Diagnostics, Mannheim, Germany) overnight at 37 °C. In this step, 10 ng maltoheptaose DP7 (Elicityl, Crolles, France) was included as a spiked internal standard to monitor sample preparation and PGC nano-LC-MS^2^ performance. Released *N*-glycans were collected, incubated in approximately 6 mmol·L^−1^ ammonium acetate (pH 5.0; Sigma-Aldrich) for 1.0 h at room temperature, and dried by vacuum centrifugation. *N*-glycans were reduced by resuspension in 50 mmol·L^−1^ potassium hydroxide (Honeywell Fluka, Thermo Fisher Scientific) supplemented with 1 mol·L^−1^ sodium borohydride (Sigma-Aldrich) at 60 °C for 3.0 h. After the removal of *N*-glycans from the immobilized proteins, *O*-glycans were released by reductive β-elimination in a 50 mmol·L^−1^ KOH solution supplemented with 500 mmol·L^−1^ NaBH_4_ at 55 °C for 16.0 h. Likewise, 5 ng maltopentaose DP5 (Elicityl) was introduced in this step as an internal standard for *O*-glycans. Next, both *N*- and *O*-glycans were desalted on a strong cation exchange resin (Dowex 50 W X8; Merck, Darmstadt, Germany) self-packed into 96-well filter plates (Orochem Technologies, Naperville, IL, USA) followed by removal of boric acid by co-evaporation with methanol in a vacuum centrifuge. Eventually, released glycans were purified on carbograph material (Grace Discovery Sciences, Columbia, TN, USA) that was also self-packed into 96-well filter plates. Purified released glycans were dried employing a vacuum centrifuge and resuspended in 10 µL of H_2_O prior to analysis.

Sialic acid linkages were determined by enzymatic digestion of purified *N*- and *O*-glycans with α-2,3 neuraminidase S and α-2,3,6,8,9 neuraminidase A (both from New England Biolabs, Ipswich, MA, USA), respectively, and subsequent PGC nano-LC-MS^2^ analysis.

### 2.3. PGC Nano-LC-MS^2^

Chromatographic separation of released *N*- and *O*-glycan alditols was conducted on an Ultimate 3000 nano-HPLC (Thermo Fisher Scientific) equipped with a self-packed trap column (5 µm particle diameter, 30 mm × 0.32 mm i.d.) and a self-packed separation column (3 µm particle diameter, 100 mm × 0.075 mm i.d.) for *N*-glycans. Separation of *O*-glycans was conducted on a 0.10 mm i.d. column. In all of the cases, Hypercarb™ KAPPA material (Thermo Fisher Scientific) was used for column packing. Separation was conducted at a flow rate of 600 nL·min^−1^ and a column oven temperature of 45 °C. For *N*-glycan analysis, 5.0 µL of sample were injected, whereas for *O*-glycan analysis, 4.0 µL of sample were injected. The nano-LC system was hyphenated to an amaZon ETD speed ion trap mass spectrometer via a CaptiveSpray ESI source (both from Bruker Daltonics, Bremen, Germany). Isopropanol was used as dopant solvent. For further details, see [34].

### 2.4. Data Evaluation

Glycans were identified based on retention time, acquired MS^1^ and MS^2^ information, general glycobiological knowledge on *N*- and *O*-glycan biosynthesis in humans [35], as well as exoglycosidase digests. Fragment ions were manually assigned with the assistance of GlycoWorkbench 2.1 [36] and established understandings of glycan fragmentation in negative ion mode collision-induced dissociation, as summarized in [37,38]. If available, UniCarbDB was used to obtain reference fragment spectra [39]. Compiled information on glycan identification and MS^2^ fragment assignments is listed in Supplementary Information 1 (*N*-glycans) and 2 (*O*-glycans). For relative quantification of glycan abundances, the data were converted into the mzml format and quantified employing Skyline 20.2.0.343, taking advantage of the small molecule interface [40]. Glycans passing the quality criteria (matching retention time, isotope dot product ≥0.85, and a signal-to-noise ratio of ≥6) were considered for quantification.

Missing values were imputed by the minimum positive number (0.01) before employing statistical evaluation of the data. PCA was conducted in SIMCA 13.0.3.0. (Sartorius, Göttingen, Germany). All of the other data evaluation steps were performed and visualized using the programming language “R”. Moreover, rCCA was conducted employing the mixOmics package [41]. Cell line transcriptomics data have been obtained from the “Expression Public 21Q1” dataset accessed via the depmap portal (Broad Institute, Cambridge, MA, USA) [42].

## 3. Results

To jointly characterize *N*- and *O*-glycans present in AML cell lines appertaining to a broad range of classes, we made use of an established sample preparation protocol relying on PVDF-assisted glycan release in a 96-well format [34]. Sequentially, *N*- and *O*-glycans were released from the immobilized proteome, purified, and subjected to in-depth structural characterization and relative quantification by means of PGC nano-LC-MS^2^. Glycan structures were assigned and quantified as detailed in the Materials and Methods section. Identified glycan structures and their assigned MS^2^ spectra are supplied as Supplementary Information 1 (*N*-glycans) and 2 (*O*-glycans). Glycans were abbreviated according to their monosaccharide composition: Hexose (H), *N-*acetylhexosamine (N), fucose (F), *N-*acetylneuraminic acid (S), phosphorylation (P), and sulfation (Su). Glycan isomers are labeled by letters as suffix, e.g., H5N4S2a and H5N4S2b starting with isomers of earlier retention time. Quantitative information on glycan fractional abundances is summarized in the Supplementary Excel file. To gain a better overview of the glycomic data and to investigate GST activity, we grouped individual glycans into specific glycan features (Supplementary Excel file). We considered glycan types, e.g., complex or hybrid type, as well as derived traits such as bisection or α-2,3 sialylation. Furthermore, we assessed sialic acid linkage in both *N*- and *O*-glycans, taking advantage of the well-studied retentive behavior in PGC chromatography [43], and confirmed linkages via neuraminidase digests and subsequent PGC nano-LC-MS^2^ analysis. Some sialic acid linkages of more complex multisialylated glycans could be additionally assigned based on matching retention times with well-studied glycans of fetuin that was measured alongside AML glycans [44]. However, for some extensively branched, multisialylated glycans, the sialic acid linkages could not be determined due to their high complexity.

### 3.1. N-Glycomics

*N*-glycosylation of AML was assessed in 21 cell lines and revealed a cumulative number of 68 glycans that passed the quality criteria for being considered in a qualitative and quantitative manner. The distribution of these glycans in the respective cell lines is visualized in Supplementary Figure S1. Although the technical variation of the employed workflow was assessed in our previous study in detail [34], we exemplarily visualized standard deviations for the cell line MOLM-14 (Supplementary Figure S2). Technical triplicates of glycans ≥ 1.0% fractional abundance obtained from the same biological replicate showed an average relative standard deviation (RSD) of 7.8%, whereas biological triplicates showed an average RSD of 11.8%. Biological variation compared to the variation between different cell lines will be discussed in Section 3.3.1.

**Figure 1:**
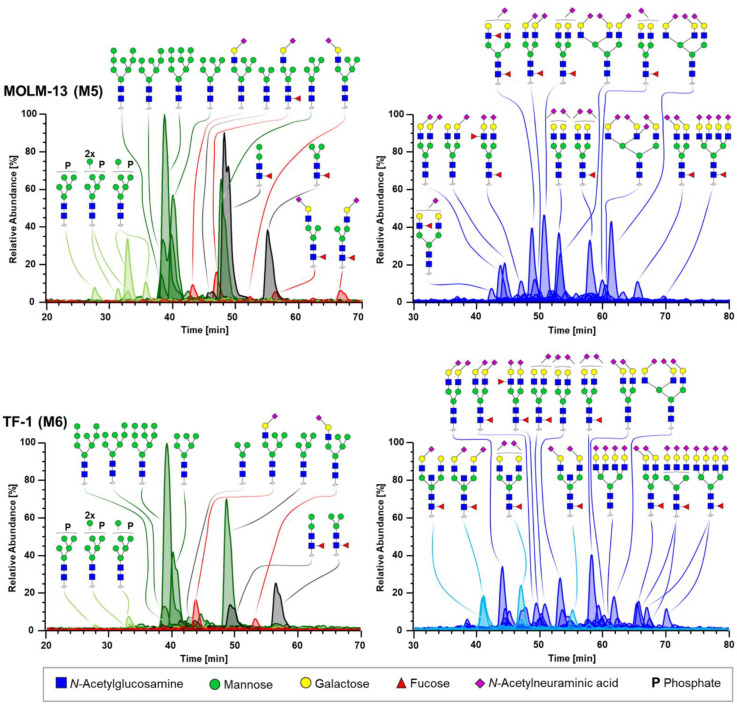
Exemplary combined EICCs of *N*-glycans obtained for the M5 subtype MOLM-13 (**upper panels**) and the M6 subtype TF-1 (**lower panels**). The most abundant glycans are annotated. Mass traces for oligomannose (dark green), phosphorylated oligomannose (light green), paucimannose (grey), and hybrid type (red) glycans are depicted in the two panels on the left. Traces of complex type (blue) glycans are shown in the right panels for the respective cell line. Bisected complex glycans are illustrated in light blue. Relative abundances are scaled to the most abundant glycan taking into account all of the glycan types. The symbol and color code of monosaccharides used for illustrating glycan structures is depicted beneath the panels. Sialic acids in α-2,3 linkage are tilted to the left, α-2,6 are tilted to the right, and sialic acids in unknown glycosidic linkage are displayed with a vertical connection.

The *N*-glycan profiles of individual AML cell lines showed a high diversity of structures covering all four main glycan types, namely oligomannose, paucimannose, hybrid, and complex type glycans (Supplementary Figure S1). Although these four glycan types were identified throughout all of the investigated cell lines, relative abundances varied drastically between the specific cell lines, as illustrated in Figure 1. Usually, large relative amounts of oligomannose glycans were detected ranging from a fractional abundance of 36.1% in KG-1 cells up to 67.4% in ML-1 cells. Paucimannose structures were found in varying abundance ranging from 2.9% in HL-60 up to 17.7% in MOLM-13. Hybrid glycans were usually of low abundance in AML cell lines with KG-1 showing the lowest fractional abundance of 1.7% and U-937 the highest fractional abundance of 6.8%. Complex type glycans ranged from 14.4% in ML-1 to 53.3% in KG-1. Despite a broad overlap in the expression of abundant species, i.e., oligo- and paucimannose structures in virtually all of the studied AML cell lines, the fractional abundances varied greatly. On the contrary, complex type glycan structures showed greater diversity between the studied cell lines (Supplementary Figure S1) differing in the extent of sialylation, branching, fucosylation, and bisection.

To exemplify the qualitative and quantitative differences in *N*-glycosylation signatures, combined extracted ion current chromatograms (EICCs) obtained for the phenotypically distinct AML cell lines MOLM-13 and TF-1 are depicted in Figure 1. The MOLM-13 cell line is classified as an M5 type (acute monocytic leukemia) according to the FAB system, whereas TF-1 is assigned to the M6 type (acute erythroid leukemia). In Figure 1, complex type glycans were graphically separated in a left and a right panel to better illustrate the wealth of glycan species detected, although all *N*-glycans were measured simultaneously. As evident from the left panels, in both depicted cell lines the oligomannose structure comprising nine mannoses (H9N2) was the predominant glycan. However, the levels of phosphorylation (7.3%) and paucimannosidics (17.7%) are elevated in MOLM-13 compared to TF-1 (3.0% and 7.0%, respectively). Of note, MOLM-13 cells express three structural isomers of truncated hybrid type glycans (H4N3F1S1a, b, and c) with a cumulative fractional abundance of 2.2% that are absent in TF-1. Concerning complex type glycans (right panels in Figure 1), diantennary glycans with variable amounts of sialylation were the predominant structures in both cell lines (73.5% and 43.6% of complex type glycans in MOLM-13 and TF-1, respectively). In contrast to MOLM-13, TF-1 showed an enrichment in highly sialylated tri- and tetra-antennary structures (32.6% of complex type glycans in TF-1 and 25.7% in MOLM-13). Although both cell lines show core-fucosylated structures in similar abundance (approx. 34%), the amount of antennary fucosylation and therefore the abundance of (s)Le^x/a^ antigens was clearly upregulated in MOLM-13 cells (4.5%) compared to TF-1 (0.3%). Despite the high abundance of bisected glycans (Figure 1; light blue) in TF-1 (7.3%), these analytes were absent in MOLM-13. Overall, high sialylation was found in TF-1 cells as only a few nonsialylated terminal galactose moieties were detected.

### 3.2. O-Glycomics

After enzymatic removal of *N*-glycans, *O*-glycans were chemically released from glycoproteins and analyzed as described for *N*-glycans with some minor amendments to the instrument parameters. In total, 23 *O*-glycans were identified and relatively quantified in 21 AML cell lines (Supplementary Figure S3). In line with a previous report [34], the method showed good precision as exemplarily indicated by the average RSD of glycans ≥ 1.0% fractional abundance for technical (7.4%) and biological (6.4%) triplicates of MOLM-14 (Supplementary Figure S2).

**Figure 2:**
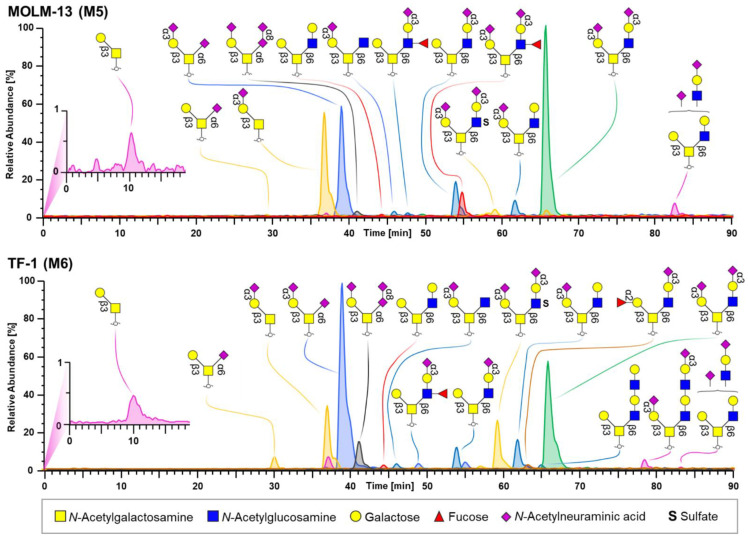
Exemplary combined EICCs of *O*-glycans obtained for the M5 subtype MOLM-13 (**upper panel**) and the M6 subtype TF-1 (**lower panel**). Individual mass traces are color coded. The symbols and colors of monosaccharides used for illustrating glycan structures are depicted beneath the panels. Identified glycosidic linkages are annotated.

In general, all of the AML cell lines showed high expression of core 1 structures, i.e., sialyl-3/6T antigen and disialyl-T antigen ranging from 26.8% in HL-60 to 82.3% in PLB-985. Additionally, core 2 glycans were identified at a fractional abundance from 17.5% in PLB-985 to 72.0% in HL-60. Core 3 and core 4 structures were not detected. Of note, diverging levels of H antigen expression, fucosylation, sulfation, and extension by LacNAc repeats were found. Mostly α-2,3 sialylation of terminal galactose residues was found next to lower levels of α-2,6 sialylation of the core N-acetylgalactosamine (GalNAc) and extension of sialic acids by α-2,8 sialylation. To illustrate these differences, exemplary EICCs of *O*-glycans obtained from MOLM-13 (M5; upper panel) and TF-1 (M6; lower panel) are shown in Figure 2. The main glycan structures (sialyl T antigen—H1N1S1, disialyl T antigen—H1N1S2 and disalylated core 2 glycan—H2N2S2) could be detected in both cell lines, albeit in differing abundances. MOLM-13 showed higher levels of H1N1S1 (21.1%) and H2N2S2 (35.9%), but lower amounts of H1N1S2 (20.7%), whereas TF-1 was found to express less H1N1S1 (14.3%) and H2N2S2 (25.5%), but more H1N1S2 (39.4%). Furthermore, sulfated glycans were elevated in TF-1 (2.9%) compared to MOLM-13 (1.7%), whereas α-1,3/α-1,4 fucosylated structures, i.e., Le^x/a^ and sLe^x/a^ antigens were prominent in MOLM-13 (3.9%). H antigen was absent in MOLM-13, yet detected in TF-1 with a fractional abundance of 0.7%. Interestingly, the pronounced expression of α-2,8 sialylation was detected in TF-1 at a fractional abundance of 5.4%.

### 3.3. Integrated N- and O-Glycomics

#### 3.3.1. Principal Component Analysis

**Figure 3:**
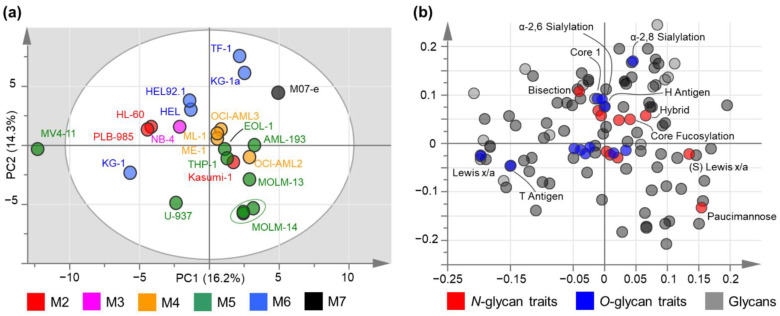
Unsupervised principal component analysis (PCA) of quantitative *N*- and *O*-glycomics data obtained from 21 AML cell lines. (**a**) Score plot of AML cell lines colored according to their FAB classification, as listed in Supplementary Table S1 [45,46]. Exemplarily, the MOLM-14 cell line was plotted as three independent biological replicates (green circle). (**b**) Corresponding loading plot depicting contributions of glycans and traits to the first and second principal component of the PCA. Individual glycans are colored in grey, whereas glycan types and derived traits are shown in red for *N*-glycans and in blue for *O*-glycans.

To assess whether the glycomic fingerprints of cell lines show associations with their AML class or a specific recurring mutation, we performed unsupervised PCA. Exemplarily, MOLM-14 was included in three biological replicates in the PCA (Figure 3a; green circle). Close clustering of these replicates indicates a low biological variation within these replicates compared to the variation observed between different cell lines. All of the cell lines were within the Hotelling’s T^2^ 95% with the exception of MV4-11, which seemed to differ pronouncedly in its glycomic phenotype.

First, we examined *N*- and *O*-glycomics separately to see if either one displayed pronounced grouping (data not shown). As both independent PCAs showed clustering of cell lines based upon their FAB classification, we continued to evaluate *N*- and *O*-glycomics in a combined manner (Figure 3). Notably, we did not observe any clear associations with their mutational status, as specified by the WHO classification (Supplementary Figure S4). However, this could be due to the fact that the majority of AML cell lines were classified as “not otherwise specified (NOS)”.

What stands out in the PCA are the FAB groups M4 (acute myelomonocytic leukemia), M5, and M6 comprising most of the investigated cell lines, which show an apparent separation in the first and second principal component (Figure 3a). AML cell lines of the M2 subtype (acute myeloblastic leukemia with maturation) seem to cluster less clearly: Although the M2 cell lines HL-60 and PLB-985 are located in the vicinity of M6 cells, Kasumi-1 cells (M2) comprised a distinct glycan repertoire more similar to the M5 cell lines. The M-07e cell line, which is classified as M7 subtype (acute megakaryoblastic leukemia) exhibited a quite unique glycomic signature based on its position in the score plot. Unfortunately, more general statements on this FAB class are not possible as M-07e was the only cell line investigated within this subtype. The M3 class (acute promyelocytic leukemia) appeared to have a similar glycomic signature as observed for the M6 cell lines. However, only one cell line of this specific subtype could be characterized limiting informative value for this FAB class. Pairs of related cell lines (derived from the same patient) such as HEL/HEL 92.1 and MOLM-13/MOLM-14 were located in each other’s vicinity suggesting similar glycosylation patterns. However, the KG-1 cell line and its less differentiated counterpart KG-1a showed a greater variation indicated by the increased distance in the score plot. This segregation is mainly driven by their differences in the core 1 to core 2 ratio of *O*-glycans (KG-1: 0.82 and KG-1a: 3.57), extension by LacNAc repeats (KG-1: 56.2% and KG-1a: 21.7%), and sLe^x/a^ expression (KG-1: 5.0% and KG-1a: 0.4%). Concerning *N*-glycans, the main drivers were found to be paucimannosidics (KG-1: 10.4% and KG-1a: 4.2%), core fucosylation (KG-1: 18.6% and KG-1a: 33.5%), and (s)Le^x/a^ (KG-1: 0.5% and KG-1a: 4.4%).

To gain better insights into what drives the discrimination of different FAB classes, we included—next to individual glycans—glycan types and derived traits from *N*-glycans (red) and *O*-glycans (blue), as specified earlier (Figure 3b, Supplementary Excel file). On the one hand, the evident separation of M6, M3, and most of M2 classes in the score plot could thus be attributed to increased bisection, core 1, T antigen, and Le^x/a^ (*O*-glycans) expression. On the other hand, M4 and especially M5 cell lines exhibit elevated levels of paucimannosidics, (s)Le^x/a^ (*N*-glycans), and hybrid type glycans.

#### 3.3.2. FAB-Grouped Glycan Features

**Figure 4:**
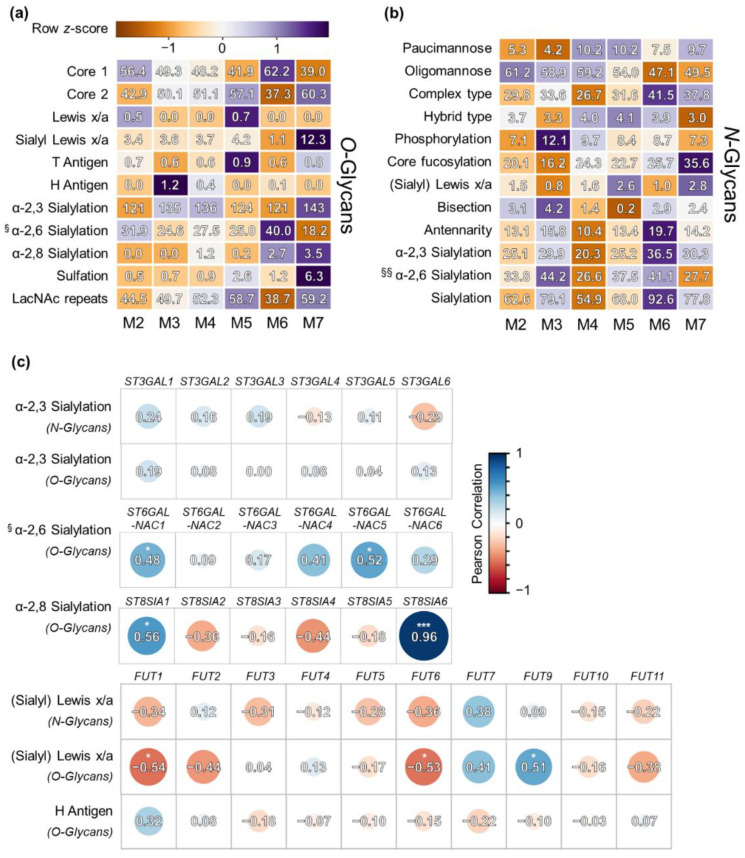
Overview of glycan types/derived traits and correlation analysis with GST expression data. In panel (**a**), *O*-glycan associated features are represented, whereas in panel (**b**), *N*-glycan derived features are depicted. The color scale represents the z-score after independent z-transformation of each glycan feature. The associated numeric values within the boxes show the average fractional abundance of derived traits and glycan types in a FAB group. ^§^ indicates α-2,6 sialylation on the core GalNAc of *O*-glycans and ^§§^ α-2,6 sialylation on terminal galactose residues of *N*-glycans. (**c**) Pearson correlation of selected glycan-antigens with transcriptomics data of relevant GSTs. Correlation coefficients are indicated by the size and color of the circles, as well as the associated numeric values. Significant values are marked (* for *p* ≤ 0.05, and *** for *p* ≤ 0.001).

As it was indicated from the PCA model that glycomic signatures associate with the phenotypic FAB class, relevant glycan epitopes and their variation across FAB classes were further explored (Figure 4). Importantly, sLe^x/a^ antigens were detected on both *N*- and *O*-glycans throughout all of the FAB classes. Yet, expression was highly variable on *O*-glycans ranging from 1.1% in M6 to a surprising 12.3% in the M7 cell line M-07e. (s)Le^x/a^ epitope expression on *N*-glycans varied less with the lowest expression in M3 (0.8%) and the highest in M7 (2.8%). H antigen was absent or of low abundance in most of the FAB classes.

Solely, the M3 type showed pronounced H antigen expression with 1.2% fractional abundance on *O*-glycans. In addition to *O*-glycan features, the M3 type showed unique abundances of *N*-glycan related features comprising the highest levels of phosphorylation (12.1%) and bisection (4.2%), but lowest levels of paucimannose (4.2%), core fucosylation (16.2%), and (s)Le^x/a^ (0.8%). In the context of sialylation, the highest levels of α-2,6 sialylation of the core GalNAc of *O*-glycans was found in the M6 subtype (40.0%), which is additionally reflected by the highest abundance of core 1 glycans (62.2%) within all of the FAB classes. Interestingly, α-2,8 sialylation of *O*-glycans could solely be detected on rather differentiated cell lines (M4 to M7), but was absent from cell lines belonging to the M2 and M3 class. *N*-glycan associated derived traits such as antennarity and sialylation (including α-2,3 and α-2,6) were of highest abundance in the M6 subtype (19.7% and 92.6%, respectively) compared to the lowest abundance that was found in M4 cell lines (10.4% and 54.9%, respectively). On top of the evidence presented in Figure 1 and Figure 2 as well as the PCA in Figure 3, the heat map visualizes strong differences between M4/M5 and the M6 subtype.

#### 3.3.3. Correlation of Glycan Features with GST Expression

Derived glycan traits were further examined by Pearson correlation analysis with transcriptomic data of GSTs obtained from public datasets (see Material and Methods, Figure 4c). We focused on several traits that could be products of a number of GST isoforms: α-2,3, α-2,6, and α-2,8 sialylation, (s)Le^x/a^, and H antigen. As GSTs may be rather specific for their substrates, we examined *N*- and *O*-glycans separately.

For α-2,3 sialylation, we detected rather weak correlations in both *N*- and *O*-glycans. The highest correlation values were obtained for *ST3GAL1* (r = 0.24 for *N*-glycans and r = 0.19 for *O*-glycans). A significant correlation was determined for α-2,6 sialylation of core GalNAc in *O*-glycans: *ST6GALNAC1* (r = 0.48) and *ST6GALNAC5* (r = 0.52) expression correlated well with this glycosylation trait. α-2,8 Sialylation of terminal sialic acids was associated highly significantly with the expression values of *ST8SIA6* (r = 0.96).

The highest positive correlation of fucosyltransferase expression with (s)Le^x/a^ abundance (*N*-glycans) was obtained for *FUT7* (r = 0.38), although it did not pass the significance threshold of *p* ≤ 0.05. (s)Le^x/a^ abundance (*O*-glycans) significantly correlated with *FUT9* (r = 0.51) and additionally showed a positive correlation with *FUT7* (r = 0.41). Both *FUT6* (r = −0.54) and *FUT1* (r = −0.54) showed a significant negative correlation with this trait in *O*-glycans. Lastly, H antigen expression correlated positively with *FUT1*, yet not significantly.

### 3.4. Association of Glycan Features with Hematopoietic TFs

**Figure 5:**
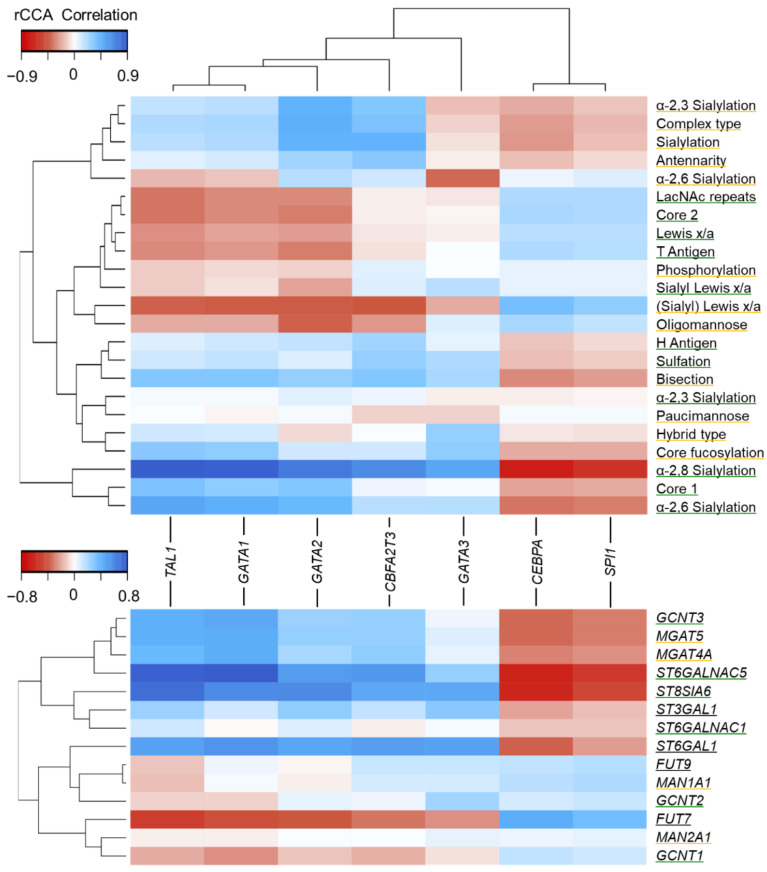
Regularized canonical correlation analysis (rCCA) of *N*- and *O*-glycan features and GSTs, respectively, with transcriptomics data of hematopoietic transcription factors. Correlation values are depicted in blue (positive correlation) and red (negative correlation), as illustrated in the provided color scale. Rows and columns were arranged based on hierarchical clustering. Glycan features and GSTs are underlined in yellow if related to *N*-glycans, in green if related to *O*-glycan or black if related to both.

To determine responsible factors further upstream of the presented glycomics data, we investigated amongst other potential candidates hematopoietic TFs. Based on current literature suggesting their involvement in AML pathobiology [47,48,49,50], we included *TAL1*, *GATA1-3*, *SPI1*, *CEPBA,* and *CBFA2T3*. Correlation of *N*- and *O*-glycan features with these TFs was assessed by regularized canonical correlation analysis (rCCA, Figure 5, Supplementary Table S2). Based on the hierarchical clustering analysis, two major groups of TFs were evident: *CEBPA*/*SPI1* and the remaining TFs, respectively. High *SPI1* and *CEPBA* expression correlated positively with several key glycan antigens such as *O*-glycan associated Le^x/a^, *N*-glycan associated (s)Le^x/a^, as well as T antigen. On the contrary, *TAL1*, *GATA 1,* and *GATA2* correlated negatively with these features. In particular, (s)Le^x/a^ on *N*-glycans correlated highly with most of the hematopoietic TFs investigated: *TAL1* (r = −0.42), *GATA1* (r = −0.44), *GATA2* (r = −0.44), *GATA3* (r = −0.46), *CBFA2T3* (r = −0.22), *CEBPA* (r = 0.40), and *SPI1* (r = 0.32). A strikingly high correlation was also observed for α-2,8 sialylation on *O*-glycans. *SPI1* and *CEBPA* correlated highly negatively with this glycomic signature (r = −0.65 and −0.74, respectively), whereas the other TFs showed highly positive correlation (r = 0.91 for *TAL1* and r = 0.89 for *GATA1*). Additionally, the total levels of sialylation including different linkages on both *N-*and *O*-glycans as well as associated traits, e.g., antennarity and complex type glycans correlated positively with *GATA2* and *CBFA2T3*. On the contrary, paucimannose, hybrid, phosphorylation, and α-2,3 sialylation on *O*-glycans showed rather weak associations with hematopoietic TFs.

Next, GST expression was included to assess correlations with said hematopoietic TFs (Figure 5) and to validate some of the associations reported earlier (Figure 4c). The highest correlations were observed for *ST8SIA6* with several TFs, e.g., r = 0.69 for *TAL1* and r = −0.59 for *CEBPA*, which were also matching well to the downstream-reported glycan signatures. In addition, *FUT7* showed pronounced correlation values ranging from r = −0.50 for *TAL1* to r = 0.43 for *CEBPA*. Potential downstream products, i.e., abundances of (s)Le^x/a^ epitopes on *N*- and *O*-glycans followed the trends observed for *FUT7* transcript levels. *FUT9* mainly showed the same associations as observed for its isoform *FUT7*, however, associations were less distinct. Although oligomannose and complex type glycans showed a moderate correlation with hematopoietic TFs, key mannosidases *MAN1A1* and *MAN2A1* showed only weak correlations with the TFs investigated. Additionally, the GSTs *MGAT5* and *MGAT4A* that are responsible for branching of *N*-glycans showed correlation values of r = 0.42 and r = 0.43 for *GATA1*, and r = 0.26 and r = 0.27 for *GATA2,* respectively, which was clearly reflected by the acquired glycomics data. The extent of α-2,3 sialylation on *N*-glycans followed the same association patterns. In addition, *TAL1* and *GATA1* showed pronounced associations with *ST6GALNAC5* with correlation values of r = 0.75 and r = 0.77, respectively. Out of the three GlcNAc transferases responsible for core 2 synthesis, only *GCNT3* showed a major association in the rCCA (r = 0.44 for *GATA1*). Intriguingly, the expression of *ST6GAL1* was highly correlated with most of the TFs, however, the expression values were not well reflected by the glycomics data. In Supplementary Figures S5 and S6, we integrated the data on *N*- and *O*-glycomics, corresponding GSTs, and correlated hematopoietic TFs for the glycomic-wise distinct subtypes M5 and M6.

## 4. Discussion

In this study, we explored the *N*- and *O*-glycome of 21 widely used AML cell lines that cover most of the genetic and phenotypical diversity encountered in AML. Exploiting the major advantages of the PGC nano-LC-MS^2^ platform, namely its excellent separation power for glycan isomers and the in-depth structural characterization provided by fragmentation in negative ion mode, we assessed a plethora of glycan species and obtained quantitative information.

First, we assessed the *N*-glycome of AML cell lines. As illustrated in Figure 1 and Figure 4, the four major *N*-glycan types (oligomannose, paucimannose, hybrid, and complex) could be identified in all of the cell lines, albeit in drastically varying abundances. Oligomannose type structures were of high abundance in all of the AML cell lines (average abundance of 54.4%). The high prevalence of this glycan type was previously observed for a small number of AML cell lines [27], in addition to other cancer types such as colorectal, breast, and pancreatic cancer, and may represent a general feature of highly proliferative cancer cells [51,52,53]. A pan-AML feature of the *N*-glycome was the strong expression of paucimannosidics (Figure 1 and Figure 4) with a mean fractional abundance of 8.57% across all of the investigated cell lines. Although only few studies have considered this glycan type in the context of cancer, a recent meta-study investigated paucimannose expression across various cancer entities and reported elevations in a number of human cancers [54]. Notably, also two AML cell lines (HL-60 and THP-1) were investigated in this study, which differed by strongly elevated levels of paucimannosidics in the M5 cell line THP-1 compared to the M2 cell line HL-60 (original data from [55]). These findings could be substantiated by our data as we observed 2.9% paucimannose glycans in HL-60 compared to 9.1% in THP-1, albeit we only detected three out of four paucimannose structures reported in this study. Of interest, paucimannose structures were identified in the PCA loading plot (Figure 3b) as one of the most pronounced factors driving the discrimination of AML cells according to the FAB class and were especially enriched in the M4 and M5 subtypes.

Contrary to the high complexity across different cell lines observed for *N*-glycans, we observed a limited qualitative variation of *O*-glycan structures (Supplementary Figure S3). In total, a cumulative number of 23 *O*-glycans belonging either to the core 1 or core 2 type were identified across all of the AML cell lines. Interestingly, abundant α-2,8 sialylation was found on core 1 structures, which was predominantly expressed by M6 and M7 AML cell lines correlating highly significantly with the expression of *ST8SIA6*. To date, little is known about the implication of *O*-glycans carrying α-2,8 sialylation in cancer. However, the role of *ST8SIA6* in tumor immune evasion is currently investigated (grant number: R01-CA243545-01A1) [56]. In this context, Ma et al. attributed acquired chemoresistance in AML cell lines to elevated levels of *ST8SIA4* and suggested *N*-glycans as the dominant effectors [27]. Notably, they could detect increased levels of *ST8SIA6* in chemo-sensitive AML cell lines.

Quantitative *N*- and *O*-glycan data were combined and evaluated by means of PCA (Figure 3). Indeed, we found a clear association between the AML glycomes and their FAB class. In particular, the FAB classes comprising most of the cell lines investigated (M4, M5, and M6) were clustering in a distinct manner in the score plot. Since the FAB classification is mainly based on the phenotypic appearance of AML blasts and this is related to the differentiation status of AML, this association appears plausible. Of note, while most of the cell lines are clearly classified as one FAB type and we based our assignments on highly credible literature (Supplementary Table S1), the classification of some cell lines, i.e., KG-1 and KG-1a, may be ambiguous. In agreement with the NCBI BioSample and Drexler et al. [45], KG-1 and the derived KG-1a cell line were classified as an M6 subtype in this study. Apparent from the PCA score plot and the heat map presented in Figure 4, we detected clearly elevated levels of highly sialylated and branched complex type glycans in the M6 group compared to M4 and M5. On the other hand, (s)Le^x/a^ expression on *N*- and *O*-glycans was higher in the M5 subtype. In our opinion, these associations of the FAB system and glycosylation are highly interesting as glycan antigens may soon serve a prognostic function in AML similar to what is observed for other cancer entities [57,58,59]. Having the prognostic value of the FAB classification for some AML patients in mind [60], this correlation may be a first indication into this direction.

The sLe^x^ and its isomer sLe^a^ are well-known tumor-associated antigens with implications in metastasis as well as immunomodulation, and are often associated with poor prognosis [14,61]. As described earlier, the implications of sLe^x^ in AML pathobiology may be fundamental. Therefore, a major finding was that the (s)Le^x/a^ antigen was abundantly expressed in all of the investigated AML cell lines. However, the two isomers (s)Le^x^ and (s)Le^a^, which are characterized by the α-1,4 linkage and the α-1,3 linkage of the fucose, respectively, cannot be easily discriminated. Therefore, we performed correlation analyses to see whether their expression correlates with *FUT3* ((s)Le^a^) or *FUT4-7, 9* ((s)Le^x^), as depicted in Figure 4c. Since no major positive correlation was found between *FUT3* and (s)Le^x/a^ epitopes present on *N*- and *O*-glycans, we assume that the reported glycans mainly carry the (s)Le^x^ isomer. This is further supported by the positive correlation of (s)Le^x/a^ expression with FUT7 in the *N*-glycan derived trait and with *FUT7,* as well as *FUT9* in the *O*-glycan derived trait.

To our knowledge, sLe^x^ expression on AML cells was to date only studied by specific monoclonal antibodies and subsequent flow cytometric analysis [20,23,62]. In this study, the dominant glycan structures that carry these essential glycan antigens are described. (s)Le^x^ was expressed throughout all of the AML cell lines investigated albeit in varying abundance (Figure 4). In particular, FAB types M5 showed high levels of (s)Le^x^, in contrast to M6 subtypes. Of note, (s)Le^x^ epitopes were present on both *N*-glycans as well as *O*-glycans further complicating the picture as both types of protein glycosylation may be responsible for its reported implications.

The role of sLe^x^ (also termed sCD15) in AML is intriguing: The expression of sLex appears to be a feature of the low differentiation status of AML blasts. Upon the induced differentiation of the AML cell line THP-1, the expression of sLe^x^ antigens on its surface was lost [62]. The Le^x^ epitope, however, was reported to be largely unaltered. Furthermore, a recent report by Barbier et al. could demonstrate that AML blasts induce an upregulation of E-selectin expression in cells of their microenvironment [20]. Upon interaction of sLe^x^ with these receptors, pro-survival pathways, i.e., AKT/NF-κB, were activated leading to enhanced chemoresistance of these leukemic blasts. Disruption of said interaction in the AML niche by the glycomimetic drug Uproleselan or genetic deletion of E-selectin in a murine model reportedly abolished this effect. In addition, they could show that disruption of E-selectin binding reduced the proportion of quiescent AML blasts significantly. Additionally, high expression of sLe^x^ associated GST *FUT7* and *ST3GAL4* has already been linked to a dismal prognosis in AML patients [63]. Our data suggest that sLe^x^ is expressed in a wide variety of different AML subtypes as specified by FAB or WHO classification, though to a different extent. Therefore, targeting chemoresistance by interruption of the sLe^x^—E-selectin axis may prove an efficient mode of action in fighting AML and is currently investigated in clinical trials [21]. Taking these findings together, sLe^x^ epitope expression may shape the pathology of AML in a profound manner.

The hematopoietic TFs *TAL1, GATA1-3, SPI1, CEBPA,* and *CBFA2T3* were found to significantly associate with several glycan features as well as upstream GST expression, as assessed by rCCA (Figure 5). In general, these TFs are crucial for stem cell maintenance as well as correct hematopoiesis and aberrant expression along with frequent mutation have been observed in AML [47,48,49,50]. First, associations between glycan features and these TFs were determined. An astoundingly high correlation of key glycan antigens such as (s)Le^x/a^, α-2,8 sialylation, and *N*-glycan sialylation levels could be observed for many of the investigated TFs. On the one hand, *SPI1* and *CEBPA* showed similar correlation values with glycan features as indicated in the hierarchical clustering analysis. Importantly, these two TFs were previously found to be expressed in lower levels in hematopoietic stem cells (HSCs) [64]. The lowest expression values for these two TFs in our AML cell line panel were observed in M6 and M7 subtypes (Supplementary Excel file). On the other hand, *TAL1*, *GATA1-2,* and *CBFA2T3*, a set required for the maintenance of both HSC and leukemic stem cells (LSCs) [47,48,49] were grouped in the hierarchical clustering. Although similar glycan antigens showed high correlations with these TFs, for most of the glycan features we observed opposing correlations compared to *SPI1/CEBPA*. Moreover, this set of TFs was found to be substantially enriched in M6 and M7 cell lines in our dataset (Supplementary Excel file). *GATA3* showed high associations with several glycan features, however, in a pattern distinct from the one observed for the two main groups stated earlier.

To corroborate our findings, we included rCCA of said TFs with the expression of relevant GSTs that relate to these altered glycan traits. As anticipated from the analysis of downstream glycan traits, GST expression showed a high correlation with hematopoietic TFs. For instance, *FUT7* expression correlated highly negatively with *SPI1/CEBPA* and concomitantly correlated negatively with the observed (s)Le^x/a^ expression in AML cell lines. *TAL1, GATA1-3,* and *CBFA2T3* showed opposing correlations with *FUT7*, again supported by the downstream abundance of glycan (s)Le^x/a^ antigens. In addition, *ST8SIA6* correlated highly positively with *TAL1* and negatively with *CEBPA/SPI1,* reflected concurrently by the abundance of this epitope in the glycomics data.

Furthermore, traits linked to increased sialylation and branching of *N*-glycans, i.e., antennarity, complex type, total sialylation, and α-2,3 sialylation, were grouped closely in the hierarchical clustering and correlated positively with *GATA2* and *CBFA2T3*. Concomitantly, we detected positive associations with the expression of branching enzymes *MGAT5* and *MGAT4A*. These traits associated with invasiveness and metastasis in many cancers [15] have been recently linked to increased *GATA2* as well as *GATA3* expression after induced hypomethylation by hypoxia or hypomethylating agents in ovarian and breast cancer [65,66]. Notably, these two reports could also link increased *GATA2-3* to elevated levels of *MGAT5* and *ST3GAL4* and reported downstream changes on the glycan level. While we could substantiate the findings for *GATA2* in our study, we did not observe clear associations of *GATA3* with extensive branching of *N*-glycans in the case of AML. Considering these findings, hematopoietic TFs may substantially regulate the differentiation status of AML blasts alongside their cytochemical characteristics as classified by FAB and, thus, may substantially shape their glycomic phenotype.

To summarize these observations, altered *N*- and *O*-glycan biosynthetic pathways including corresponding GSTs and hematopoietic TFs were compiled (Supplementary Figures S5 and S6). In these two overviews, we propose a model of how hematopoietic TFs may lead to increased or decreased levels of specific GSTs and how this translates into distinct glycomic fingerprints, as observed for AML cell lines belonging to the divergent M5 and M6 subtypes, respectively.

A potential limitation of our study is being solely focused on cell line models that may not necessarily recapitulate the molecular phenotype encountered in an in vivo scenario. Nonetheless, Sandberg et al. have indicated the translational value of these cellular models by comparing the cell lines to primary cancer tissue based on expression data for several cancer entities including leukemic cell lines [67]. Nevertheless, a direct comparison of AML cell line glycomes and that of primary blasts would be essential. However, the poor accessibility of primary AML blasts that reside predominantly in the bone marrow and the varying cellular purity of bone marrow needle aspirates hindered us from performing that kind of study at this point. In the future, this problem may be circumvented by establishing suitable purification strategies for AML blasts such as fluorescence-activated cell sorting.

Moreover, this glycomics-centered study does not intend to provide information on specific glycosylation sites within proteins and their respective glycan structures. Therefore, a future glycoproteomics study would be vital to further unravel the role of global protein glycosylation and identify critical protein glycoforms involved in AML pathobiology. Since glycoproteomics often lack the ability to identify the structural details of glycans present on a glycopeptide, the in-depth structural characterization performed in this study may even constitute the basis for a prospective glycomics-assisted glycoproteomics study [68].

## 5. Conclusions

Although a lot of evidence for the involvement of aberrant protein glycosylation in AML has been gathered, a global exploratory study on the *N*- and *O*-glycome of widely used AML cell lines has been missing to date. Here, we show an in-depth qualitative and quantitative glycomic characterization of 21 AML cell lines and provide protein glycosylation signatures as a valuable resource for further research. These glycomic fingerprints expressed by AML cell lines could be associated with their phenotypic and cytochemical characteristics, as classified by the FAB system. In addition to other important glycan antigens, a number of glycan structures (both *N*- and *O*-linked) were described that carry the (s)Le^x/a^ antigen, which has profound implications in chemoresistance, metastasis, and immunomodulation in AML, and is currently of high interest with several clinical trials registered. In this regard, striking differences in the expression levels of these cancer-associated antigens across different FAB subtypes could be reported. By integrating our glycomics data with transcriptomics data from public repositories, we could propose the involvement of specific GSTs in the expression of certain glycan epitopes. Eventually, we provide evidence for the upstream involvement of hematopoietic TFs in the glycosylation machinery that are both found severely dysregulated in AML.

## Data Availability

The raw mass spectrometric data files that support the findings of this study are available in GlycoPOST (accession number: GPST000214) [69].

## Supplementary Materials

The following are available online at https://www.mdpi.com/article/10.3390/cells10113058/s1. Supplementary Information 1, Supplementary Information 2, Supplementary Excel file, Supplementary Figure S1: Overview of N-glycans identified in AML cell lines; Supplementary Figure S2: Technical and biological variation of N- and O-glycomics; Supplementary Figure S3: Overview of O-glycans identified in AML cell lines; Supplementary Figure S4: Association of mutational status and glycomic signature; Supplementary Figure S5: Overview of predominantly altered N-glycan biosynthesis pathways; Supplementary Figure S6: Overview of predominantly altered O-glycan biosynthesis pathways; Supplementary Table S1: Overview of investigated cell lines and their FAB-classification; Supplementary Table S2: rCCA-correlation values.
