# Supplementary material for: Integrated N- and O-Glycomics of Acute Myeloid Leukemia (AML) Cell Lines
Source: Cells. 2021 Nov 6;10(11):3058. doi: 10.3390/cells10113058 (PMC8616353; doi:10.3390/cells10113058)
Supplement: Supplementary file 1 [file cells-10-03058-s001.zip › Supplementary_Figures_Tables.pdf]

Supplementary Figures and Tables

Integrated *N*- and *O*-Glycomics of Acute Myeloid Leukemia (AML) Cell Lines

Constantin Blöchl [1,2], Di Wang [1], Katarina Madunic [1], Guinevere S. M. Lageveen-Kammeijer [1], Christian G. Huber [2,3,*], Manfred Wuhrer [1,*] and Tao Zhang [1,*]

[1] *Center for Proteomics and Metabolomics, Leiden University Medical Center, Albinusdreef 2, 2333 ZA Leiden, the Netherlands; constantin.bloechl@sbg.ac.at, d.wang@lumc.nl, k.madunic@lumc.nl, g.s.m.kammeijer@lumc.nl, m.wuhrer@lumc.nl, t.zhang@lumc.nl*

[2] *Department of Biosciences, University of Salzburg, Hellbrunnerstrasse 34, 5020 Salzburg, Austria; constantin.bloechl@sbg.ac.at, c.huber@sbg.ac.at*

[3] *Cancer Cluster Salzburg, Department of Biosciences, University of Salzburg, Hellbrunnerstrasse 34, 5020 Salzburg, Austria; c.huber@sbg.ac.at*

**Correspondence: c.huber@sbg.ac.at, m.wuhrer@lumc.nl, t.zhang@lumc.nl*

Table of Contents

Figure S1: Overview of *N*-glycans identified in AML cell lines

Figure S2: Technical and biological variation of *N*- and *O*-glycomics

Figure S3: Overview of *O*-glycans identified in AML cell lines

Figure S4: Association of mutational status and glycomic signature of AML cell lines

Figure S5: Overview of altered *N*-glycan biosynthesis in AML subtypes M5 and M6

Figure S6: Overview of altered *O*-glycan biosynthesis in AML subtypes M5 and M6

Supplementary Table S1: Overview of investigated cell lines and their FAB classification

Supplementary Table S2: rCCA-correlation values corresponding to Figure 5

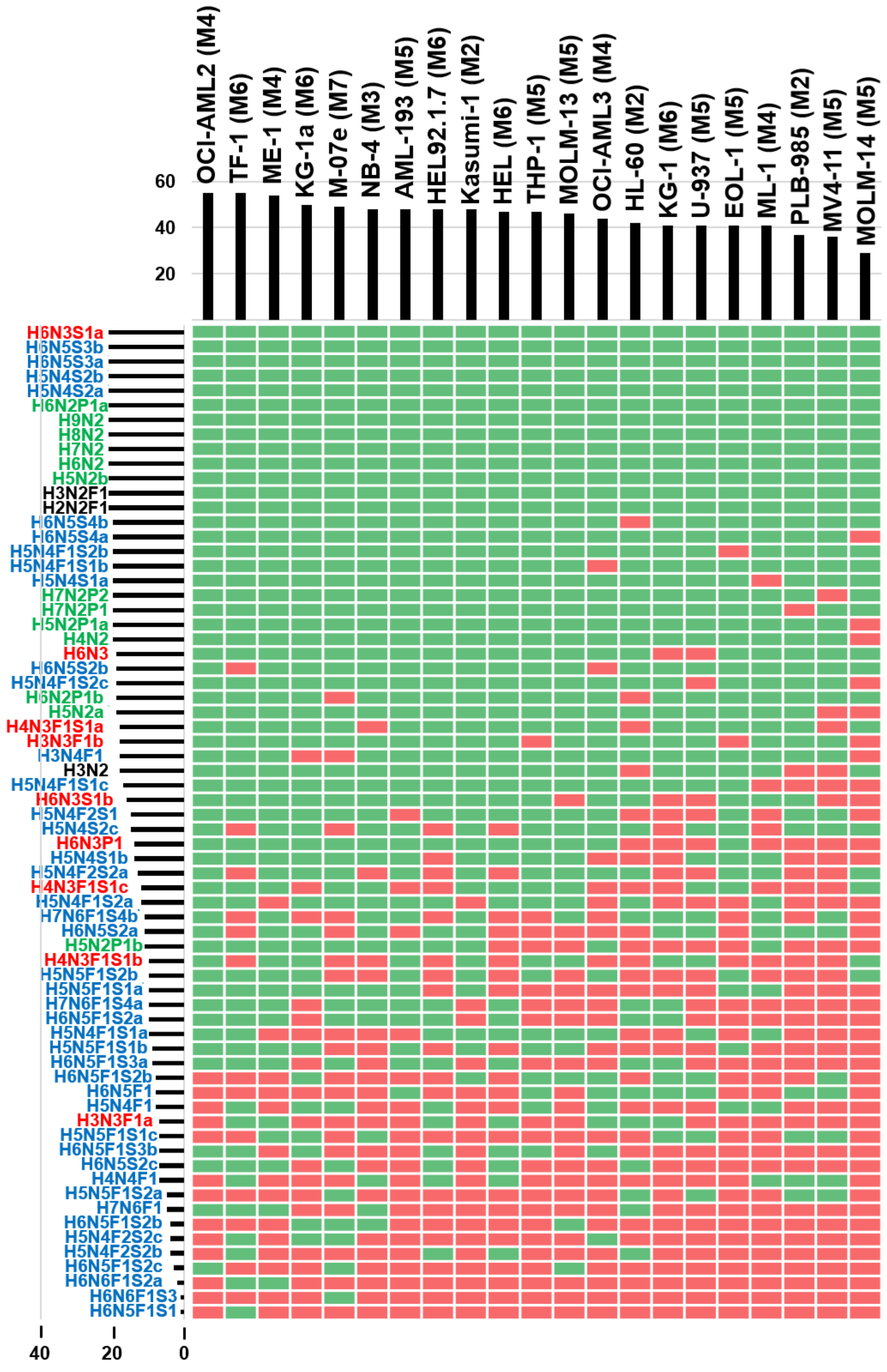

Figure S1. Overview of *N*-glycans identified in AML cell lines. Cell lines are plotted against glycan structures (green (identified) and red (not identified)). Glycans are abbreviated according to their monosaccharide composition: hexose (H), *N*-acetylhexosamine (N), fucose (F), *N*-acetylneuraminic acid (S), and phosphorylation (P). The number of glycans identified per cell line is depicted in a horizontal bar graph, whereas the number of cell lines in which a specific glycan has been observed in a vertical bar graph. Glycans are colored according to glycan types: oligomannose (dark green), paucimannose (black), hybrid type (red) and complex type (blue). Structures of identified *N*-glycans are provided in Supplementary Information 1.

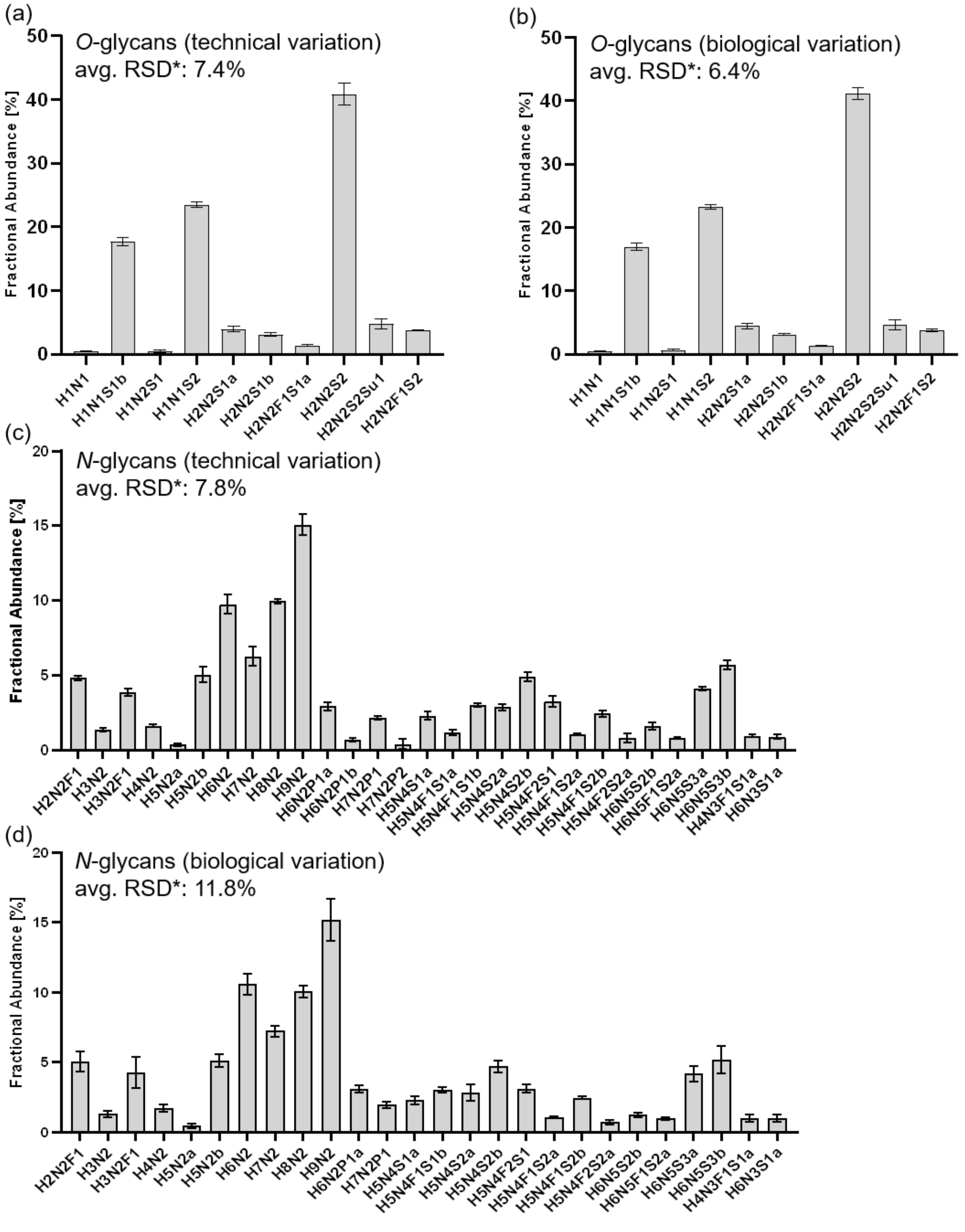

Figure S2. Technical and biological variability exemplarily assessed for the MOLM-14 cell line. Fractional abundances of identified *O*-glycans and corresponding standard deviations of (a) three technical replicates and (b) three biological replicates of the MOLM-14 cell line, respectively, are shown. Similarly, fractional abundances of identified *N*-glycans and corresponding standard deviations of (c) three technical replicates and (d) three biological replicates of the MOLM-14 cell line, respectively, are plotted. Glycan structures are provided in Supplementary Information 1 and 2. RSD* denotes average relative standard deviations determined from glycans ≥ 1.0% fractional abundance.

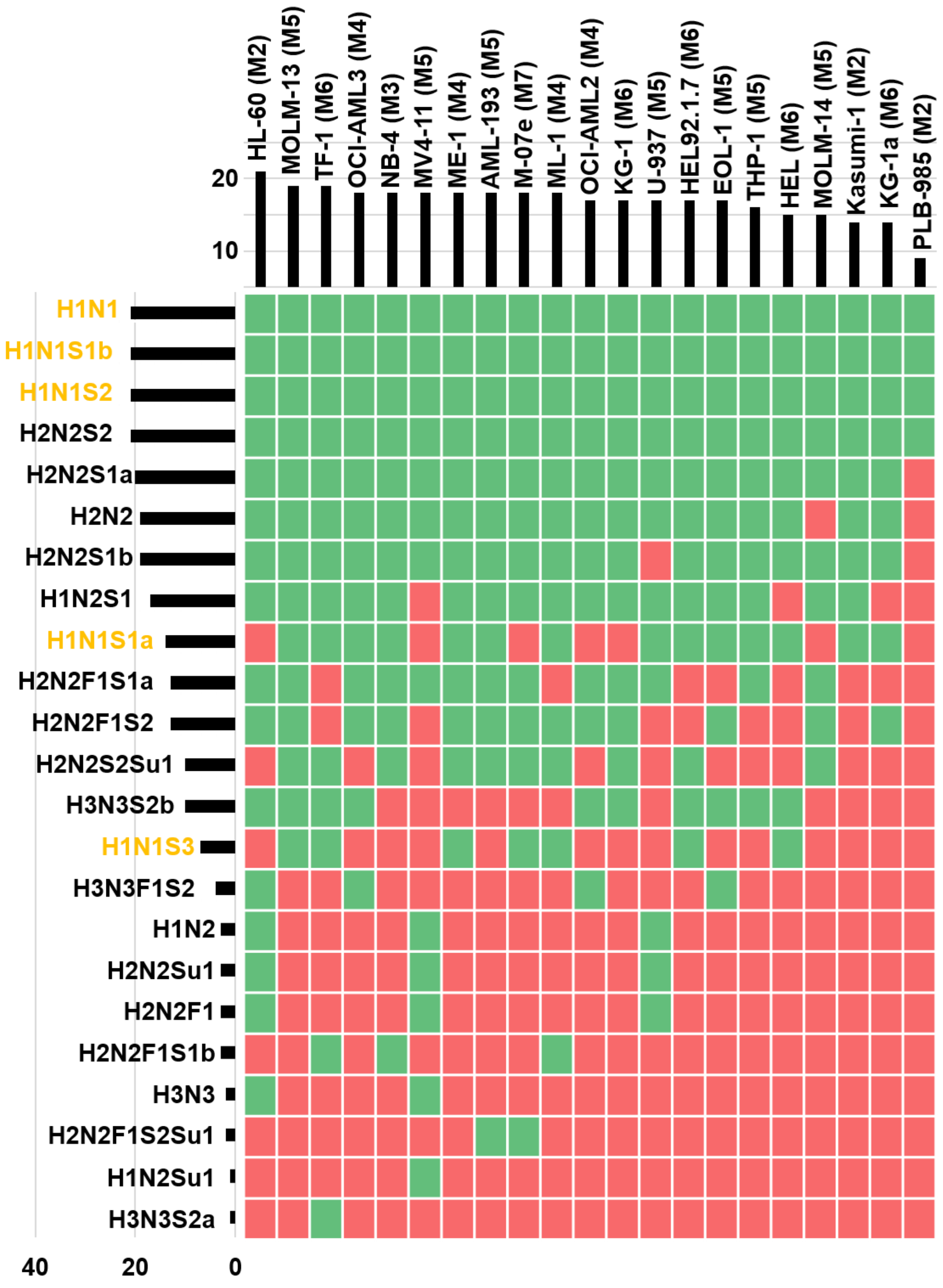

Figure S3. Overview of *O*-glycans identified in AML cell lines. Cell lines are plotted against glycan structures (green (identified) and red (not identified)). The number of glycans identified per cell line is depicted in a horizontal bar graph, whereas the number of cell lines in which a specific glycan has been observed in a vertical bar graph. Glycans are abbreviated according to their monosaccharide composition: hexose (H), *N*-acetylhexosamine (N), fucose (F), *N*-acetylneuraminic acid (S), and sulfation (Su). Core 1 structures are marked in yellow and core 2 structures in black. Structural information of identified glycans are provided in Supplementary Information 2.

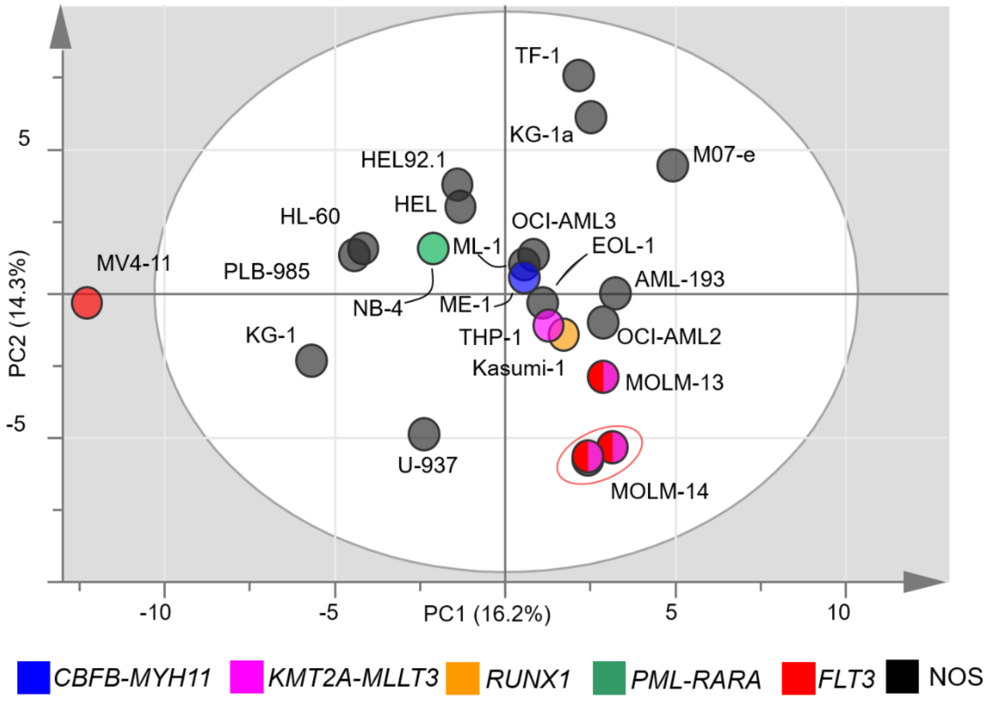

Supplementary Figure S4. Association of mutational status and glycomic signature of AML cell lines. Unsupervised principal component analysis (PCA) of quantitative *N*- and *O*-glycomics data obtained from 21 AML cell lines. Score plot of AML cell lines colored according to recurring genetic abnormalities as specified in the WHO classification including *FLT3* mutation or not otherwise specified (NOS). Exemplarily, the MOLM-14 cell line was plotted as three independent biological replicates.

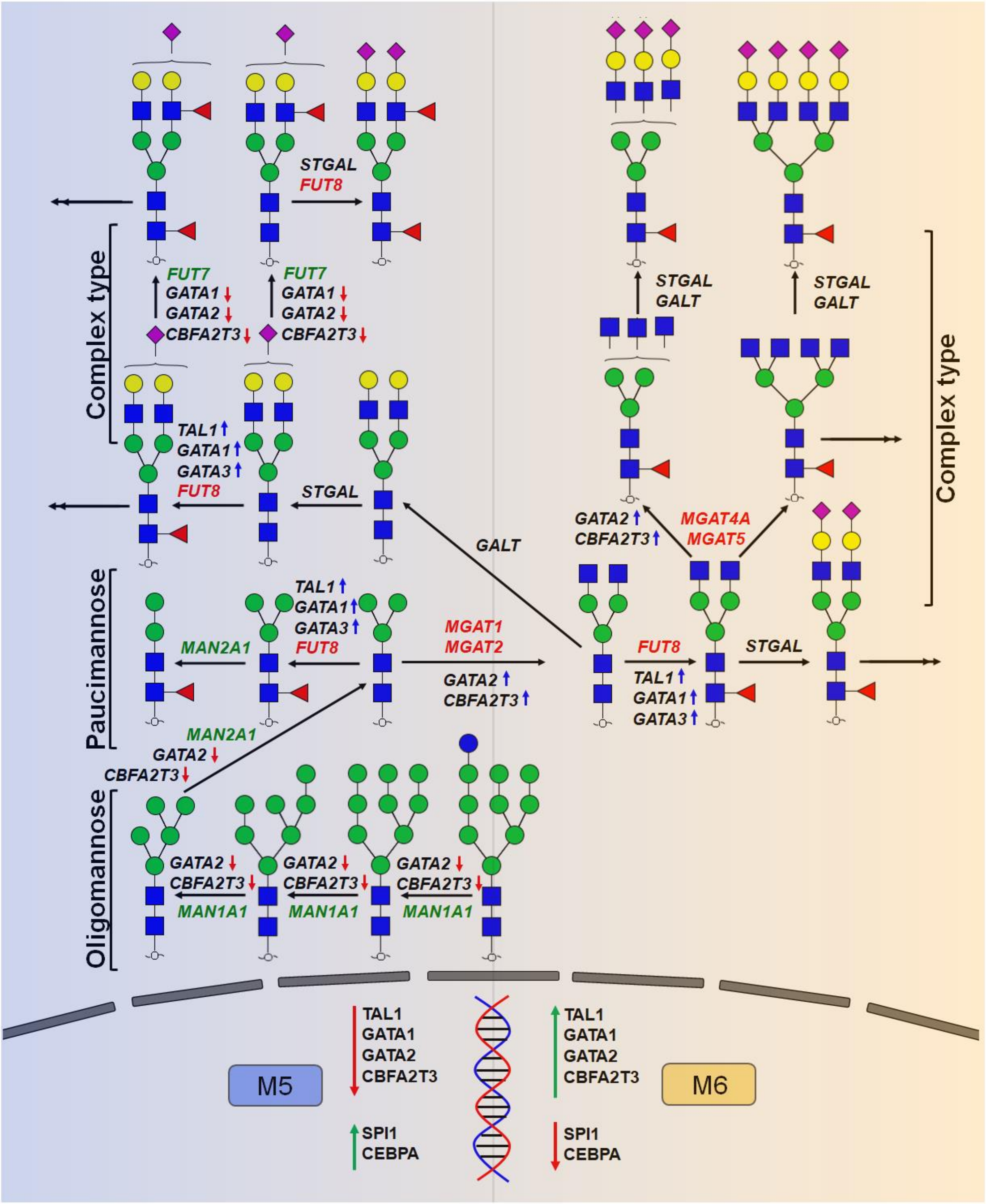

Supplementary Figure S5. Overview of predominantly altered *N*-glycan biosynthesis steps in AML subtypes M5 and M6. Glycan structures associated with derived traits upregulated in M5 (left, blue) or M6 cells (orange, right) based on acquired glycomics data are depicted. Corresponding glycosyltransferase genes are colored in green if upregulated in M5 or colored in red if they are upregulated in M6. In addition, differences in expression of hematopoietic TFs between M5 and M6 are shown. Hematopoietic TFs are indicated if they are strongly positively (upwards facing blue arrow) or negatively correlated (downwards facing red arrow) with the expression of a specific glycan trait. Double arrows indicate structures that may be substrate to further extension by glycosyltransferases. Note that this model does not claim to be holistic.

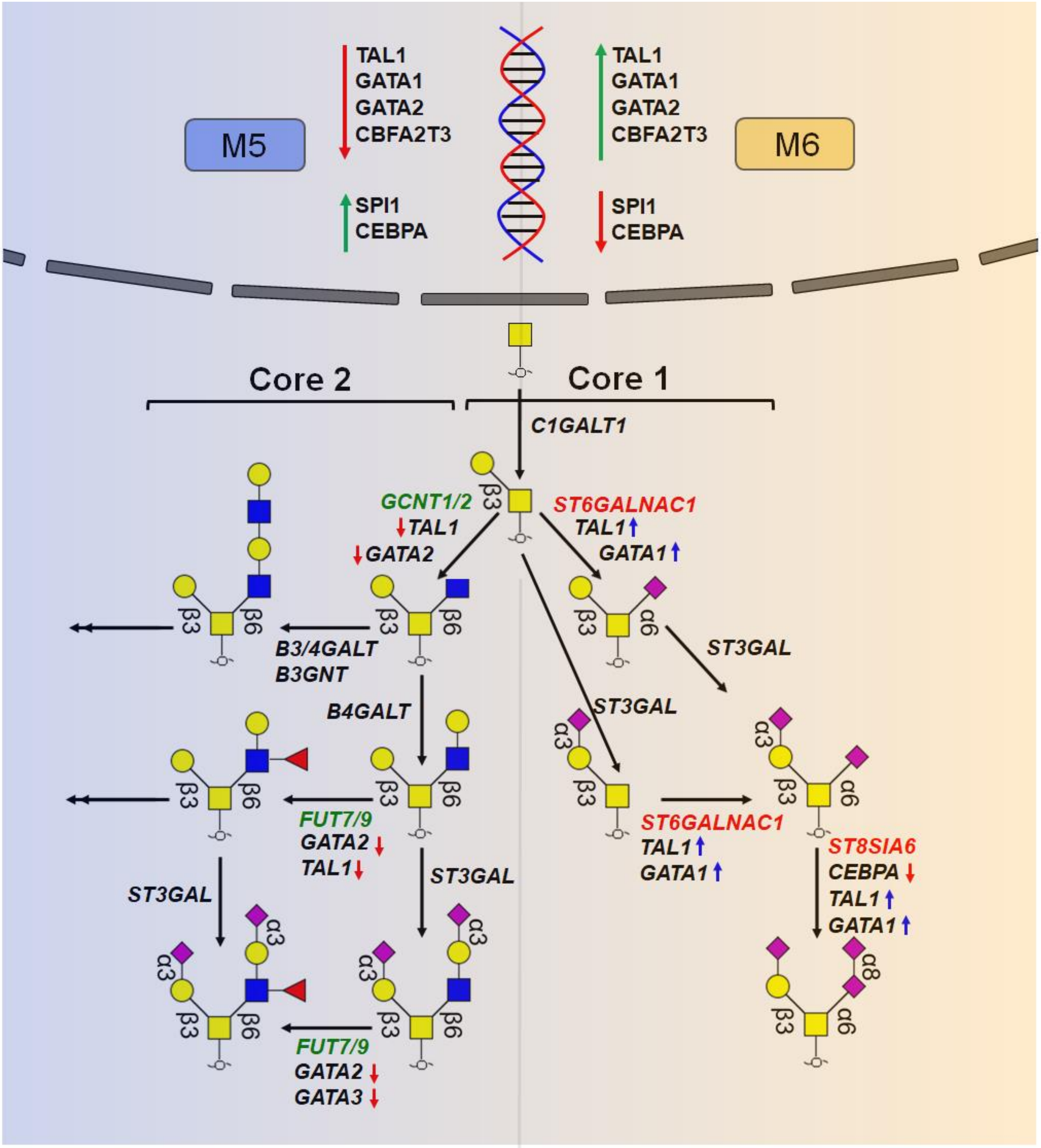

Supplementary Figure S6. Overview of predominantly altered *O*-glycan biosynthesis in AML subtypes M5 and M6. Glycan structures associated with derived traits upregulated in M5 (left, blue) or M6 cells (orange, right) based on acquired glycomics data are depicted. Corresponding glycosyltransferase genes are colored in green if upregulated in M5 or colored in red if they are upregulated in M6. In addition, differences in expression of hematopoietic TFs between M5 and M6 are shown. Hematopoietic TFs are indicated if they are strongly positively (upwards facing blue arrow) or negatively correlated (downwards facing red arrow) with the expression of a specific glycan trait. Double arrows indicate structures that may be substrate to further extension by glycosyltransferases. Note that this model does not claim to be holistic.

Supplementary Table S1. Overview of investigated cell lines and their FAB-classification according to literature (footnotes).

Nr.	Cell line	FAB	NCBI BioSample	Note
**1**	HL-60	M2[1,2,3]	SAMN10988245	
**2**	Kasumi-1	M2[1,2,3]	SAMN10988020	
**3**	PLB985	M2[3]	-	Sister cell line of HL-60
**4**	NB-4	M3[1,2,3]	SAMN10988571	
**5**	ME-1	M4[1,2,3]	SAMN10988452	
**6**	ML-1	M4[2,3]	-	
**7**	OCI-AML2	M4[1,2,3]	SAMN10988499	
**8**	OCI-AML3	M4[1,2,3]	SAMN10988470	
**9**	EOL-1	M5[1,3]	SAMN10988490	
**10**	AML-193	M5[1,2,3]	SAMN10987604	
**11**	MOLM-13	M5[1,2,3]	SAMN10988379	
**12**	MOLM-14	M5[3]		Sister cell line of MOLM-13
**13**	MV4-11	M5[1,2,3]	SAMN10988366	
**14**	THP-1	M5[1,2,3]	SAMN10987847	
**15**	U937	M5[3]	-	
**16**	KG-1	M6[1,2]	SAMN10988135	
**17**	KG-1a	(M6)	-	Sister cell line of KG-1
**18**	HEL	M6[1,2,3]	SAMN10987697	
**19**	HEL 92.1.7	M6[1]	SAMN10987842	Sister cell line of HEL
**20**	TF-1	M6[1,2,3]	SAMN10988443	
**21**	M-07e	M7[1,2,3]	SAMN10988568	

[1] FAB classification as listed in NCBI´s BioSample database. [2] Drexler, H.G. The leukemia-lymphoma cell line factsbook; Academic Press: 2000. [3] FAB classification as stated in: Quentmeier, H.; Reinhardt, J.; Zaborski, M.; Drexler, H.G. FLT3 mutations in acute myeloid leukemia cell lines. Leukemia ***2003****, 17, 120-124, doi:10.1038/sj.leu.2402740.*

Supplementary Table S2. rCCA of hematopoietic TFs with glycan features and glycosyltransferases, respectively. Correlation values and their order correspond to the heat map shown in Figure 5. In the upper panel the correlation values of hematopoietic TFs with glycan features are plotted. In the lower panel the correlation values of these TFs with glycosyltransferases are shown. Glycan features and glycosyltransferases are underlined if likely related to *N*-glycans (yellow), *O*-glycans (green), or both (black).

	**TAL1**	**GATA1**	**GATA2**	**CBFA2T3**	**GATA3**	**CEBPA**	**SPI1**
**α-2.3 Sialylation**	0.17	0.19	0.47	0.36	-0.17	-0.23	-0.15
**Complex type**	0.22	0.24	0.47	0.38	-0.11	-0.27	-0.19
**Sialylation**	0.18	0.22	0.46	0.47	-0.09	-0.27	-0.18
**Antennarity**	0.08	0.12	0.27	0.34	-0.04	-0.16	-0.10
**α-2.6 Sialylation**	-0.19	-0.16	0.21	0.13	-0.41	0.04	0.10
**LacNAc repeats**	-0.37	-0.32	-0.32	-0.05	-0.06	0.23	0.23
**Core 2**	-0.38	-0.32	-0.35	-0.04	-0.03	0.24	0.23
**Lewis x/a**	-0.29	-0.25	-0.26	-0.06	-0.05	0.19	0.18
**T Antigen**	-0.32	-0.28	-0.35	-0.09	0.00	0.23	0.21
**Phosphorylation**	-0.14	-0.10	-0.11	0.08	0.02	0.06	0.07
**Sialyl Lewis x/a**	-0.14	-0.09	-0.24	0.09	0.19	0.06	0.07
**(Sialyl) Lewis x/a**	-0.42	-0.44	-0.44	-0.46	-0.22	0.40	0.32
**Oligomannose**	-0.23	-0.23	-0.43	-0.28	0.10	0.24	0.18
**H Antigen**	0.10	0.13	0.17	0.27	0.07	-0.15	-0.10
**Sulfation**	0.13	0.17	0.09	0.30	0.22	-0.17	-0.13
**Bisection**	0.35	0.36	0.30	0.36	0.25	-0.31	-0.26
**α-2.3 Sialylation**	0.03	0.03	0.09	0.05	-0.05	-0.04	-0.03
**Paucimannose**	0.00	-0.02	0.04	-0.13	-0.12	0.03	0.02
**Hybrid type**	0.13	0.12	-0.10	0.02	0.30	-0.06	-0.08
**Core fucosylation**	0.33	0.31	0.14	0.14	0.31	-0.23	-0.22
**α-2.8 Sialylation**	0.91	0.89	0.75	0.66	0.54	-0.74	-0.65
**Core 1**	0.38	0.32	0.36	0.05	0.03	-0.24	-0.23
**α-2.6 Sialylation**	0.53	0.48	0.43	0.19	0.21	-0.37	-0.35

	**TAL1**	**GATA1**	**GATA2**	**CBFA2T3**	**GATA3**	**CEBPA**	**SPI1**
**GCNT3**	0.42	0.44	0.24	0.26	0.05	-0.34	-0.30
**MGAT5**	0.41	0.42	0.26	0.26	0.09	-0.34	-0.29
**MGAT4A**	0.36	0.43	0.21	0.27	0.05	-0.29	-0.25
**ST6GALNAC5**	0.75	0.77	0.51	0.52	0.25	-0.62	-0.51
**ST8SIA6**	0.69	0.56	0.58	0.44	0.44	-0.59	-0.46
**ST3GAL1**	0.24	0.12	0.27	0.15	0.29	-0.21	-0.15
**ST6GALNAC1**	0.12	-0.01	0.08	-0.04	0.02	-0.13	-0.12
**ST6GAL1**	0.47	0.53	0.46	0.49	0.47	-0.36	-0.23
**FUT9**	-0.13	0.04	-0.02	0.13	0.13	0.15	0.18
**MAN1A1**	-0.15	0.02	-0.04	0.11	0.11	0.17	0.19
**GCNT2**	-0.10	-0.10	0.05	0.04	0.22	0.10	0.14
**FUT7**	-0.50	-0.43	-0.39	-0.31	-0.26	0.43	0.35
**MAN2A1**	-0.05	-0.04	0.00	0.00	0.06	0.04	0.05
**GCNT1**	-0.19	-0.25	-0.13	-0.18	-0.07	0.14	0.11
