# Supplementary material for: Integrated N- and O-Glycomics of Acute Myeloid Leukemia (AML) Cell Lines
Source: Cells. 2021 Nov 6;10(11):3058. doi: 10.3390/cells10113058 (PMC8616353; doi:10.3390/cells10113058)

Supplementary Information 1

N-Glycomics – Structural Elucidation

Integrated *N*- and *O*-Glycomics of Acute Myeloid Leukemia (AML) Cell Lines

Constantin Blöchl [1, 2], Di Wang [1], Katarina Madunic [1], Guinevere S. M. Lageveen-Kammeijer [1], Christian G. Huber [2, 3, *], Manfred Wuhrer [1, *] and Tao Zhang [1, *]

[1] *Center for Proteomics and Metabolomics, Leiden University Medical Center, Albinusdreef 2, Leiden, the Netherlands; constantin.bloechl@sbg.ac.at, d.wang@lumc.nl, k.madunic@lumc.nl, g.s.m.kammeijer@lumc.nl, m.wuhrer@lumc.nl, t.zhang@lumc.nl*

[2] *Department of Biosciences, University of Salzburg, Hellbrunnerstrasse 34, Salzburg, Austria; constantin.bloechl@sbg.ac.at, c.huber@sbg.ac.at*

[3] *Cancer Cluster Salzburg, Department of Biosciences, University of Salzburg, Hellbrunnerstrasse 34, Salzburg, Austria; c.huber@sbg.ac.at*

* *Correspondence: c.huber@sbg.ac.at, m.wuhrer@lumc.nl, t.zhang@lumc.nl*

Glycan 1

H2N2F1

Depicted MS[2] was obtained from analysis of cell line: MOLM-13

**Monoisotopic mass:**	**896.35 Da**
**Charge observed:**	**1-**
**Theoretical ion:**	***m/z* 895.34**
**Observed ion:**	***m/z* 895.38**
**Mass deviation:**	***m/z* 0.04**
**Retention time:**	**49.0 min**

UniCarb-DB: #2223

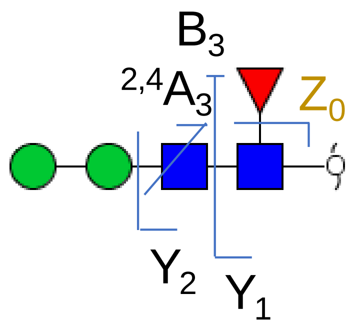

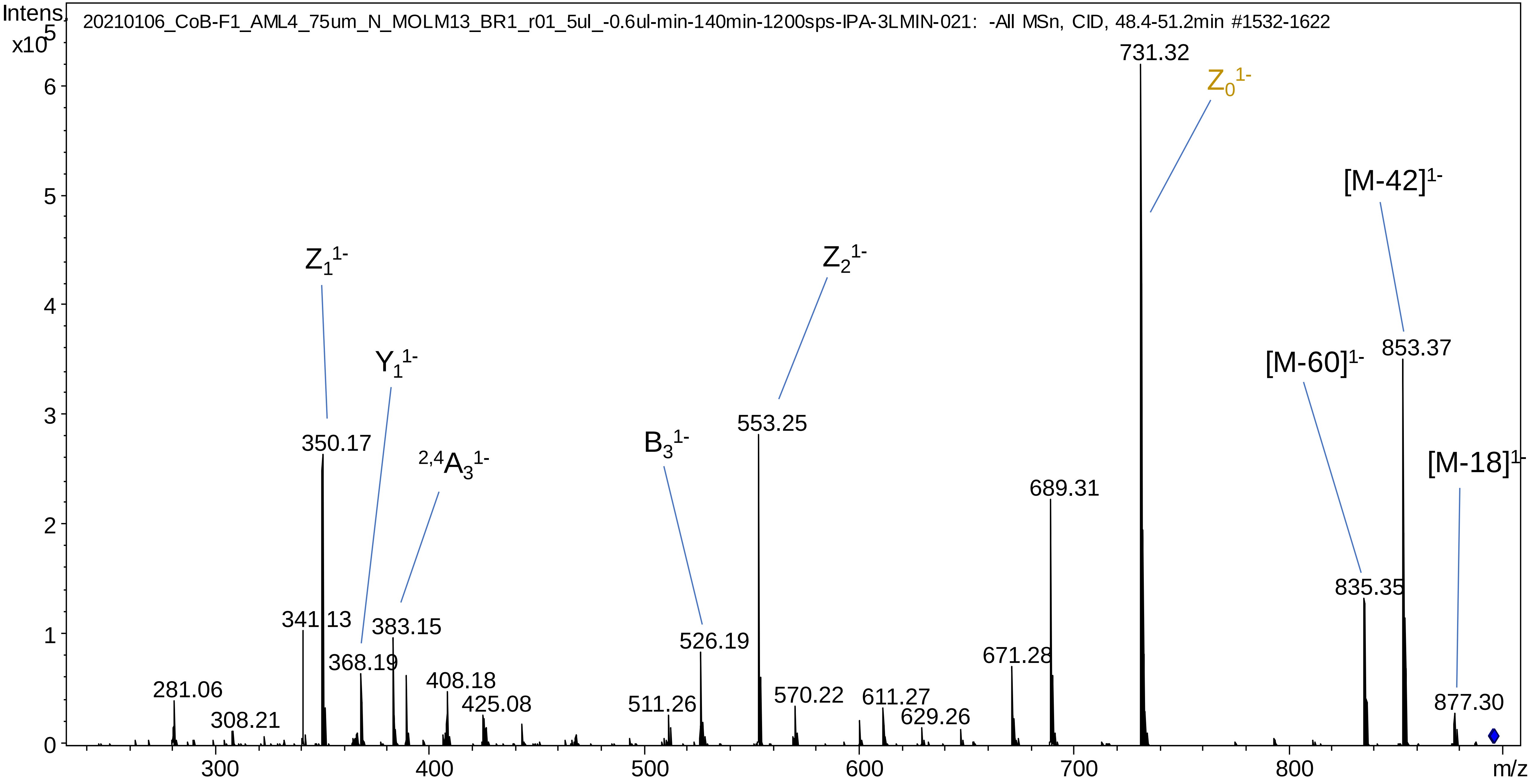

Glycan 2

H3N2

Depicted MS^2 was obtained from analysis of cell line: MOLM-13

**Monoisotopic mass:**	**912.35 Da**
**Charge observed:**	**1-**
**Theoretical ion:**	***m/z* 911.34**
**Observed ion:**	***m/z* 911.36**
**Mass deviation:**	***m/z* 0.02**
**Retention time:**	**47.1 min**

UniCarb-DB: #610

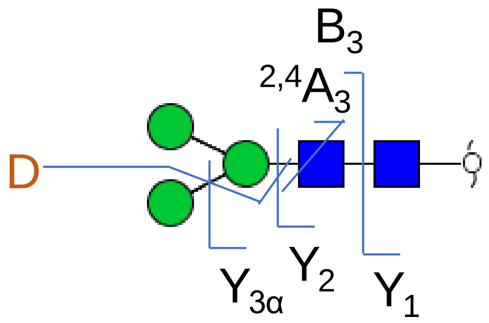

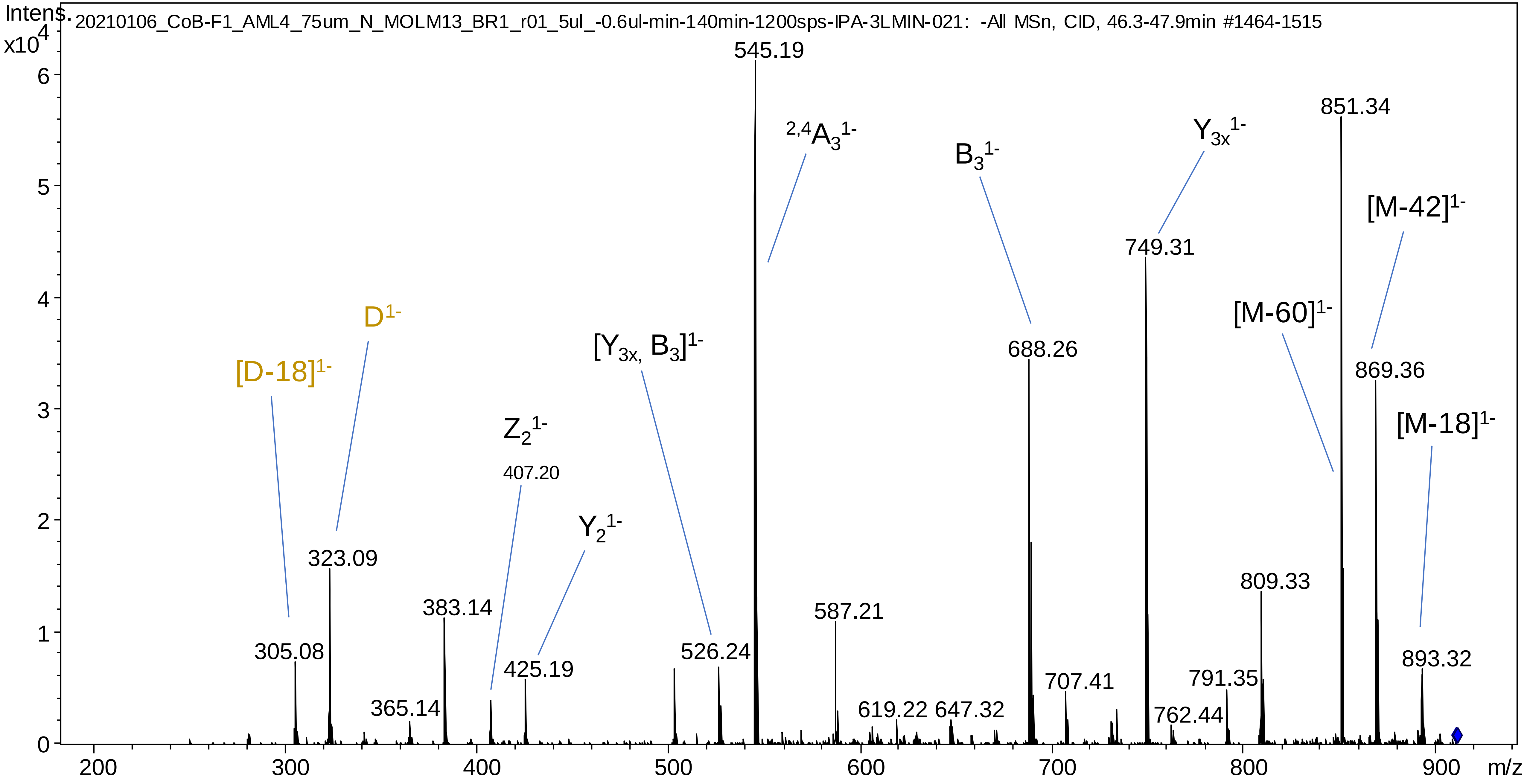

Glycan 3

H3N2F1

Depicted MS² was obtained from analysis of cell line: MOLM-13

**Monoisotopic mass:**	**1058.40 Da**
**Charge observed:**	**1-**
**Theoretical ion:**	***m/z* 1057.39**
**Observed ion:**	***m/z* 1057.40**
**Mass deviation:**	***m/z* 0.01**
**Retention time:**	**56.4 min**

UniCarb-DB: #441

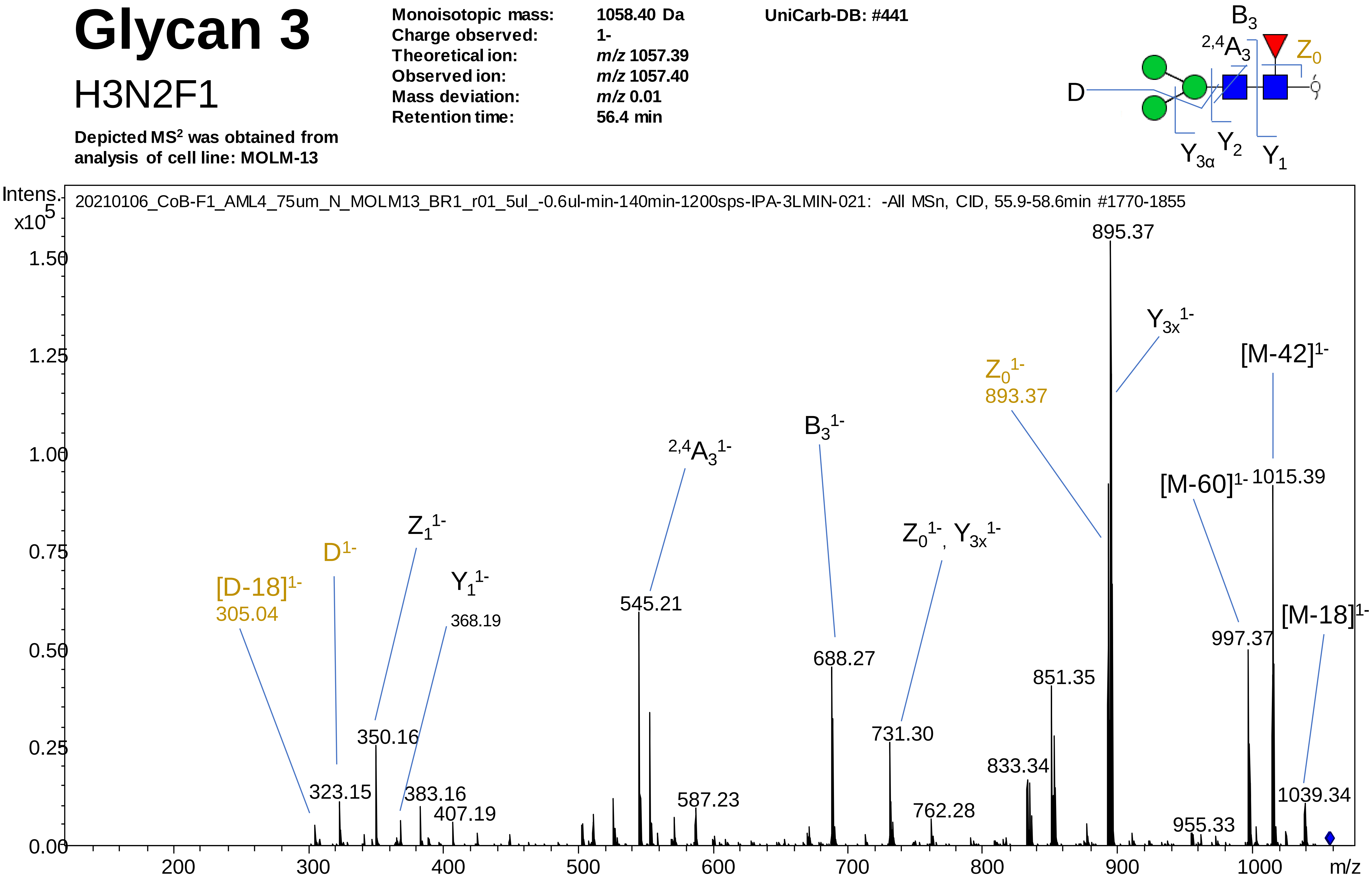

Glycan 4

H4N2

Depicted MS[2] was obtained from analysis of cell line: MOLM-13

**Monoisotopic mass:**	**1074.40 Da**
**Charge observed:**	**1-**
**Theoretical ion:**	***m/z* 1073.39**
**Observed ion:**	***m/z* 1073.39**
**Mass deviation:**	***m/z* 0.00**
**Retention time:**	**43.3 min**

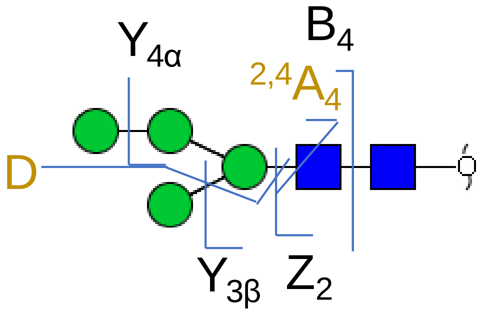

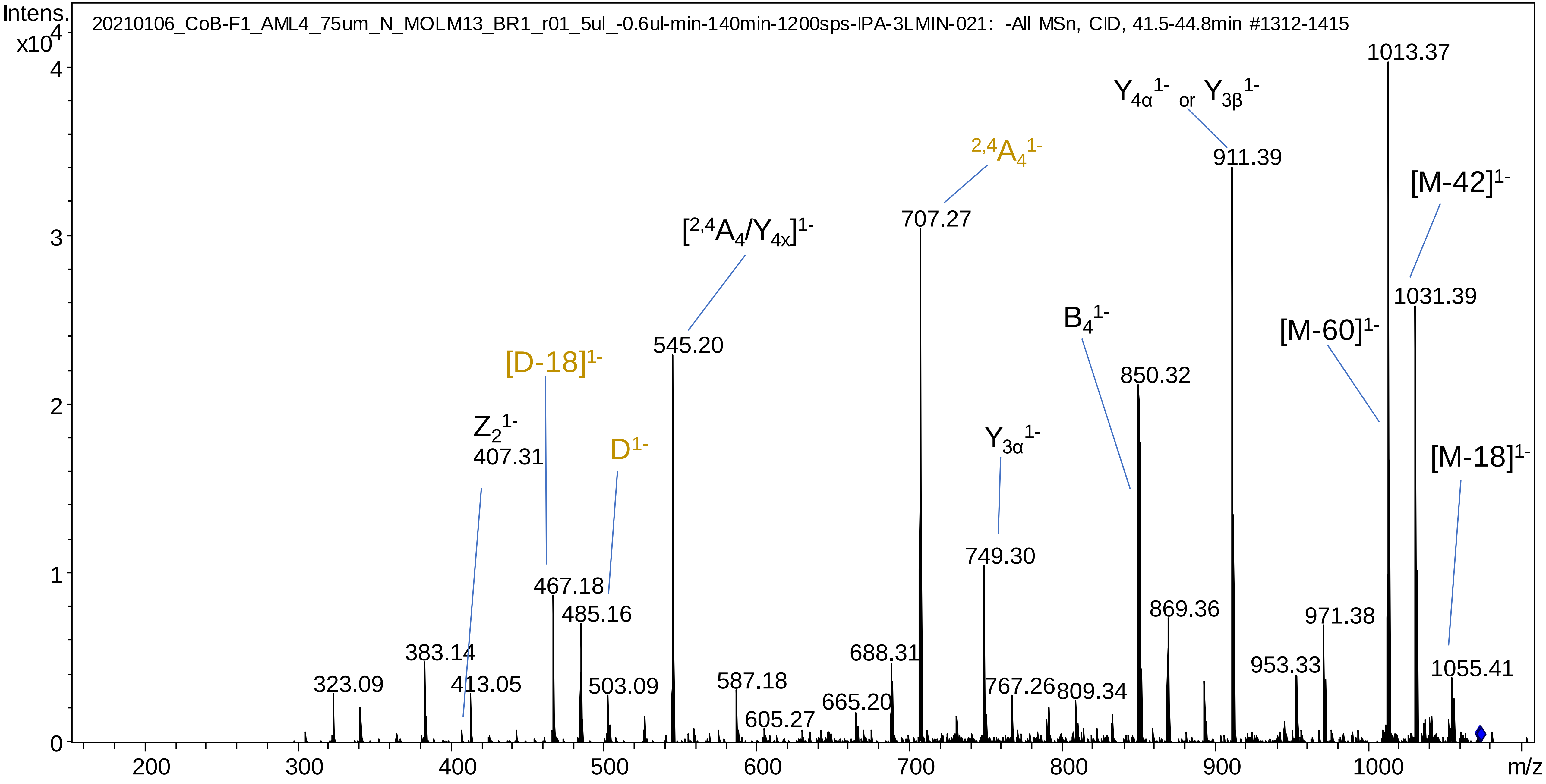

Glycan 5a

H5N2

Depicted MS^2 was obtained from analysis of cell line: Kasumi-1

**Monoisotopic mass:**	**1236.45 Da**
**Charge observed:**	**1-**
**Theoretical ion:**	***m/z* 1235.44**
**Observed ion:**	***m/z* 1235.47**
**Mass deviation:**	***m/z* 0.03**
**Retention time:**	**34.7 min**

UniCarb-DB: #2213

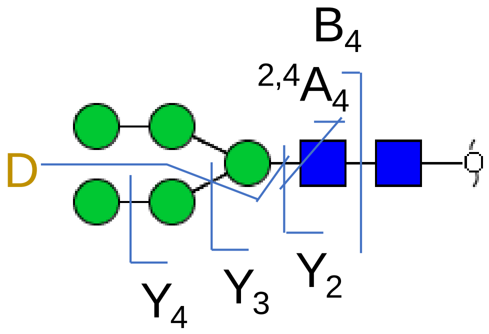

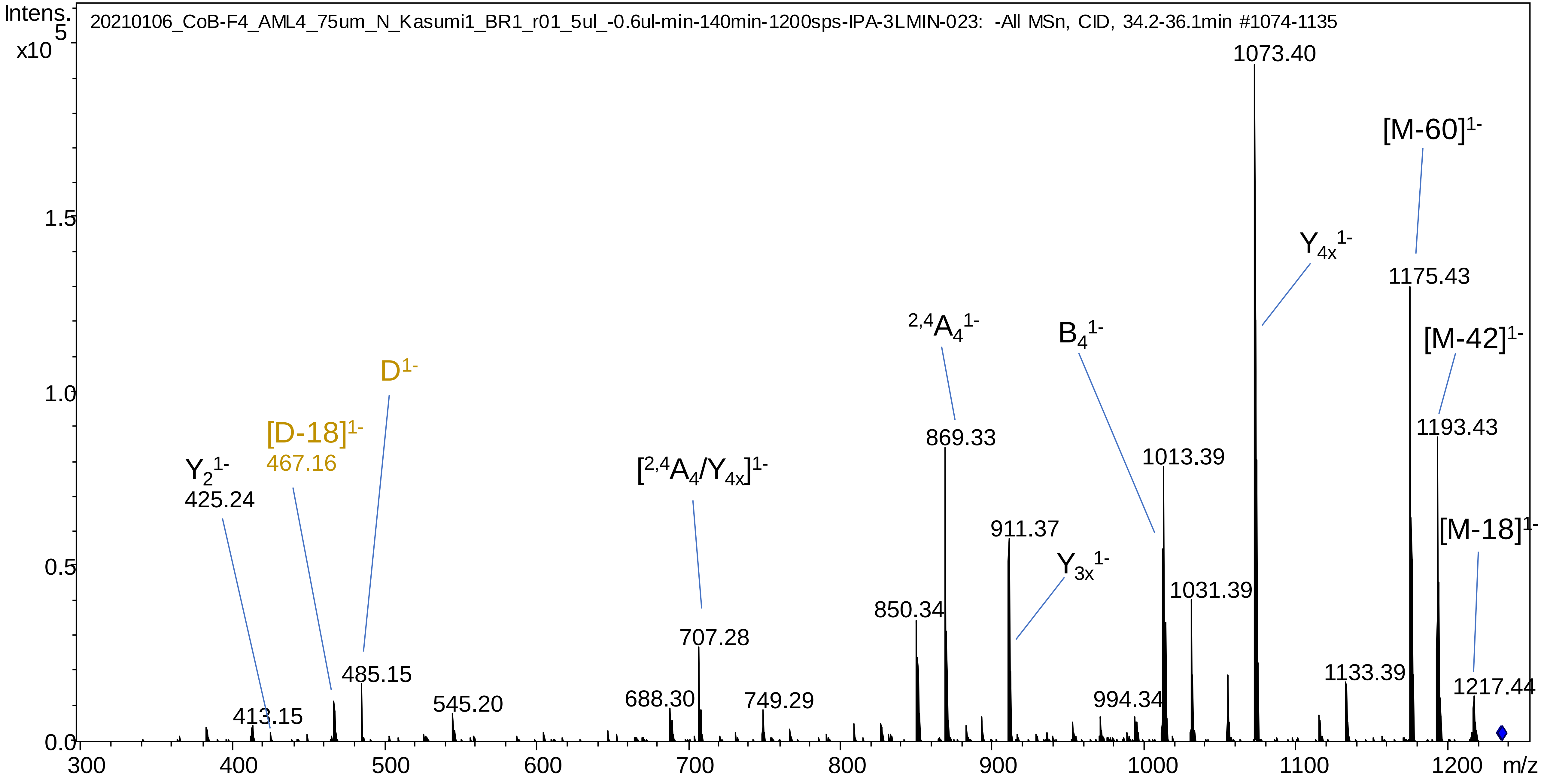

Glycan 5b

H5N2

Depicted MS[2] was obtained from analysis of cell line: Kasumi-1

**Monoisotopic mass:**	**1236.45 Da**
**Charge observed:**	**1-**
**Theoretical ion:**	***m/z* 1235.44**
**Observed ion:**	***m/z* 1235.46**
**Mass deviation:**	***m/z* 0.02**
**Retention time:**	**48.4 min**

UniCarb-DB: #395

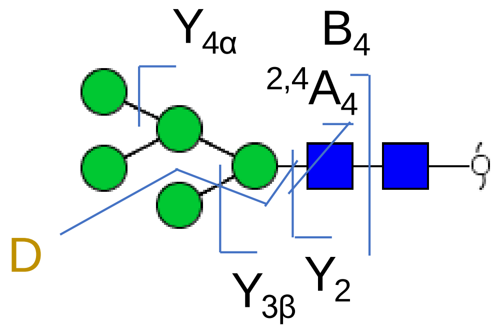

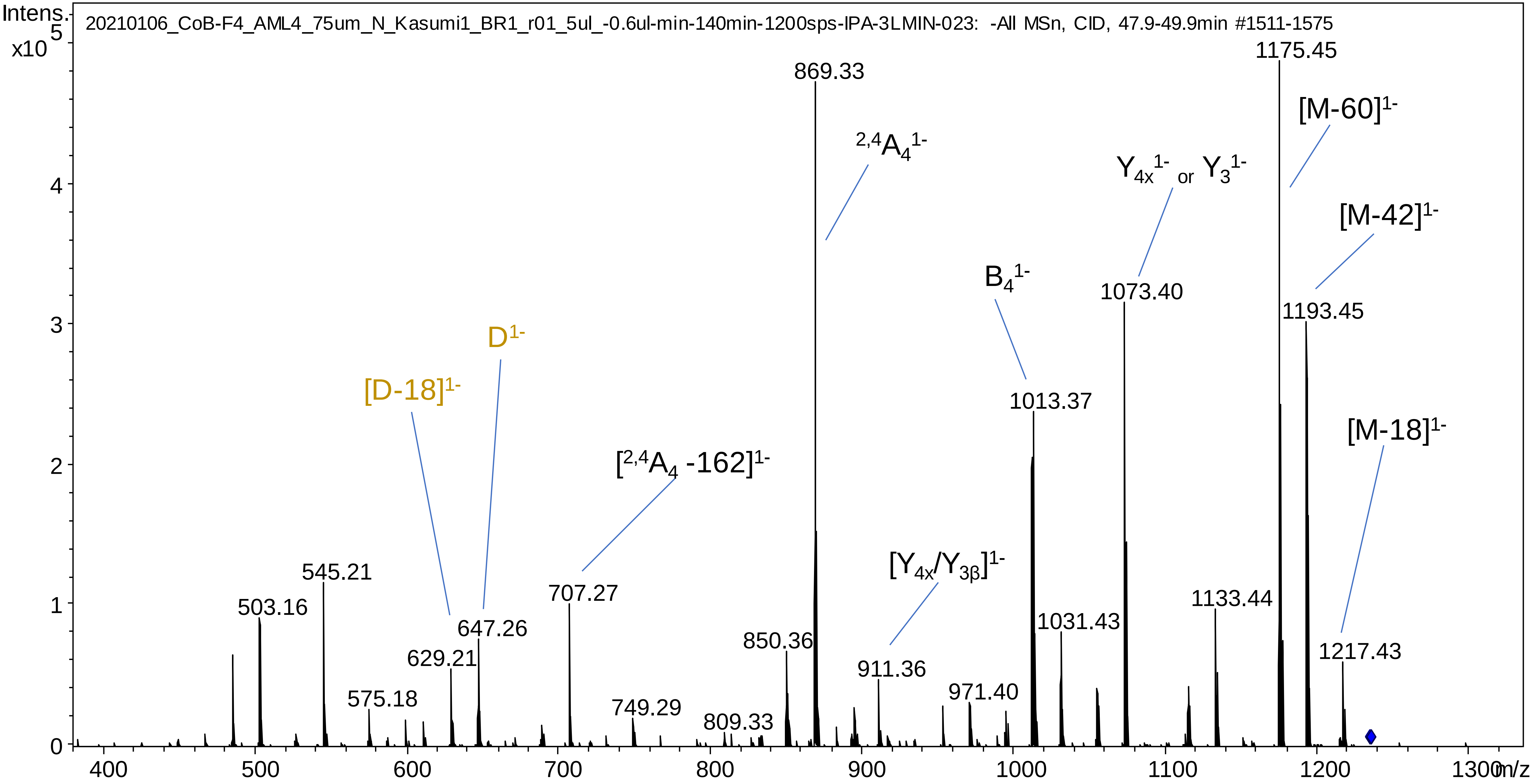

Glycan 6

H6N2

Depicted MS² was obtained from analysis of cell line: MOLM-13

**Monoisotopic mass:**	**1398.51 Da**
**Charge observed:**	**1-**
**Theoretical ion:**	***m/z* 1397.50**
**Observed ion:**	***m/z* 1397.54**
**Mass deviation:**	***m/z* 0.04**
**Retention time:**	**39.9 min**

UniCarb-DB: #613

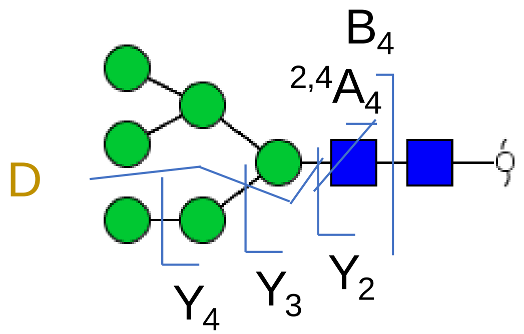

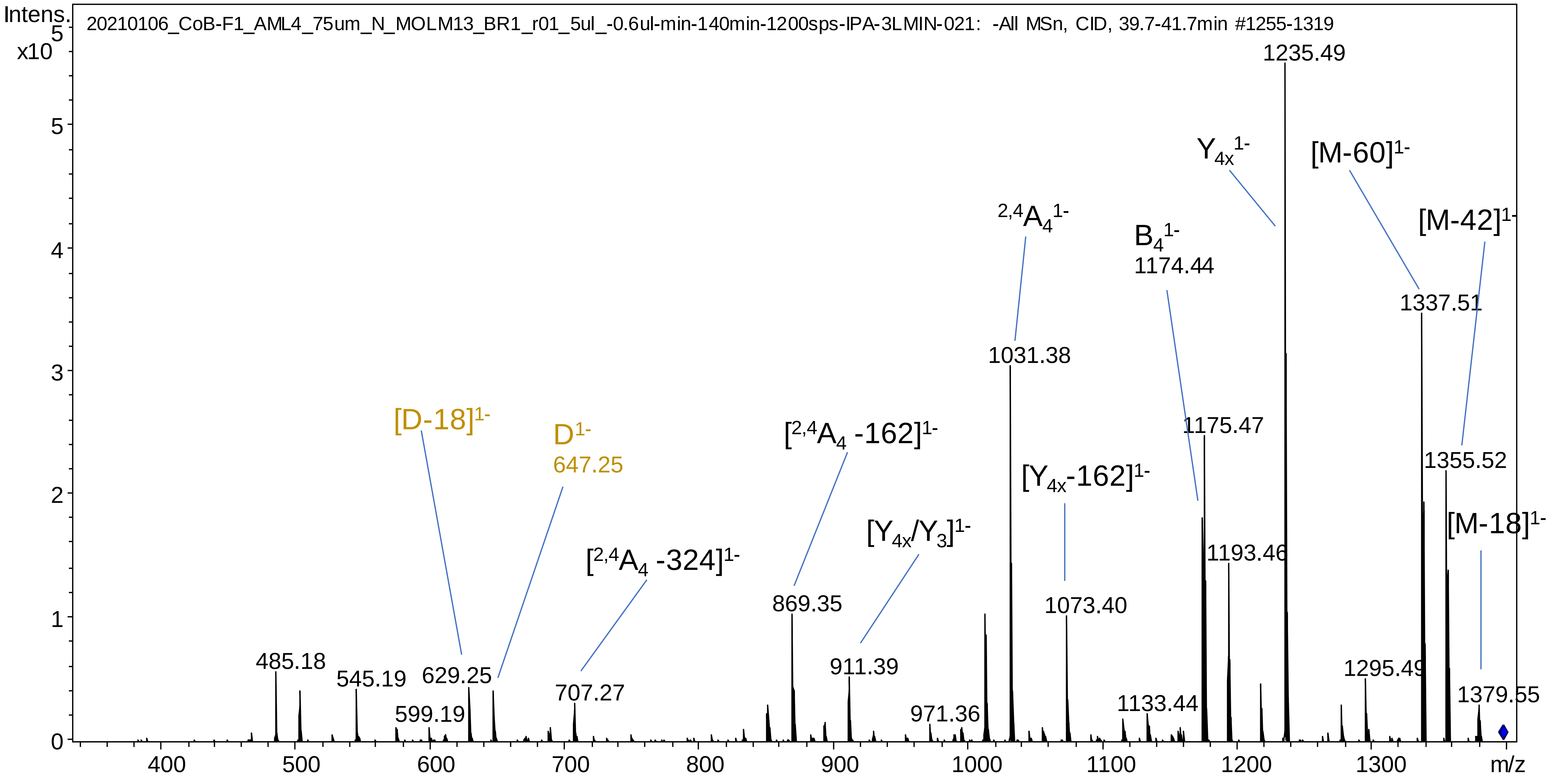

Glycan 7

H7N2

Depicted MS² was obtained from analysis of cell line: MOLM-13

**Monoisotopic mass:**	**1560.55 Da**
**Charge observed:**	**2-**
**Theoretical ion:**	***m/z* 779.27**
**Observed ion:**	***m/z* 779.31**
**Mass deviation:**	***m/z* 0.04**
**Retention time:**	**38.4 min**

UniCarb-DB: #403

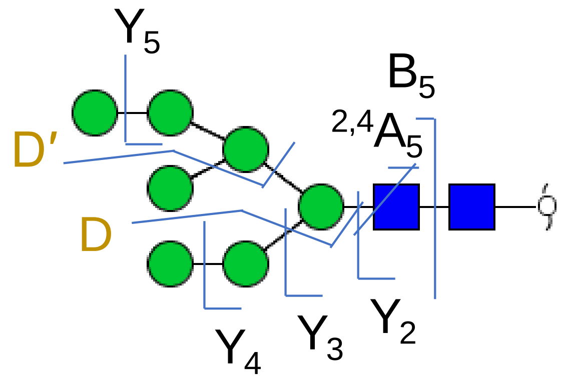

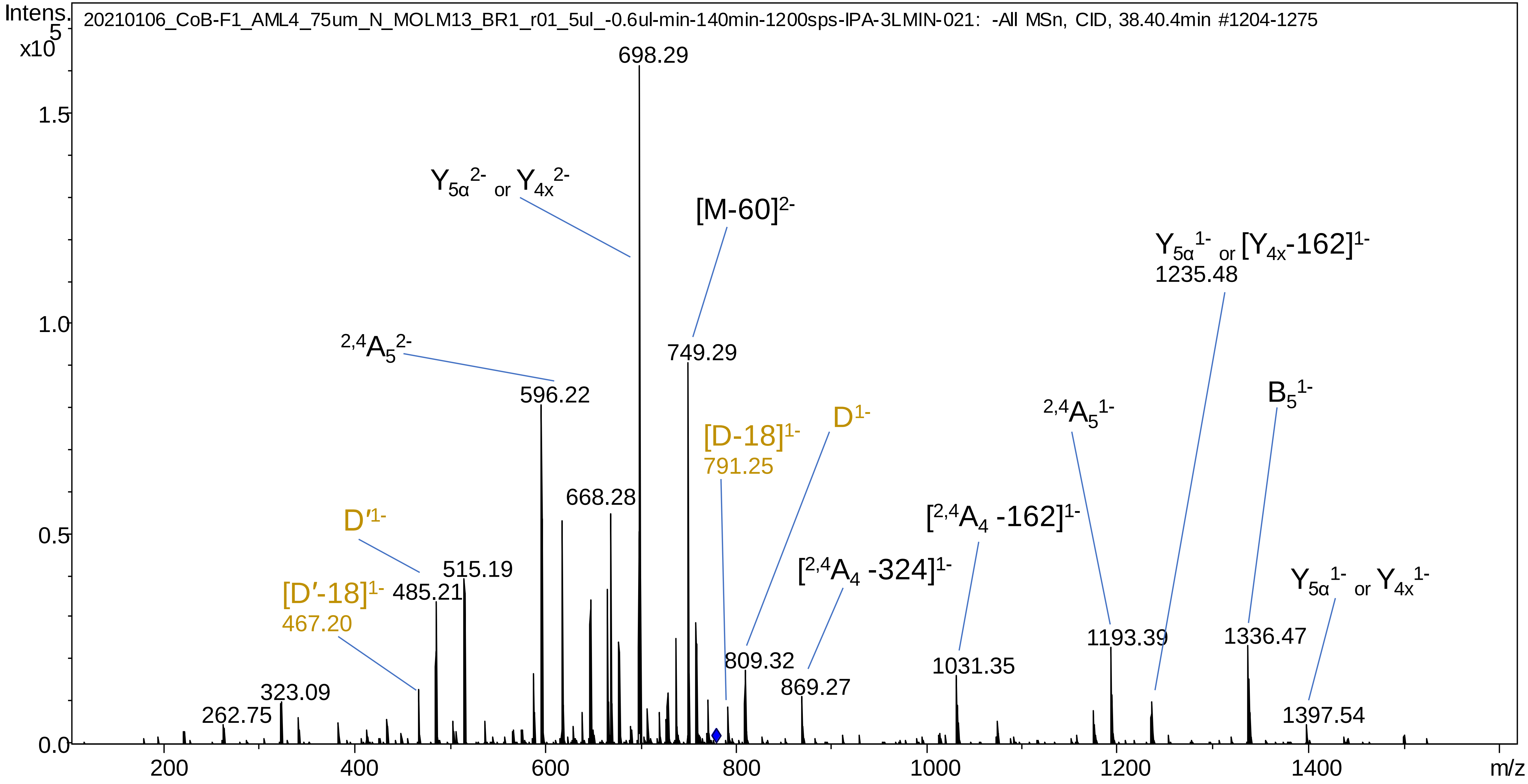

Glycan 8

H8N2

Depicted MS² was obtained from analysis of cell line: MOLM-13

**Monoisotopic mass:**	**1722.61 Da**
**Charge observed:**	**2-**
**Theoretical ion:**	***m/z* 860.30**
**Observed ion:**	***m/z* 860.34**
**Mass deviation:**	***m/z* 0.04**
**Retention time:**	**38.4 min**

UniCarb-DB: #2296

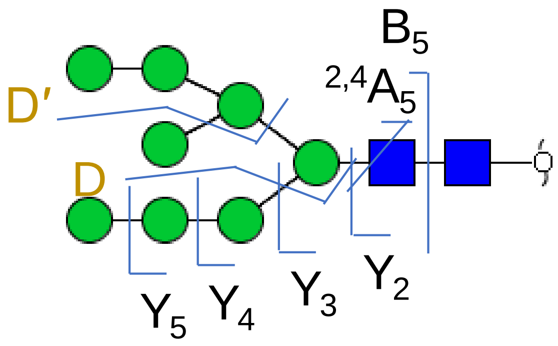

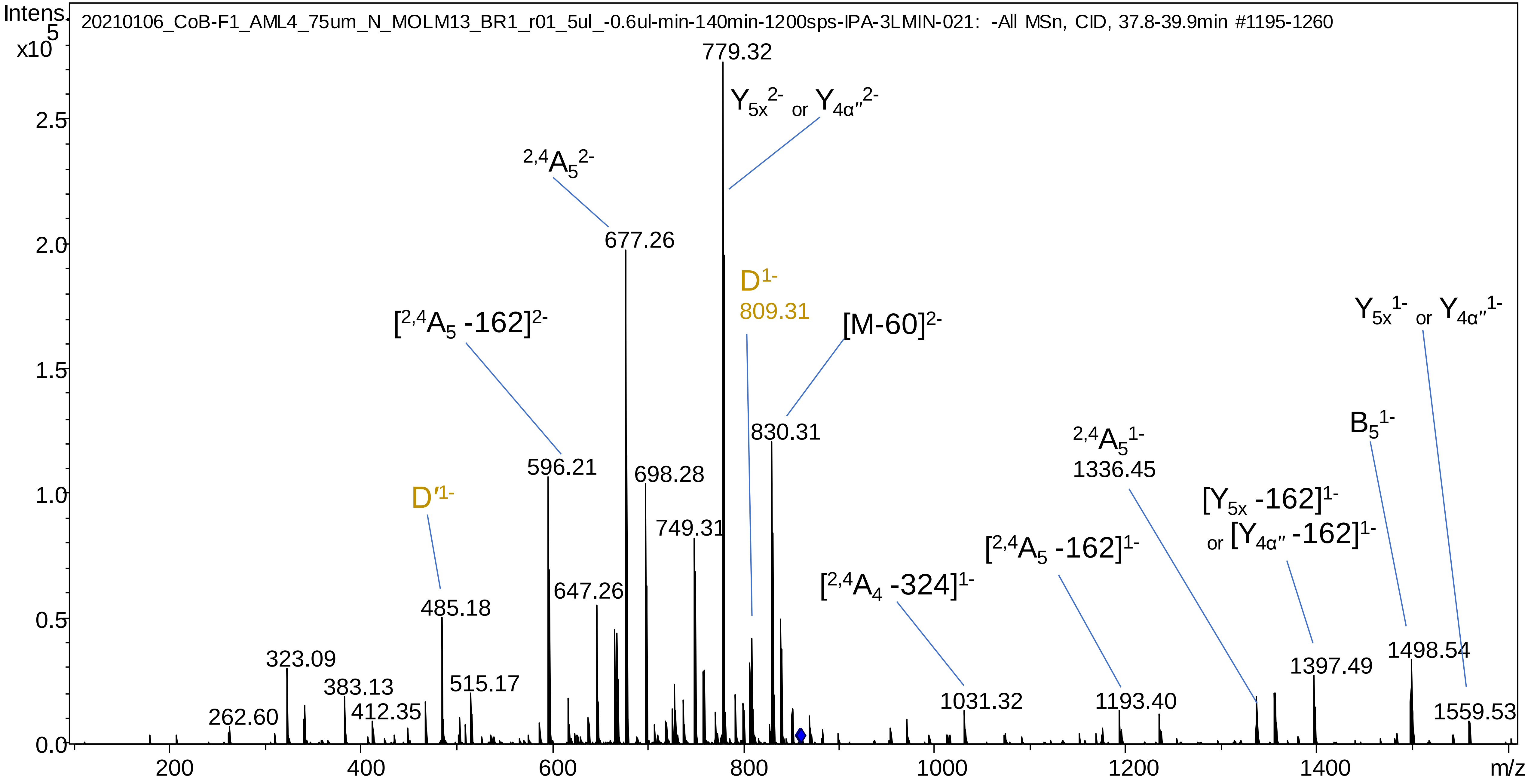

Glycan 9

H9N2

Depicted MS² was obtained from analysis of cell line: MOLM-13

**Monoisotopic mass:**	**1884.66 Da**
**Charge observed:**	**2-**
**Theoretical ion:**	***m/z* 941.32**
**Observed ion:**	***m/z* 941.34**
**Mass deviation:**	***m/z* 0.02**
**Retention time:**	**38.8 min**

UniCarb-DB: #429

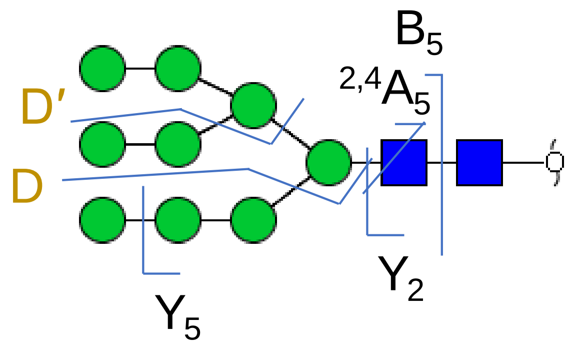

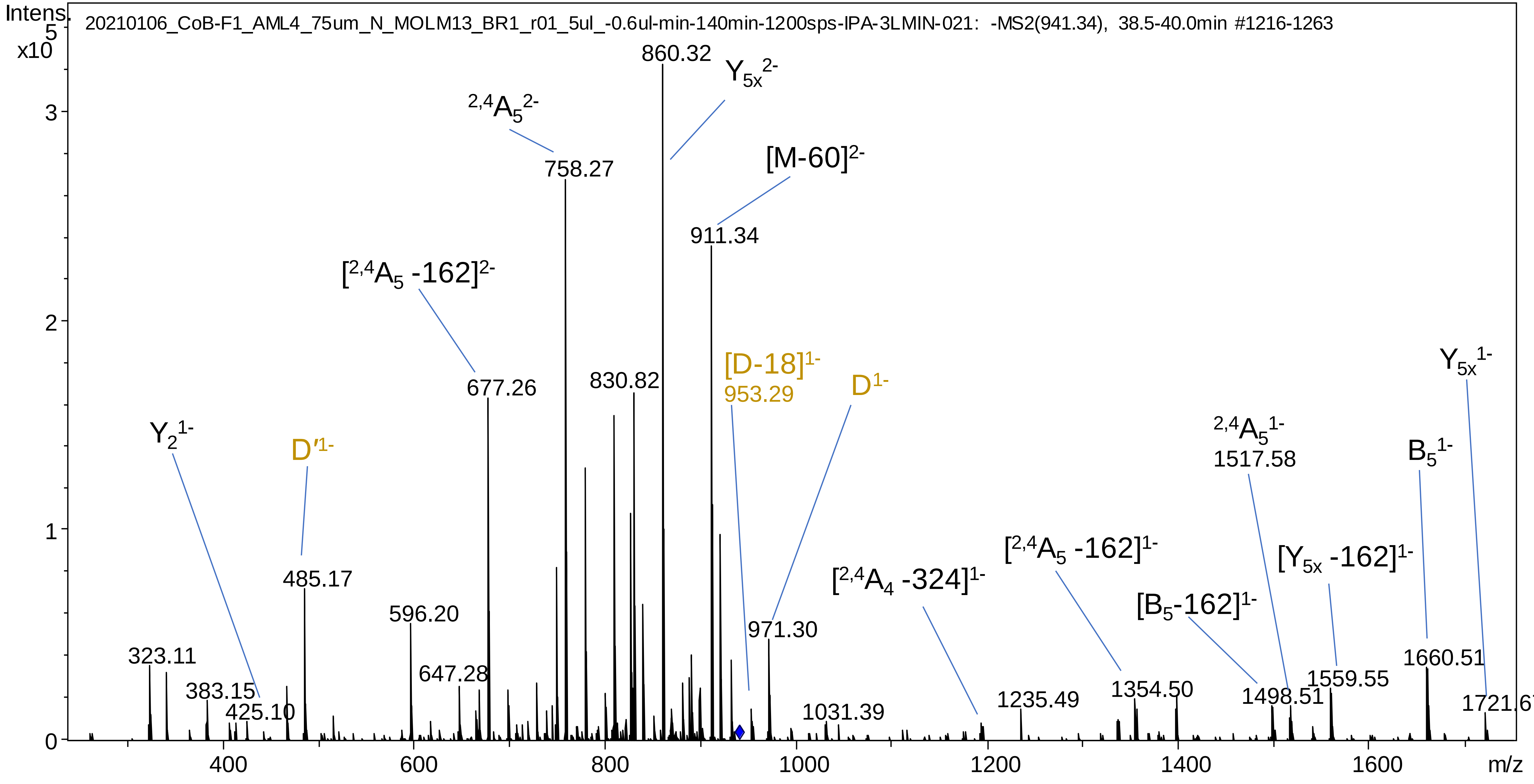

Glycan 10a

H5N2P1

Depicted MS^2 was obtained from analysis of cell line: Kasumi-1

**Monoisotopic mass:**	**1316.42 Da**
**Charge observed:**	**1-**
**Theoretical ion:**	***m/z* 1315.41**
**Observed ion:**	***m/z* 1315.42**
**Mass deviation:**	***m/z* 0.01**
**Retention time:**	**27.2 min**

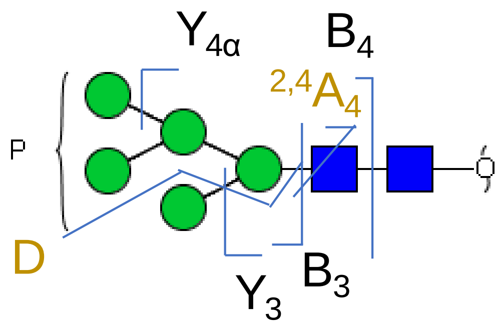

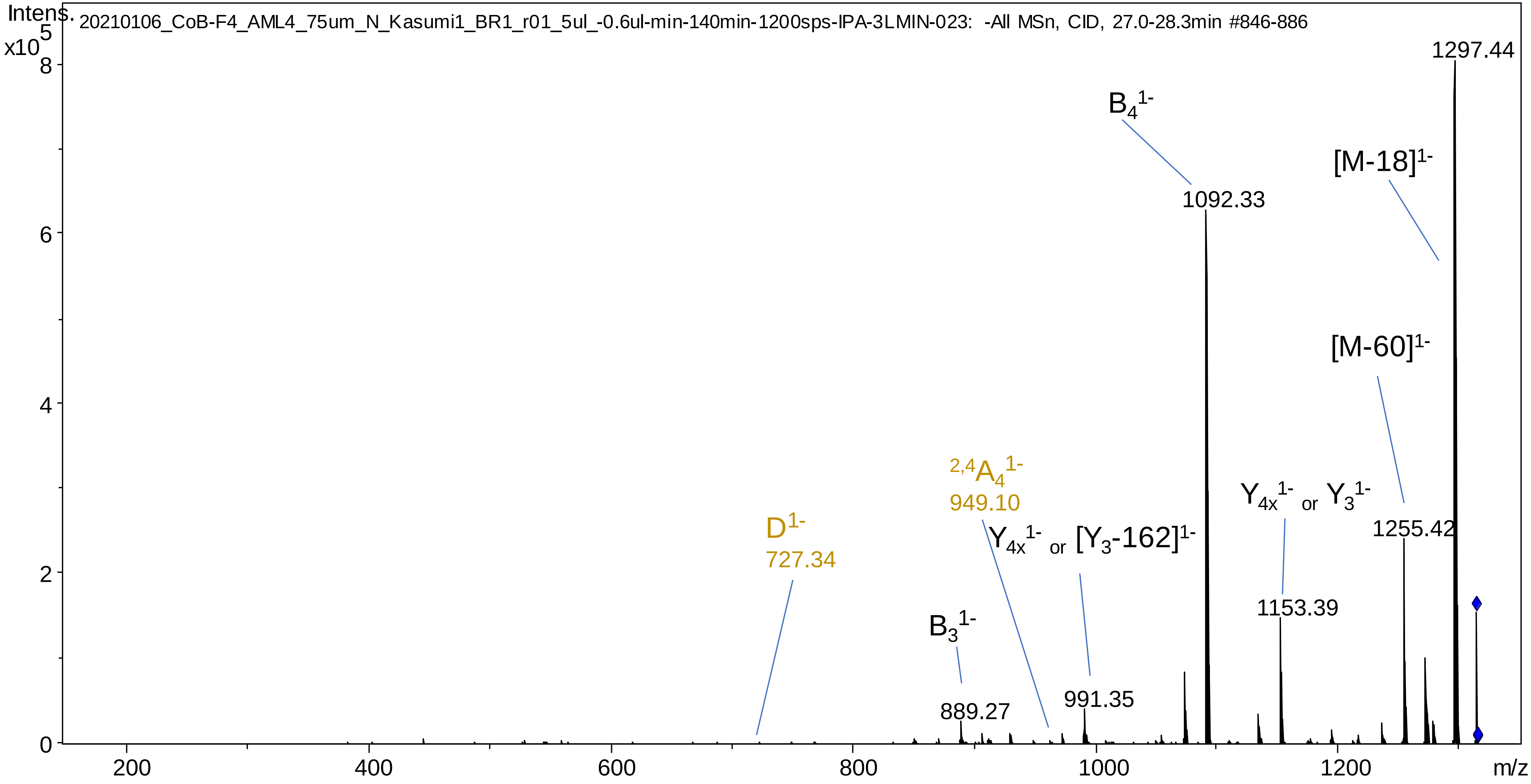

Glycan 10b

H5N2P1

Depicted MS² was obtained from analysis of cell line: Kasumi-1

**Monoisotopic mass:**	**1316.42 Da**
**Charge observed:**	**2-**
**Theoretical ion:**	***m/z* 1315.41**
**Observed ion:**	***m/z* 1315.44**
**Mass deviation:**	***m/z* 0.03**
**Retention time:**	**31.1 min**

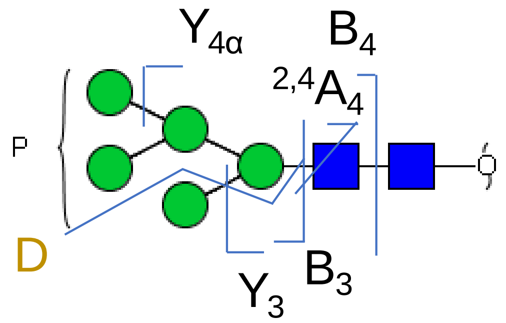

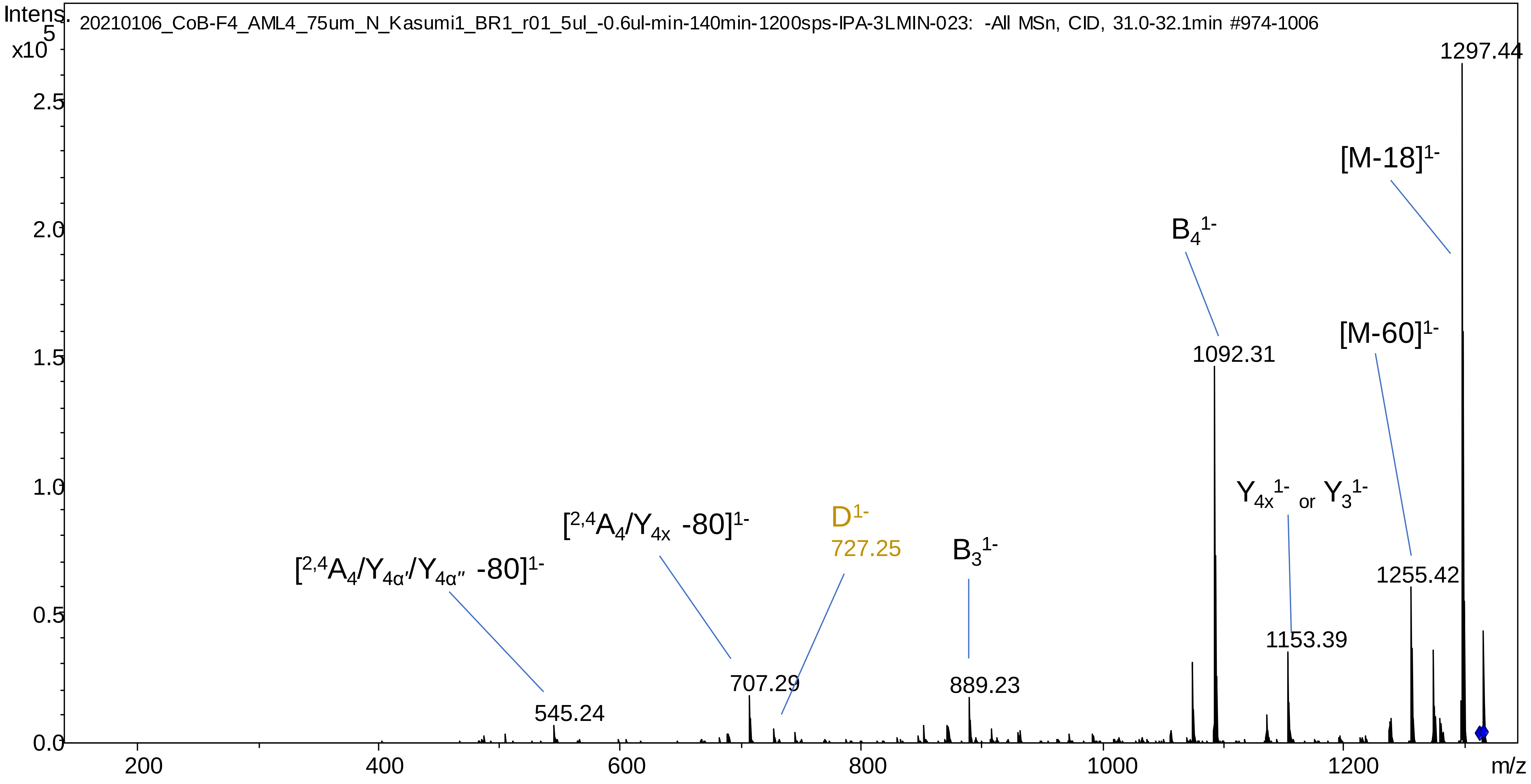

Glycan 11a

H6N2P1

Depicted MS² was obtained from analysis of cell line: OCI-AML-2

**Monoisotopic mass:**	**1478.47 Da**
**Charge observed:**	**2-**
**Theoretical ion:**	***m/z* 738.23**
**Observed ion:**	***m/z* 738.25**
**Mass deviation:**	***m/z* 0.02**
**Retention time:**	**32.6 min**

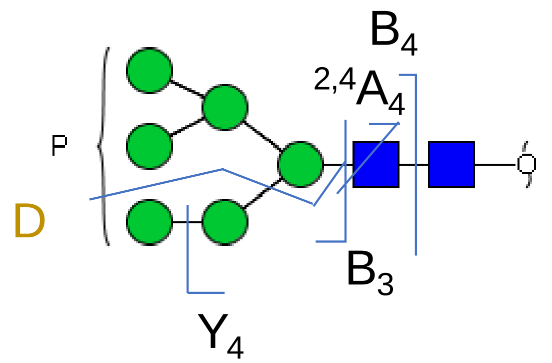

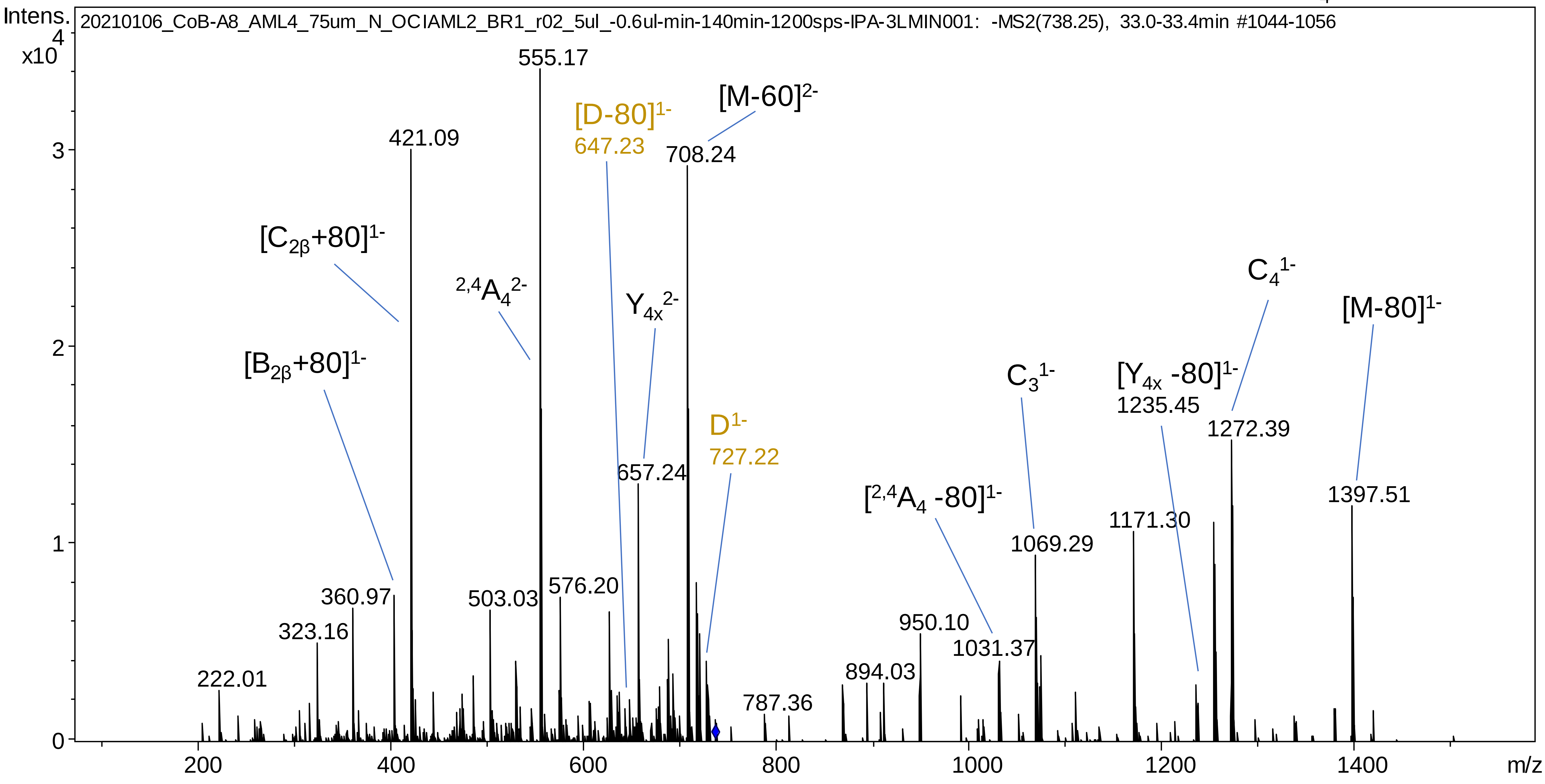

Glycan 11b

H6N2P1

Depicted MS² was obtained from analysis of cell line: MOLM-13

**Monoisotopic mass:**	**1478.47 Da**
**Charge observed:**	**2-**
**Theoretical ion:**	***m/z* 738.23**
**Observed ion:**	***m/z* 738.25**
**Mass deviation:**	***m/z* 0.02**
**Retention time:**	**35.6 min**

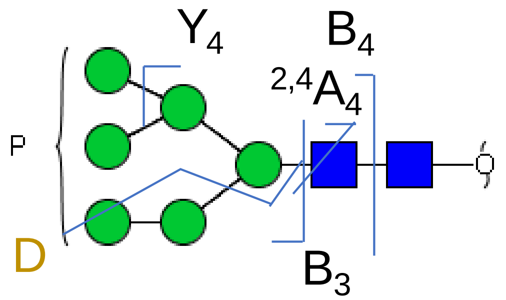

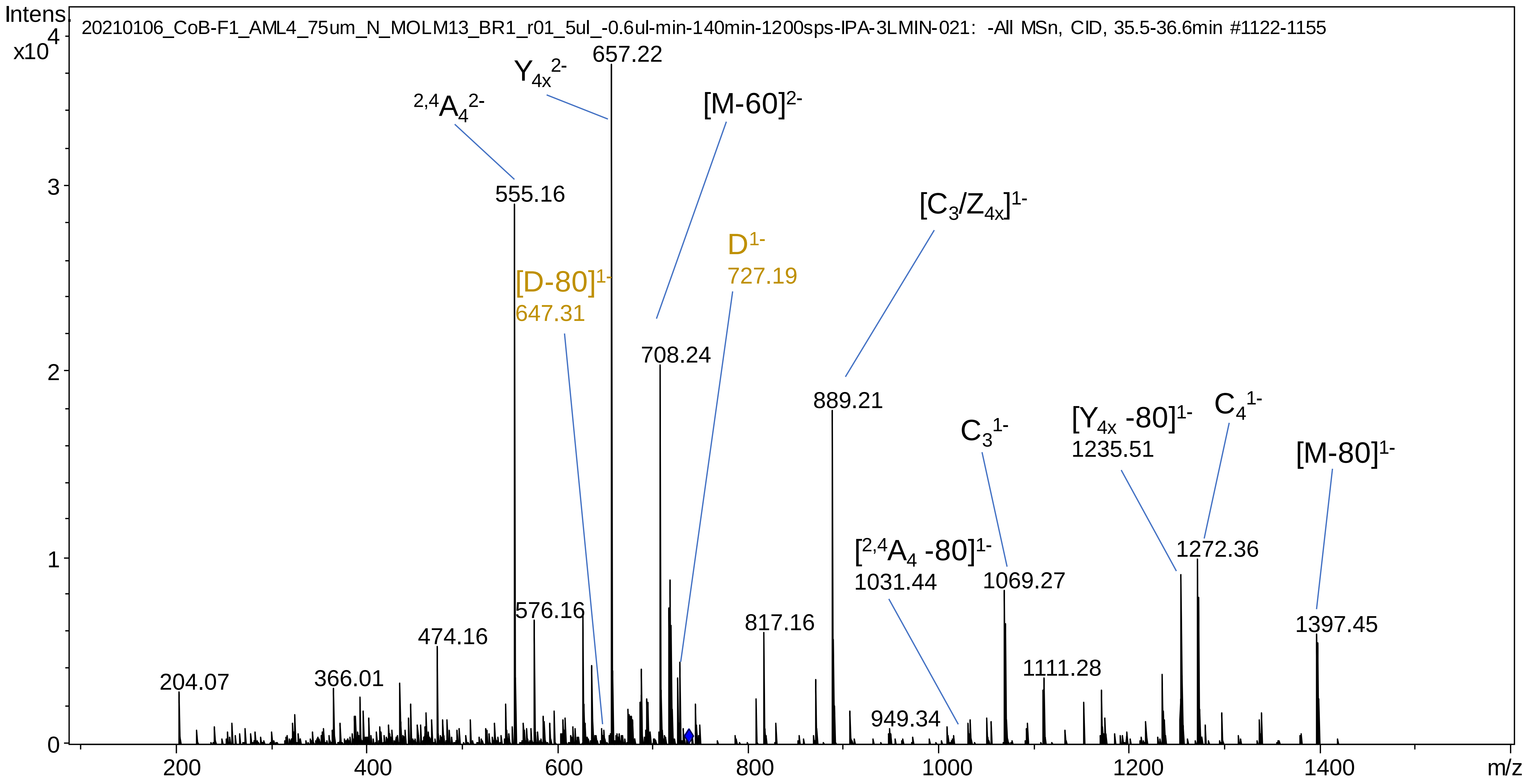

Glycan 12

H7N2P1

Depicted MS² was obtained from analysis of cell line: MOLM-13

**Monoisotopic mass:**	**1640.52 Da**
**Charge observed:**	**2-**
**Theoretical ion:**	***m/z* 819.25**
**Observed ion:**	***m/z* 819.28**
**Mass deviation:**	***m/z* 0.03**
**Retention time:**	**32.4 min**

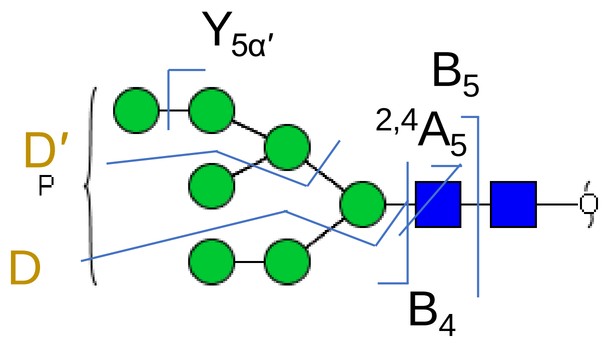

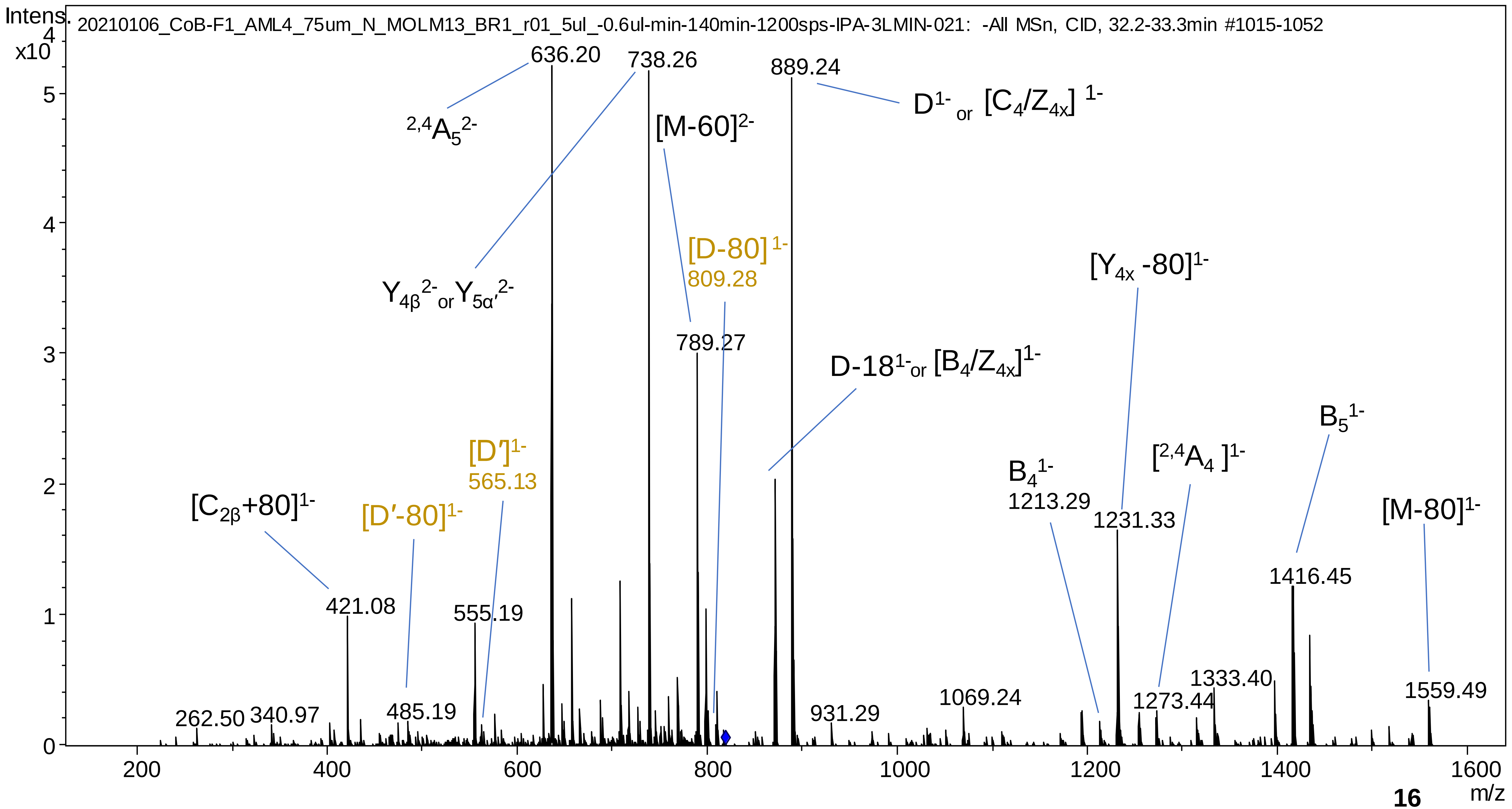

Glycan 13

H7N2P2

Depicted MS^2 was obtained from analysis of cell line: MOLM-13

**Monoisotopic mass:**	**1720.49 Da**
**Charge observed:**	**2-**
**Theoretical ion:**	***m/z* 859.24**
**Observed ion:**	***m/z* 859.26**
**Mass deviation:**	***m/z* 0.02**
**Retention time:**	**26.2 min**

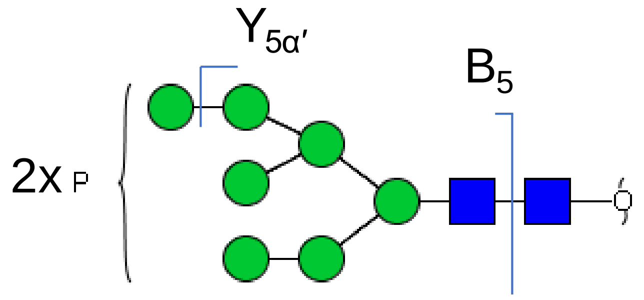

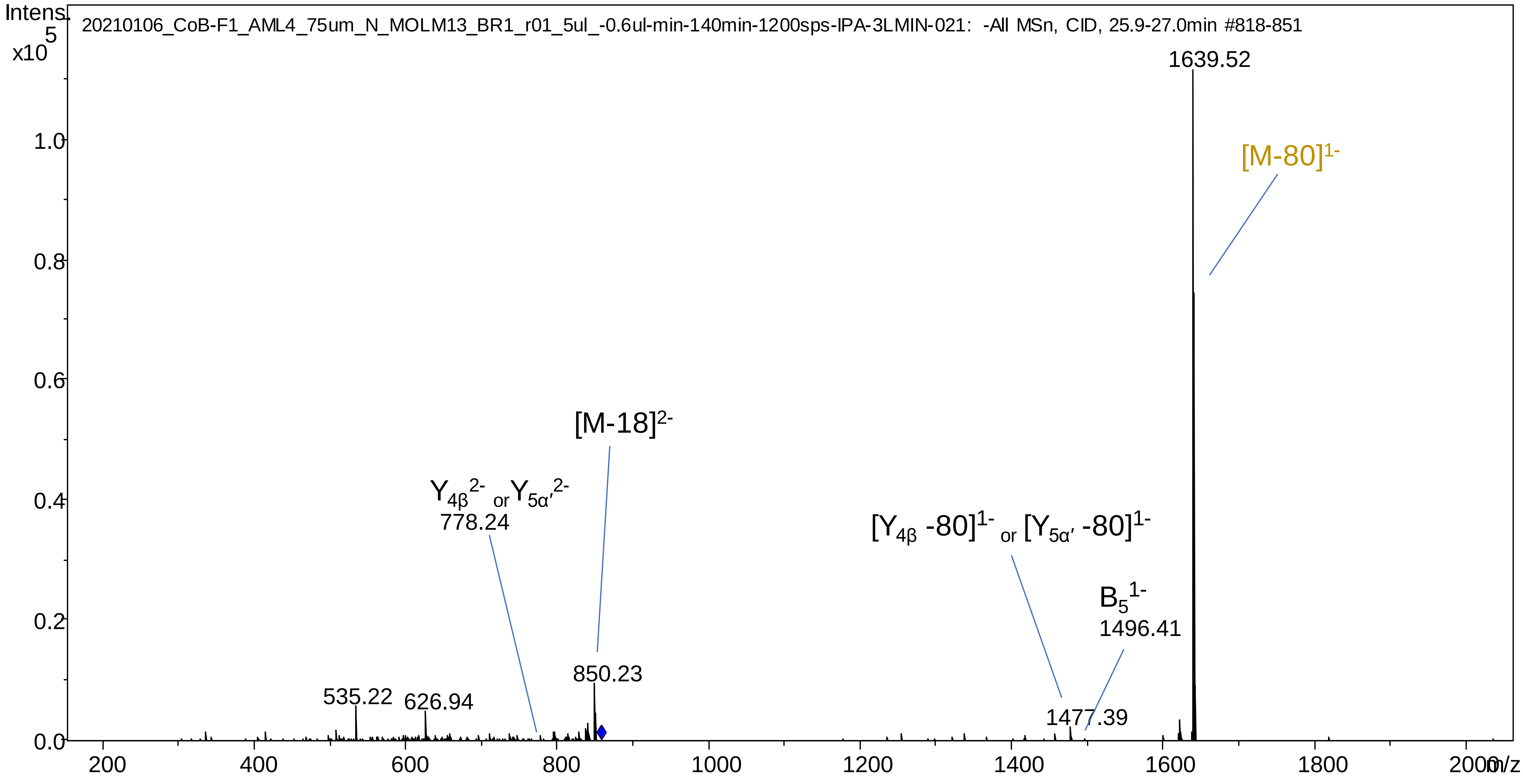

Glycan 14

H3N4F1

Depicted MS^2 was obtained from analysis of cell line: MV4-11

**Monoisotopic mass:**	**1464.56 Da**
**Charge observed:**	**2-**
**Theoretical ion:**	***m/z* 731.28**
**Observed ion:**	***m/z* 731.32**
**Mass deviation:**	***m/z* 0.04**
**Retention time:**	**48.4 min**

UniCarb-DB: #399

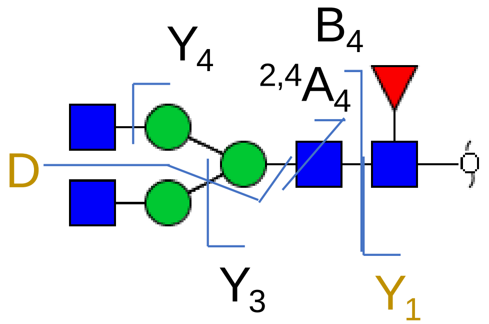

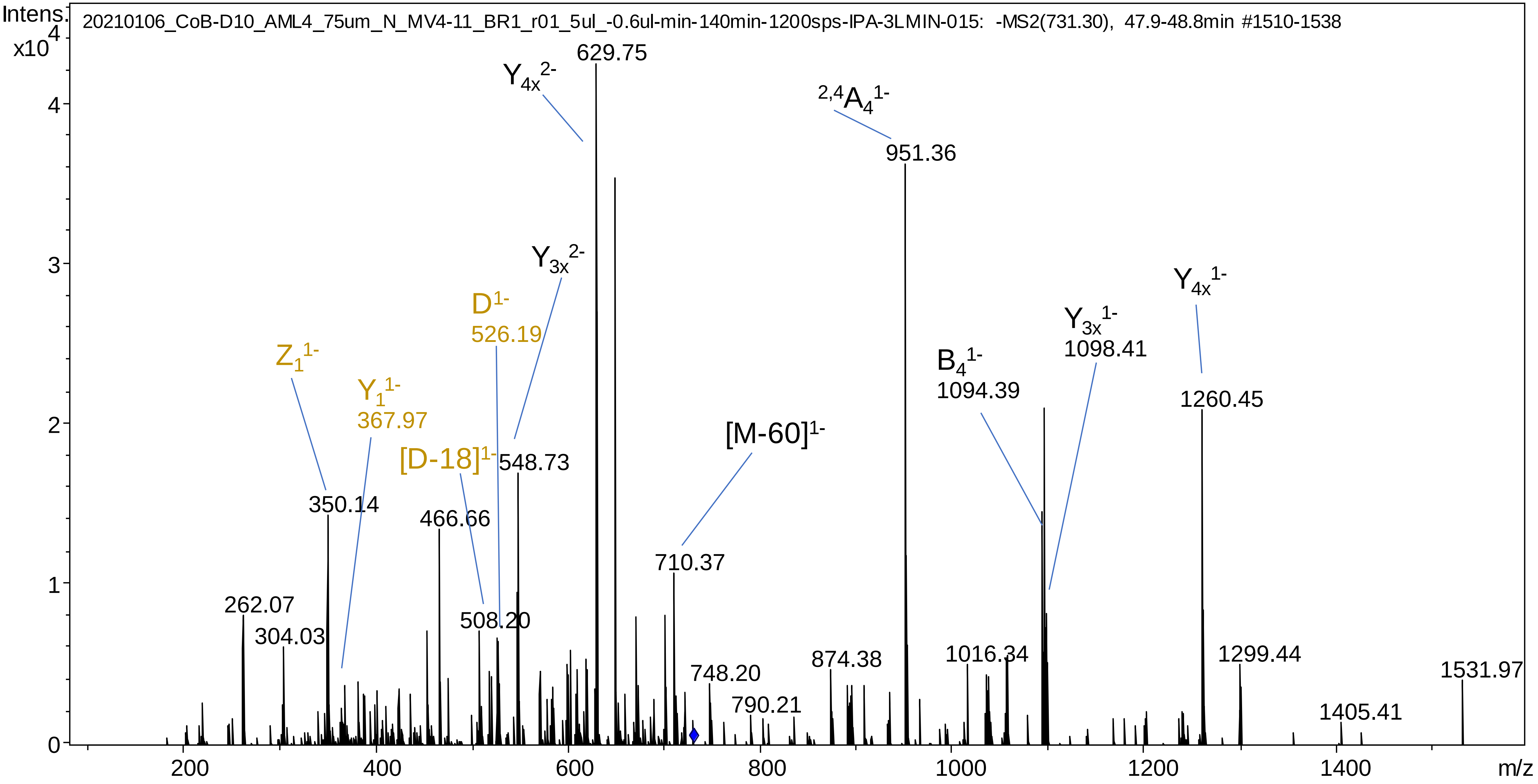

Glycan 14

H3N4F1

Depicted MS^2 was obtained from analysis of cell line: MV4-11

**Monoisotopic mass:**	**1464.56 Da**
**Charge observed:**	**1-**
**Theoretical ion:**	***m/z* 1463.55**
**Observed ion:**	***m/z* 1463.60**
**Mass deviation:**	***m/z* 0.05**
**Retention time:**	**48.4 min**

UniCarb-DB: #399

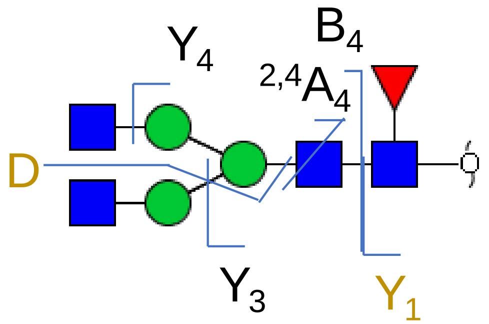

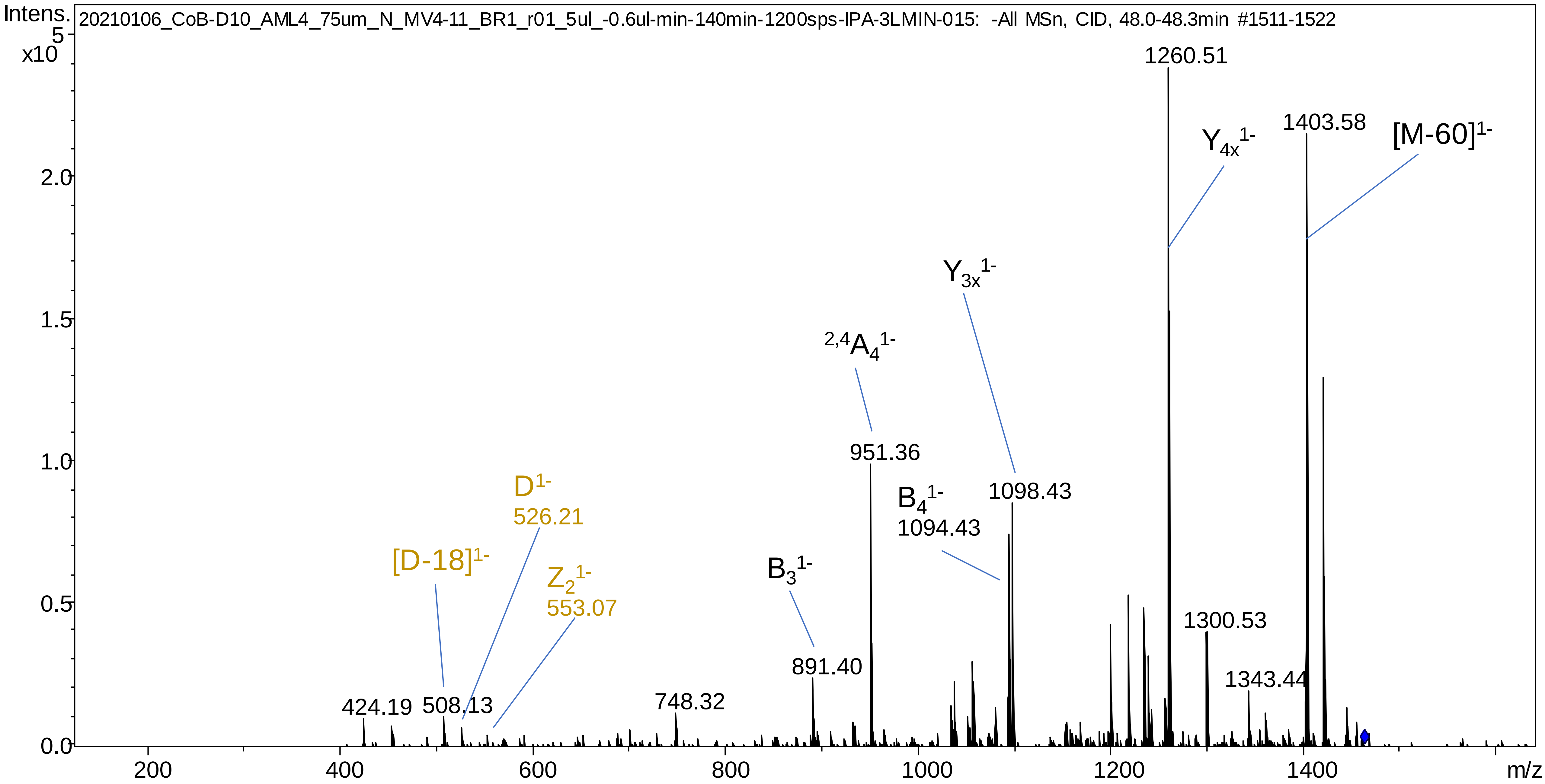

Glycan 15

H4N4F1

Depicted MS^2 was obtained from analysis of cell line: KG-1

**Monoisotopic mass:**	**1626.61 Da**
**Charge observed:**	**2-**
**Theoretical ion:**	***m/z* 812.30**
**Observed ion:**	***m/z* 812.32**
**Mass deviation:**	***m/z* 0.02**
**Retention time:**	**50.3 min**

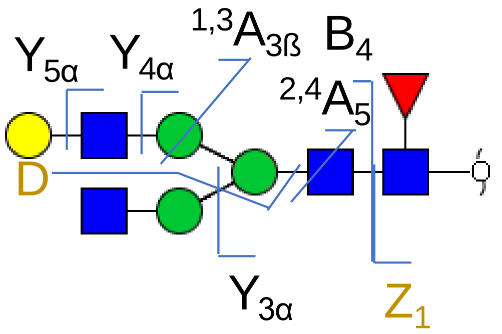

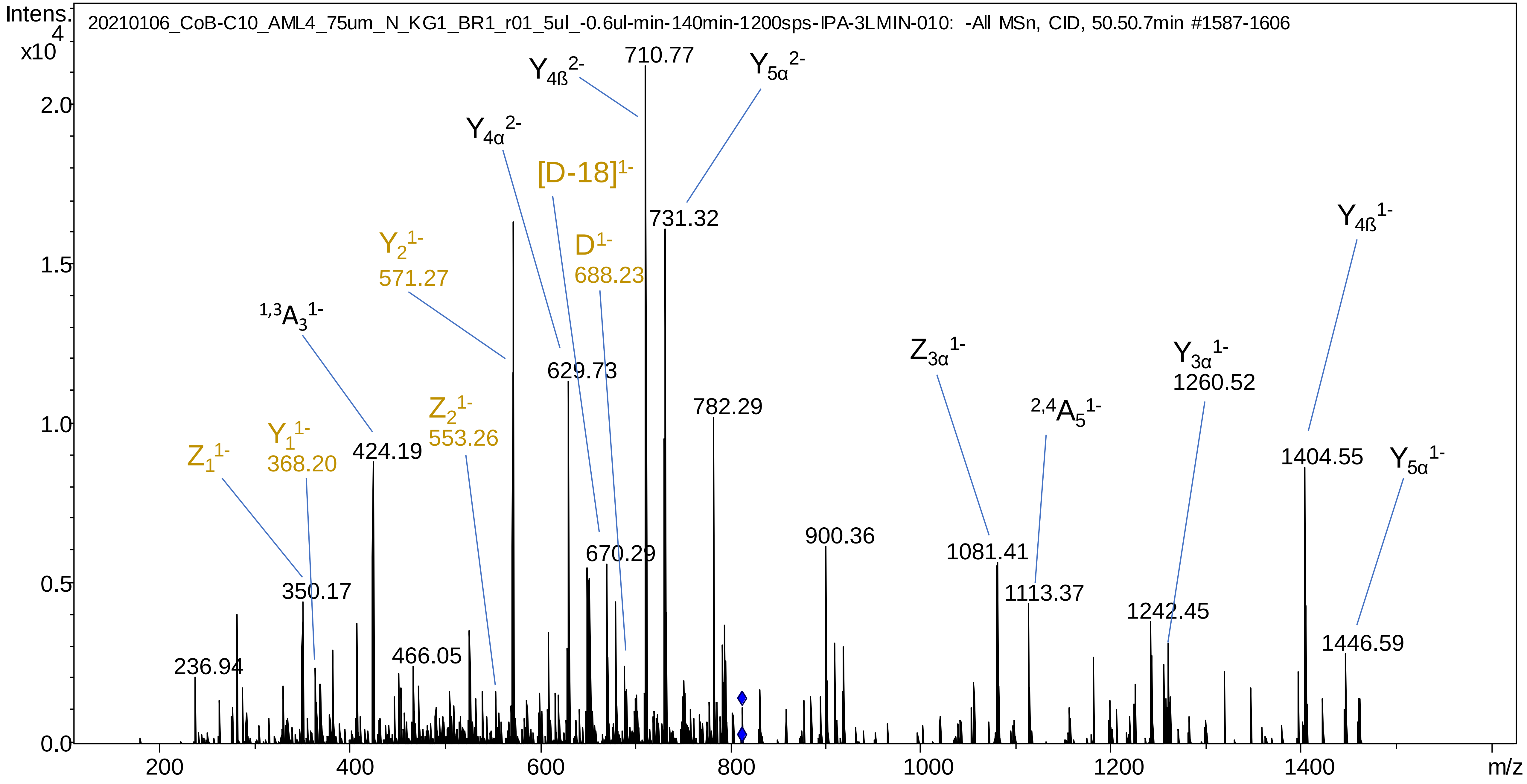

Glycan 16

H5N4F1

Depicted MS² was obtained from analysis of cell line: OCI-AML-3

**Monoisotopic mass:**	**1788.67 Da**
**Charge observed:**	**2-**
**Theoretical ion:**	***m/z* 893.33**
**Observed ion:**	***m/z* 893.36**
**Mass deviation:**	***m/z* 0.03**
**Retention time:**	**53.6 min**

UniCarb-DB: #425

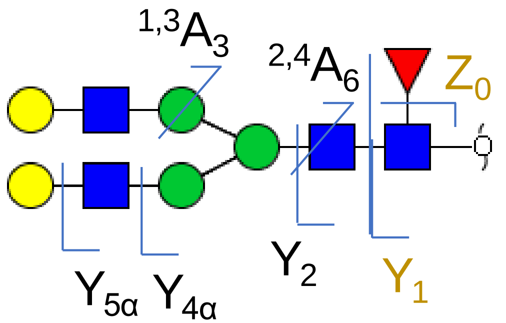

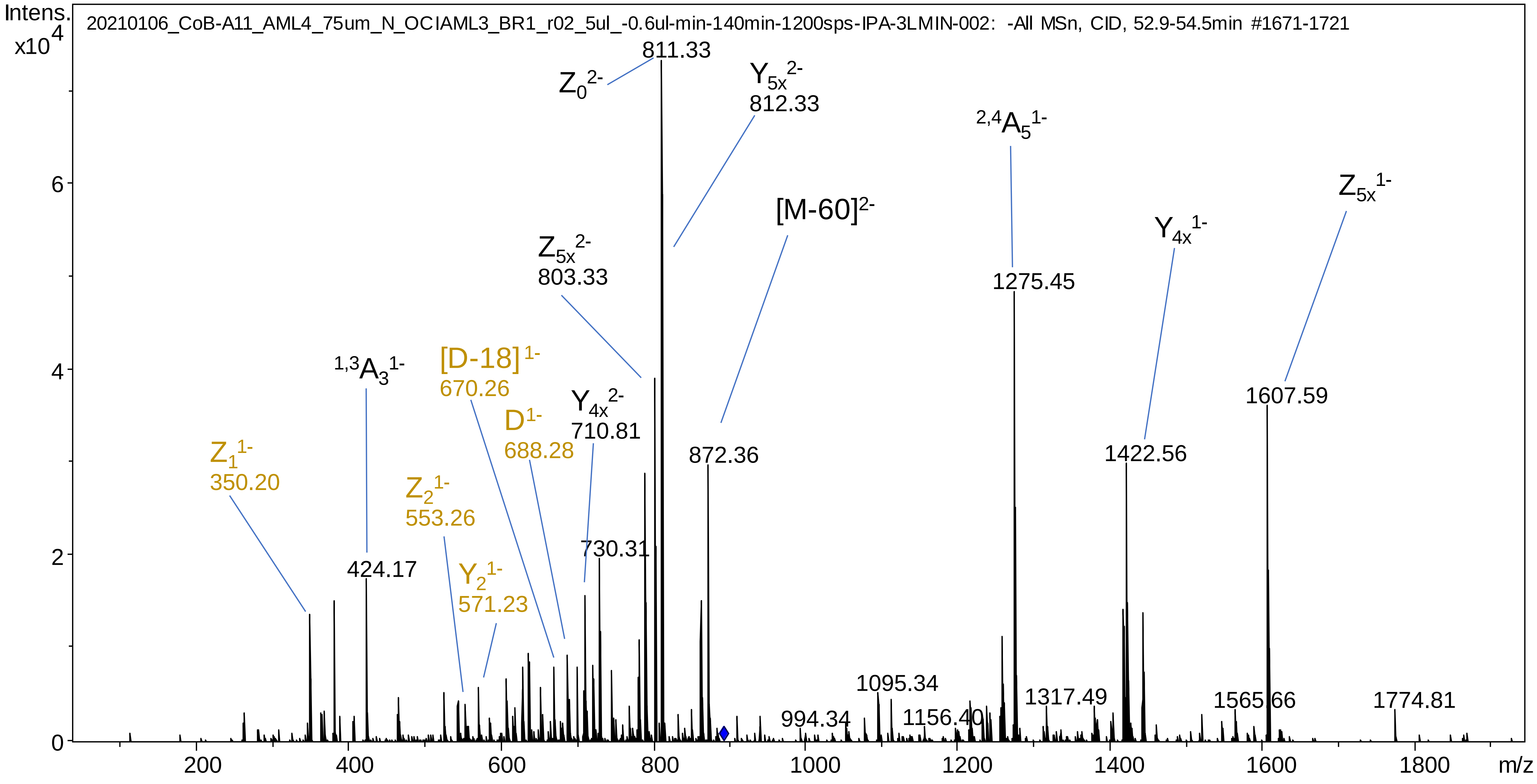

Glycan 17a

H5N4S1

Depicted MS² was obtained from analysis of cell line: OCI-AML-3

**Monoisotopic mass:**	**1933.70 Da**
**Charge observed:**	**2-**
**Theoretical ion:**	***m/z* 965.84**
**Observed ion:**	***m/z* 965.84**
**Mass deviation:**	***m/z* 0.00**
**Retention time:**	**45.7 min**

Note: α-2,6 sialic acid linkage confirmed by neuraminidase S and A treatment

UniCarb-DB: #2338

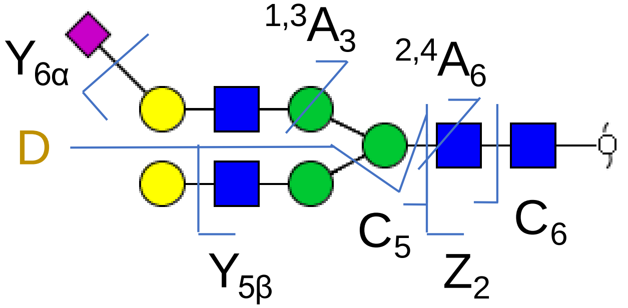

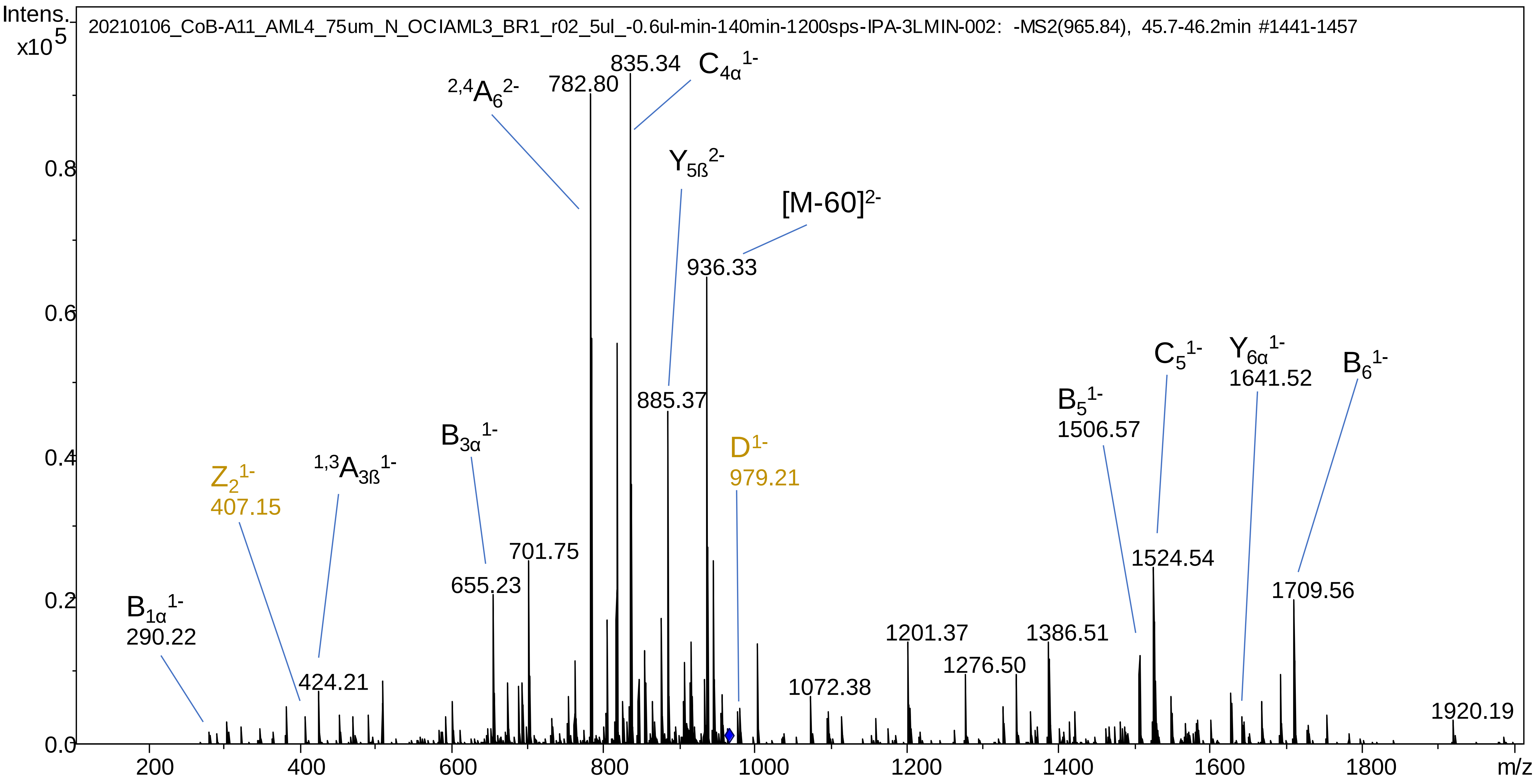

Glycan 17b

H5N4S1

Depicted MS^2 was obtained from analysis of cell line: OCI-AML-3

**Monoisotopic mass:**	**1933.70 Da**
**Charge observed:**	**2-**
**Theoretical ion:**	***m/z* 965.84**
**Observed ion:**	***m/z* 965.88**
**Mass deviation:**	***m/z* 0.04**
**Retention time:**	**55.2 min**

Note: α-2,3 sialic acid linkage confirmed by neuraminidase S and A treatment

UniCarb-DB: #2338

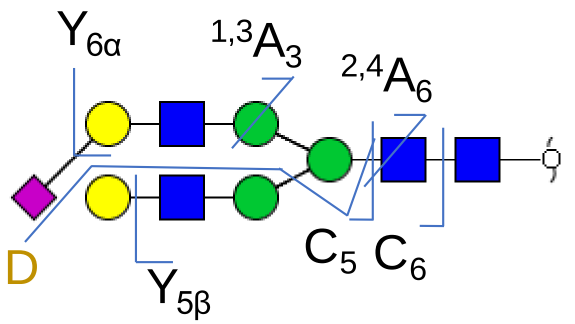

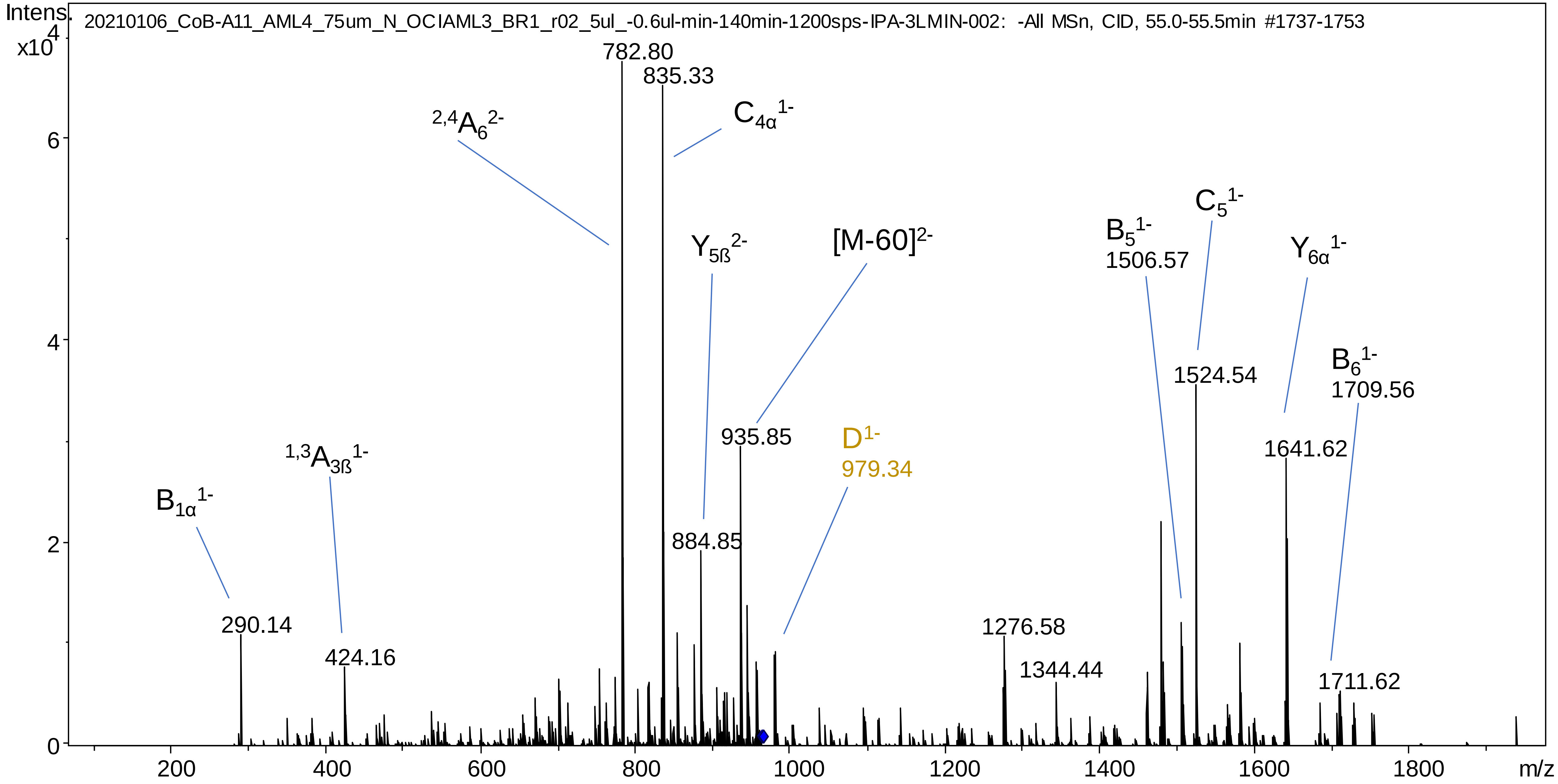

Glycan 18a

H5N4F1S1

Depicted MS[2] was obtained from analysis of cell line: MOLM-13

Monoisotopic mass: 2079.76 Da
Charge observed: 2-
Theoretical ion: *m/z* 1038.87
Observed ion: *m/z* 1038.88
Mass deviation: *m/z* 0.01
Retention time: 43.9 min
Note: α-2,6 sialic acid linkage confirmed by neuraminidase S and A treatment; no diagnostic ions for core fucosylation detected

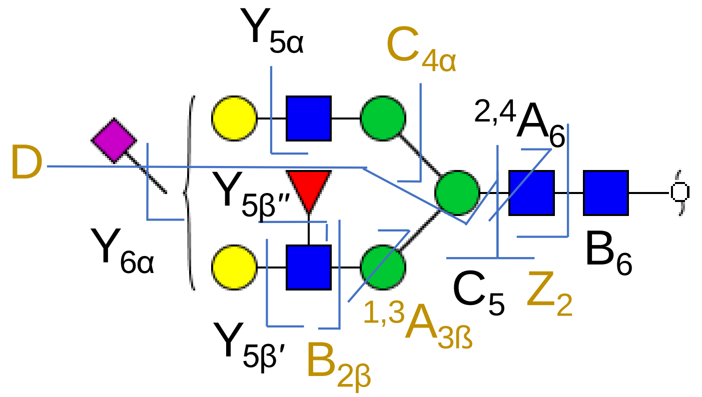

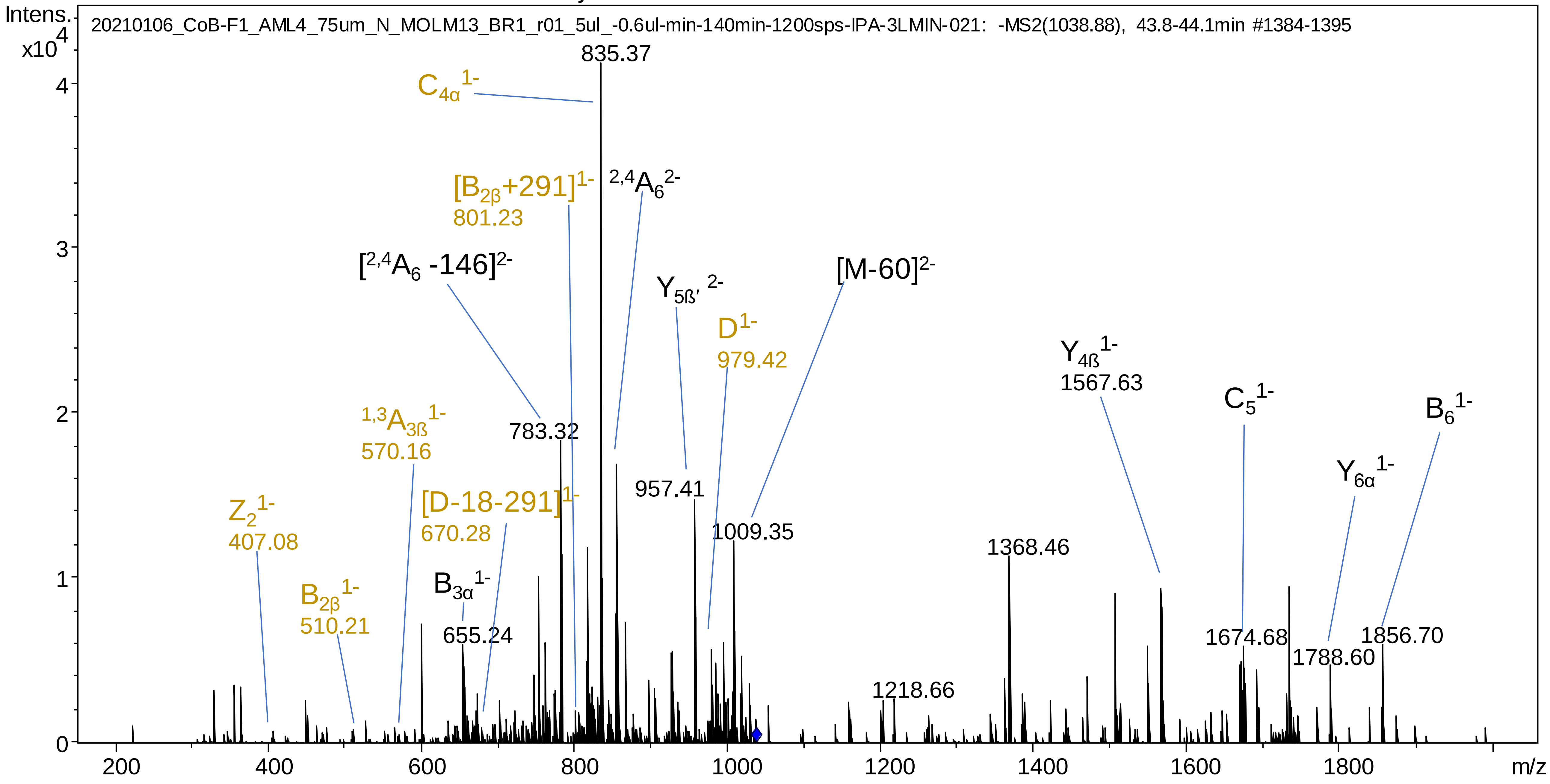

Glycan 18b

H5N4F1S1

Depicted MS² was obtained from analysis of cell line: MOLM-13

Monoisotopic mass: 2079.76 Da
Charge observed: 2-
Theoretical ion: *m/z* 1038.87
Observed ion: *m/z* 1038.88
Mass deviation: *m/z* 0.01
Retention time: 51.8 min
Note: α-2,6 sialic acid linkage confirmed by neuraminidase S and A treatment

UniCarb-DB: #2364

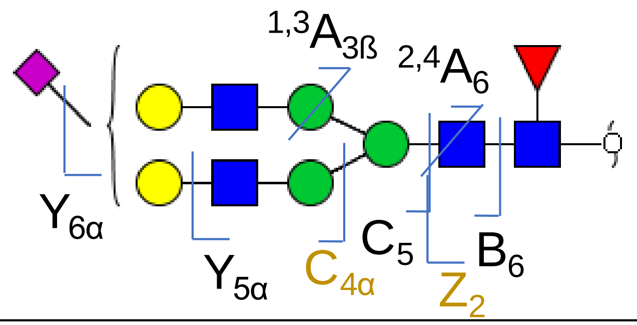

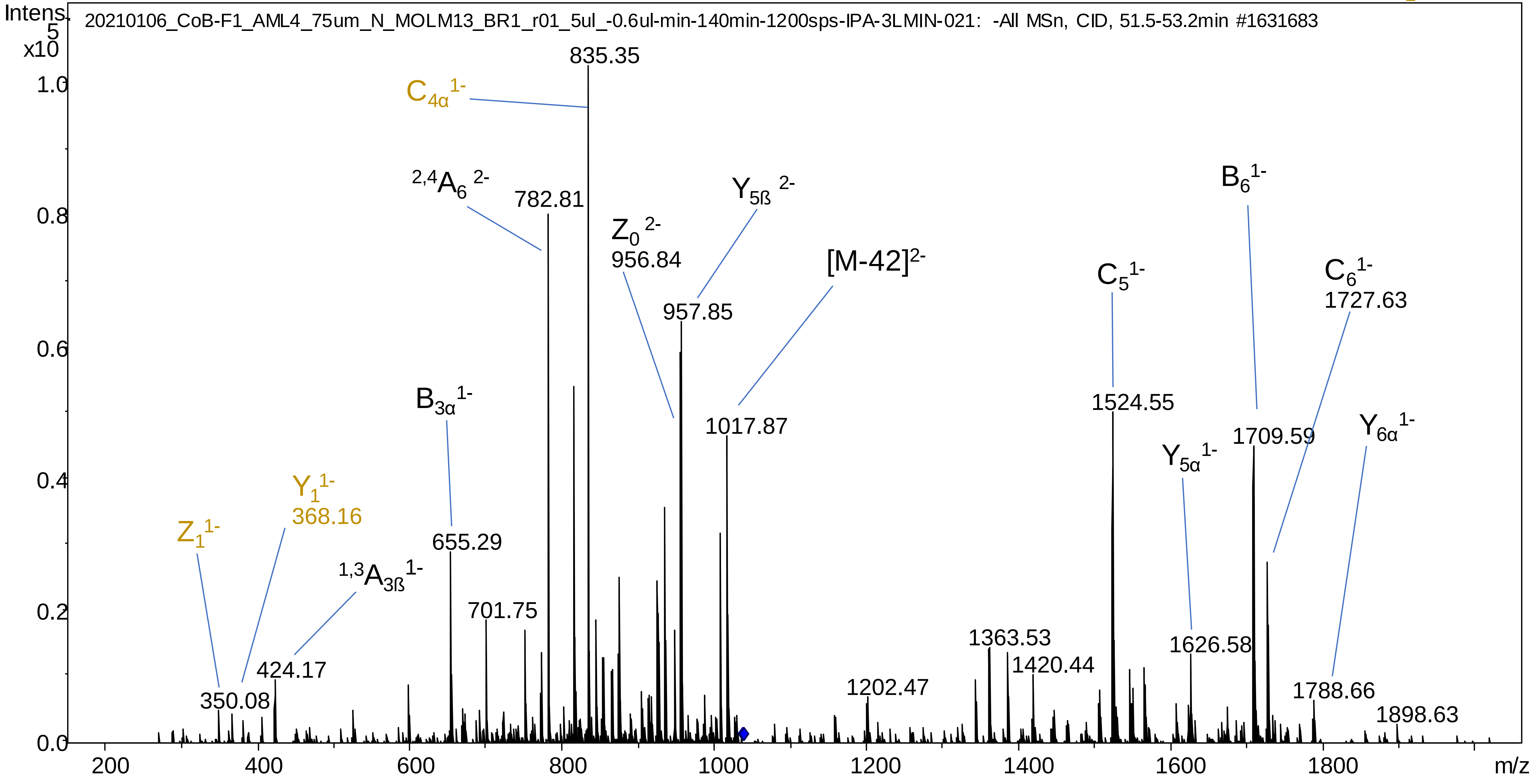

Glycan 18c

H5N4F1S1

Depicted MS² was obtained from analysis of cell line: MOLM-13

Monoisotopic mass: 2079.76 Da
Charge observed: 2-
Theoretical ion: *m/z* 1038.87
Observed ion: *m/z* 1038.88
Mass deviation: *m/z* 0.01
Retention time: 60.8 min
Note: α-2,3 sialic acid linkage confirmed by neuraminidase S and A treatment

UniCarb-DB: #2364

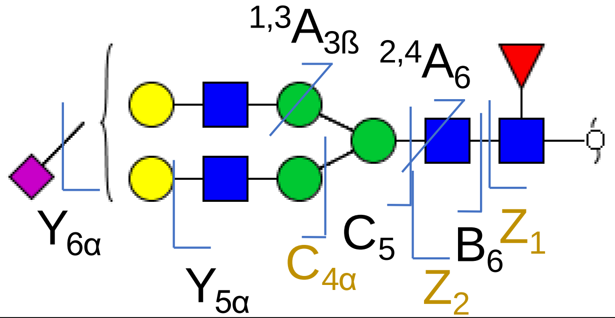

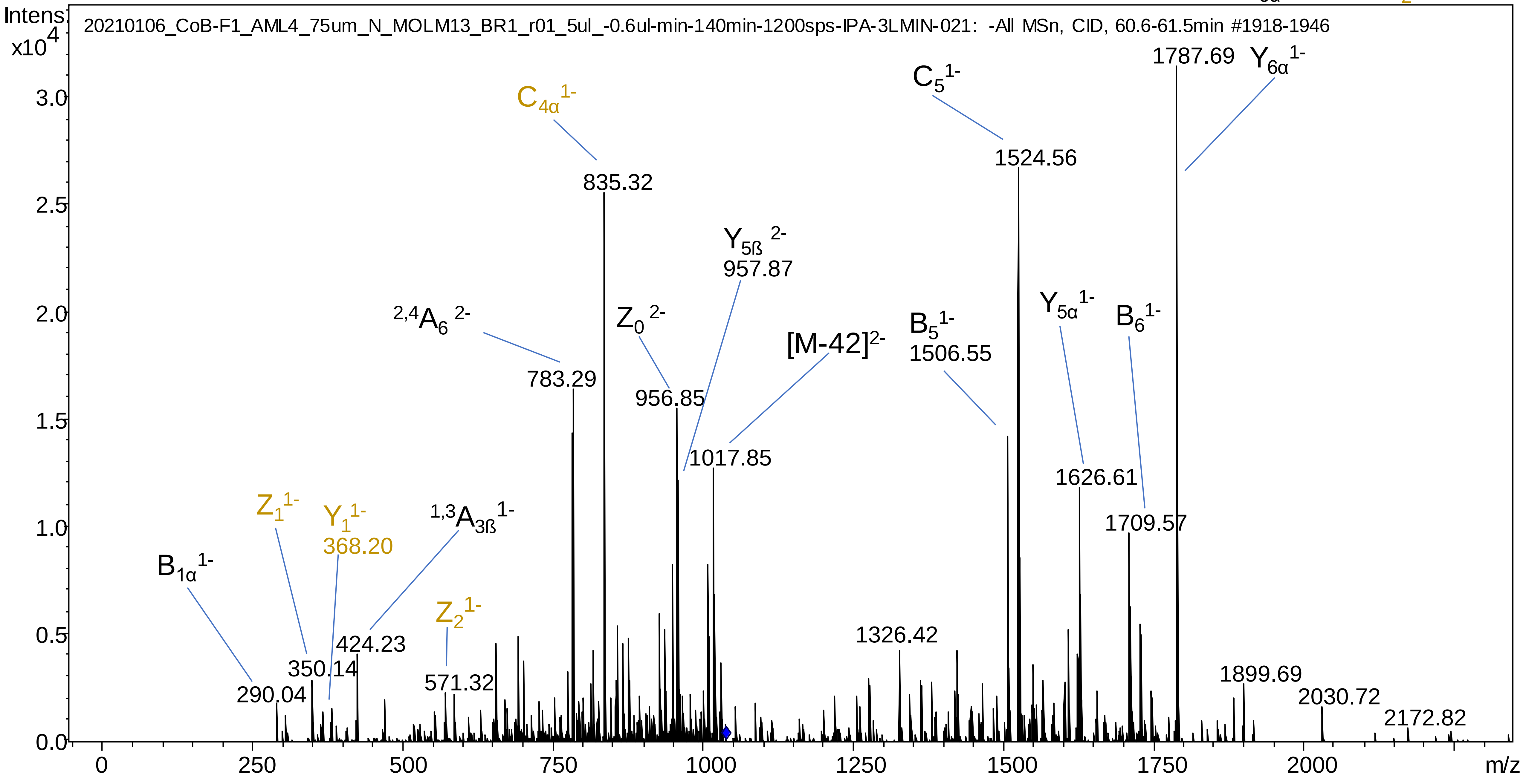

Glycan 19

H6N5F1

Depicted MS^2 was obtained from analysis of cell line: OCI-AML-3

**Monoisotopic mass:**	**2153.80 Da**
**Charge observed:**	**2-**
**Theoretical ion:**	***m/z* 1075.89**
**Observed ion:**	***m/z* 1075.88**
**Mass deviation:**	***m/z* 0.01**
**Retention time:**	**60.8 min**

UniCarb-DB: #2549

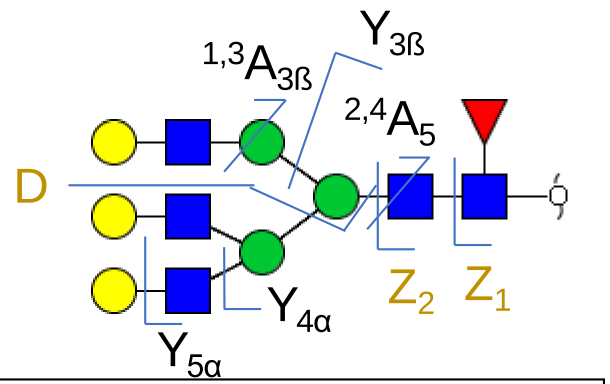

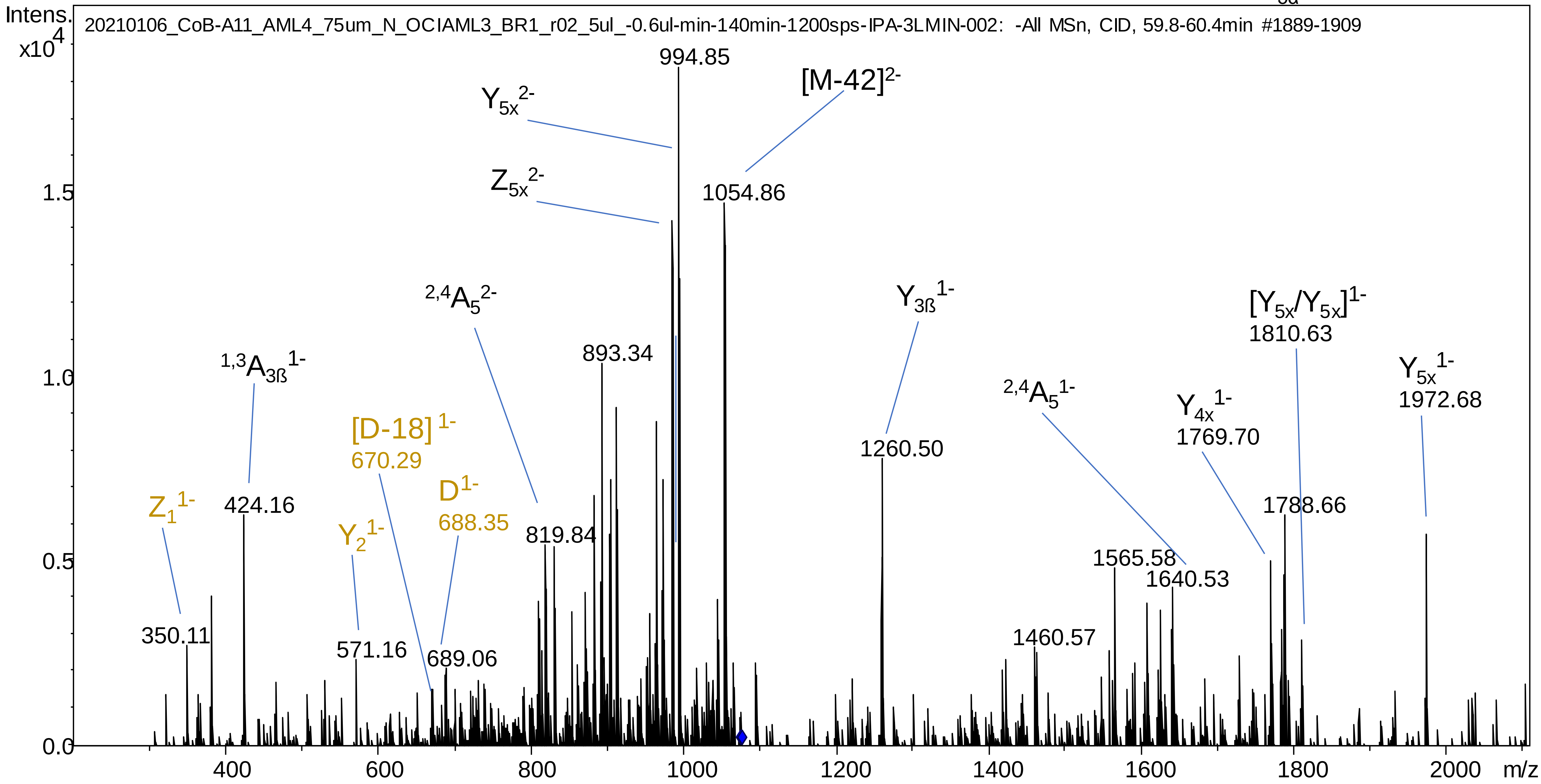

Glycan 20a

H5N4S2

Depicted MS^2 was obtained from analysis of cell line: MOLM-13

Monoisotopic mass: **2224.80 Da**
Charge observed: **2-**
Theoretical ion: ***m/z* 1111.39**
Observed ion: ***m/z* 1111.38**
Mass deviation: ***m/z* 0.01**
Retention time: **45.2 min**
Note: α-2,6 sialic acid linkages confirmed by neuraminidase S and A treatment

UniCarb-DB: #378

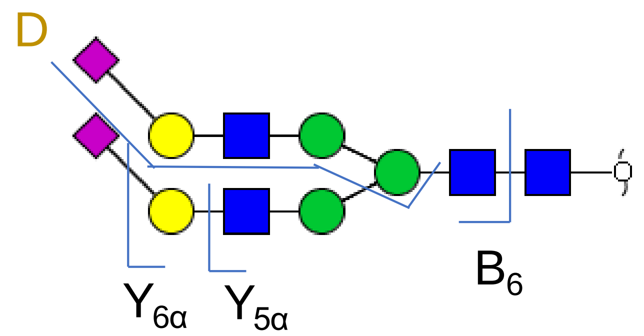

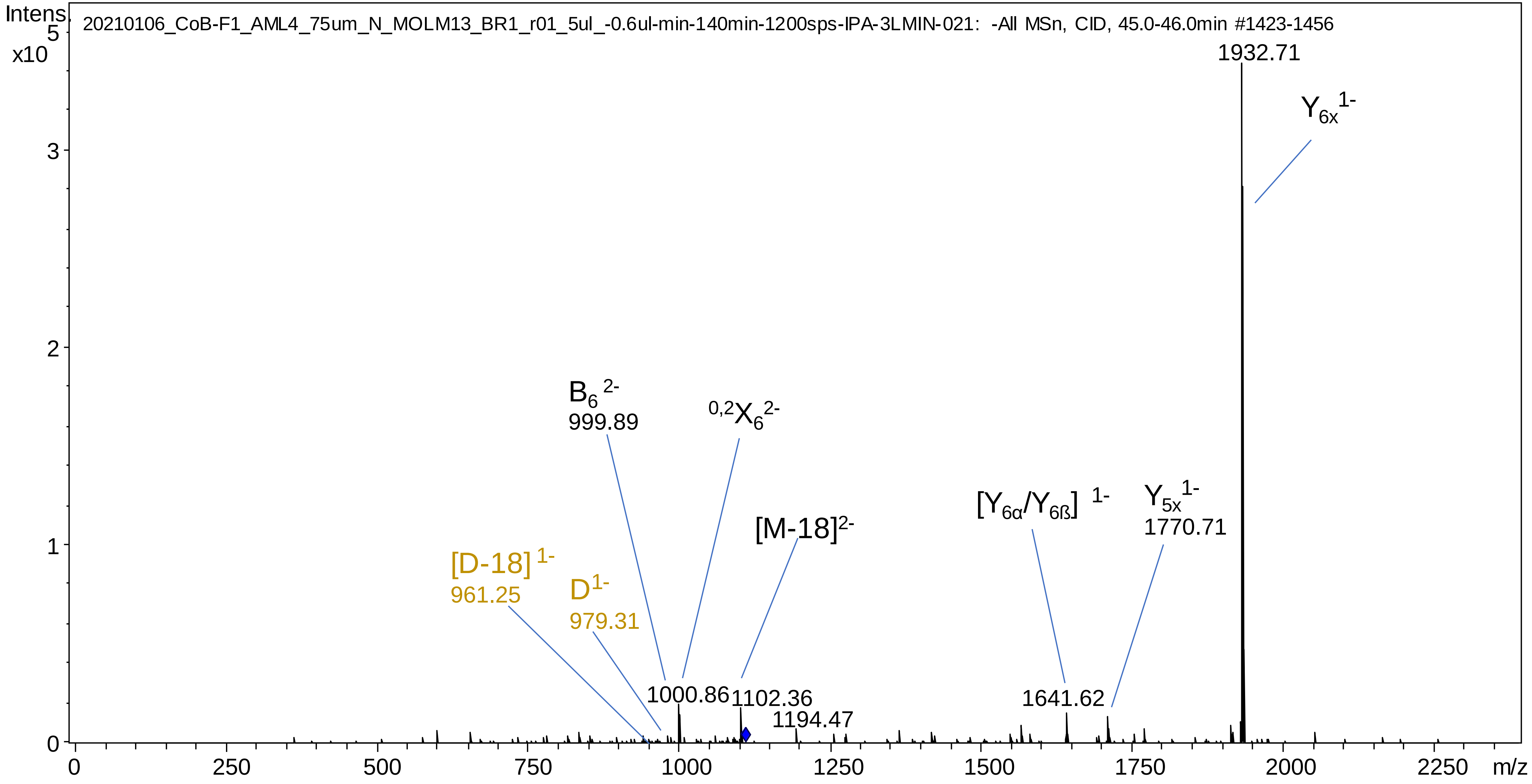

Glycan 20b

H5N4S2

Depicted MS[2] was obtained from analysis of cell line: MOLM-13

Monoisotopic mass: 2224.80 Da
Charge observed: 2-
Theoretical ion: *m/z* 1111.39
Observed ion: *m/z* 1111.40
Mass deviation: *m/z* 0.01
Retention time: 54.1 min
Note: Sialic acid linkages confirmed by neuraminidase S and A treatment

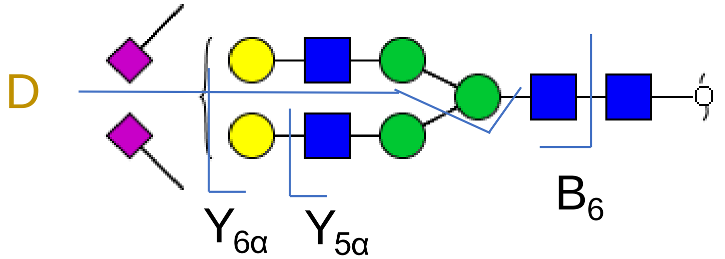

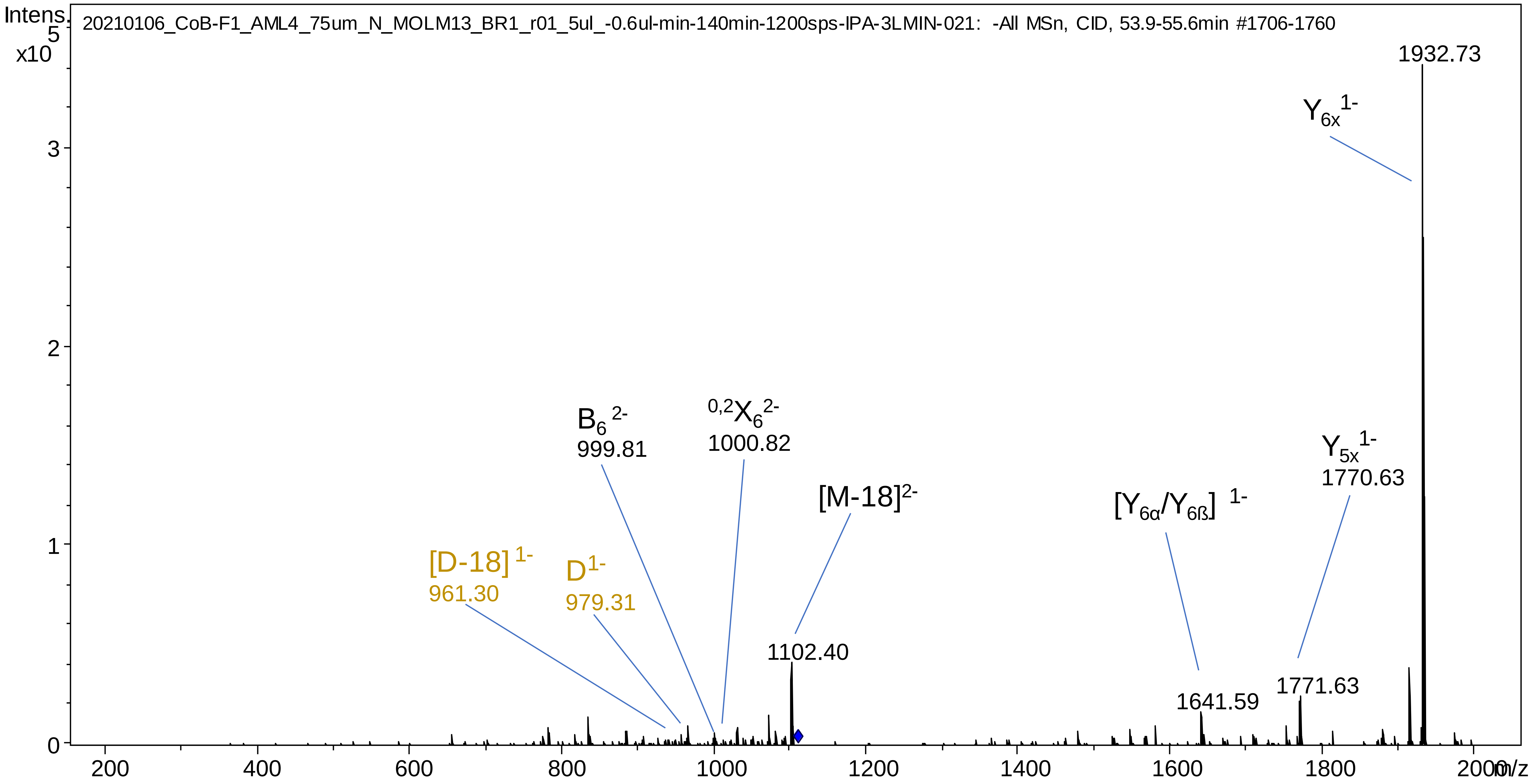

Glycan 20c

H5N4S2

Depicted MS² was obtained from analysis of cell line: KG-1a

Monoisotopic mass: 2224.80 Da
Charge observed: 2-
Theoretical ion: *m/z* 1111.42
Observed ion: *m/z* 1111.39
Mass deviation: *m/z* 0.01
Retention time: 61.7 min
Note: α-2,6 sialic acid linkages confirmed by neuraminidase S and A treatment

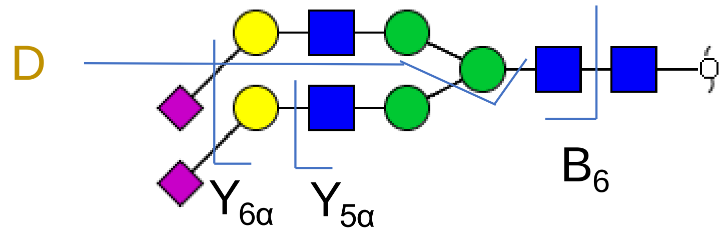

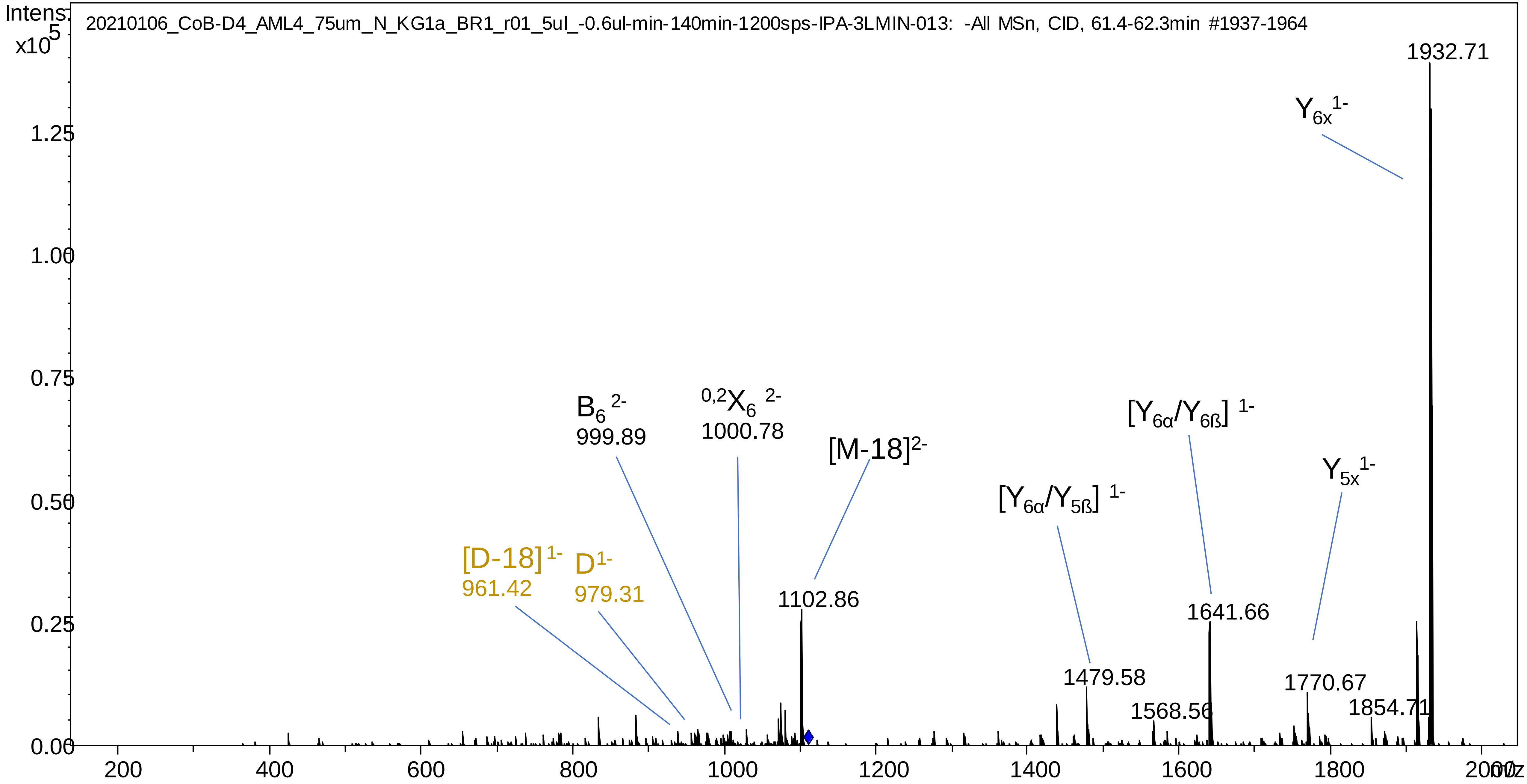

Glycan 21

H5N4F2S1

Depicted MS² was obtained from analysis of cell line: MOLM-13

**Monoisotopic mass:**	**2225.82 Da**
**Charge observed:**	**2-**
**Theoretical ion:**	***m/z* 1111.90**
**Observed ion:**	***m/z* 1111.90**
**Mass deviation:**	***m/z* 0.00**
**Retention time:**	**50.1 min**

Note: α-2,6 sialic acid linkages confirmed by neuraminidase S and A treatment

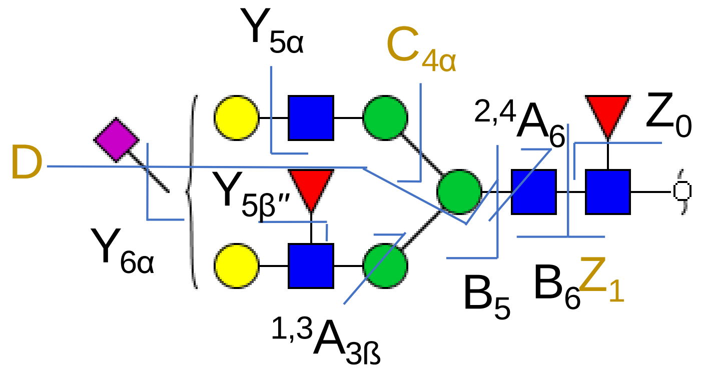

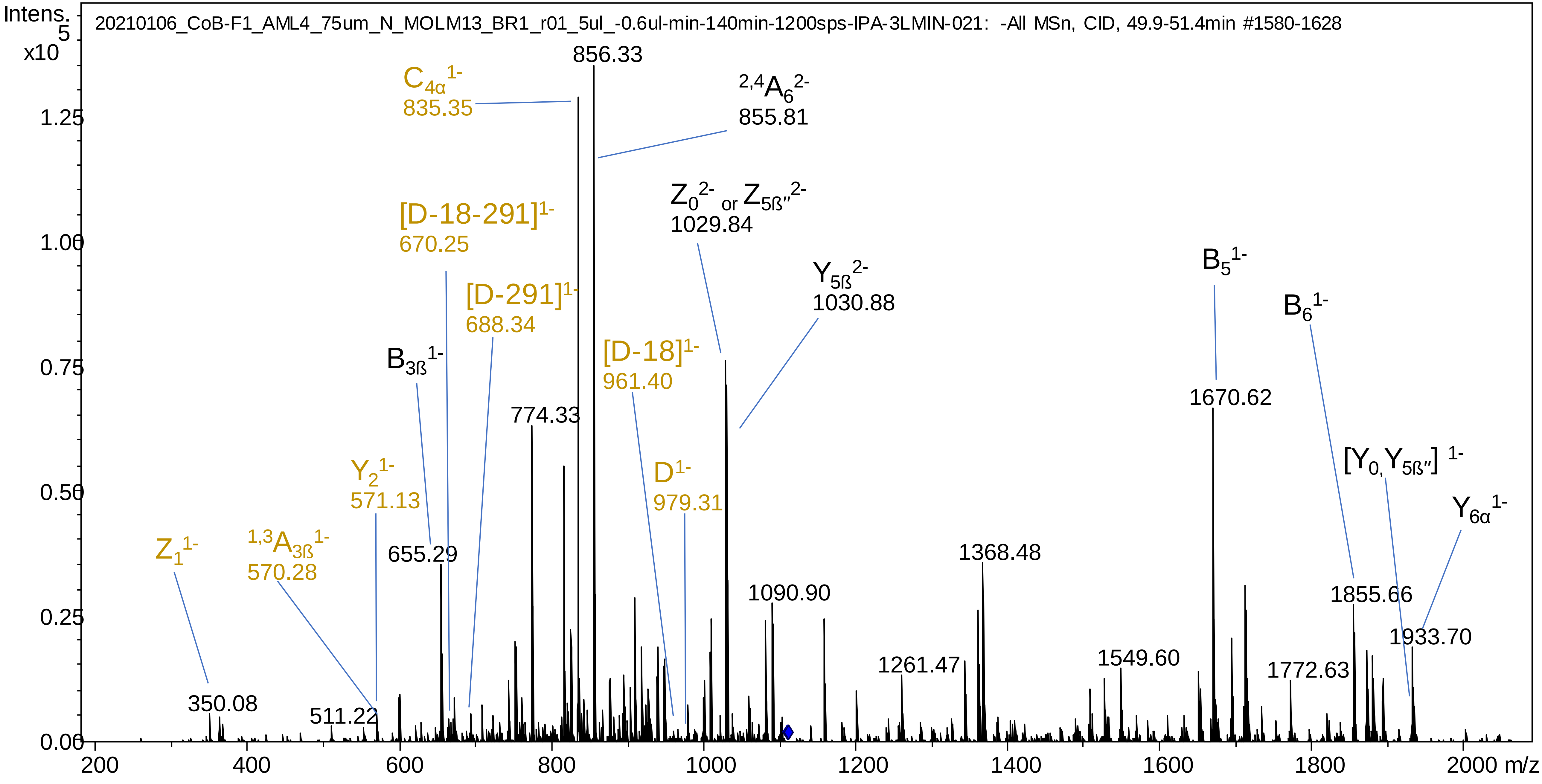

Glycan 22a

H5N4S2F1

Depicted MS[2] was obtained from analysis of cell line: TF-1

Monoisotopic mass: 2370.86 Da
Charge observed: 2-
Theoretical ion: *m/z* 1184.42
Observed ion: *m/z* 1184.43
Mass deviation: *m/z* 0.01
Retention time: 50.8 min
Note: α-2,6 sialic acid linkages confirmed by neuraminidase S and A treatment

UniCarb-DB: #389

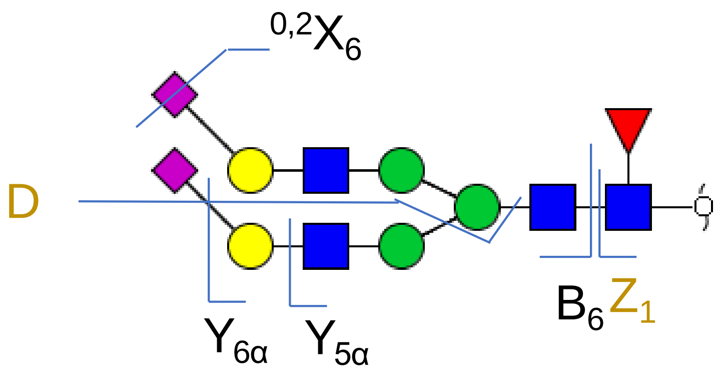

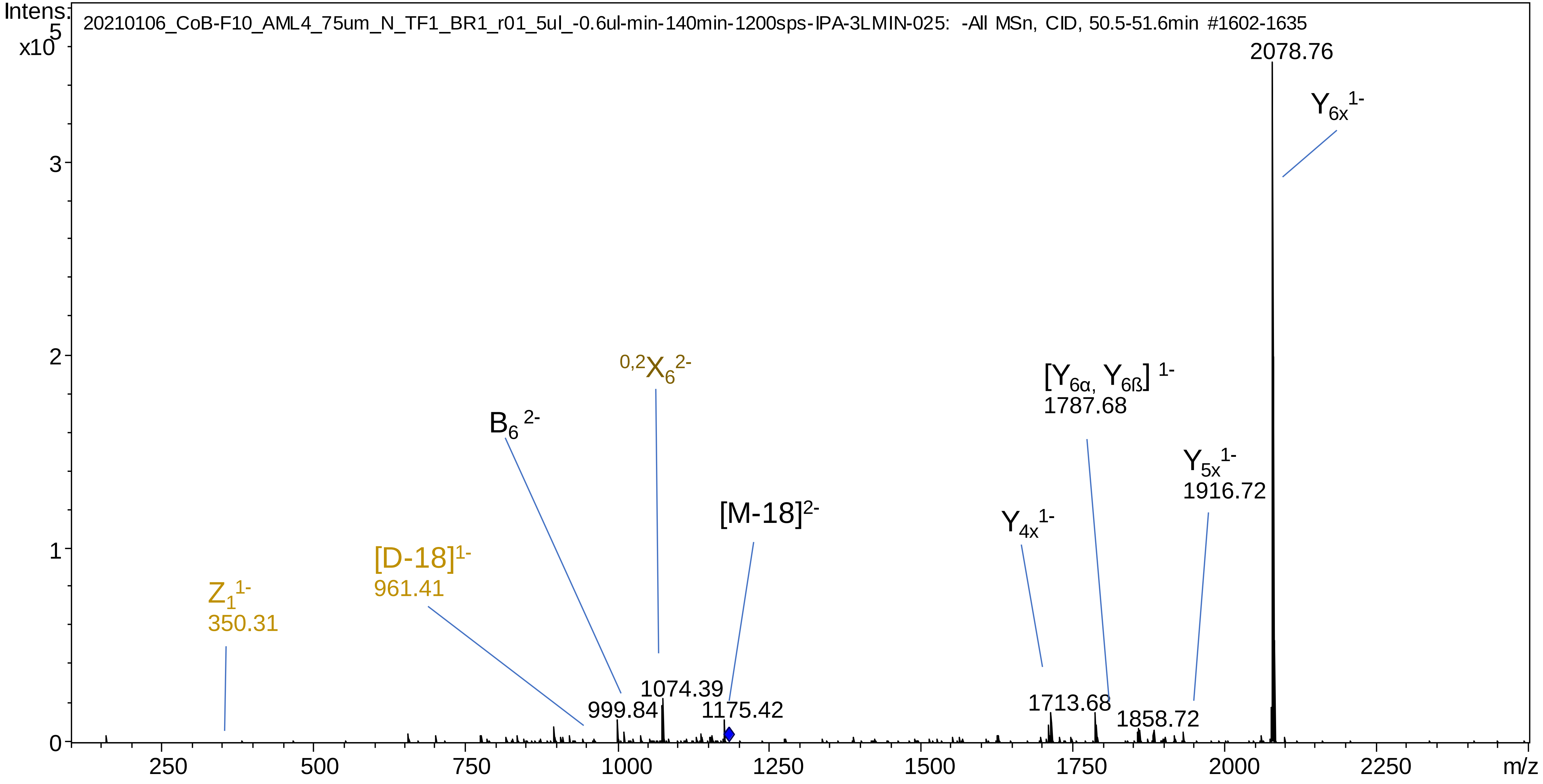

Glycan 22b

H5N4S2F1

Depicted MS^2 was obtained from analysis of cell line: TF-1

**Monoisotopic mass:**	**2370.86 Da**
**Charge observed:**	**2-**
**Theoretical ion:**	***m/z* 1184.42**
**Observed ion:**	***m/z* 1184.44**
**Mass deviation:**	***m/z* 0.02**
**Retention time:**	**59.4 min**

Note: Sialic acid linkages confirmed by neuraminidase S and A treatment

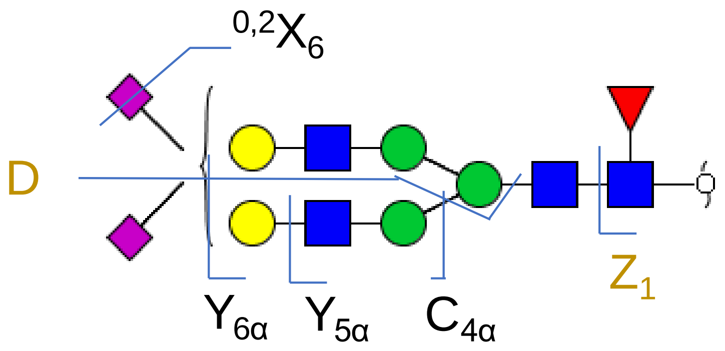

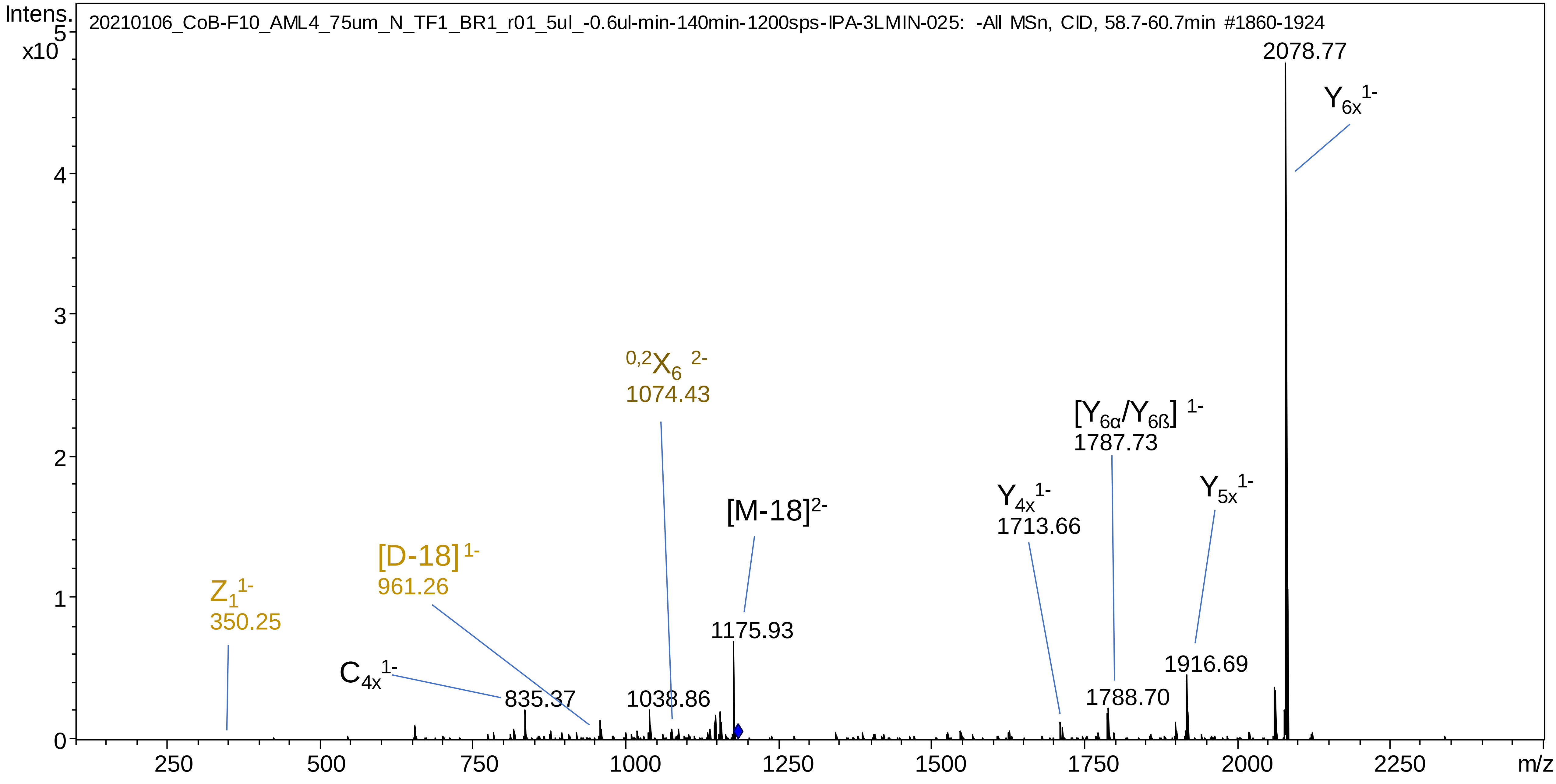

Glycan 22c

H5N4S2F1

Depicted MS² was obtained from analysis of cell line: TF-1

Monoisotopic mass: 2370.86 Da
Charge observed: 2-
Theoretical ion: *m/z* 1184.42
Observed ion: *m/z* 1184.44
Mass deviation: *m/z* 0.02
Retention time: 66.6 min
Note: Sialic acid linkages confirmed by neuraminidase S and A treatment

UniCarb-DB: #2563

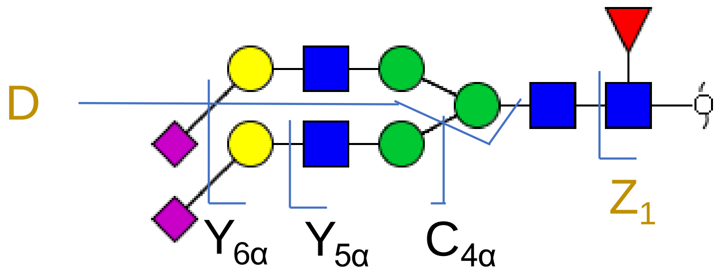

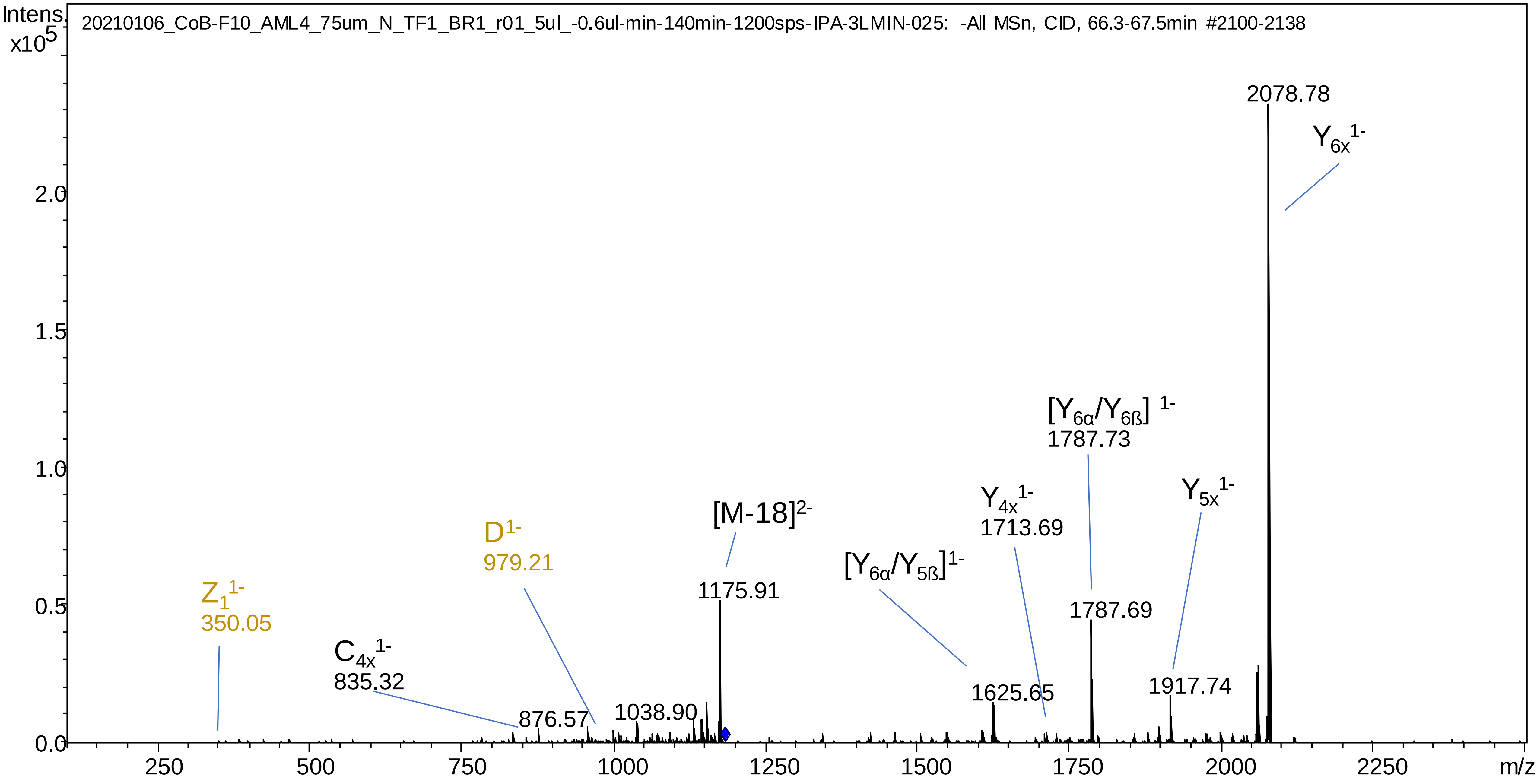

Glycan 23

H6N5F1S1

Depicted MS² was obtained from analysis of cell line: M-07e

Monoisotopic mass: 2444.89 Da
Charge observed: 2-
Theoretical ion: *m/z* 1221.45
Observed ion: *m/z* 1221.45
Mass deviation: *m/z* 0.00
Retention time: 60.9 min
Note: α-2,6 sialic acid linkage confirmed by neuraminidase S and A treatment

UniCarb-DB: #2566

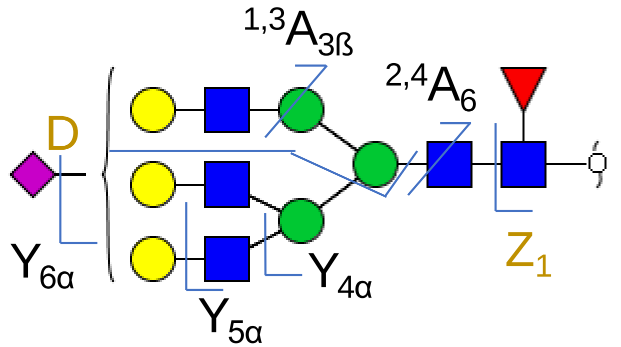

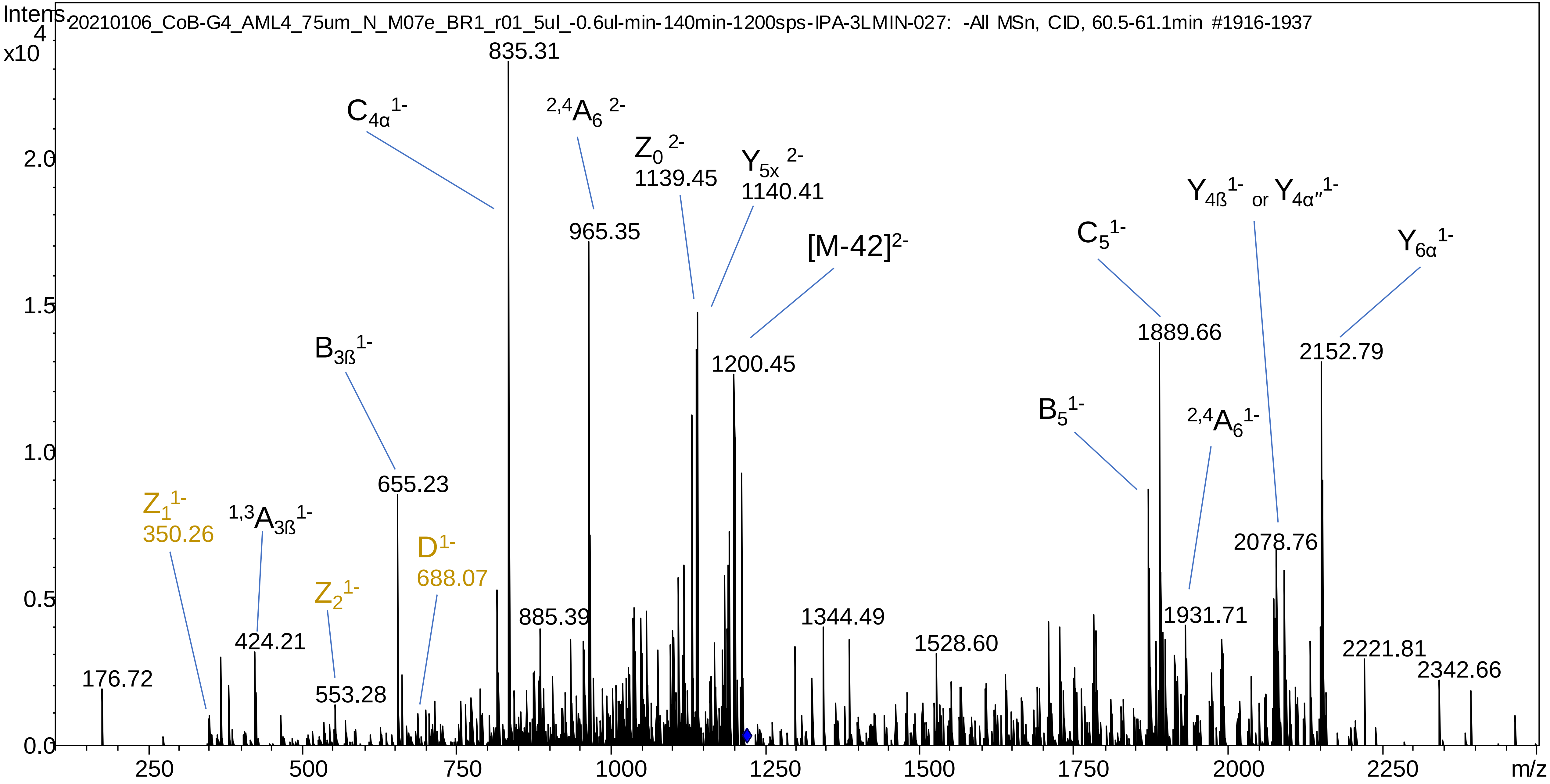

Glycan 24a

H5N4S2F2

Depicted MS[2] was obtained from analysis of cell line: M-07e

**Monoisotopic mass:**	**2516.92 Da**
**Charge observed:**	**2-**
**Theoretical ion:**	***m/z* 1257.45**
**Observed ion:**	***m/z* 1257.46**
**Mass deviation:**	***m/z* 0.01**
**Retention time:**	**53.4 min**

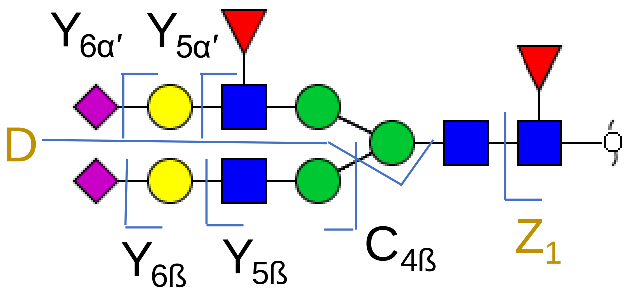

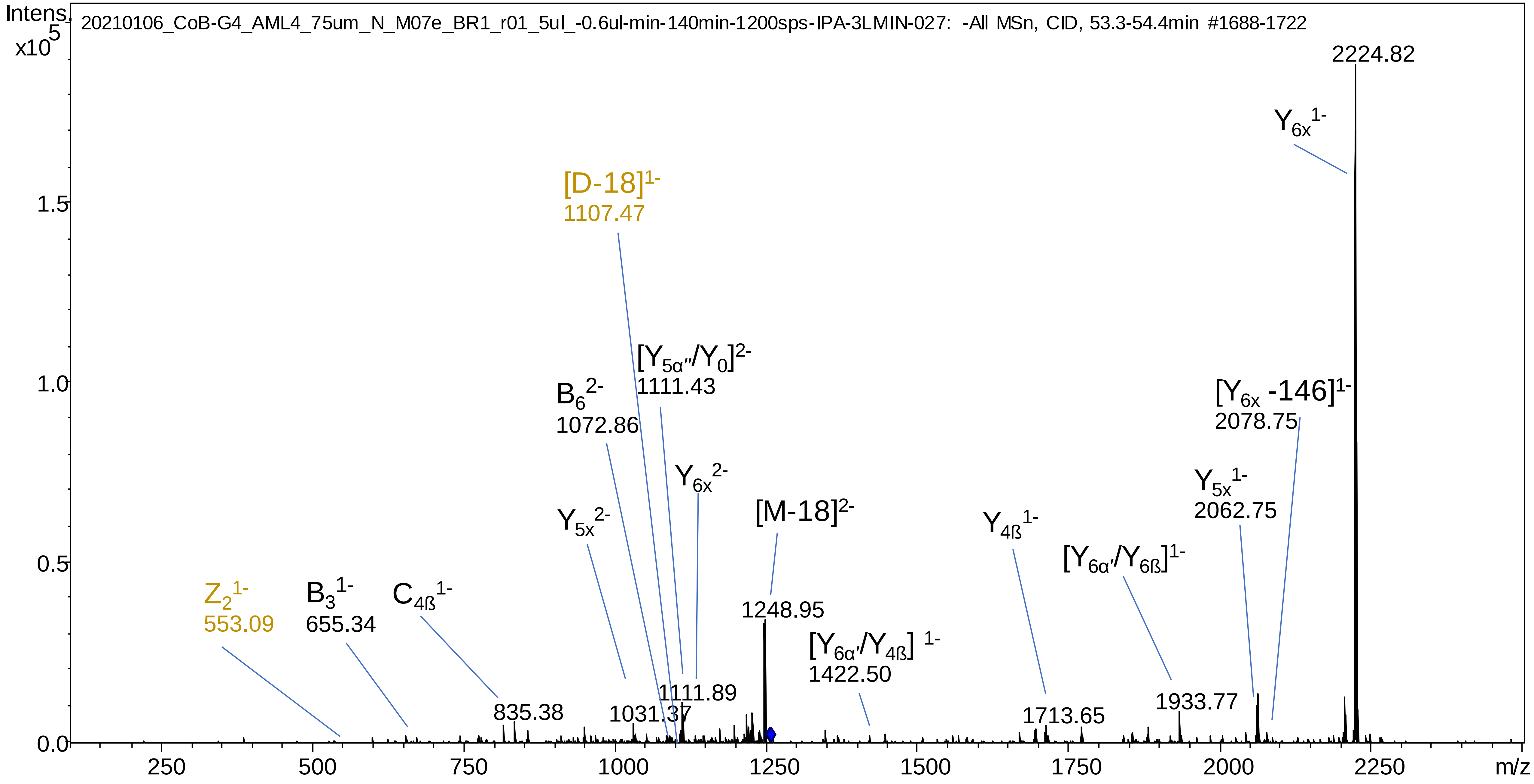

Glycan 24b

H5N4S2F2

Depicted MS² was obtained from analysis of cell line: M-07e

Monoisotopic mass: 2516.92 Da
Charge observed: 2-
Theoretical ion: *m/z* 1257.45
Observed ion: *m/z* 1257.48
Mass deviation: *m/z* 0.03
Retention time: 59.9 min

UniCarb-DB: #2254

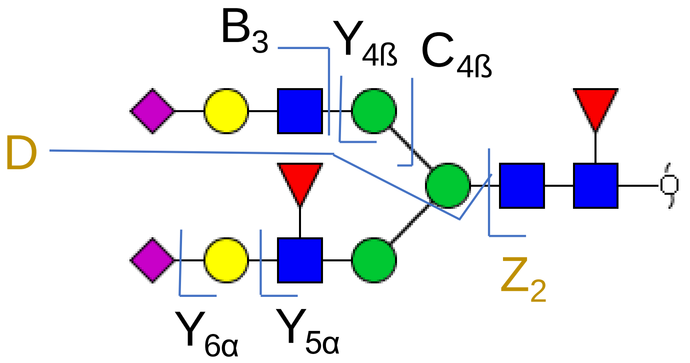

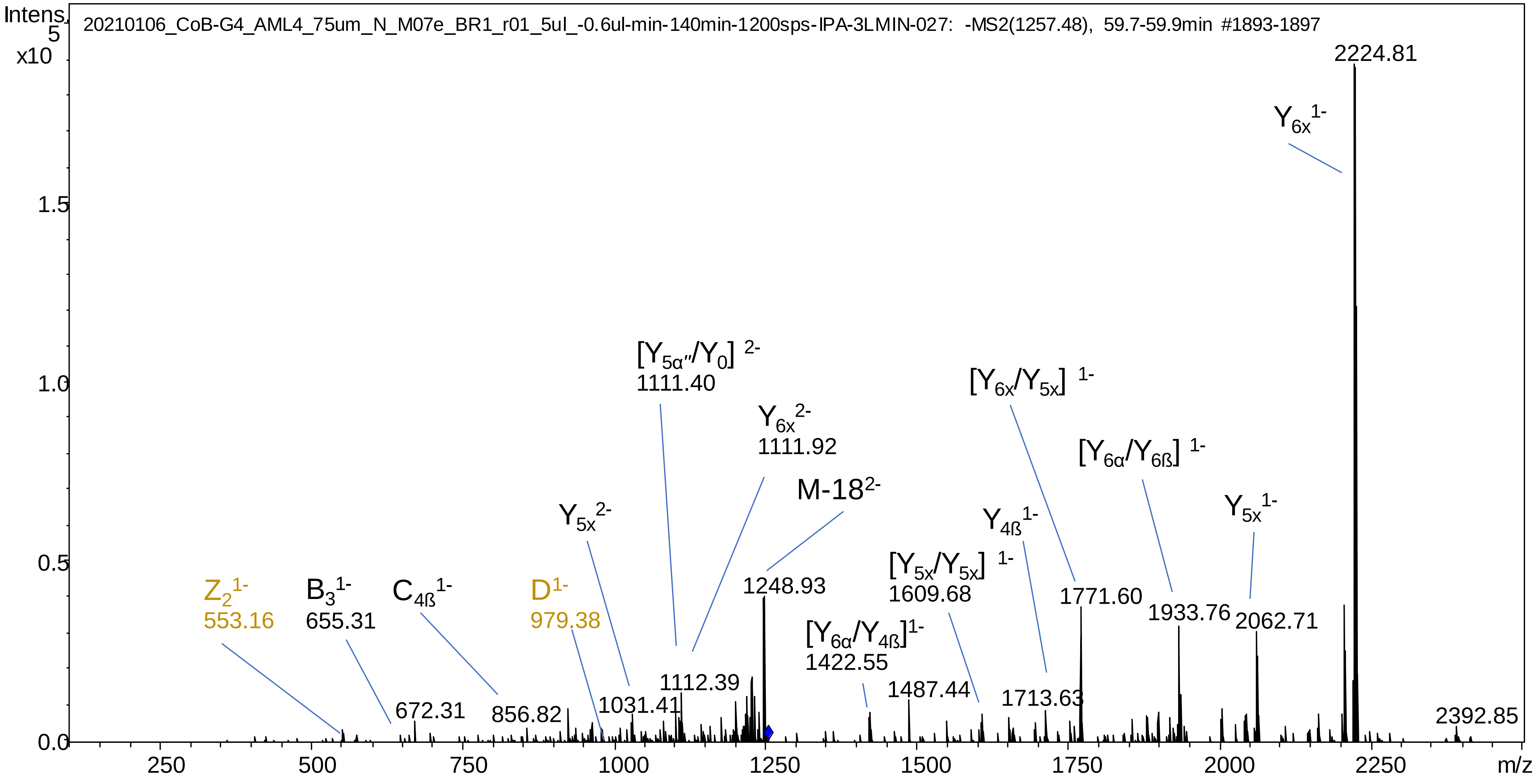

Glycan 24c

H5N4S2F2

Depicted MS² was obtained from analysis of cell line: KG-1a

**Monoisotopic mass:**	**2516.92 Da**
**Charge observed:**	**2-**
**Theoretical ion:**	***m/z* 1257.45**
**Observed ion:**	***m/z* 1257.46**
**Mass deviation:**	***m/z* 0.01**
**Retention time:**	**62.1 min**

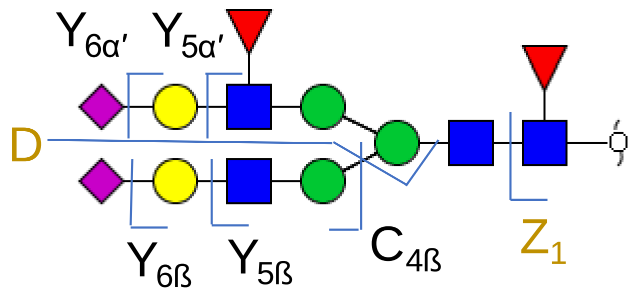

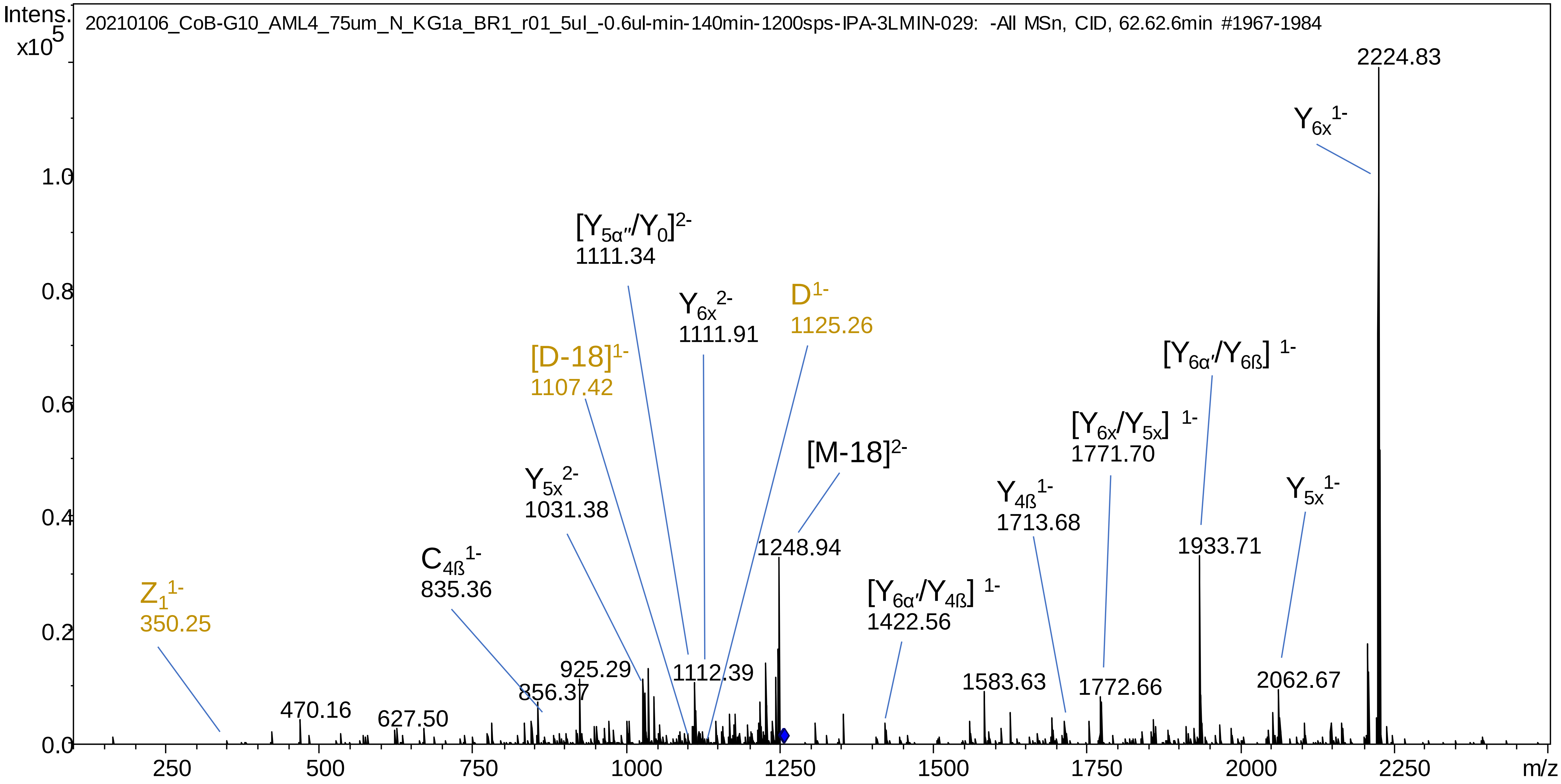

Glycan 25

H7N6F1

Depicted MS² was obtained from analysis of cell line: HL-60

**Monoisotopic mass:**	**2518.93 Da**
**Charge observed:**	**2-**
**Theoretical ion:**	***m/z* 1258.46**
**Observed ion:**	***m/z* 1258.45**
**Mass deviation:**	***m/z* 0.01**
**Retention time:**	**61.7 min**

UniCarb-DB: #2417

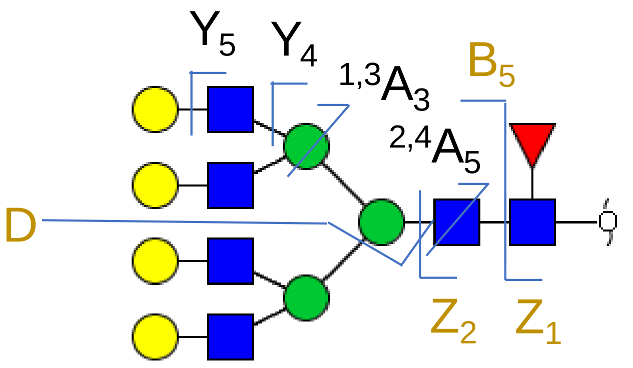

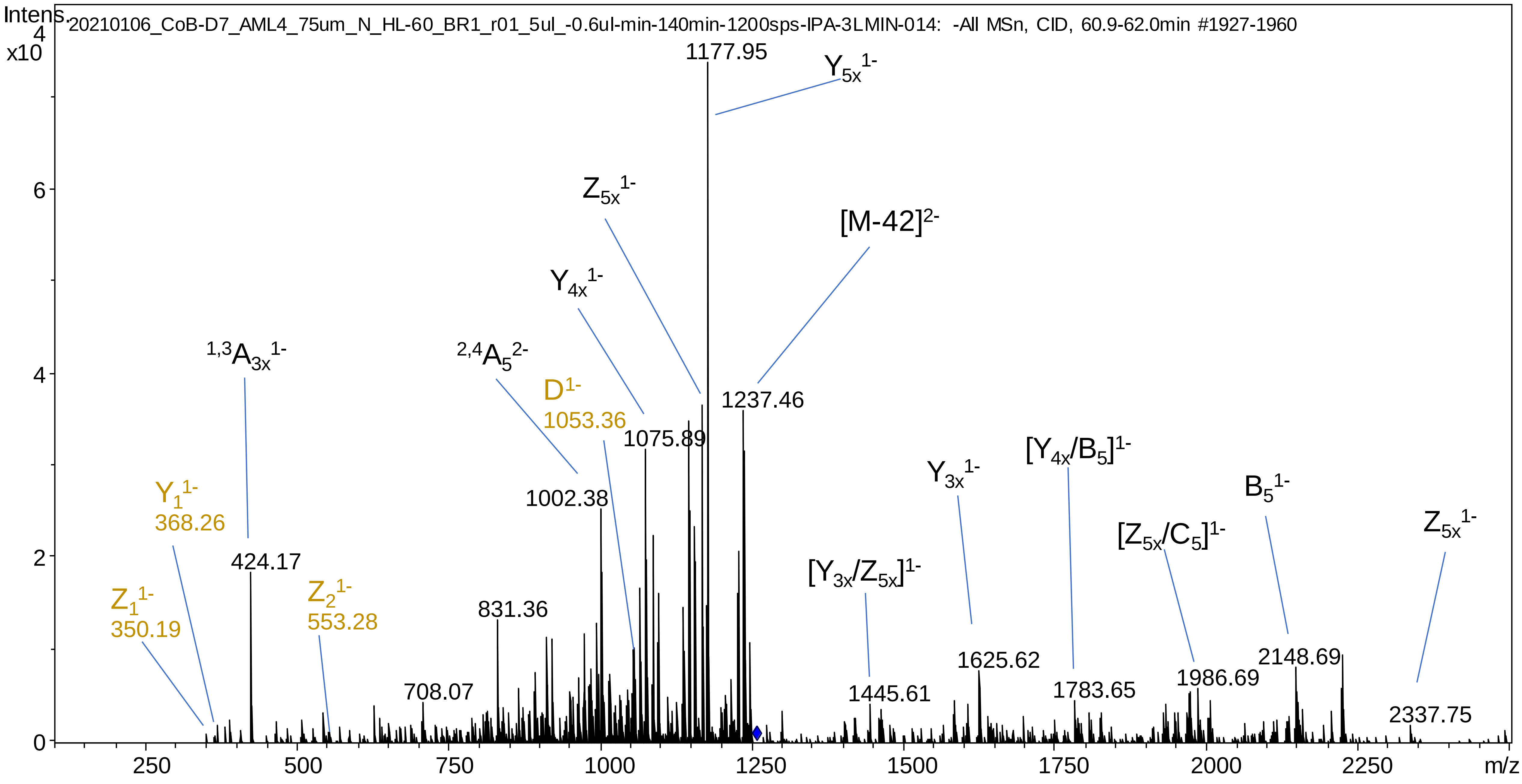

Glycan 26a

H6N5S2

Depicted MS[2] was obtained from analysis of cell line: MV4-11

Monoisotopic mass: **2589.93 Da**
Charge observed: **2-**
Theoretical ion: ***m/z* 1293.96**
Observed ion: ***m/z* 1293.96**
Mass deviation: ***m/z* 0.00**
Retention time: **51.4 min**
Note: α-2,6 sialic acid linkages confirmed by neuraminidase S and A treatment

UniCarb-DB: #2420

Retention time fits to glycan isomer identifed in Fetuin standard (G. Palmisano et al. Rsc Adv, 2013, 3, 22706-22726).

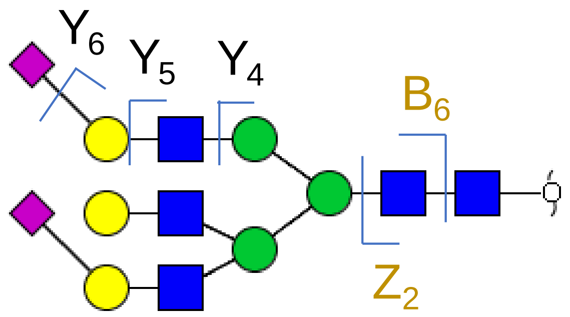

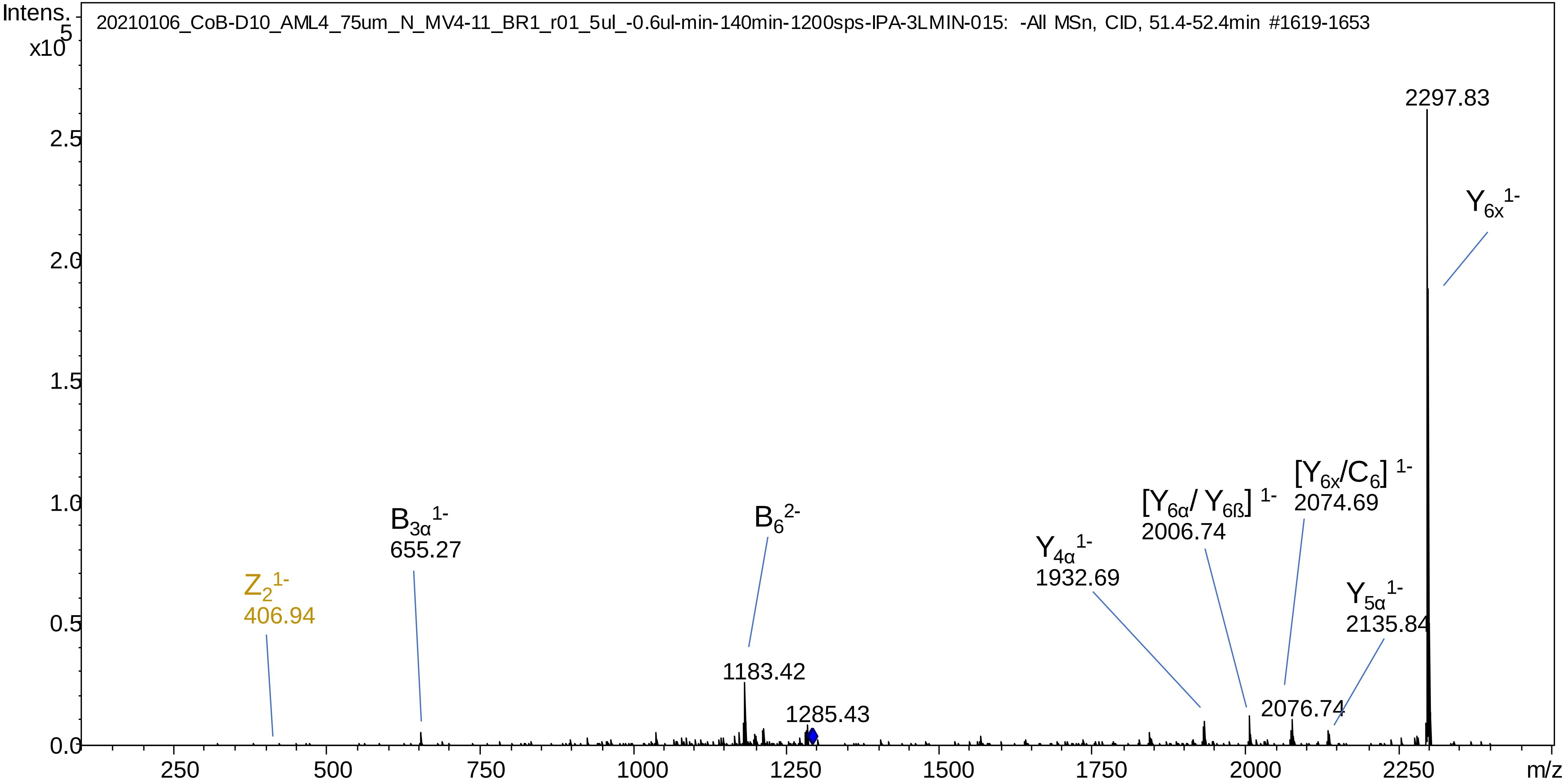

Glycan 26b

H6N5S2

Depicted MS² was obtained from analysis of cell line: MV4-11

Monoisotopic mass: 2589.93 Da
Charge observed: 2-
Theoretical ion: *m/z* 1293.96
Observed ion: *m/z* 1293.96
Mass deviation: *m/z* 0.00
Retention time: 56.1 min
Note: Sialic acid linkages confirmed by neuraminidase S and A treatment

UniCarb-DB: #2420

Retention time fits to glycan isomer identifed in Fetuin standard (G. Palmisano et al. Rsc Adv, 2013, 3, 22706-22726).

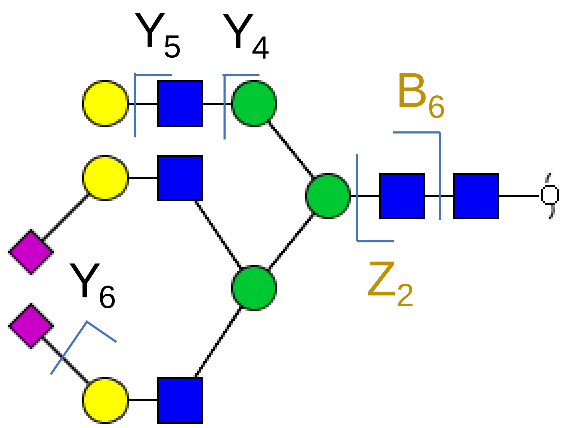

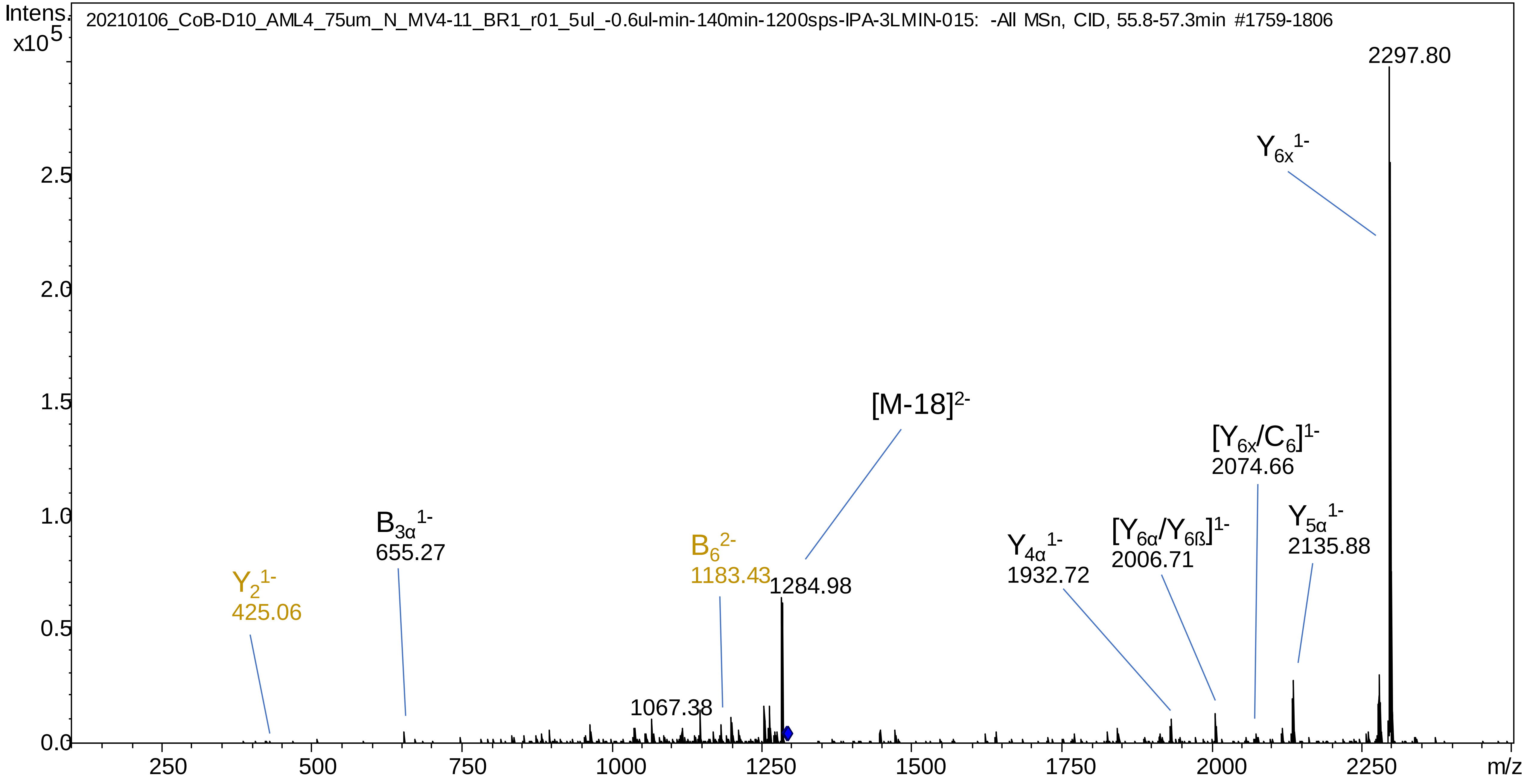

Glycan 26c

H6N5S2

Depicted MS² was obtained from analysis of cell line: MV4-11

**Monoisotopic mass:**	**2589.93 Da**
**Charge observed:**	**2-**
**Theoretical ion:**	***m/z* 1293.96**
**Observed ion:**	***m/z* 1293.95**
**Mass deviation:**	***m/z* 0.01**
**Retention time:**	**60.5 min**

Note: α-2,3 sialic acid linkages confirmed by neuraminidase S and A treatment

UniCarb-DB: #2420

Retention time fits to glycan isomer identifed in Fetuin standard (G. Palmisano et al. Rsc Adv, 2013, 3, 22706-22726).

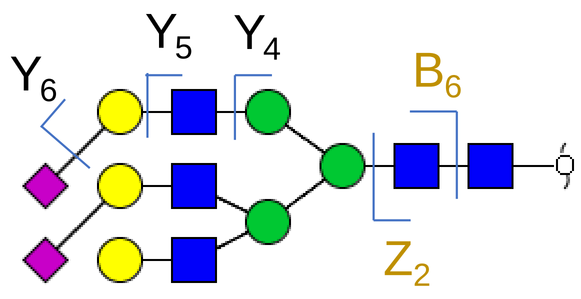

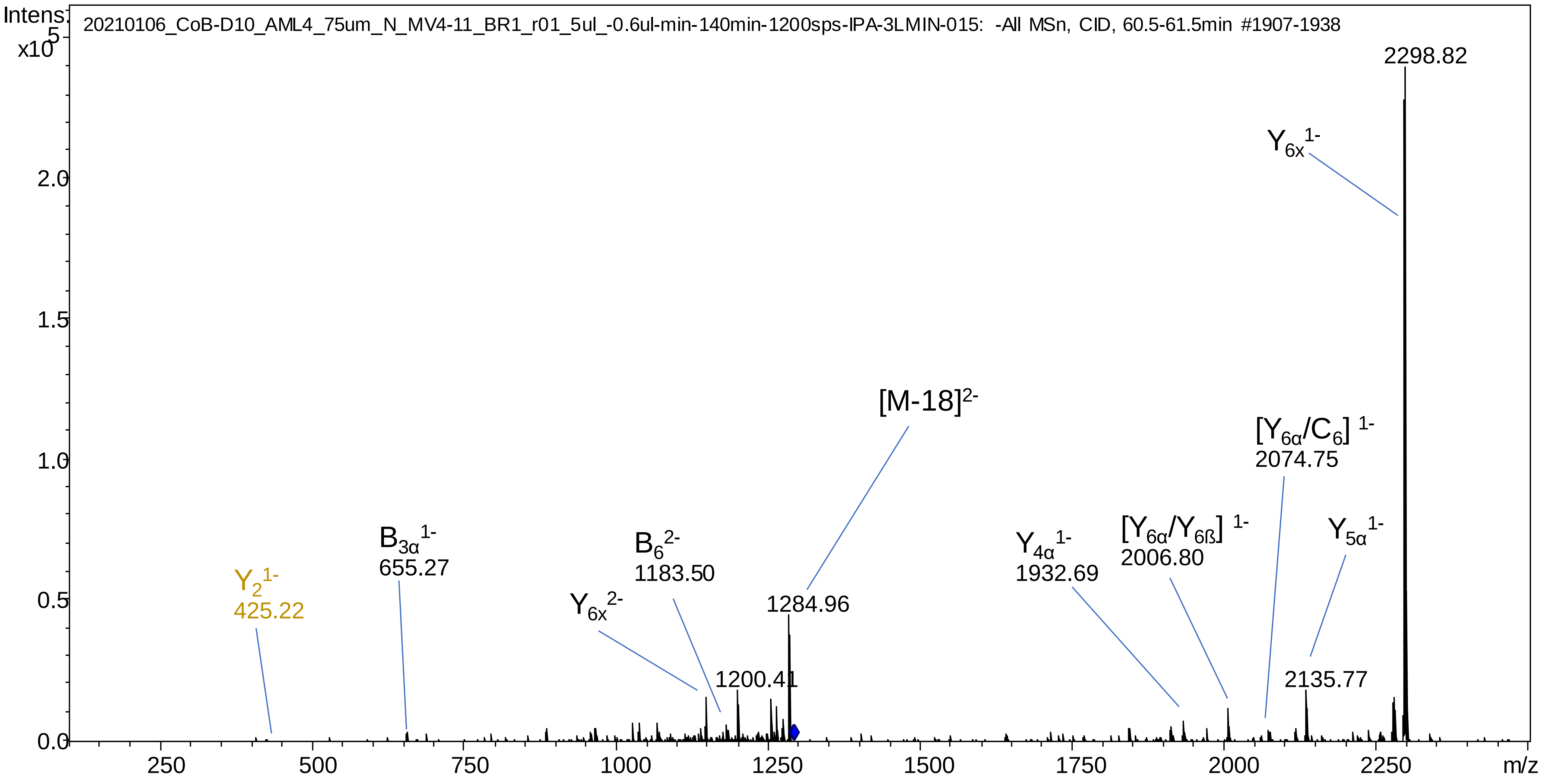

Glycan 27a

H6N5F1S2

Depicted MS² was obtained from analysis of cell line: Kasumi-1

**Monoisotopic mass:**	**2589.93 Da**
**Charge observed:**	**2-**
**Theoretical ion:**	***m/z* 1366.99**
**Observed ion:**	***m/z* 1366.98**
**Mass deviation:**	***m/z* 0.01**
**Retention time:**	**60.0 min**

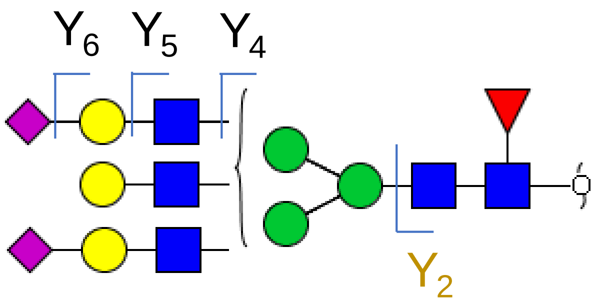

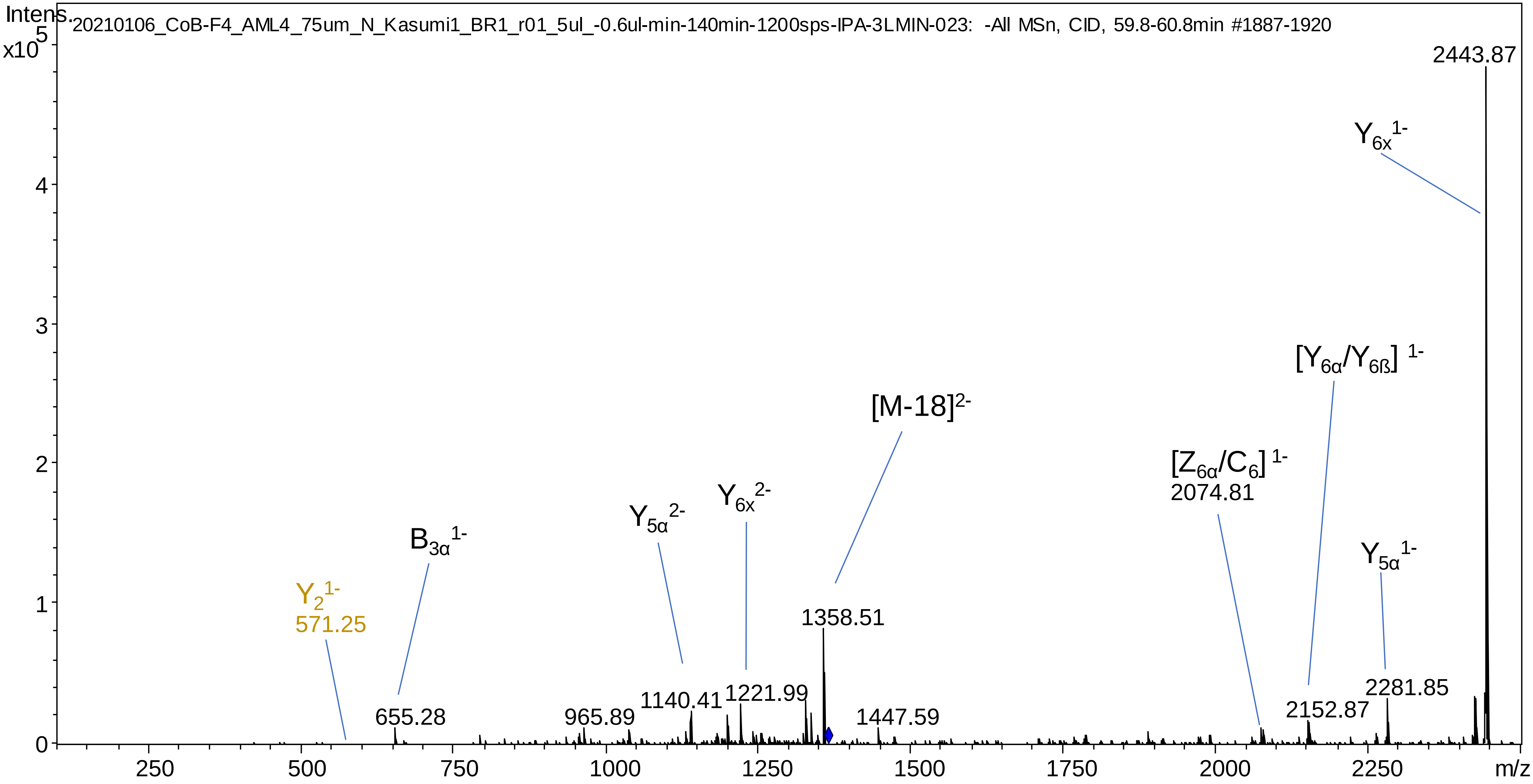

Glycan 27b

H6N5F1S2

Depicted MS² was obtained from analysis of cell line: OCI-AML-3

**Monoisotopic mass:**	**2589.93 Da**
**Charge observed:**	**2-**
**Theoretical ion:**	***m/z* 1366.99**
**Observed ion:**	***m/z* 1367.00**
**Mass deviation:**	***m/z* 0.01**
**Retention time:**	**63.4 min**

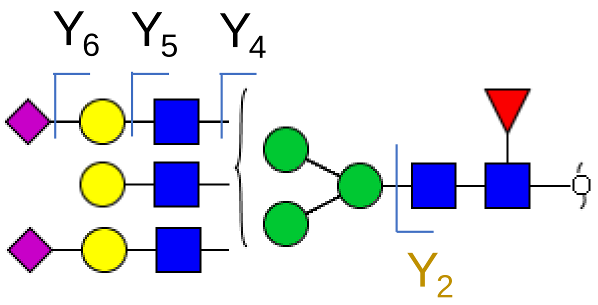

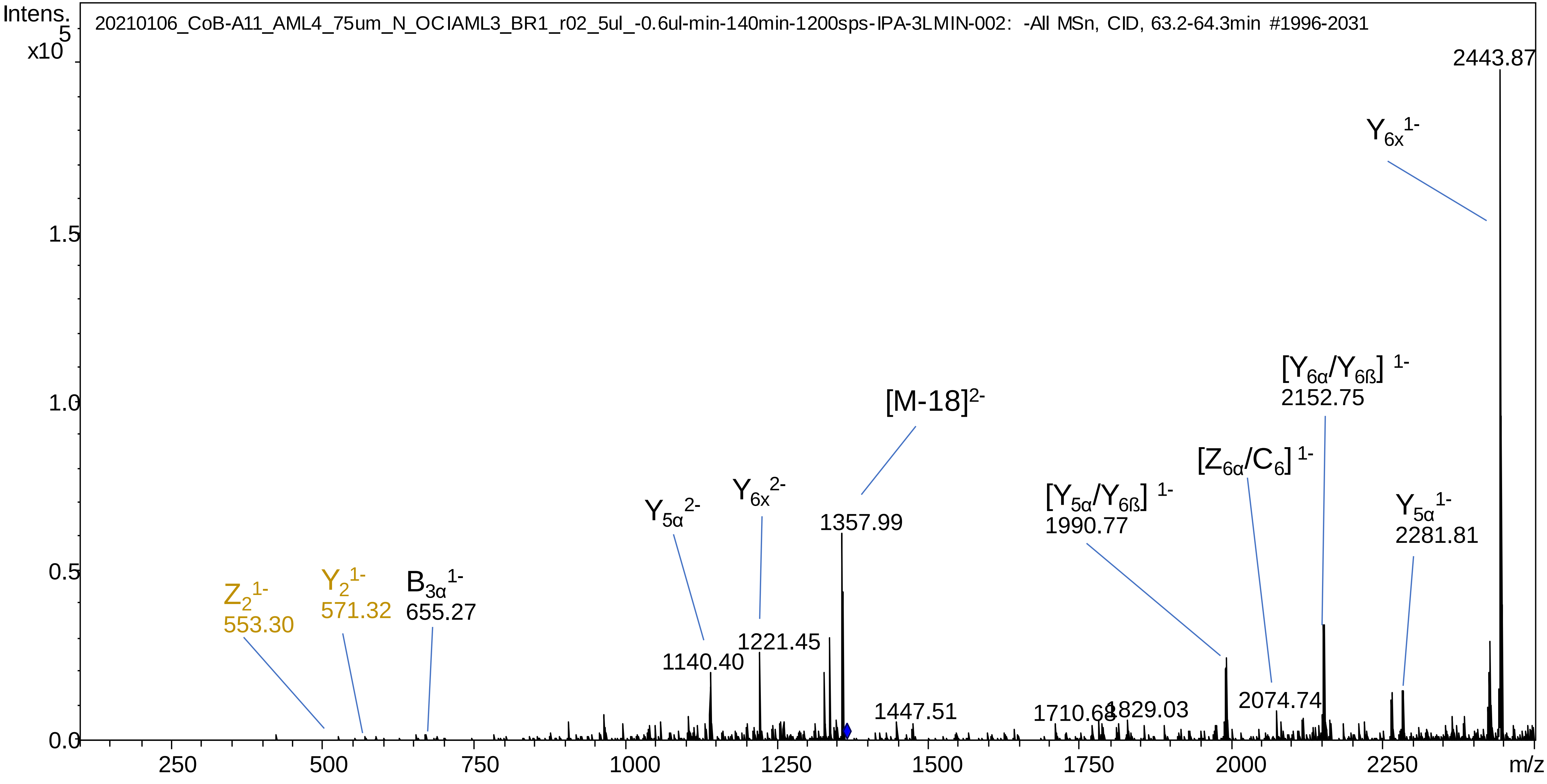

Glycan 27c

H6N5F1S2

Depicted MS² was obtained from analysis of cell line: M-07e

**Monoisotopic mass:**	**2735.99 Da**
**Charge observed:**	**2-**
**Theoretical ion:**	***m/z* 1366.99**
**Observed ion:**	***m/z* 1366.98**
**Mass deviation:**	***m/z* 0.02**
**Retention time:**	**66.5 min**

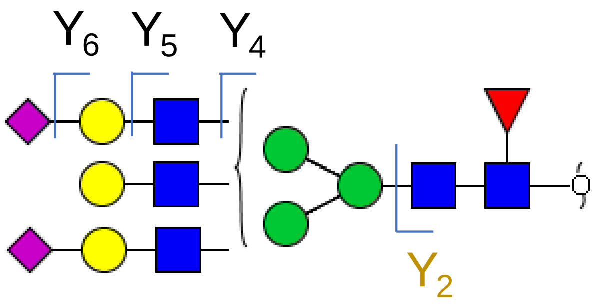

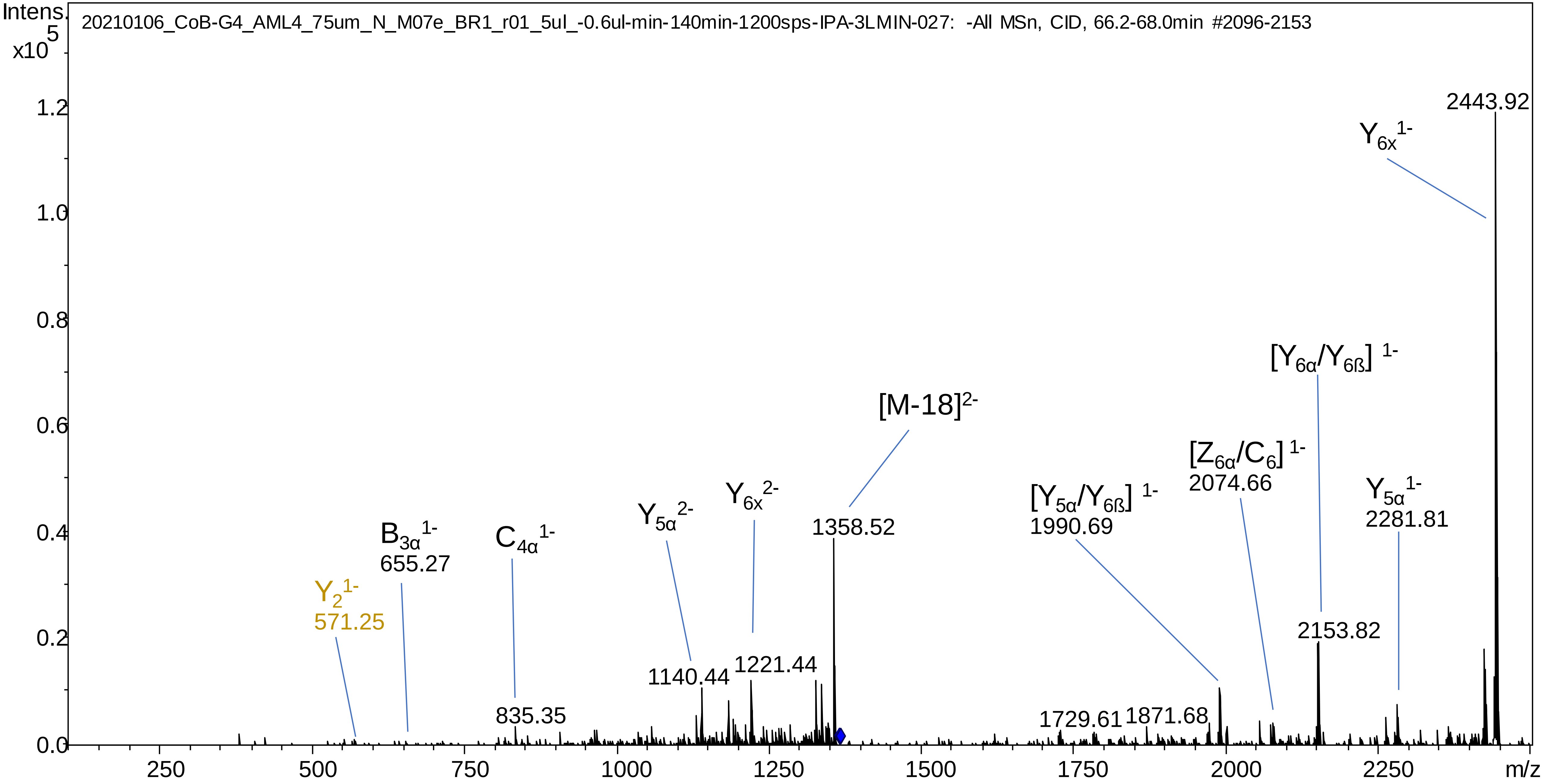

Glycan 28a

H6N5S3

Depicted MS[2] was obtained from analysis of cell line: MV4-11

**Monoisotopic mass:**	**2881.03 Da**
**Charge observed:**	**2-**
**Theoretical ion:**	***m/z* 1439.51**
**Observed ion:**	***m/z* 1439.51**
**Mass deviation:**	***m/z* 0.00**
**Retention time:**	**54.7 min**

Retention time fits to glycan isomer identifed in Fetuin standard (G. Palmisano et al. Rsc Adv, 2013, 3, 22706-22726).

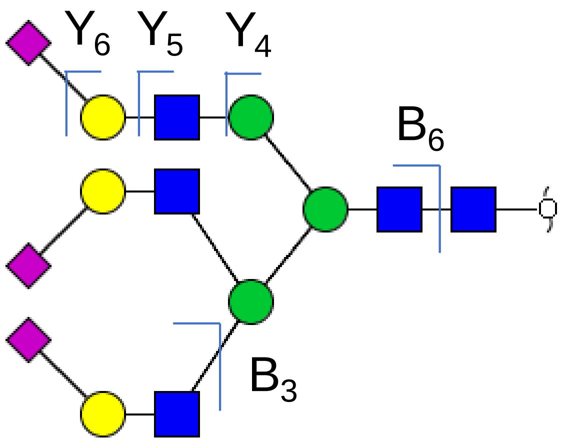

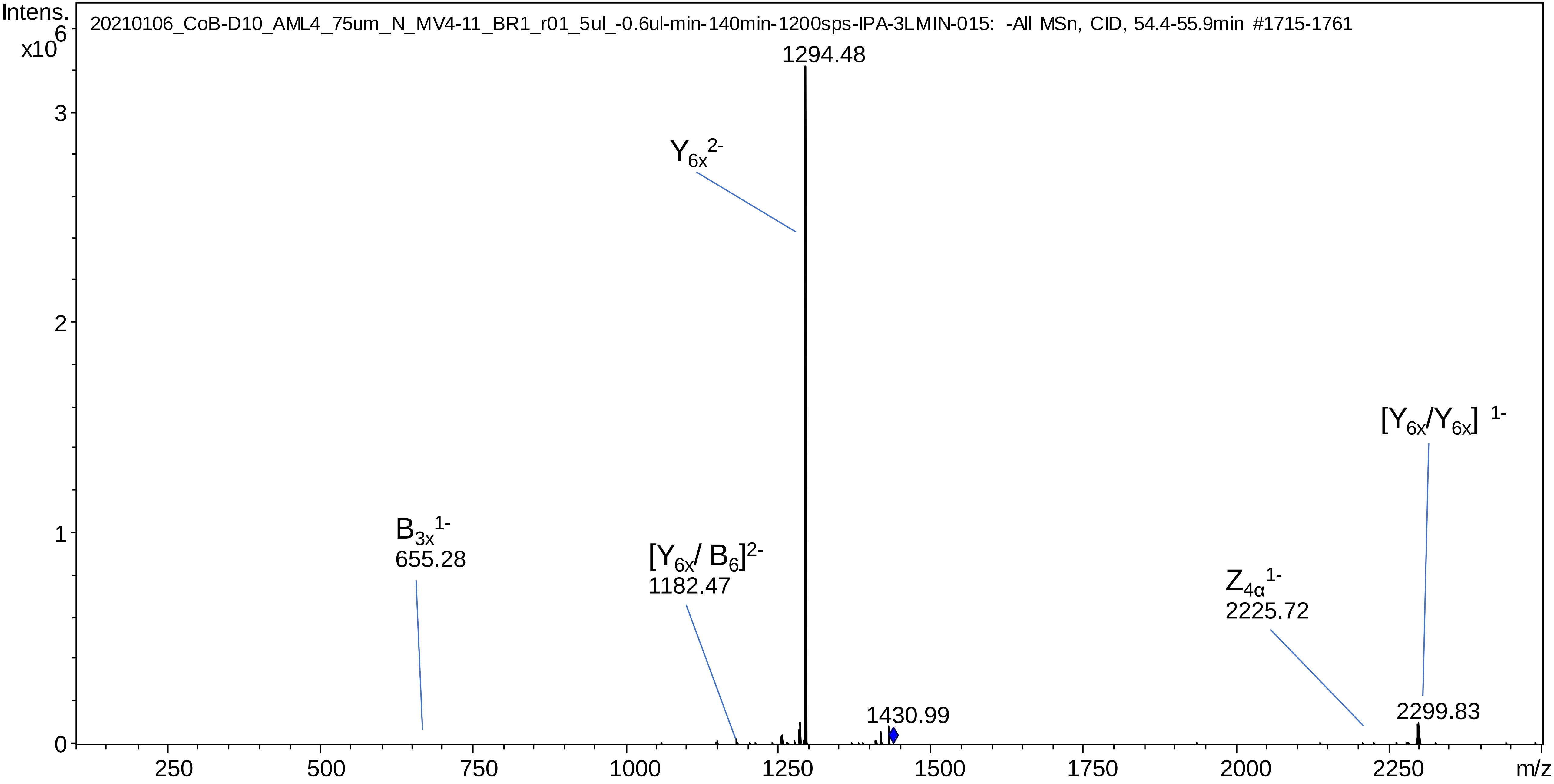

Glycan 28b

H6N5S3

Depicted MS[2] was obtained from analysis of cell line: MV4-11

**Monoisotopic mass:**	**2881.03 Da**
**Charge observed:**	**2-**
**Theoretical ion:**	***m/z* 1439.51**
**Observed ion:**	***m/z* 1439.51**
**Mass deviation:**	***m/z* 0.00**
**Retention time:**	**62.8 min**

Retention time fits to glycan isomer identifed in Fetuin standard (G. Palmisano et al. Rsc Adv, 2013, 3, 22706-22726).

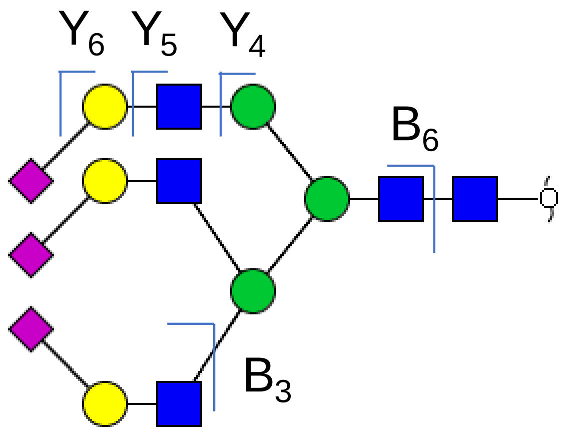

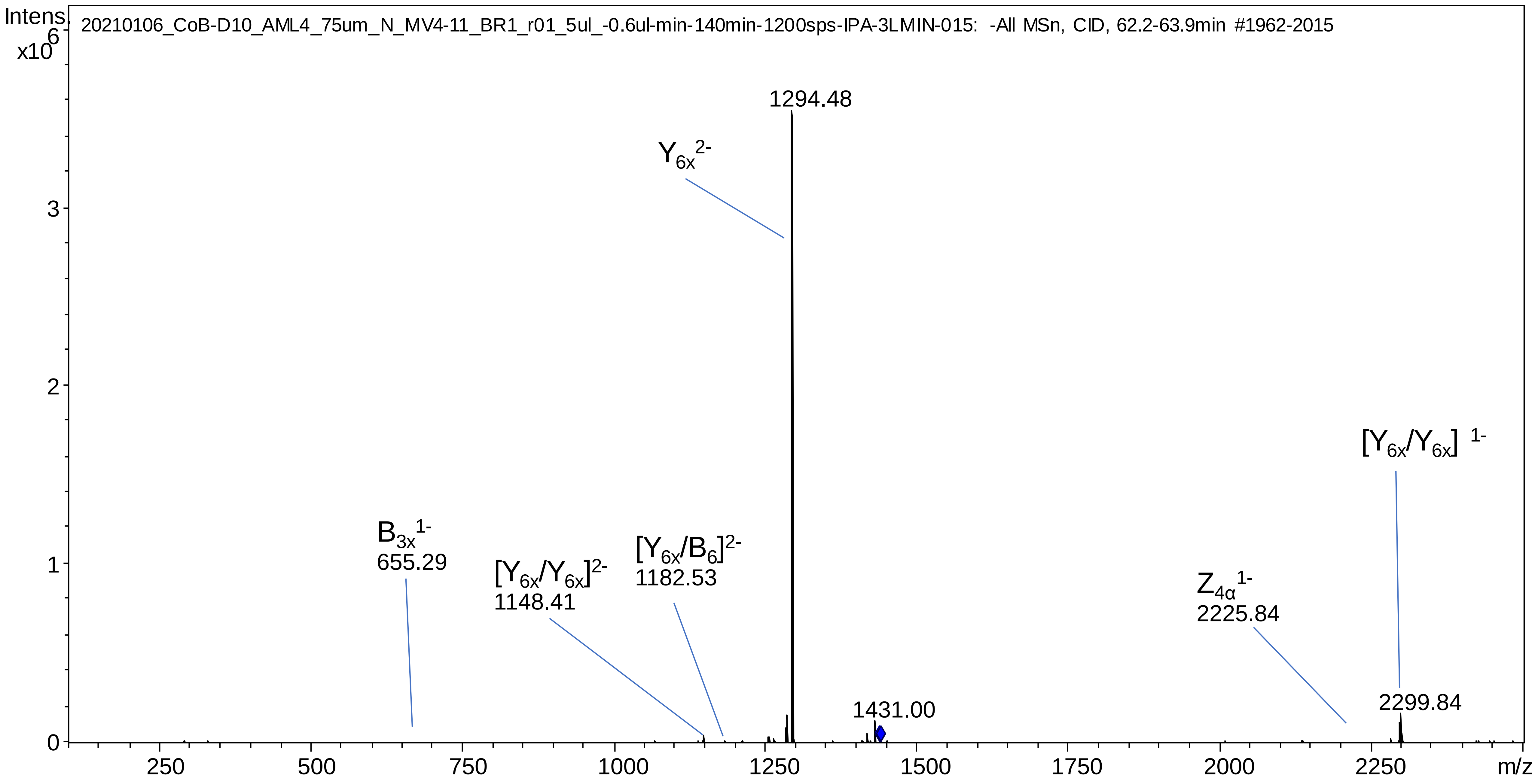

Glycan 29a

H6N6F1S2

Depicted MS² was obtained from analysis of cell line: TF-1

**Monoisotopic mass:**	**2939.07 Da**
**Charge observed:**	**2-**
**Theoretical ion:**	***m/z* 1468.53**
**Observed ion:**	***m/z* 1468.53**
**Mass deviation:**	***m/z* 0.00**
**Retention time:**	**46.6 min**

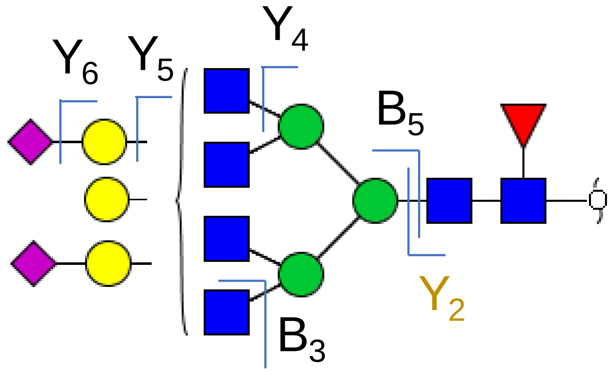

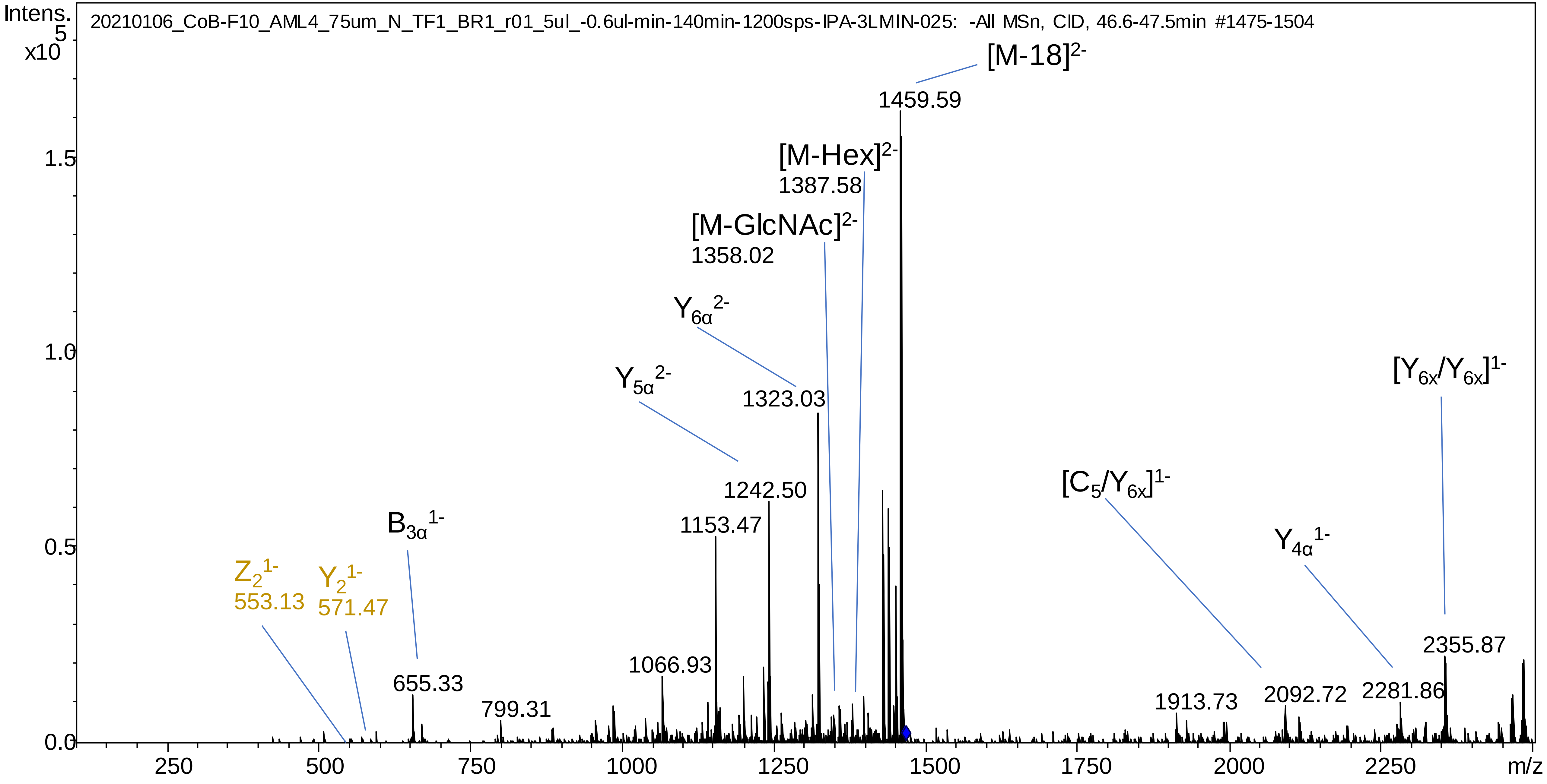

Glycan 29b

H6N6F1S2

Depicted MS² was obtained from analysis of cell line: TF-1

**Monoisotopic mass:**	**2939.07 Da**
**Charge observed:**	**2-**
**Theoretical ion:**	***m/z* 1468.53**
**Observed ion:**	***m/z* 1468.53**
**Mass deviation:**	***m/z* 0.00**
**Retention time:**	**51.5 min**

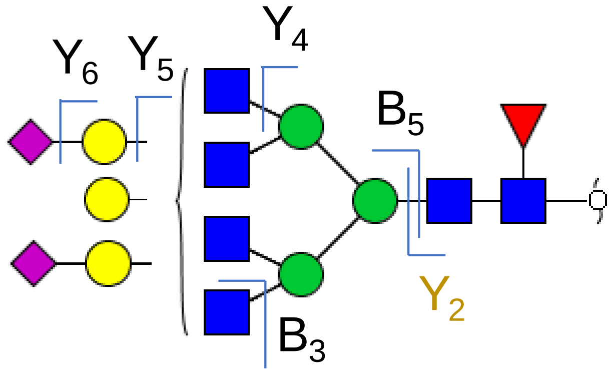

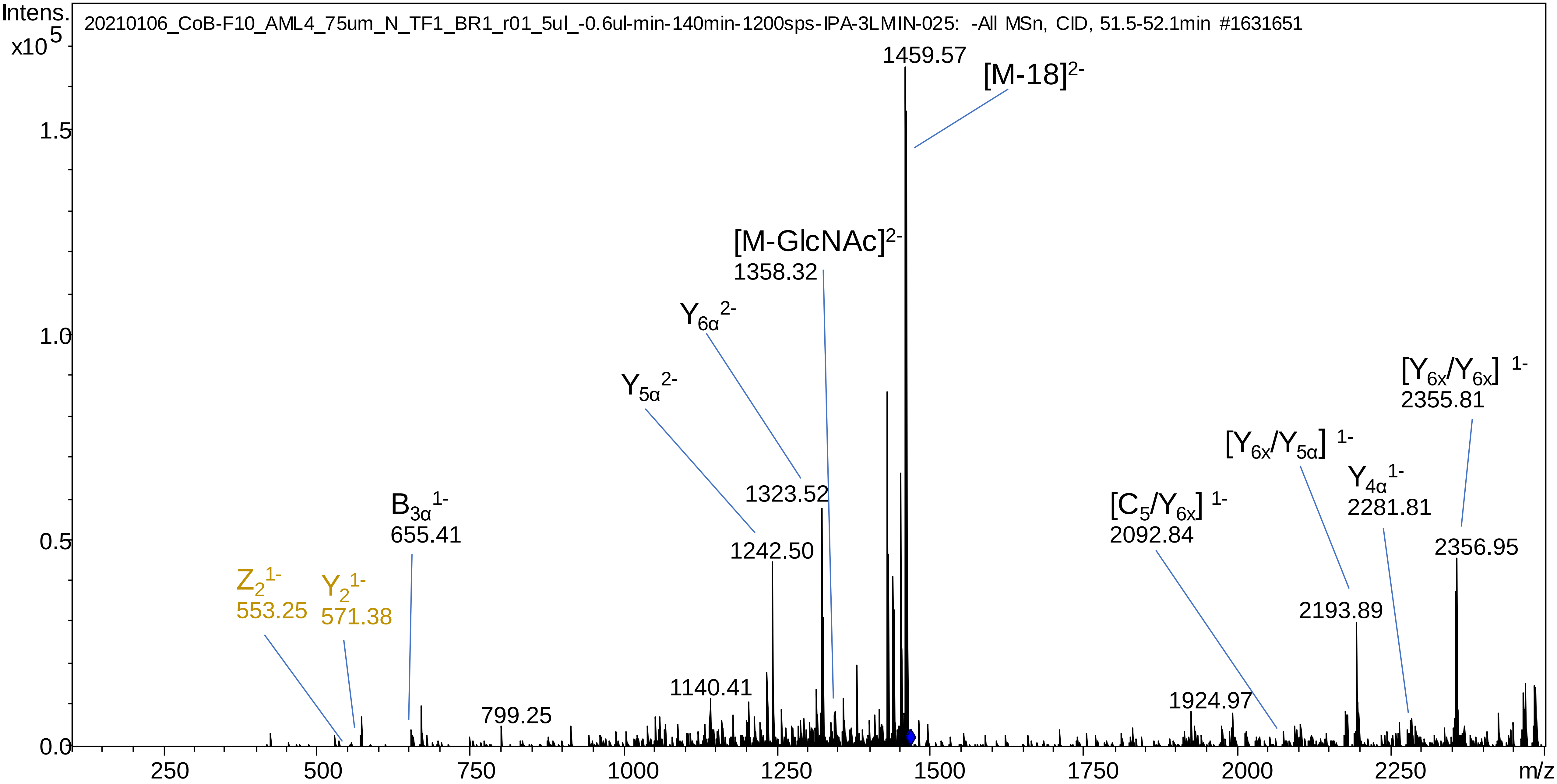

Glycan 30a

H6N5F1S3

Depicted MS² was obtained from analysis of cell line: TF-1

**Monoisotopic mass:**	**3027.08 Da**
**Charge observed:**	**2-**
**Theoretical ion:**	***m/z* 1512.54**
**Observed ion:**	***m/z* 1512.52**
**Mass deviation:**	***m/z* 0.02**
**Retention time:**	**66.5 min**

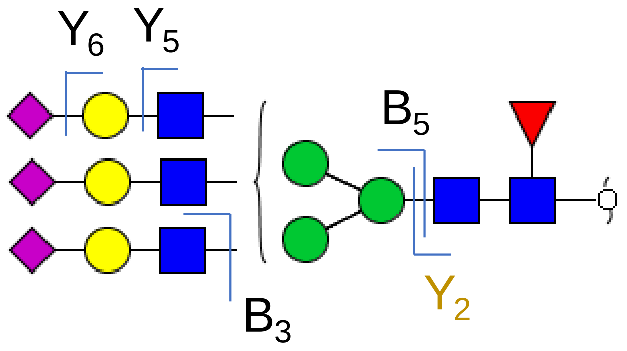

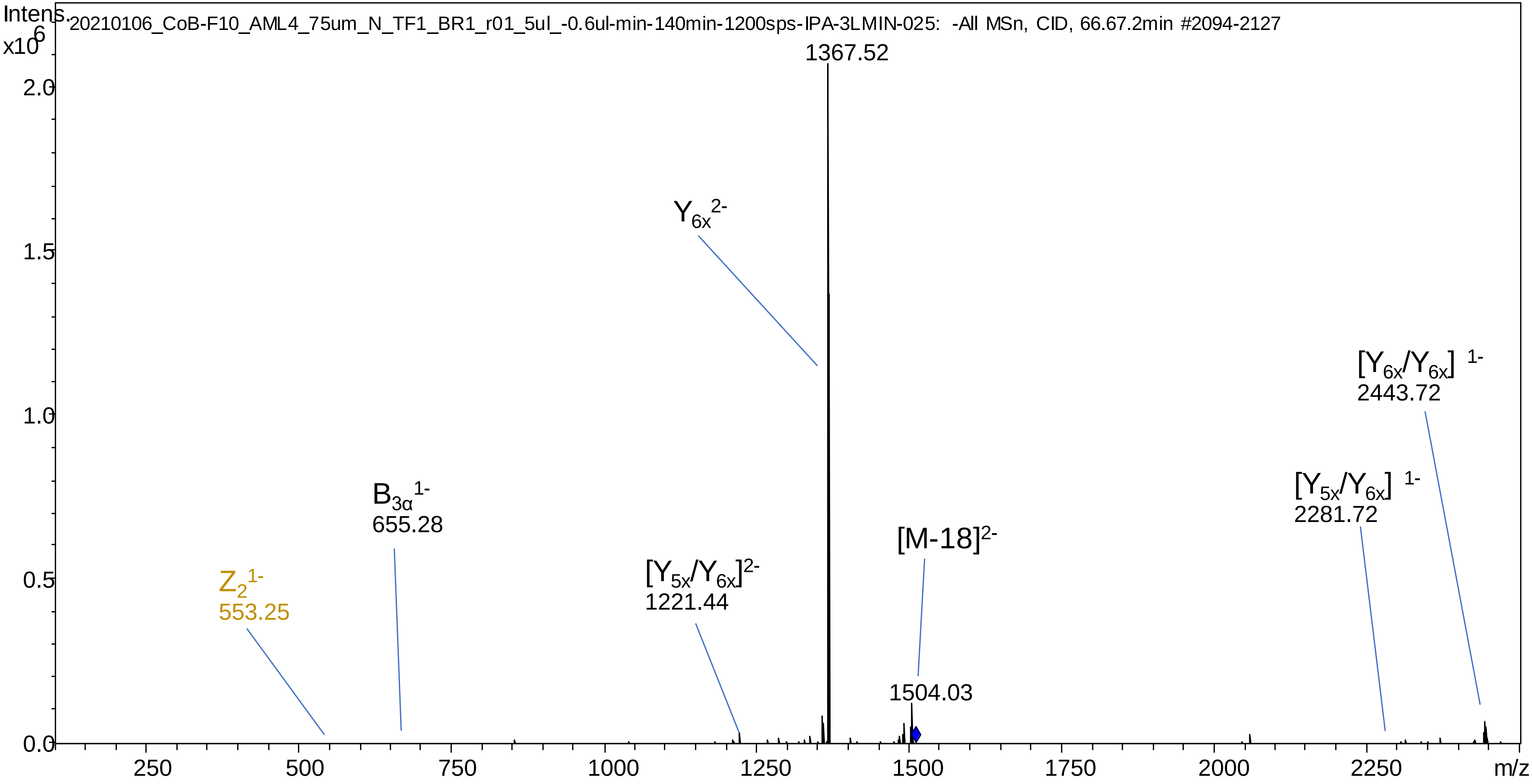

Glycan 30b

H6N5F1S3

Depicted MS² was obtained from analysis of cell line: KG-1a

**Monoisotopic mass:**	**3027.08 Da**
**Charge observed:**	**2-**
**Theoretical ion:**	***m/z* 1512.54**
**Observed ion:**	***m/z* 1512.53**
**Mass deviation:**	***m/z* 0.01**
**Retention time:**	**68.1 min**

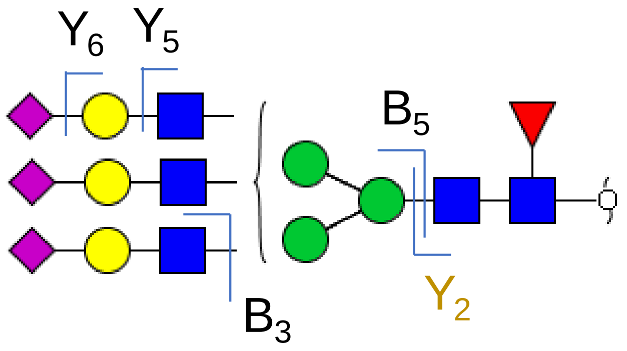

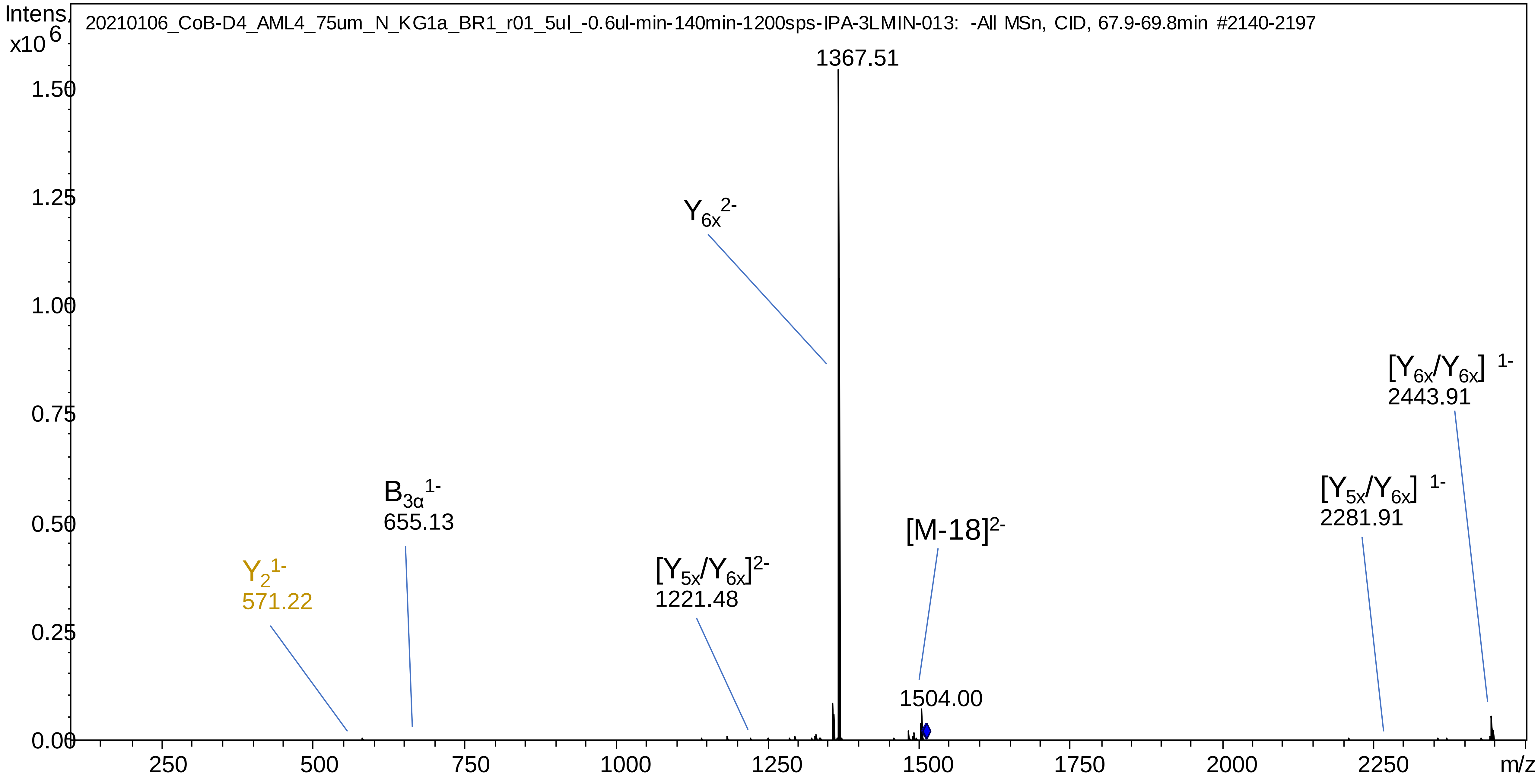

Glycan 31a

H6N5S4

Depicted MS[2] was obtained from analysis of cell line: MV4-11

**Monoisotopic mass:**	**3172.12 Da**
**Charge observed:**	**2-**
**Theoretical ion:**	***m/z* 1585.05**
**Observed ion:**	***m/z* 1585.05**
**Mass deviation:**	***m/z* 0.00**
**Retention time:**	**53.4 min**

Retention time fits to glycan isomer identifed in Fetuin standard (G. Palmisano et al. Rsc Adv, 2013, 3, 22706-22726).

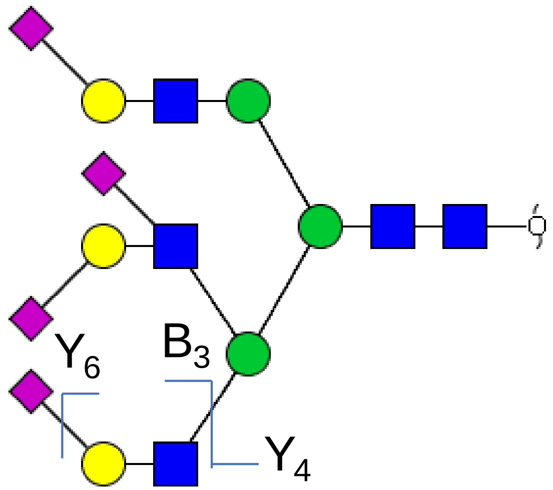

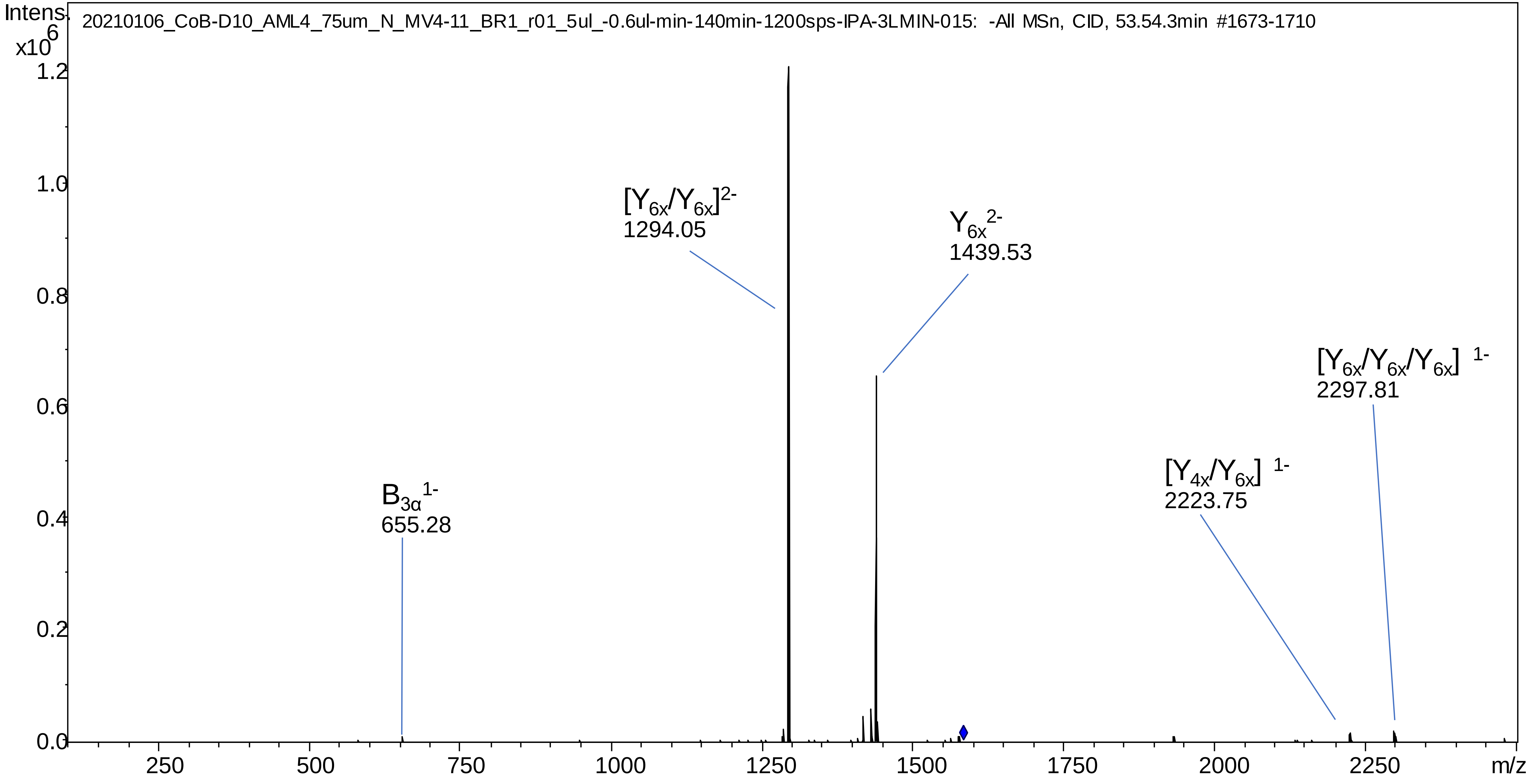

Glycan 31b

H6N5S4

Depicted MS² was obtained from analysis of cell line: MV4-11

**Monoisotopic mass:**	**3172.12 Da**
**Charge observed:**	**2-**
**Theoretical ion:**	***m/z* 1585.05**
**Observed ion:**	***m/z* 1585.04**
**Mass deviation:**	***m/z* 0.01**
**Retention time:**	**62.0 min**

Retention time fits to glycan isomer identifed in Fetuin standard (G. Palmisano et al. Rsc Adv, 2013, 3, 22706-22726).

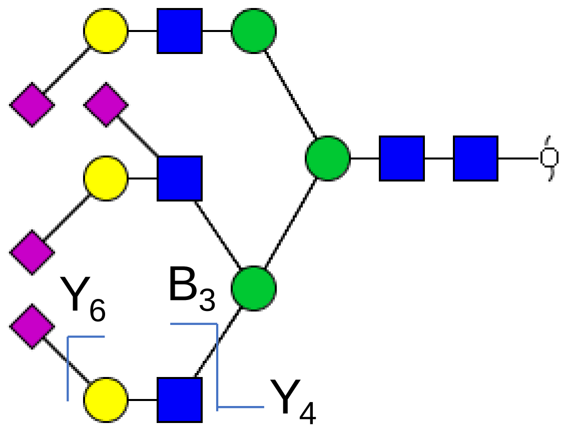

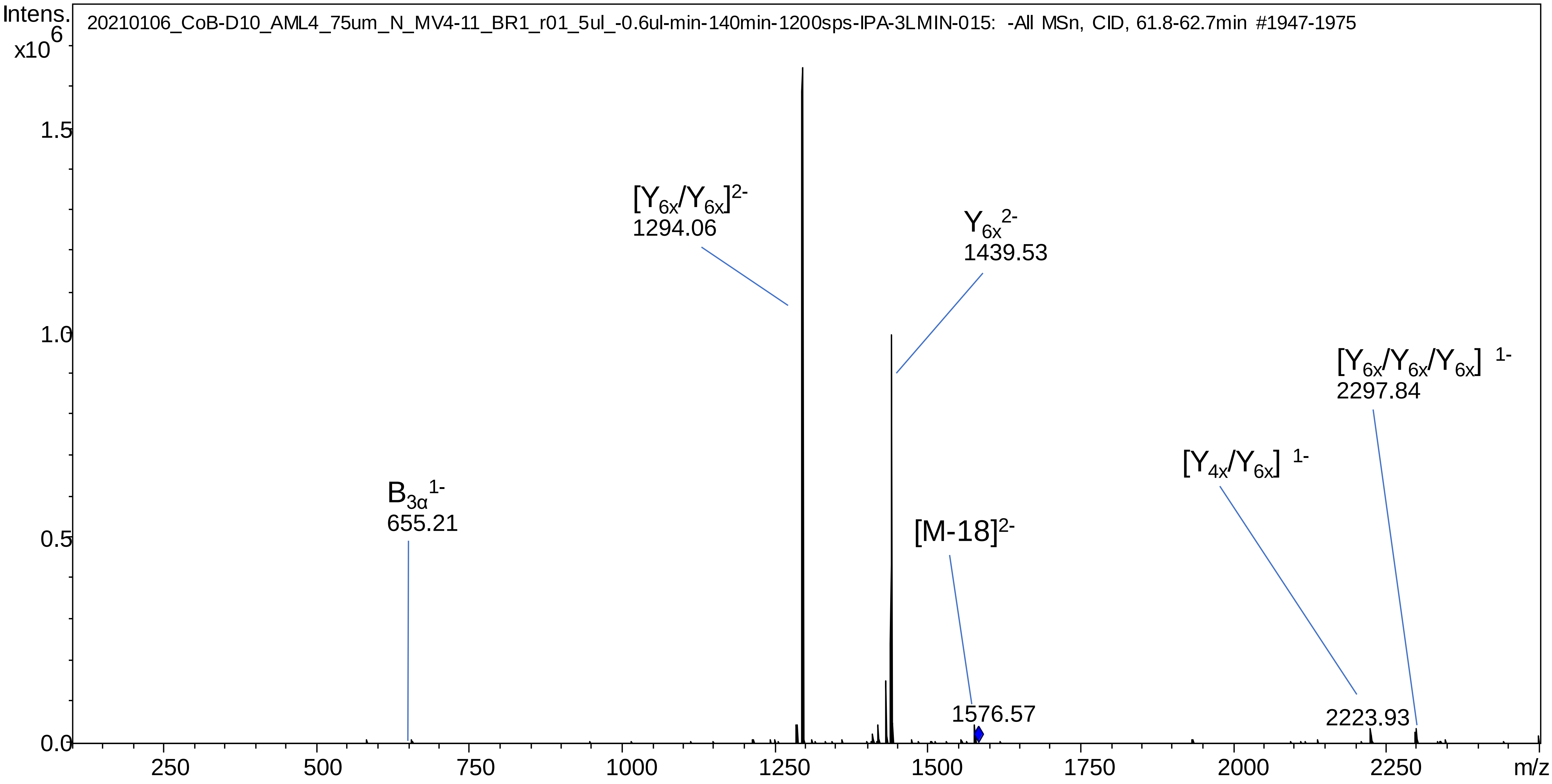

Glycan 32

H6N6F1S3

Depicted MS[2] was obtained from analysis of cell line: TF-1

**Monoisotopic mass:**	**3230.17 Da**
**Charge observed:**	**2-**
**Theoretical ion:**	***m/z* 1614.08**
**Observed ion:**	***m/z* 1614.06**
**Mass deviation:**	***m/z* 0.02**
**Retention time:**	**51.1 min**

UniCarb-DB: #

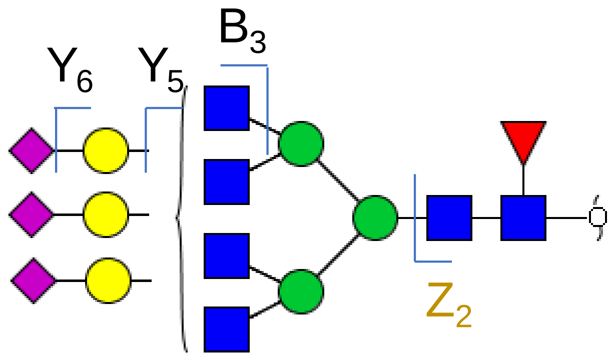

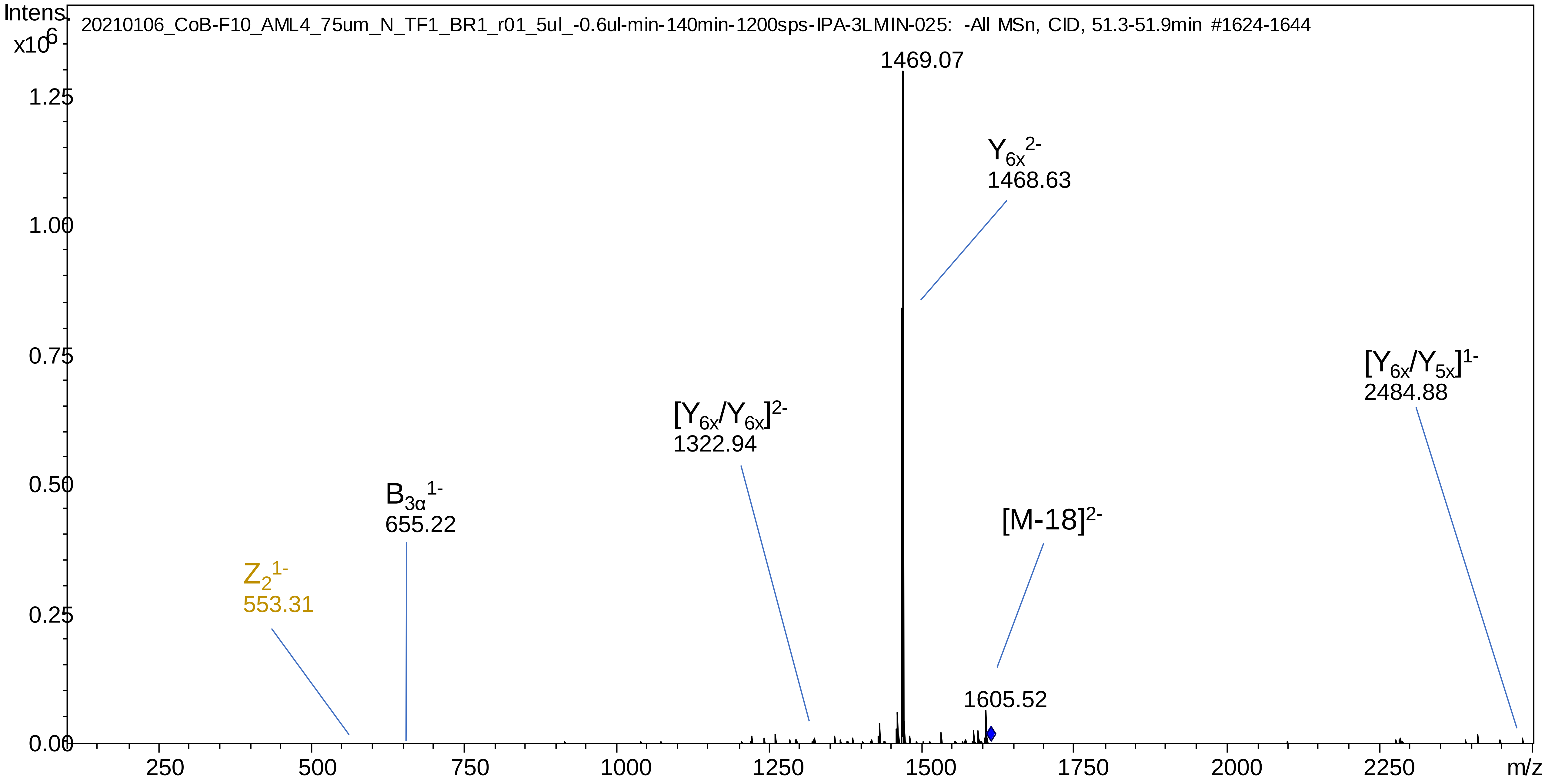

Glycan 33a

H7N6F1S4

Depicted MS^2 was obtained from analysis of cell line: Kasumi-1

**Monoisotopic mass:**	**3683.31 Da**
**Charge observed:**	**3-**
**Theoretical ion:**	***m/z* 1226.76**
**Observed ion:**	***m/z* 1226.76**
**Mass deviation:**	***m/z* 0.00**
**Retention time:**	**67.8 min**

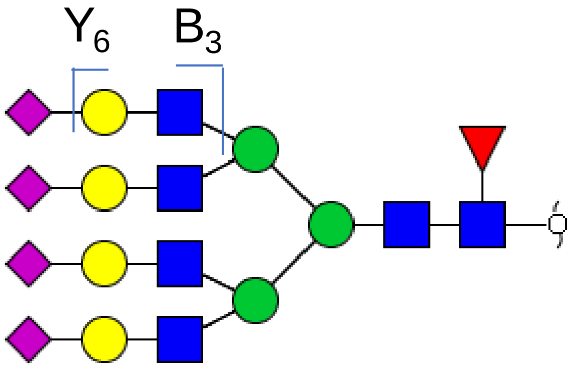

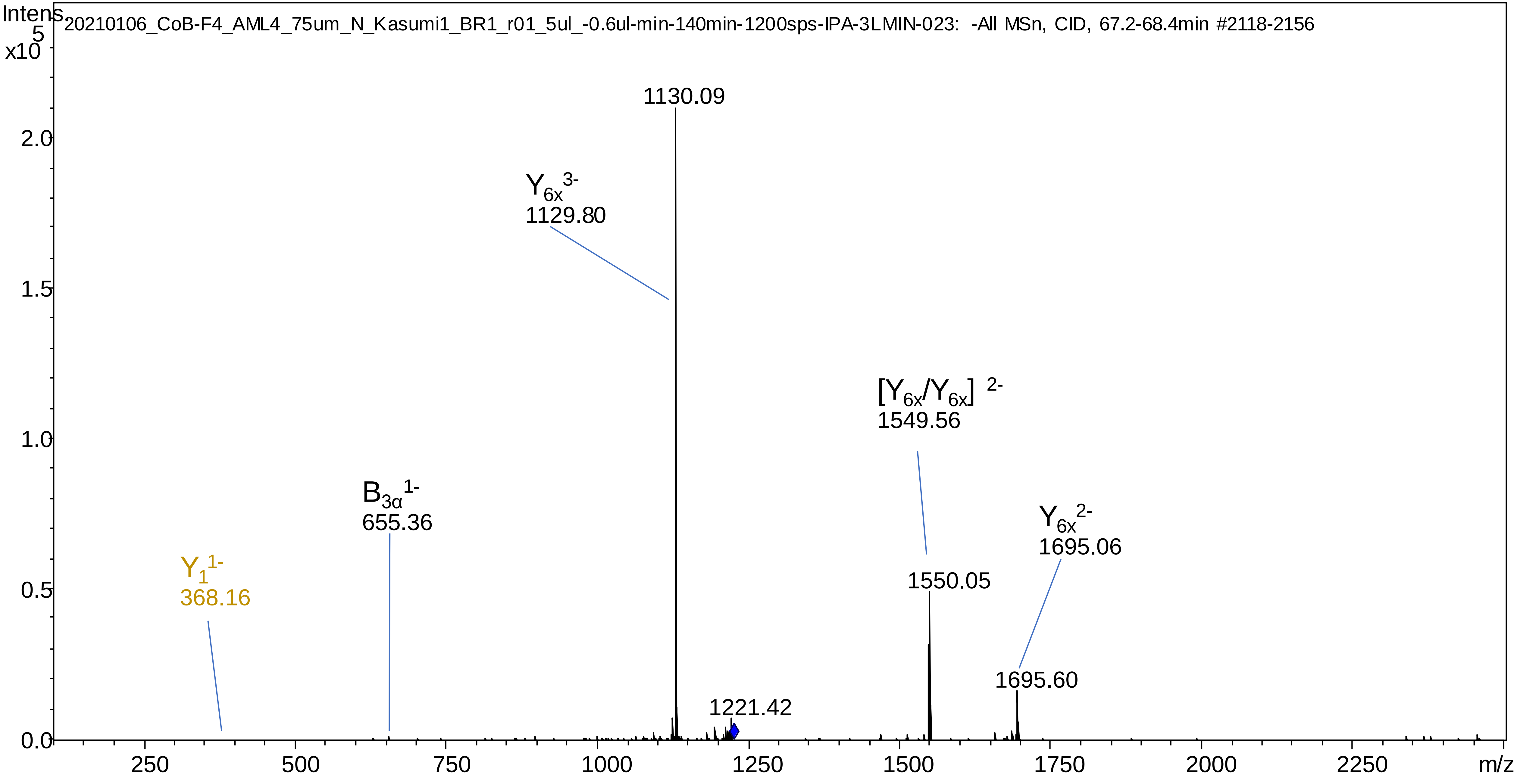

Glycan 33b

H7N6F1S4

Depicted MS^2 was obtained from analysis of cell line: TF-1

**Monoisotopic mass:**	**3683.31 Da**
**Charge observed:**	**3-**
**Theoretical ion:**	***m/z* 1226.76**
**Observed ion:**	***m/z* 1226.76**
**Mass deviation:**	***m/z* 0.00**
**Retention time:**	**71.0 min**

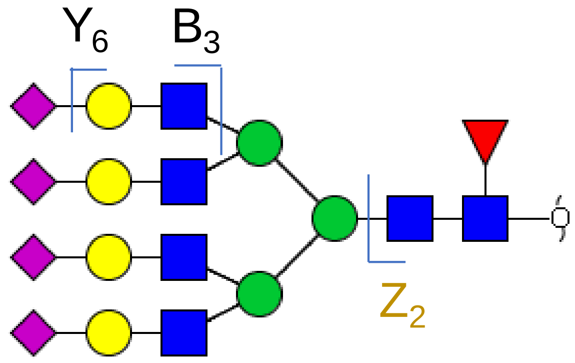

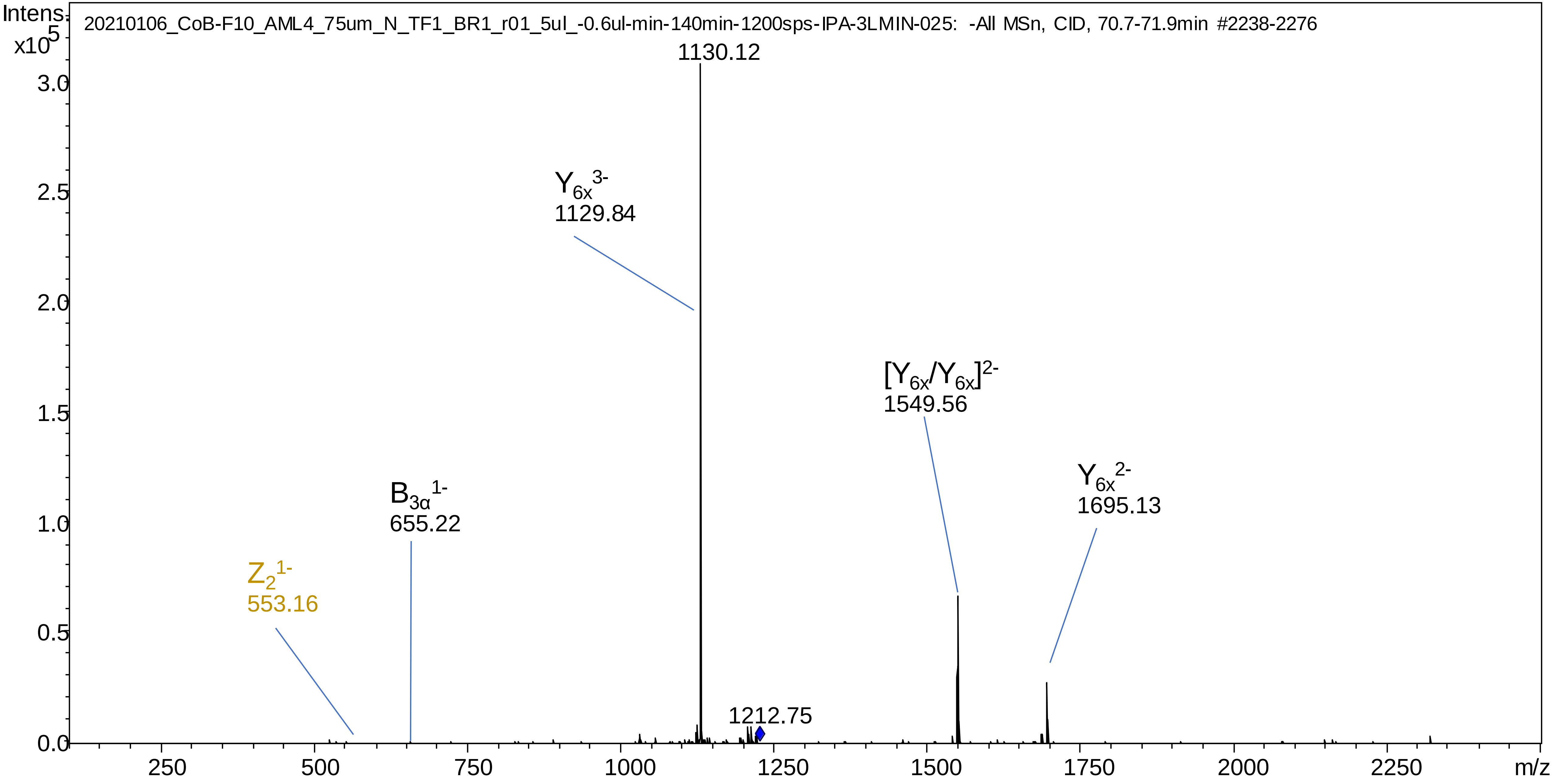

Glycan 34a

H5N5F1S1

Depicted MS^2 was obtained from analysis of cell line: HL-60

Monoisotopic mass: 2282.85 Da
Charge observed: 2-
Theoretical ion: *m/z* 1140.41
Observed ion: *m/z* 1140.42
Mass deviation: *m/z* 0.01
Retention time: 42.2 min
Note: α-2,6 sialic acid linkage confirmed by neuraminidase S and A treatment

UniCarb-DB: #2395

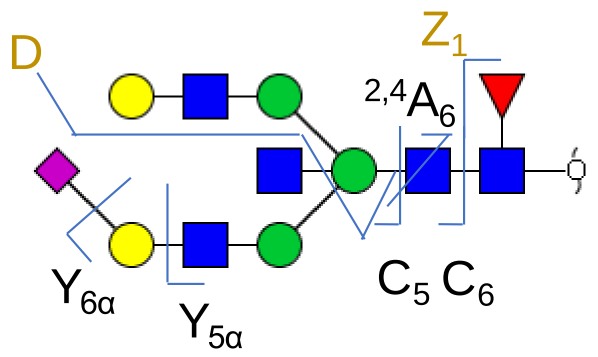

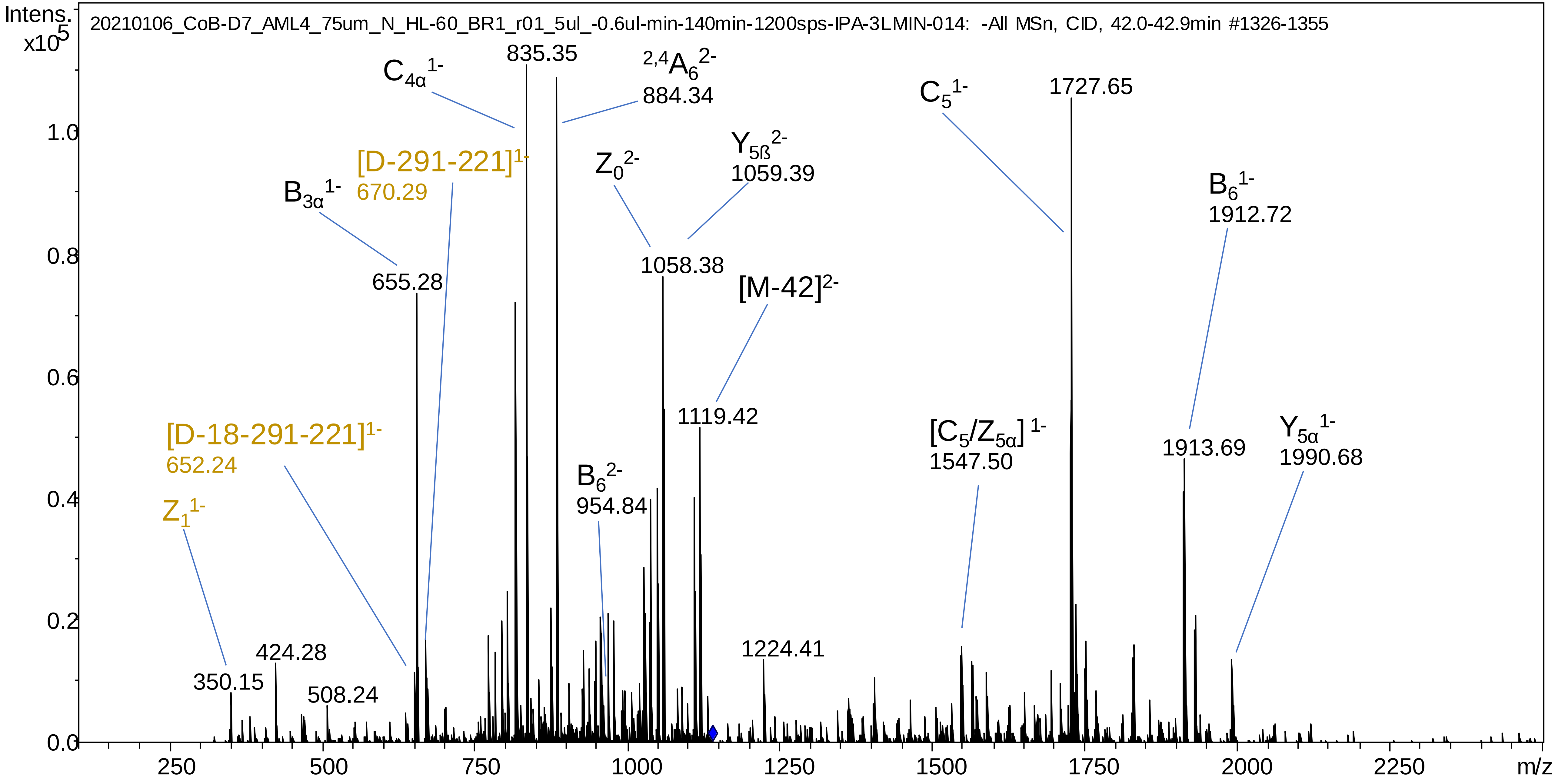

Glycan 34b

H5N5F1S1

Depicted MS[2] was obtained from analysis of cell line: HL-60

Monoisotopic mass: 2282.85 Da
Charge observed: 2-
Theoretical ion: *m/z* 1140.41
Observed ion: *m/z* 1140.40
Mass deviation: *m/z* 0.01
Retention time: 48.1 min
Note: α-2,3 sialic acid linkage confirmed by neuraminidase S and A treatment

UniCarb-DB: #2395

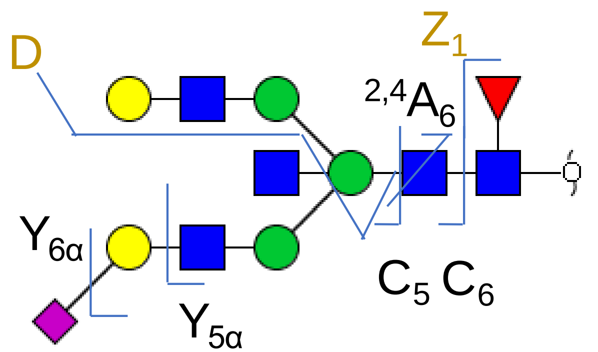

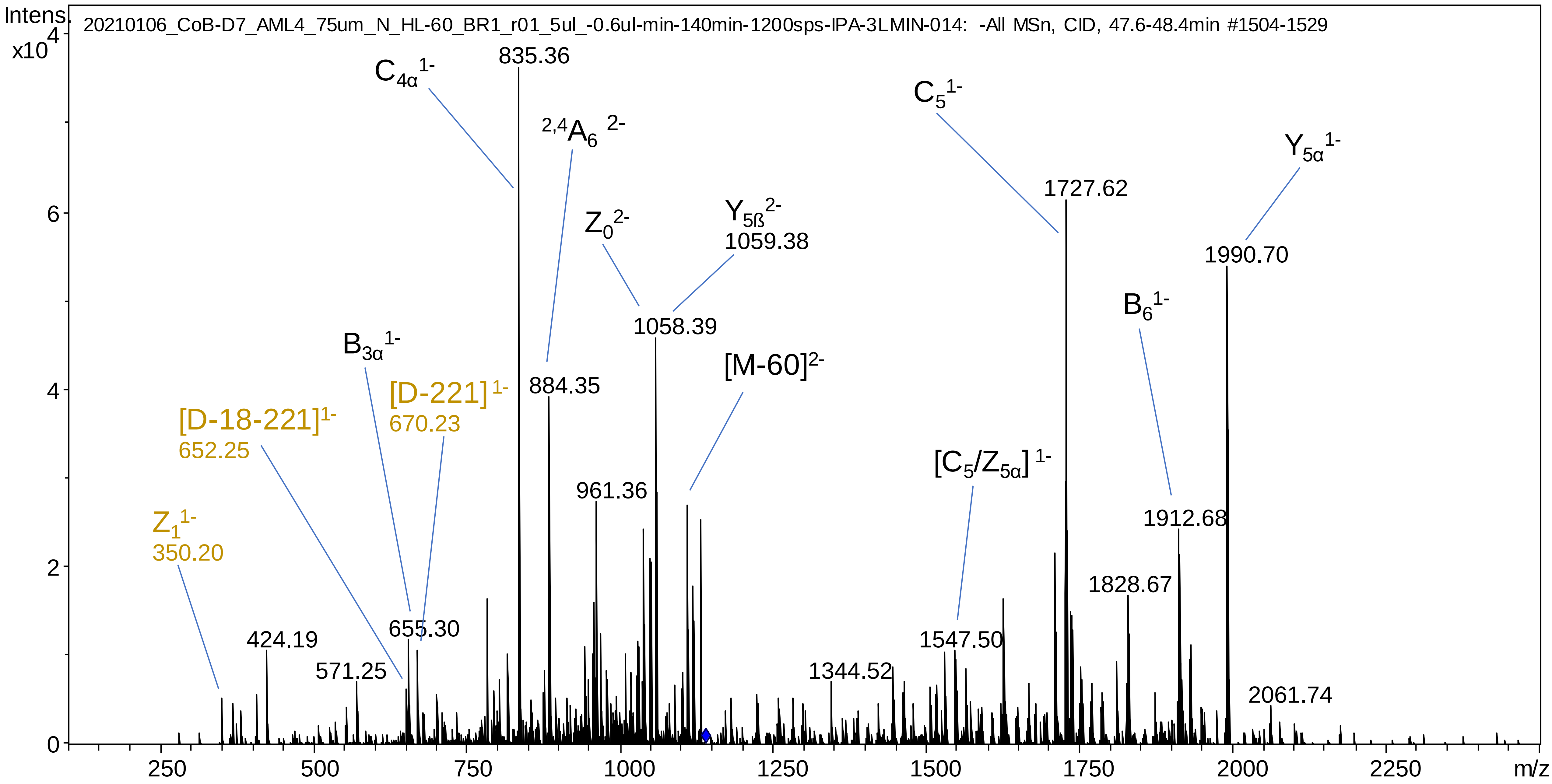

Glycan 35a

H5N5F1S2

Depicted MS² was obtained from analysis of cell line: TF-1

Monoisotopic mass: 2573.94 Da
Charge observed: 2-
Theoretical ion: *m/z* 1285.96
Observed ion: *m/z* 1285.96
Mass deviation: *m/z* 0.00
Retention time: 42.4 min
Note: α-2,6 sialic acid linkage confirmed by neuraminidase S and A treatment

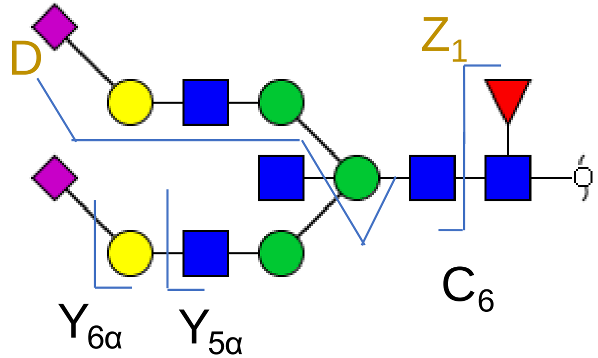

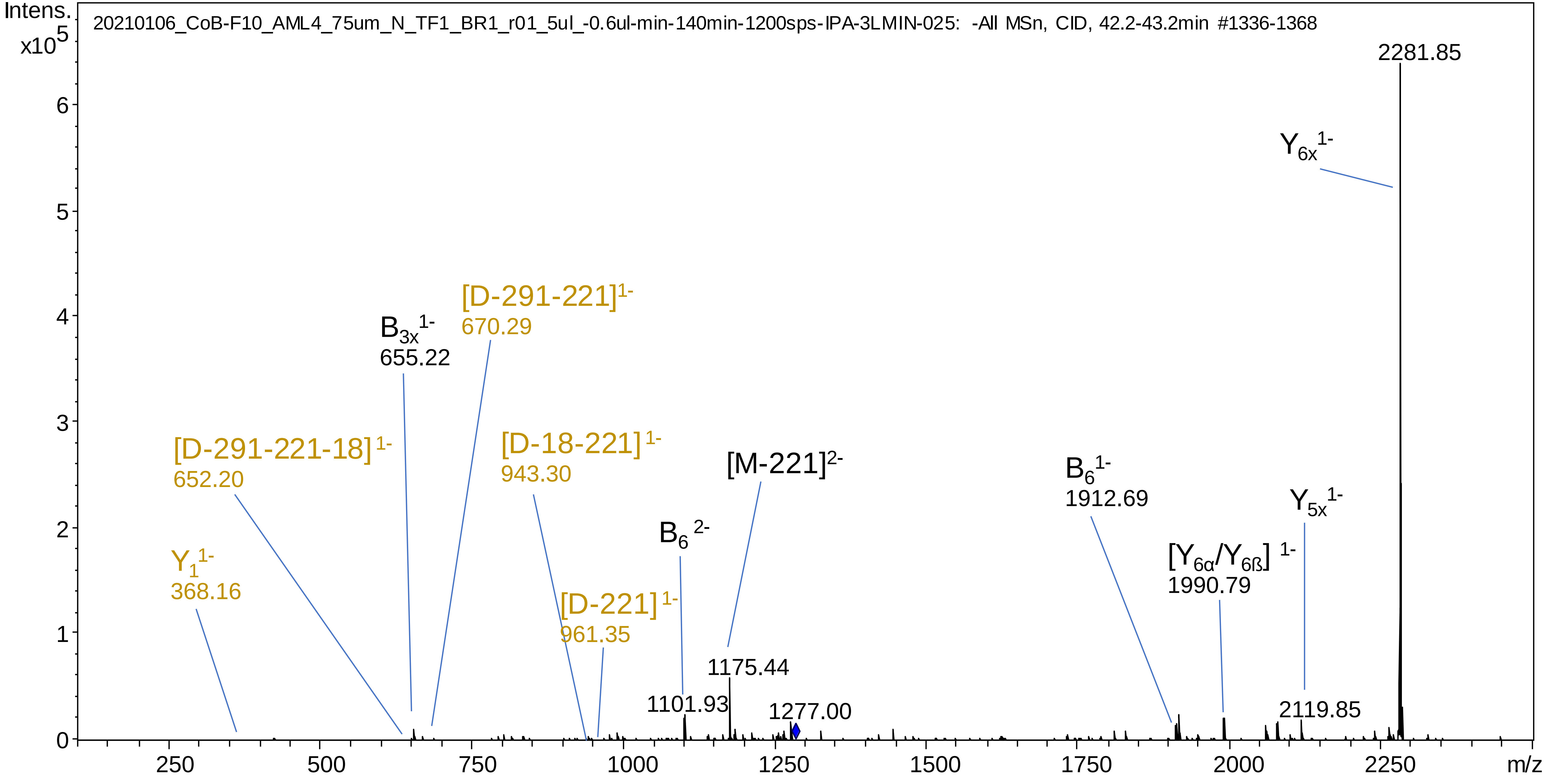

Glycan 35b

H5N5F1S2

Depicted MS² was obtained from analysis of cell line: TF-1

**Monoisotopic mass:**	**2573.94 Da**
**Charge observed:**	**2-**
**Theoretical ion:**	***m/z* 1285.96**
**Observed ion:**	***m/z* 1285.95**
**Mass deviation:**	***m/z* 0.01**
**Retention time:**	**48.4 min**

Note: Sialic acid linkages confirmed by neuraminidase S and A treatment

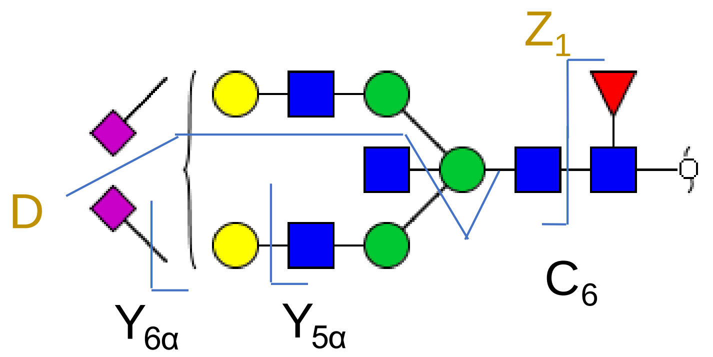

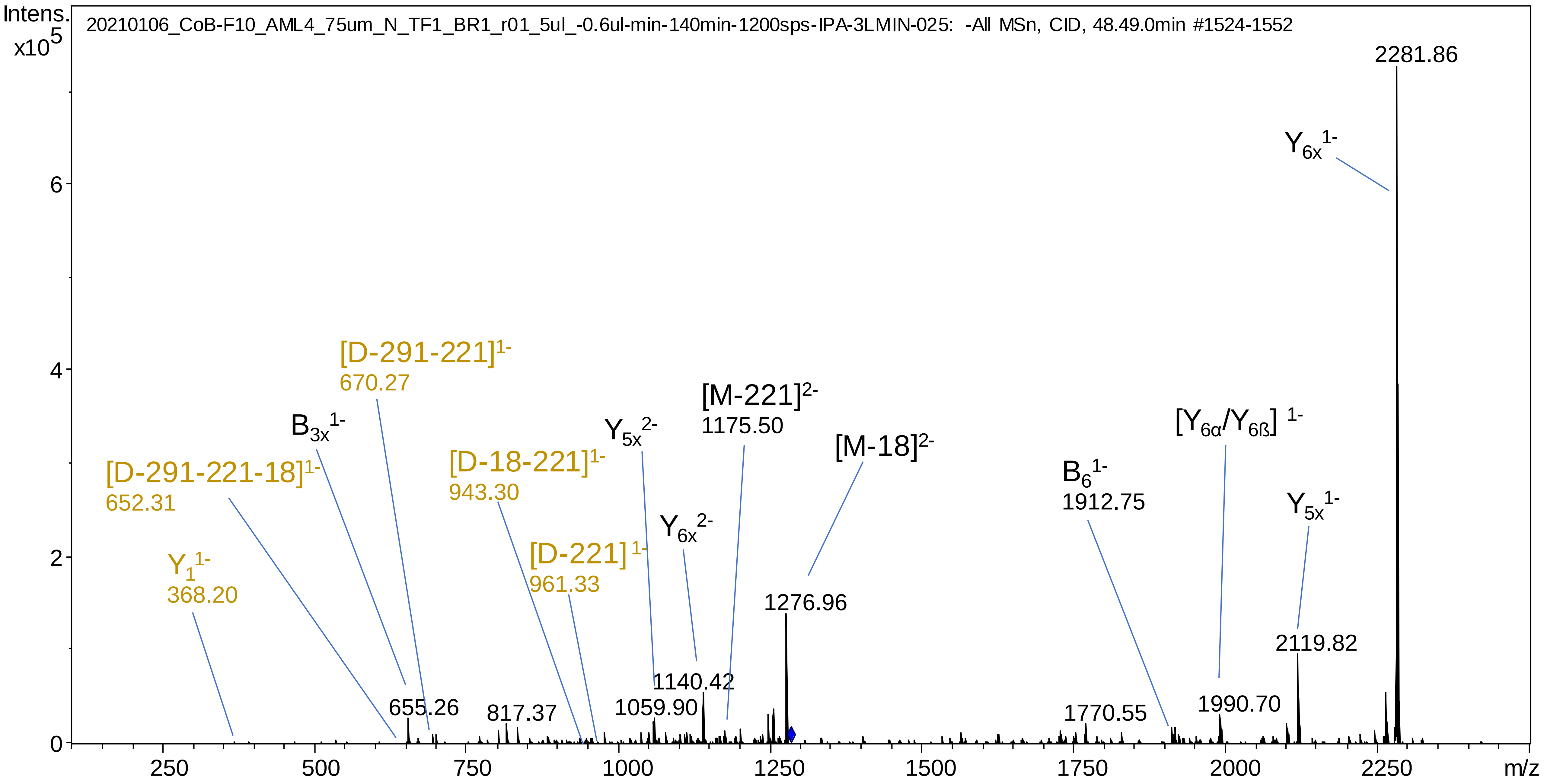

Glycan 35c

H5N5F1S2

Depicted MS^2 was obtained from analysis of cell line: HL-60

Monoisotopic mass: 2573.94 Da
Charge observed: 2-
Theoretical ion: *m/z* 1285.96
Observed ion: *m/z* 1285.96
Mass deviation: *m/z* 0.00
Retention time: 56.1 min
Note: Sialic acid linkages confirmed by neuraminidase S and A treatment

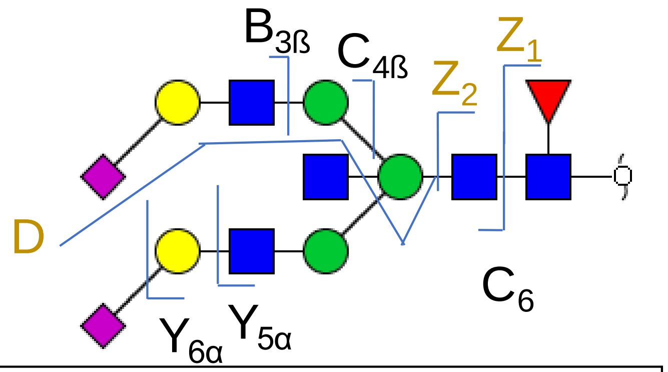

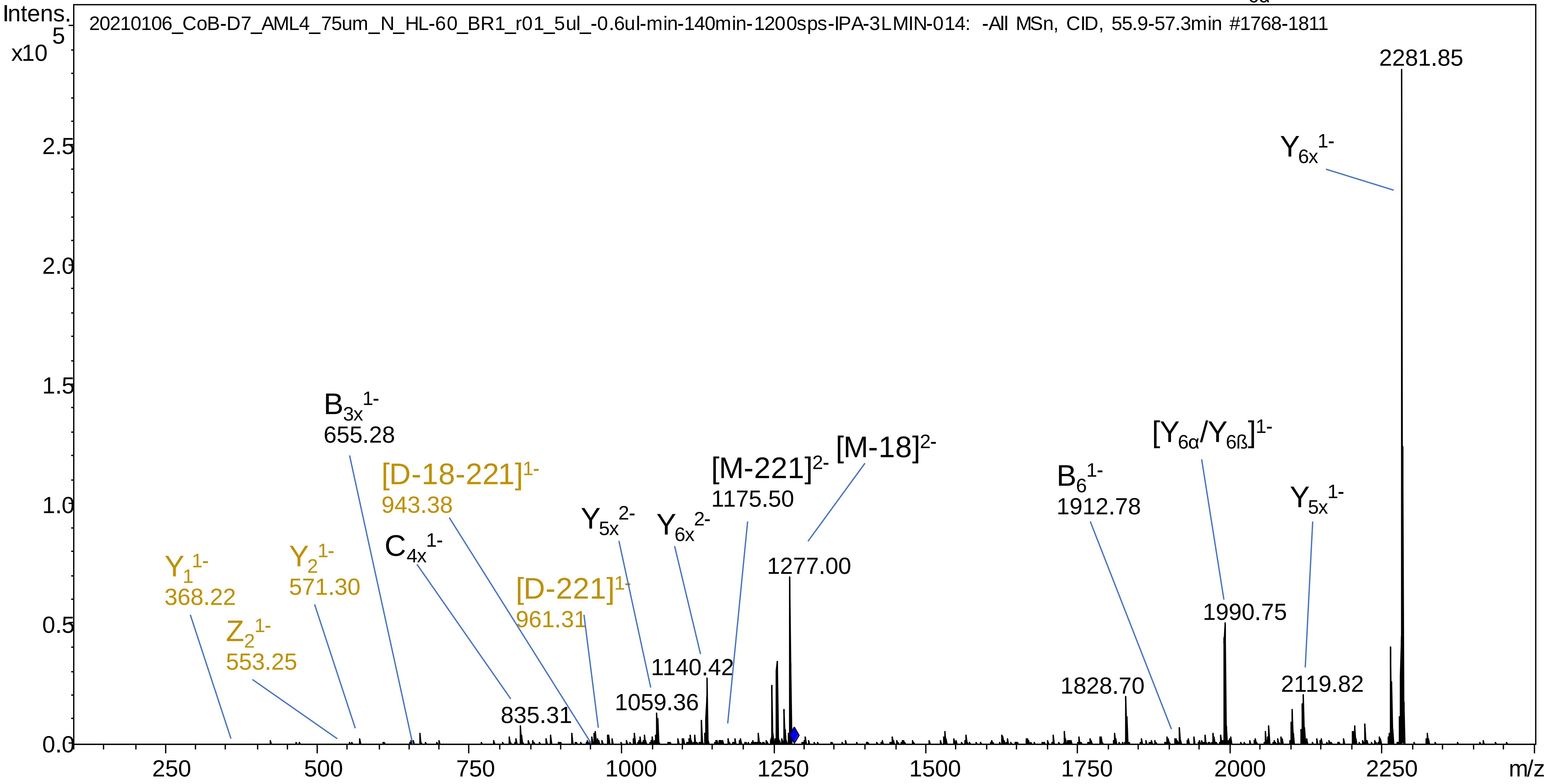

Glycan 36a

H3N3F1

Depicted MS² was obtained from analysis of cell line: TF-1

Monoisotopic mass: **1261.48 Da**
Charge observed: **1-**
Theoretical ion: ***m/z* 1260.47**
Observed ion: ***m/z* 1260.48**
Mass deviation: ***m/z* 0.01**
Retention time: **46.2 min**
Note: No D-ion at *m/z* 526 which would indicate that the GlcNAc is on the 3-arm

UniCarb-DB: #396

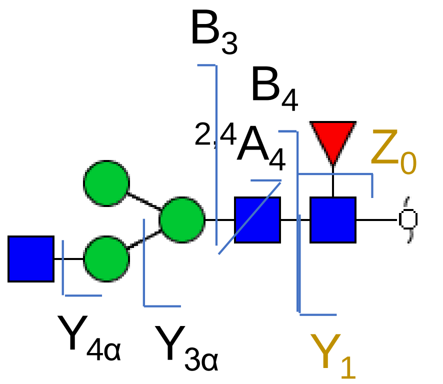

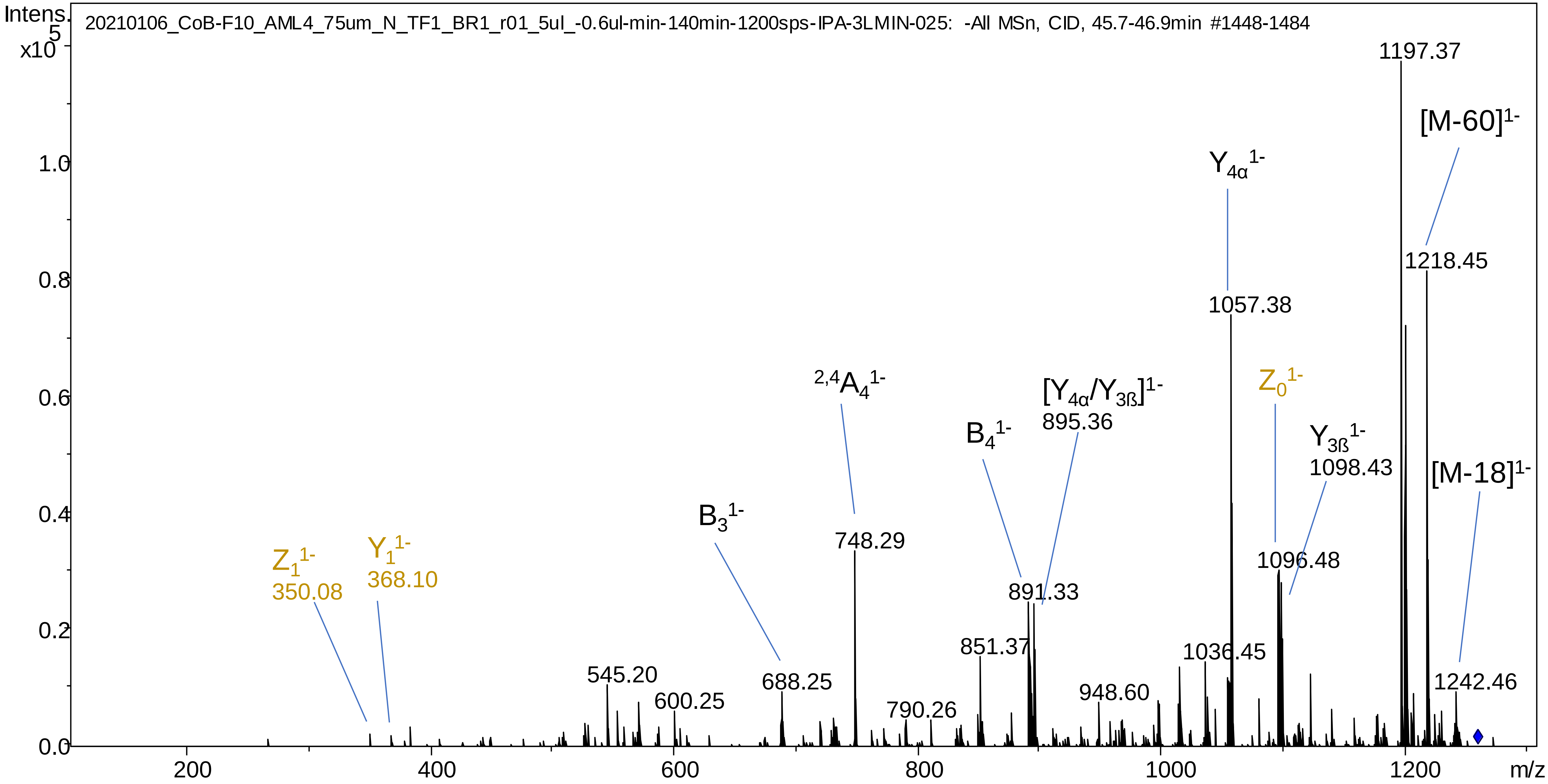

Glycan 36b

H3N3F1

Depicted MS² was obtained from analysis of cell line: TF-1

**Monoisotopic mass:**	**1261.48 Da**
**Charge observed:**	**1-**
**Theoretical ion:**	***m/z* 1260.47**
**Observed ion:**	***m/z* 1260.49**
**Mass deviation:**	***m/z* 0.02**
**Retention time:**	**59.1 min**

UniCarb-DB: #2574

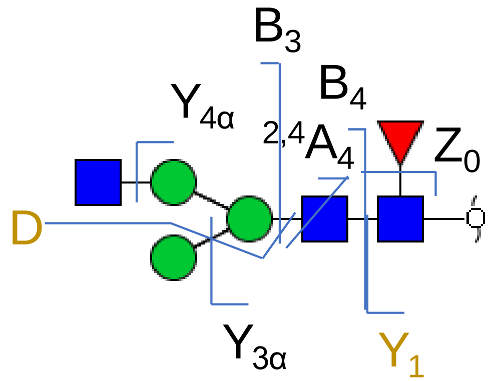

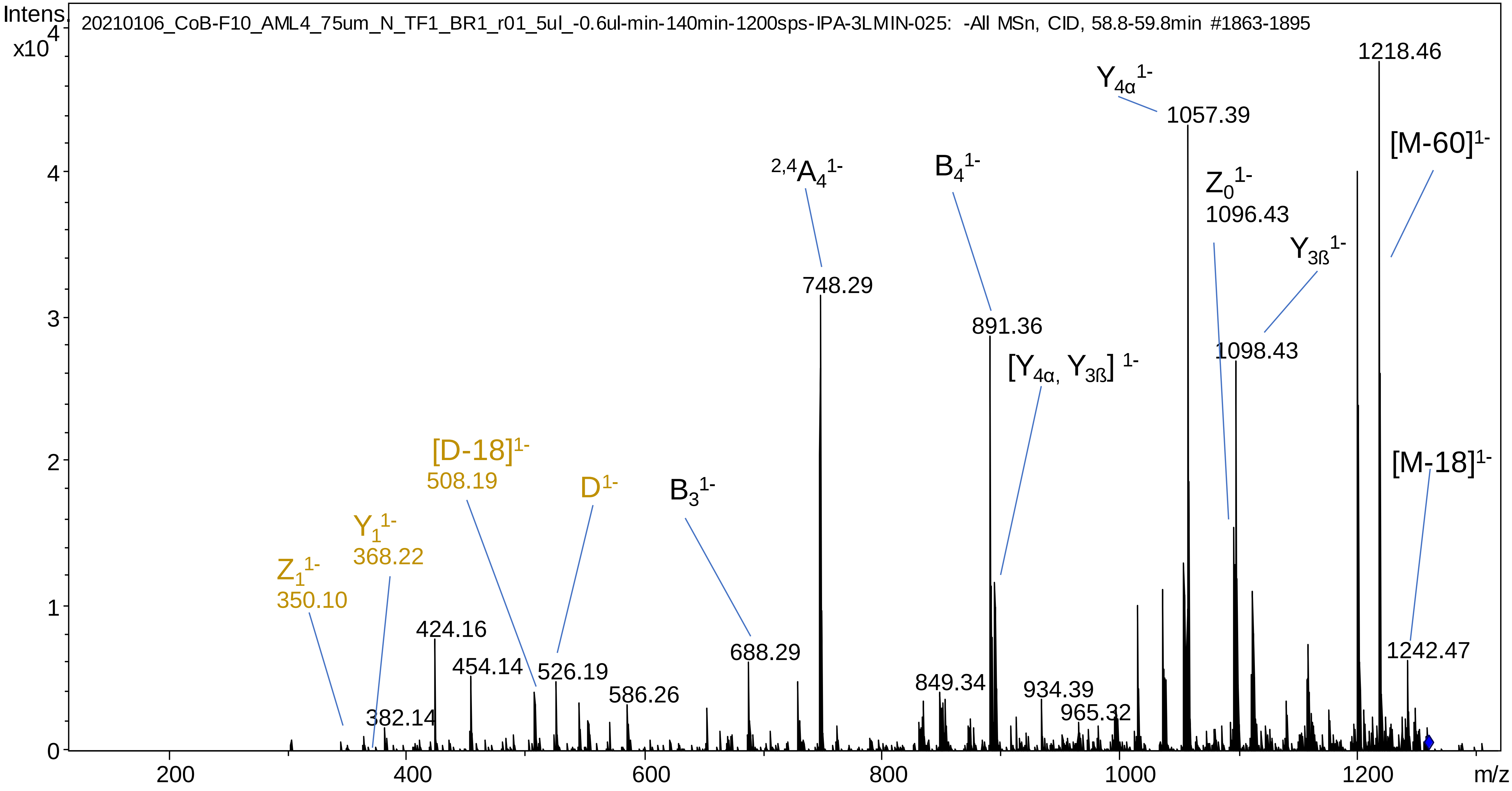

Glycan 37

H6N3

Depicted MS^2 was obtained from analysis of cell line: MV4-11

**Monoisotopic mass:**	**1601.59 Da**
**Charge observed:**	**2-**
**Theoretical ion:**	***m/z* 799.78**
**Observed ion:**	***m/z* 799.82**
**Mass deviation:**	***m/z* 0.04**
**Retention time:**	**34.9 min**

UniCarb-DB: #2482

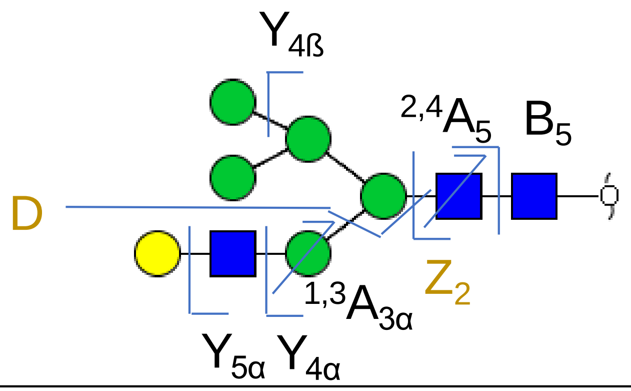

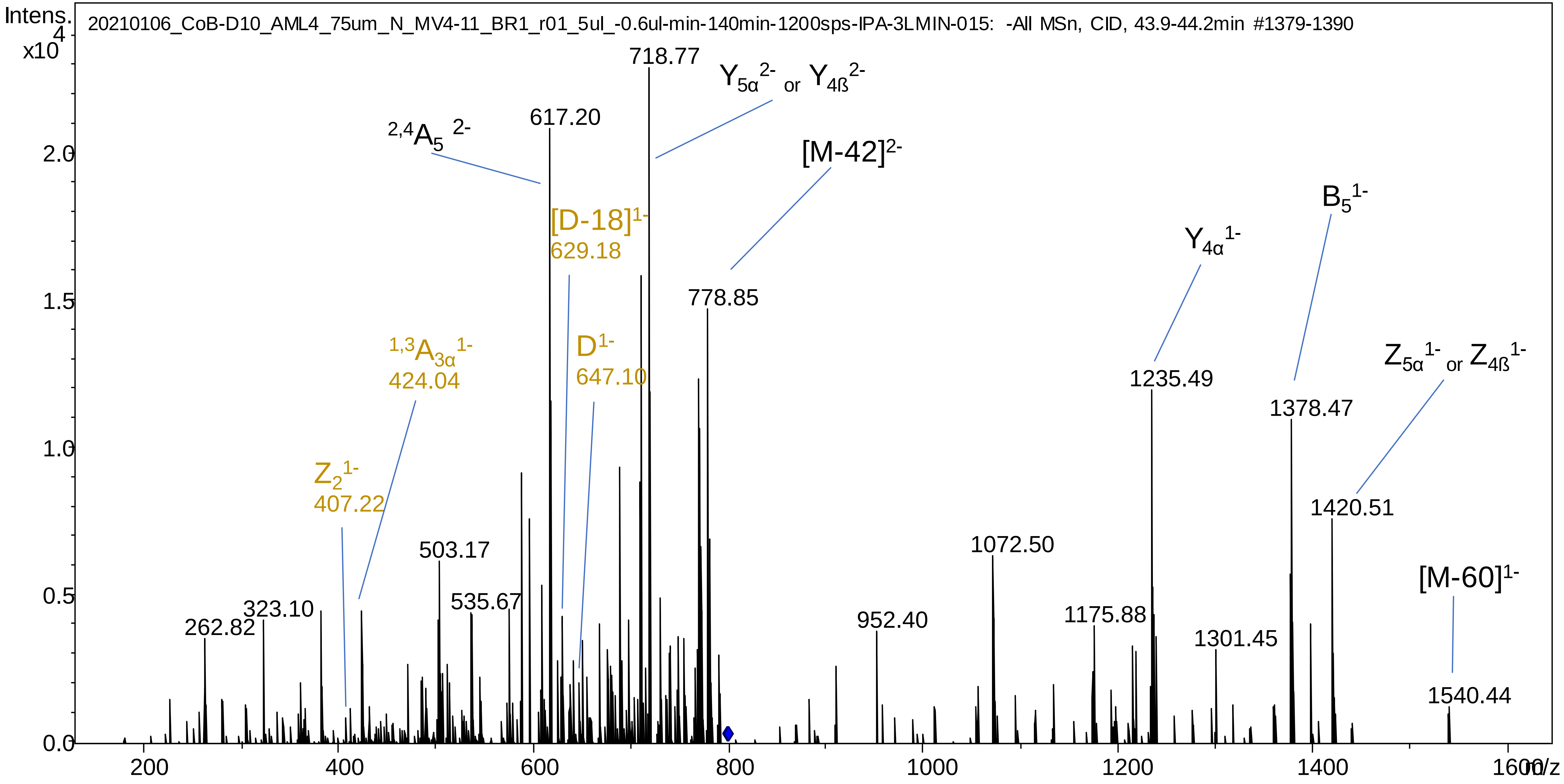

Glycan 38

H6N3P1

Depicted MS² was obtained from analysis of cell line: THP-1

**Monoisotopic mass:**	**1681.55 Da**
**Charge observed:**	**2-**
**Theoretical ion:**	***m/z* 839.77**
**Observed ion:**	***m/z* 839.79**
**Mass deviation:**	***m/z* 0.02**
**Retention time:**	**34.8 min**

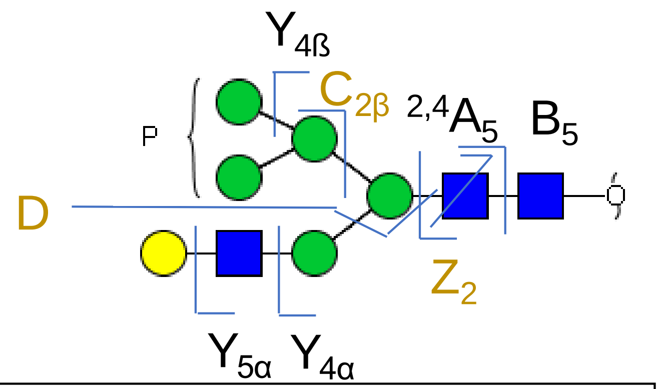

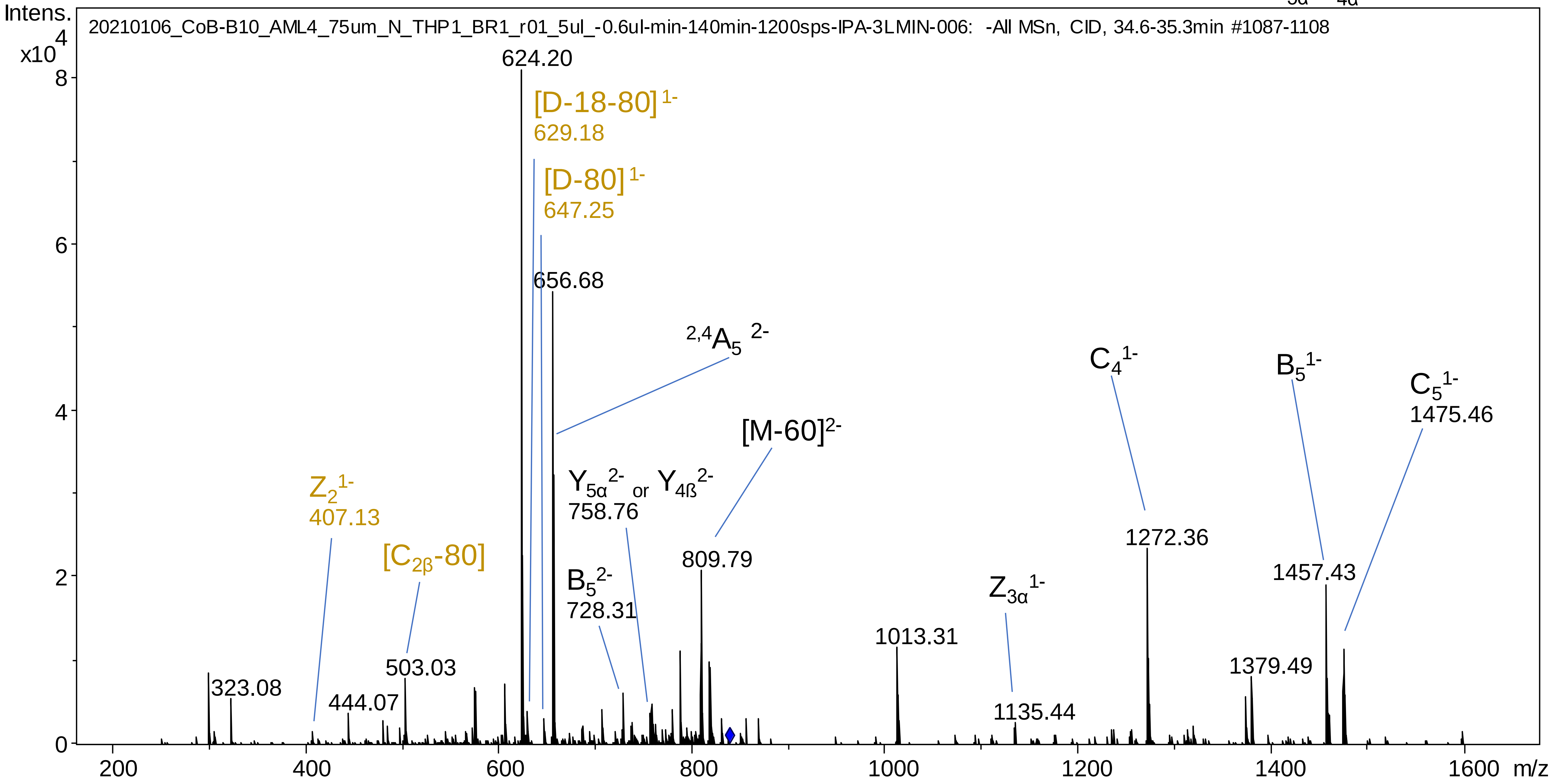

Glycan 39a

H4N3F1S1

Depicted MS² was obtained from analysis of cell line: MOLM-13

Monoisotopic mass: 1714.63 Da
Charge observed: 2-
Theoretical ion: *m/z* 856.30
Observed ion: *m/z* 856.35
Mass deviation: *m/z* 0.05
Retention time: 48.7 min
Note: α-2.6 sialic acid linkage confirmed by neuraminidase S and A treatment

UniCarb-DB: #416

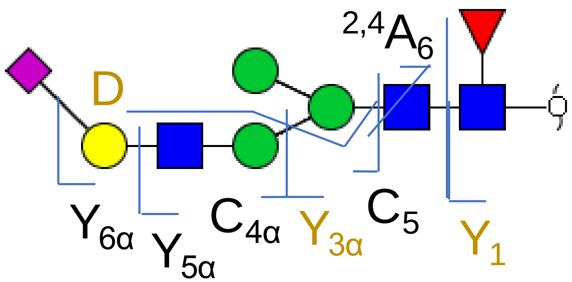

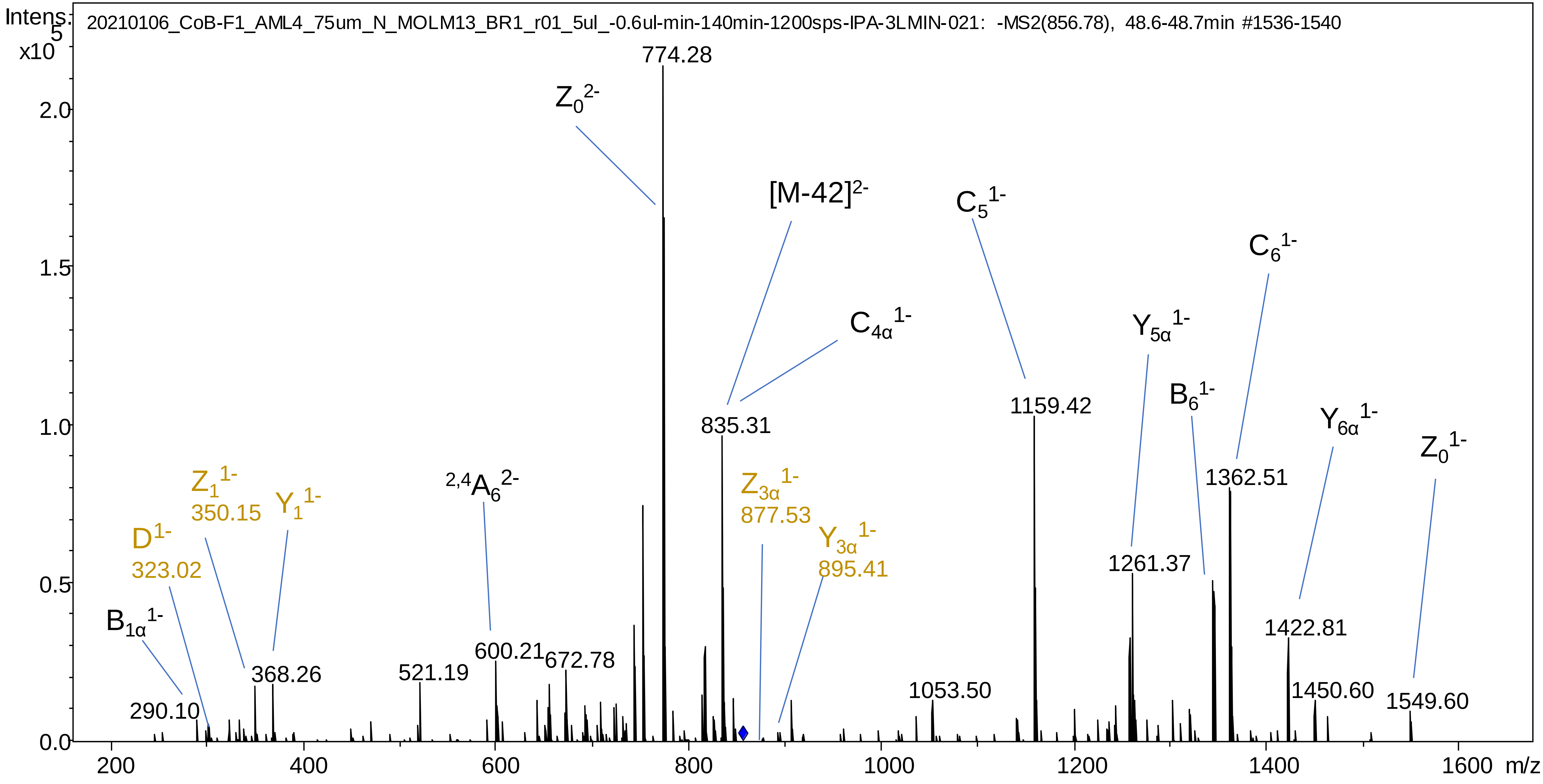

Glycan 39b

H4N3F1S1

Depicted MS² was obtained from analysis of cell line: MOLM-13

Monoisotopic mass: 1714.63 Da
Charge observed: 2-
Theoretical ion: *m/z* 856.30
Observed ion: *m/z* 856.36
Mass deviation: *m/z* 0.06
Retention time: 57.9 min
Note: α-2,3 sialic acid linkage confirmed by neuraminidase S and A treatment

UniCarb-DB: #2220

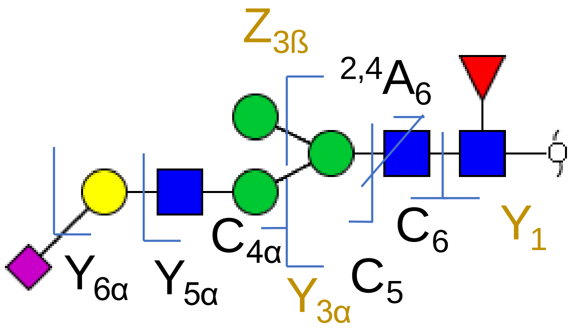

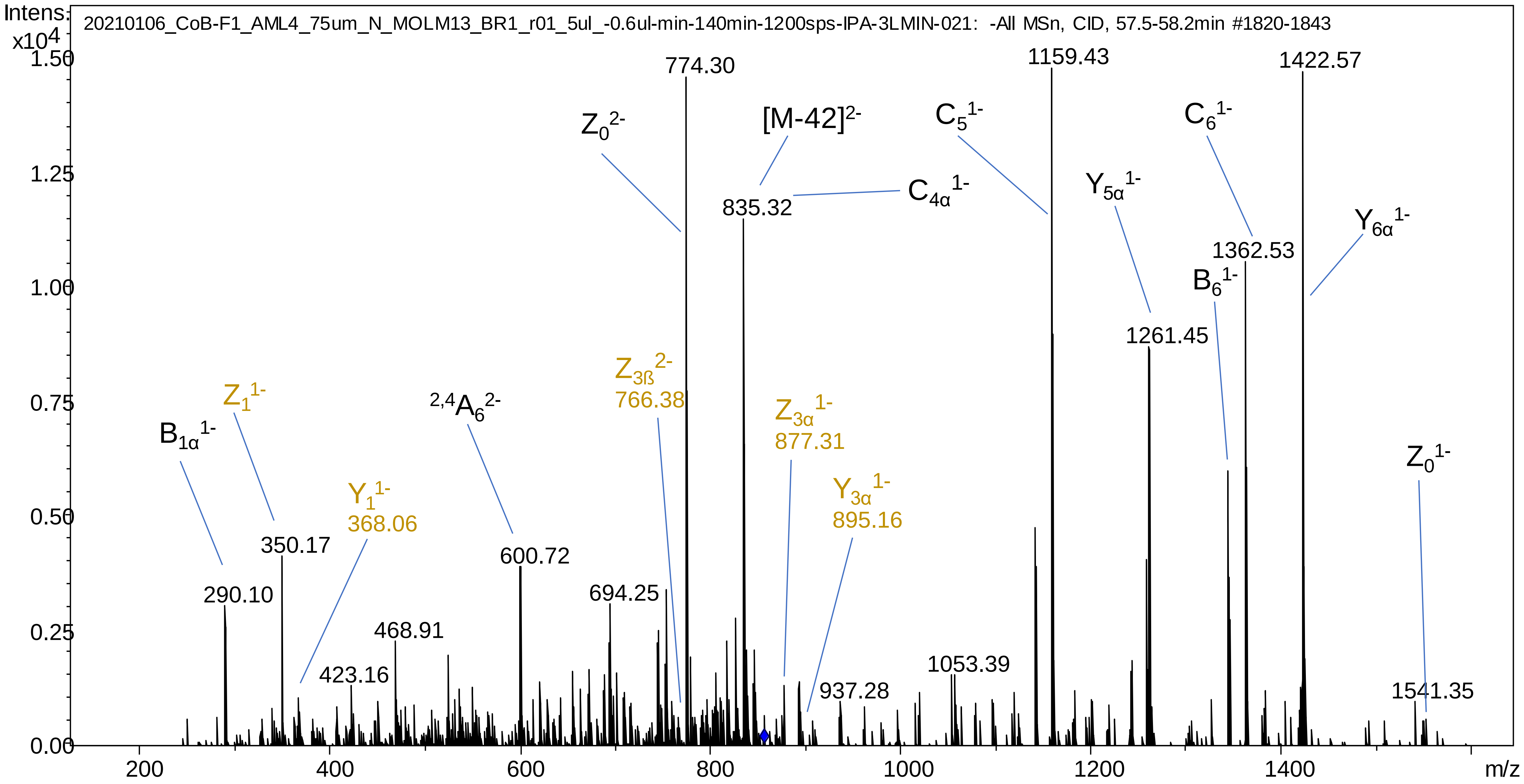

Glycan 39c

H4N3F1S1

Depicted MS^2 was obtained from analysis of cell line: MOLM-13

Monoisotopic mass: 1714.63 Da
Charge observed: 2-
Theoretical ion: *m/z* 856.30
Observed ion: *m/z* 856.34
Mass deviation: *m/z* 0.04
Retention time: 67.7 min
Note: α-2,6 sialic acid linkage confirmed by neuraminidase S and A treatment

UniCarb-DB: #2488

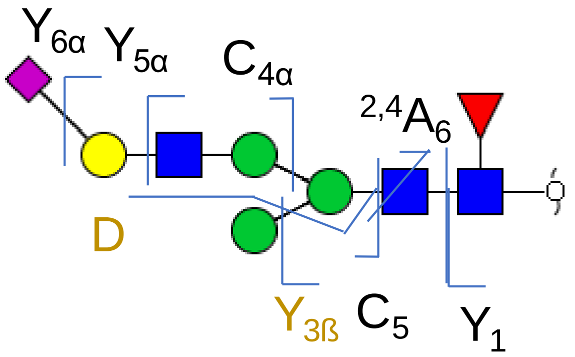

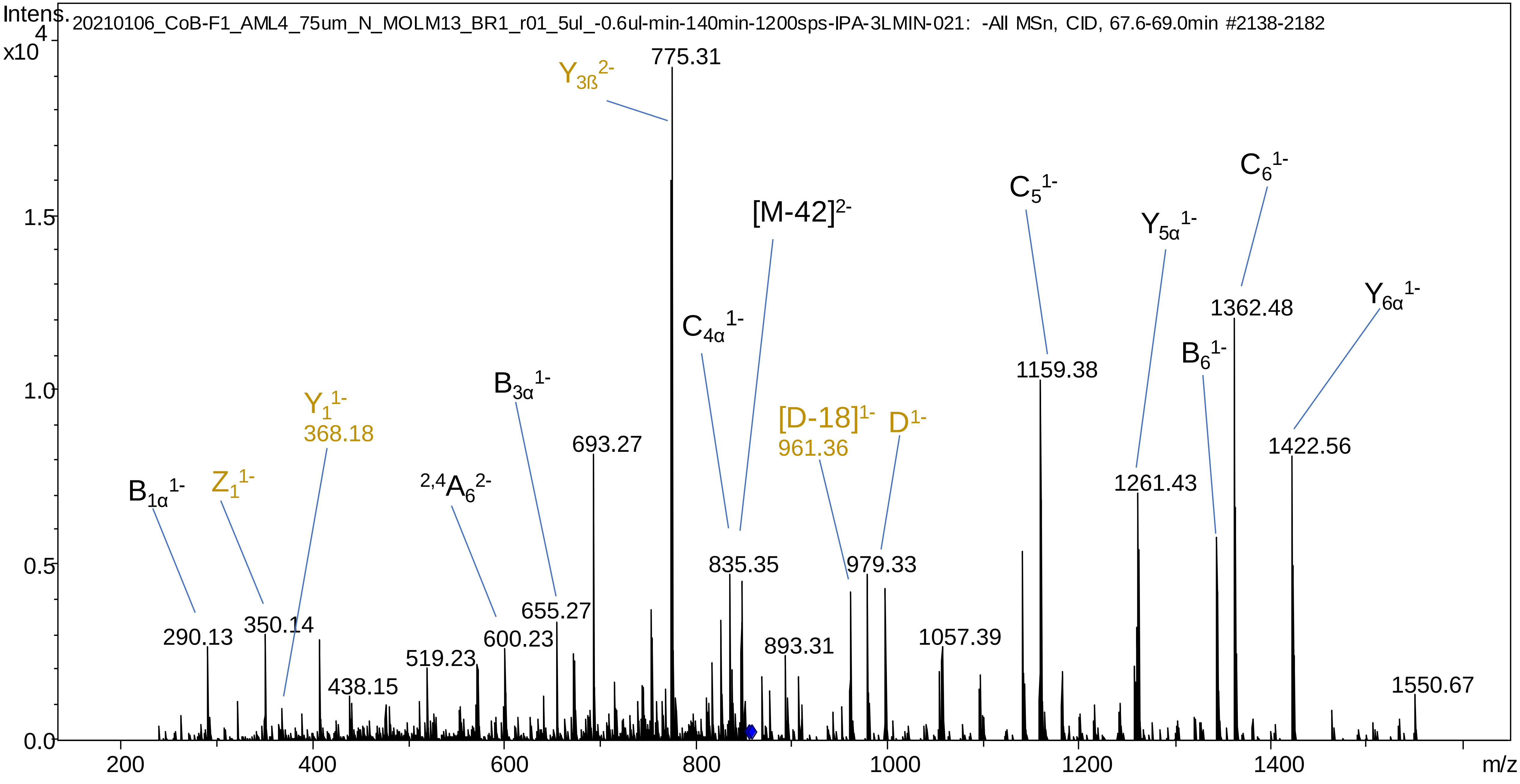

Glycan 40a

H6N3S1

Depicted MS² was obtained from analysis of cell line: TF-1

Monoisotopic mass: 1892.68 Da
Charge observed: 2-
Theoretical ion: *m/z* 945.33
Observed ion: *m/z* 945.36
Mass deviation: *m/z* 0.03
Retention time: 43.5 min
Note: α-2,6 sialic acid linkage confirmed by neuraminidase S and A treatment

UniCarb-DB: #430

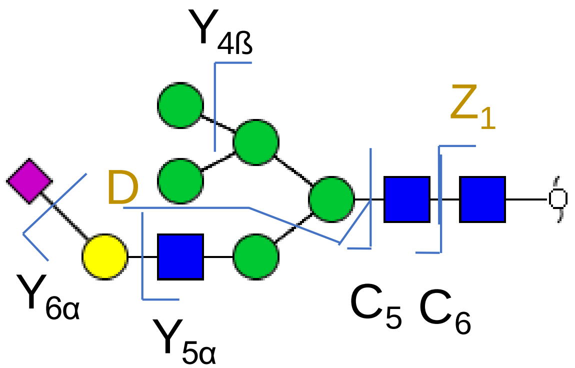

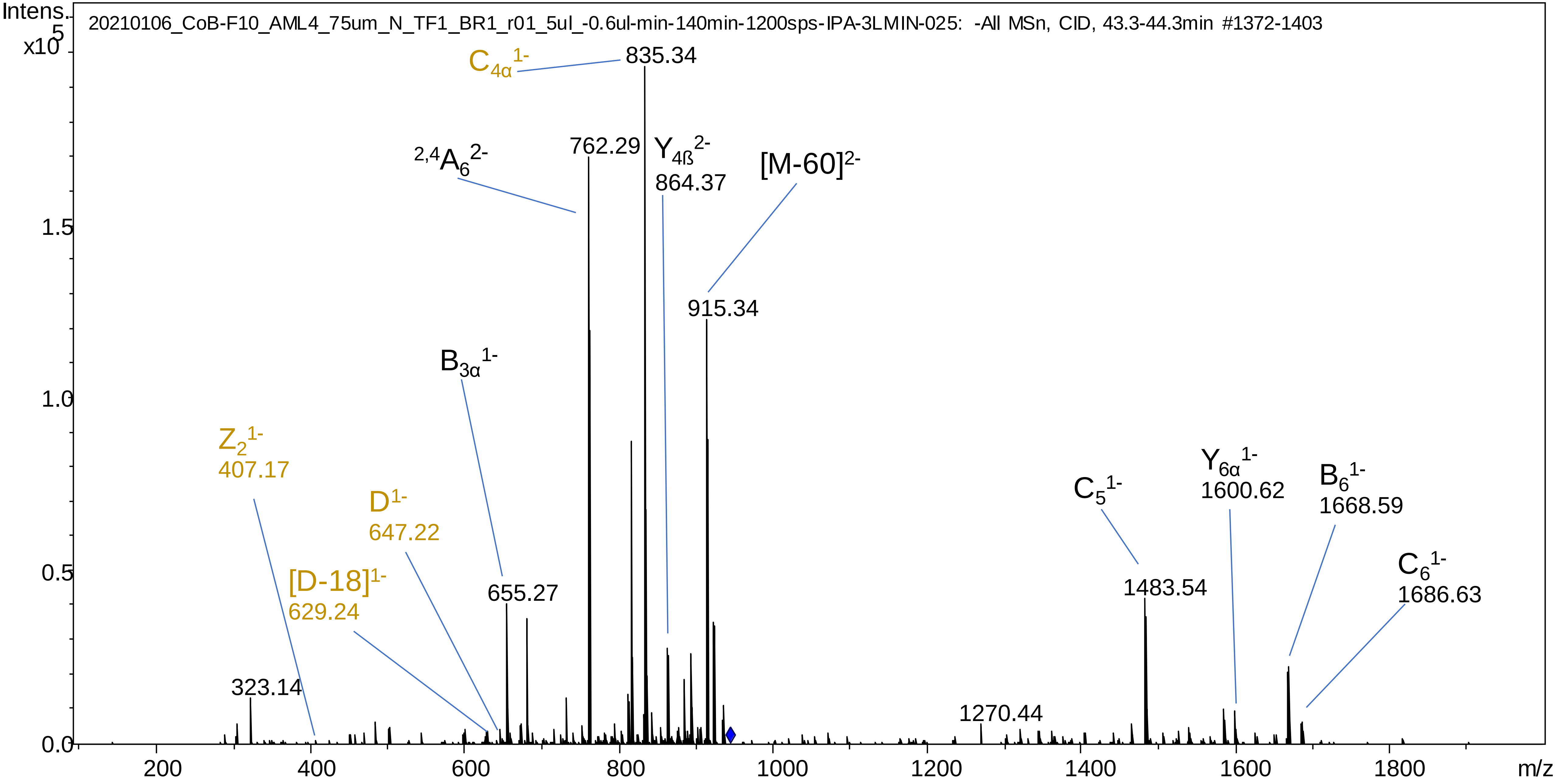

Glycan 40b

H6N3S1

Depicted MS^2 was obtained from analysis of cell line: TF-1

**Monoisotopic mass:**	**1892.68 Da**
**Charge observed:**	**2-**
**Theoretical ion:**	***m/z* 945.35**
**Observed ion:**	***m/z* 945.36**
**Mass deviation:**	***m/z* 0.01**
**Retention time:**	**53.0 min**

Note: α-2,3 sialic acid linkage confirmed by neuraminidase S and A treatment

UniCarb-DB: #2523

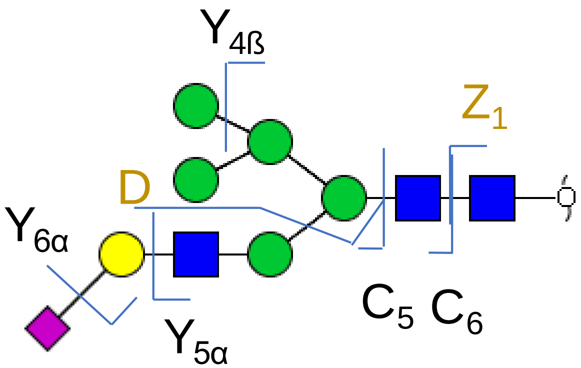

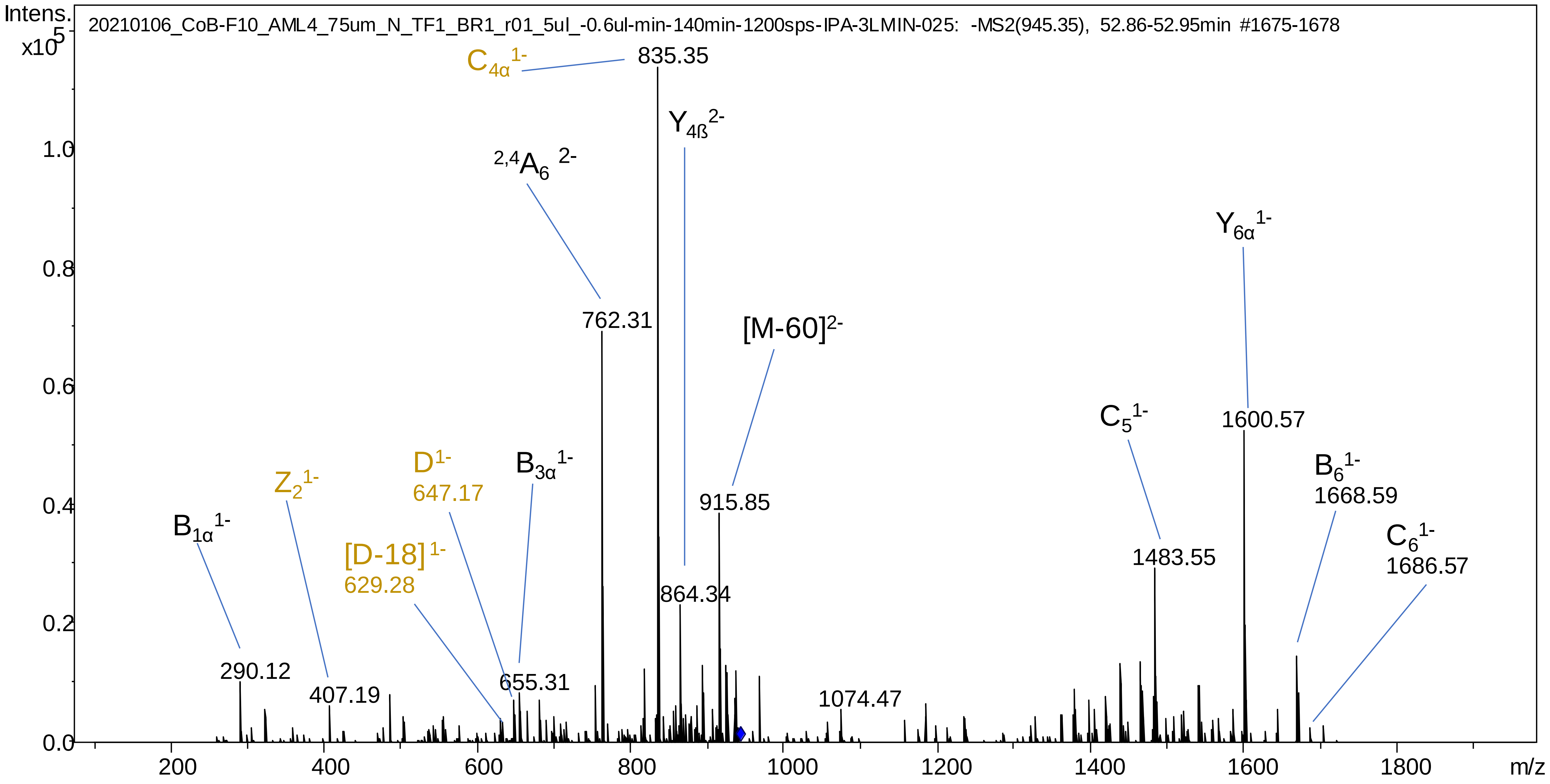

Supplement: Supplementary file 1 [file cells-10-03058-s001.zip › Supplementary_Information_1.pdf]
