# Supplementary material for: Integrated N- and O-Glycomics of Acute Myeloid Leukemia (AML) Cell Lines
Source: Cells. 2021 Nov 6;10(11):3058. doi: 10.3390/cells10113058 (PMC8616353; doi:10.3390/cells10113058)

Supplementary Information 2

O-Glycomics – Structural Elucidation

Integrated *N*- and *O*-Glycomics of Acute Myeloid Leukemia (AML) Cell Lines

Constantin Blöchl [1,2], Di Wang [1], Katarina Madunic [1], Guinevere S. M. Lageveen-Kammeijer [1], Christian G. Huber [2,3,*], Manfred Wuhrer [1,*] and Tao Zhang [1,*]

[1] *Center for Proteomics and Metabolomics, Leiden University Medical Center, Albinusdreef 2, Leiden, the Netherlands; constantin.bloechl@sbg.ac.at, d.wang@lumc.nl, k.madunic@lumc.nl, g.s.m.kammeijer@lumc.nl, m.wuhrer@lumc.nl, t.zhang@lumc.nl*

[2] *Department of Biosciences, University of Salzburg, Hellbrunnerstrasse 34, Salzburg, Austria; constantin.bloechl@sbg.ac.at, c.huber@sbg.ac.at*

[3] *Cancer Cluster Salzburg, Department of Biosciences, University of Salzburg, Hellbrunnerstrasse 34, Salzburg, Austria; c.huber@sbg.ac.at*

** Correspondence: c.huber@sbg.ac.at, m.wuhrer@lumc.nl, t.zhang@lumc.nl*

Glycan 1

H1N1

Depicted MS^2 was obtained from analysis of cell line: HL-60

**Monoisotopic mass:**	**385.16 Da**
**Charge observed:**	**1-**
**Theoretical ion:**	***m/z* 384.15**
**Observed ion:**	***m/z* 384.05**
**Mass deviation:**	***m/z* 0.10**
**Retention time:**	**10.6 min**

UniCarb-DB: #171

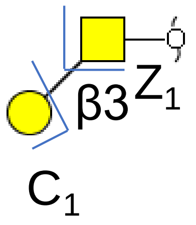

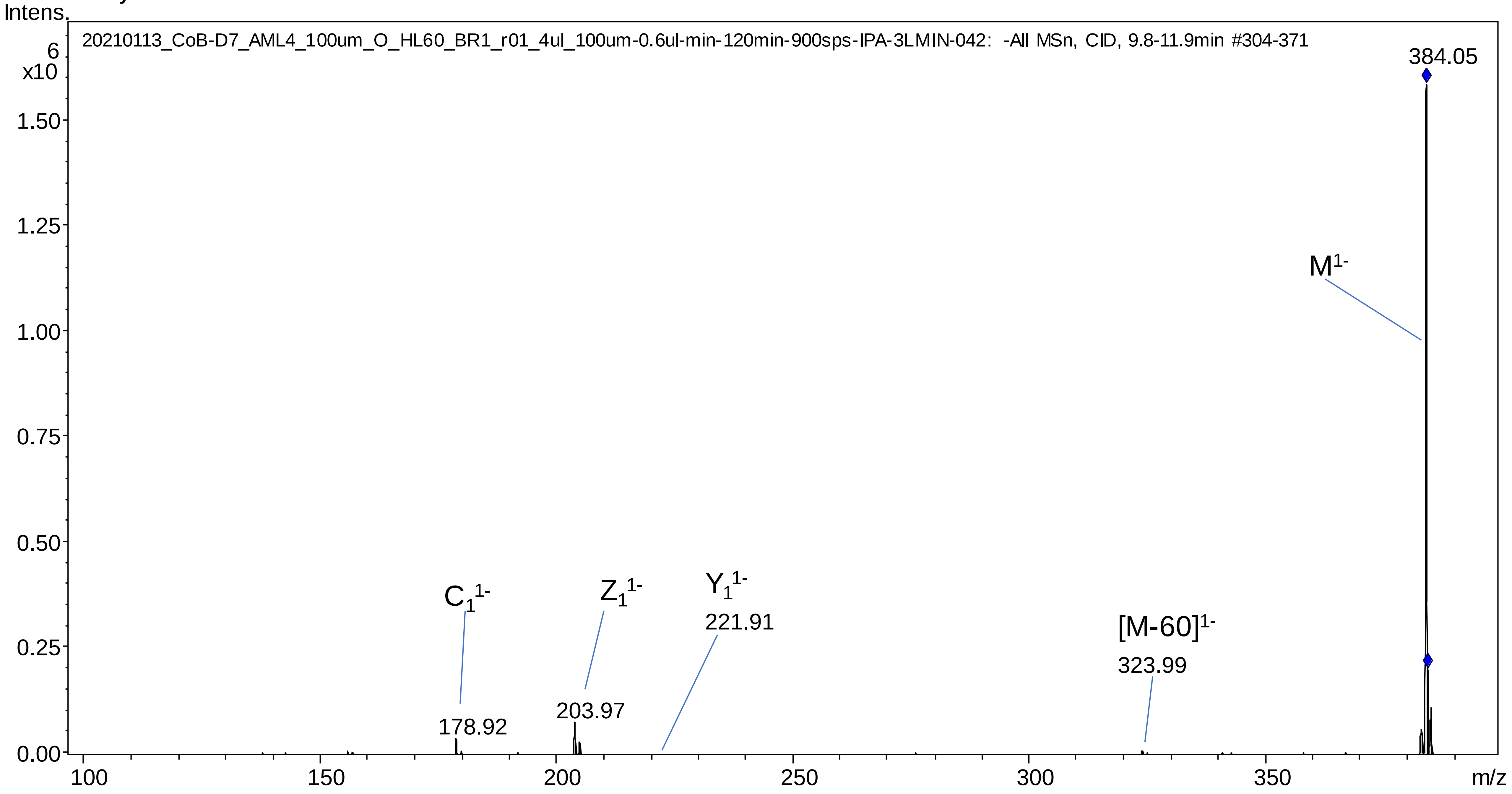

Glycan 2

H1N2

Depicted MS² was obtained from analysis of cell line: HL-60

**Monoisotopic mass:**	**588.24 Da**
**Charge observed:**	**1-**
**Theoretical ion:**	***m/z* 587.23**
**Observed ion:**	***m/z* 587.19**
**Mass deviation:**	***m/z* 0.04**
**Retention time:**	**33.3 min**

UniCarb-DB: #177

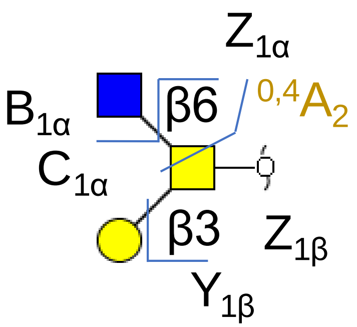

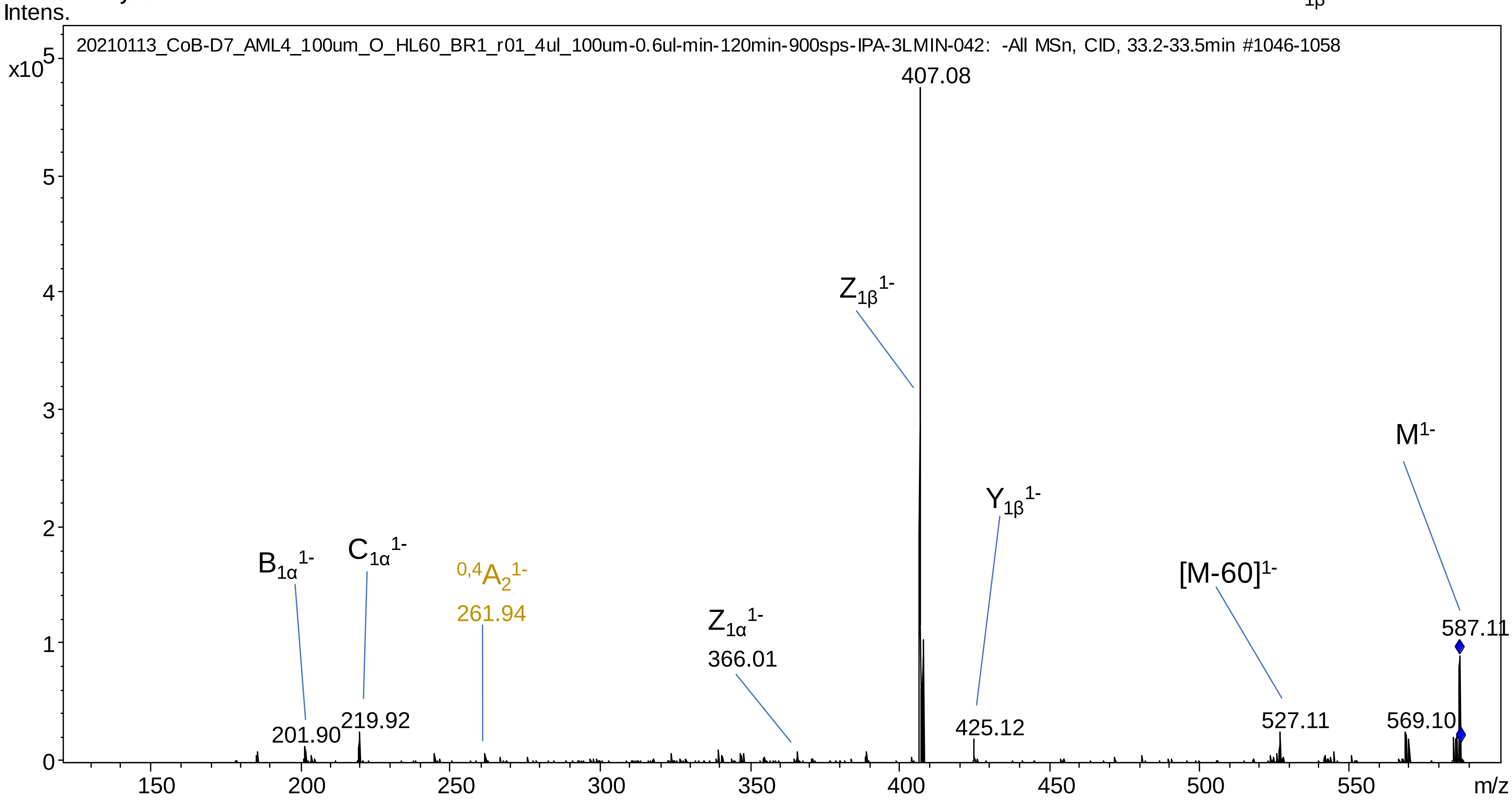

Glycan 3

H1N2Su1

Depicted MS² was obtained from analysis of cell line: MV4-11

**Monoisotopic mass:**	**668.20 Da**
**Charge observed:**	**1-**
**Theoretical ion:**	***m/z* 667.19**
**Observed ion:**	***m/z* 667.11**
**Mass deviation:**	***m/z* 0.07**
**Retention time:**	**35.0 min**

UniCarb-DB: #3039

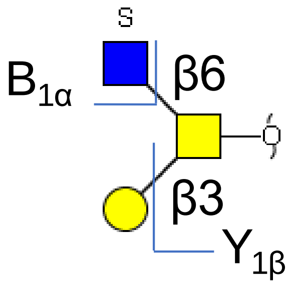

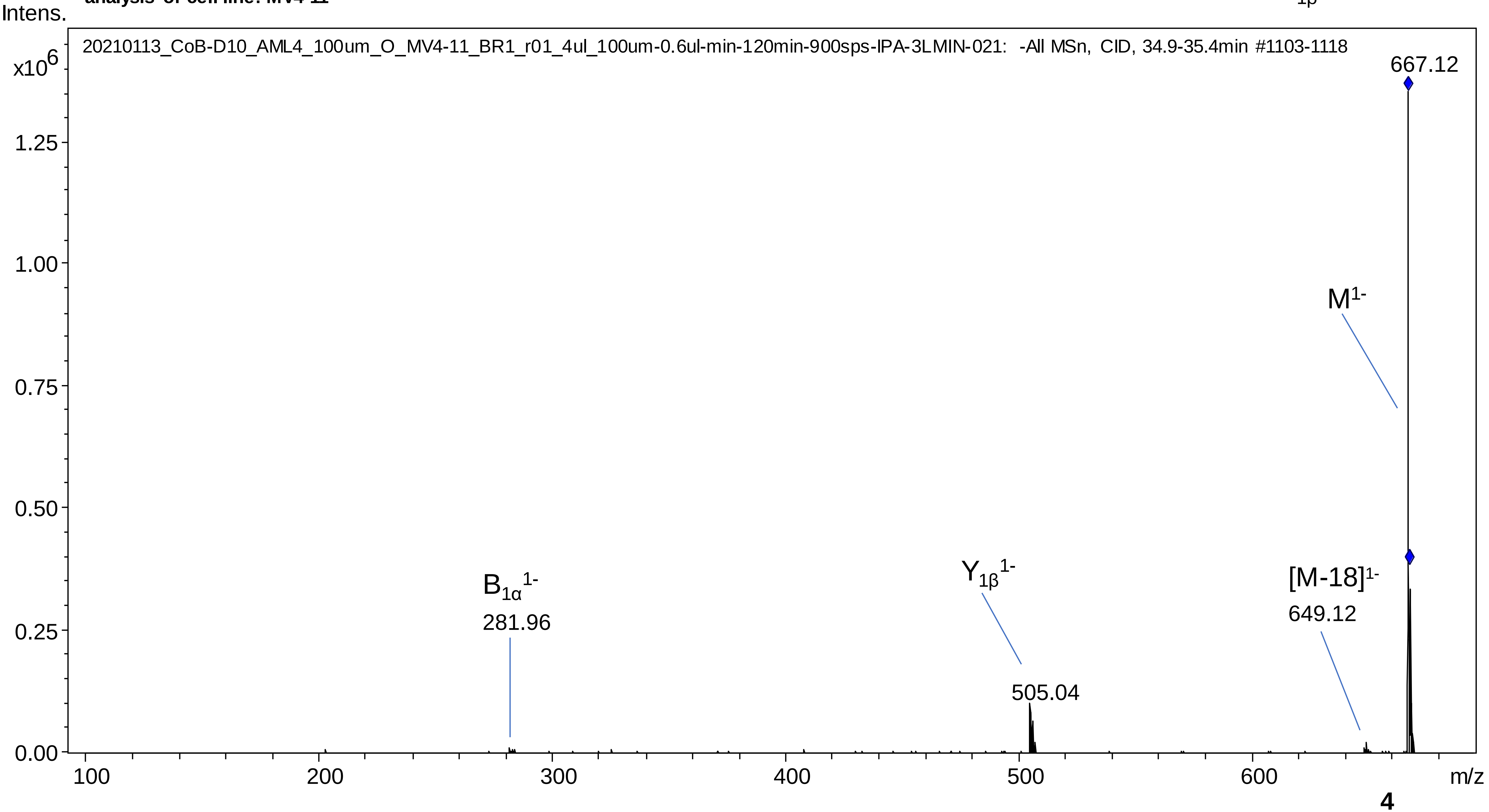

Glycan 4a

H1N1S1

Depicted MS[2] was obtained from analysis of cell line: TF-1

**Monoisotopic mass:**	**676.26 Da**
**Charge observed:**	**1-**
**Theoretical ion:**	***m/z* 675.25**
**Observed ion:**	***m/z* 675.17**
**Mass deviation:**	***m/z* 0.08**
**Retention time:**	**30.1 min**

Note: α-2,6 linkage of sialic acid confirmed by neuraminidase S and A treatment

UniCarb-DB: #41

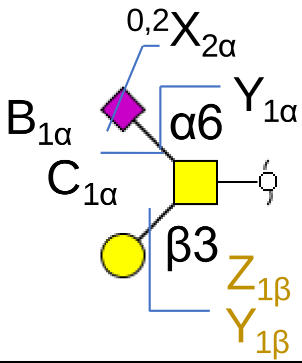

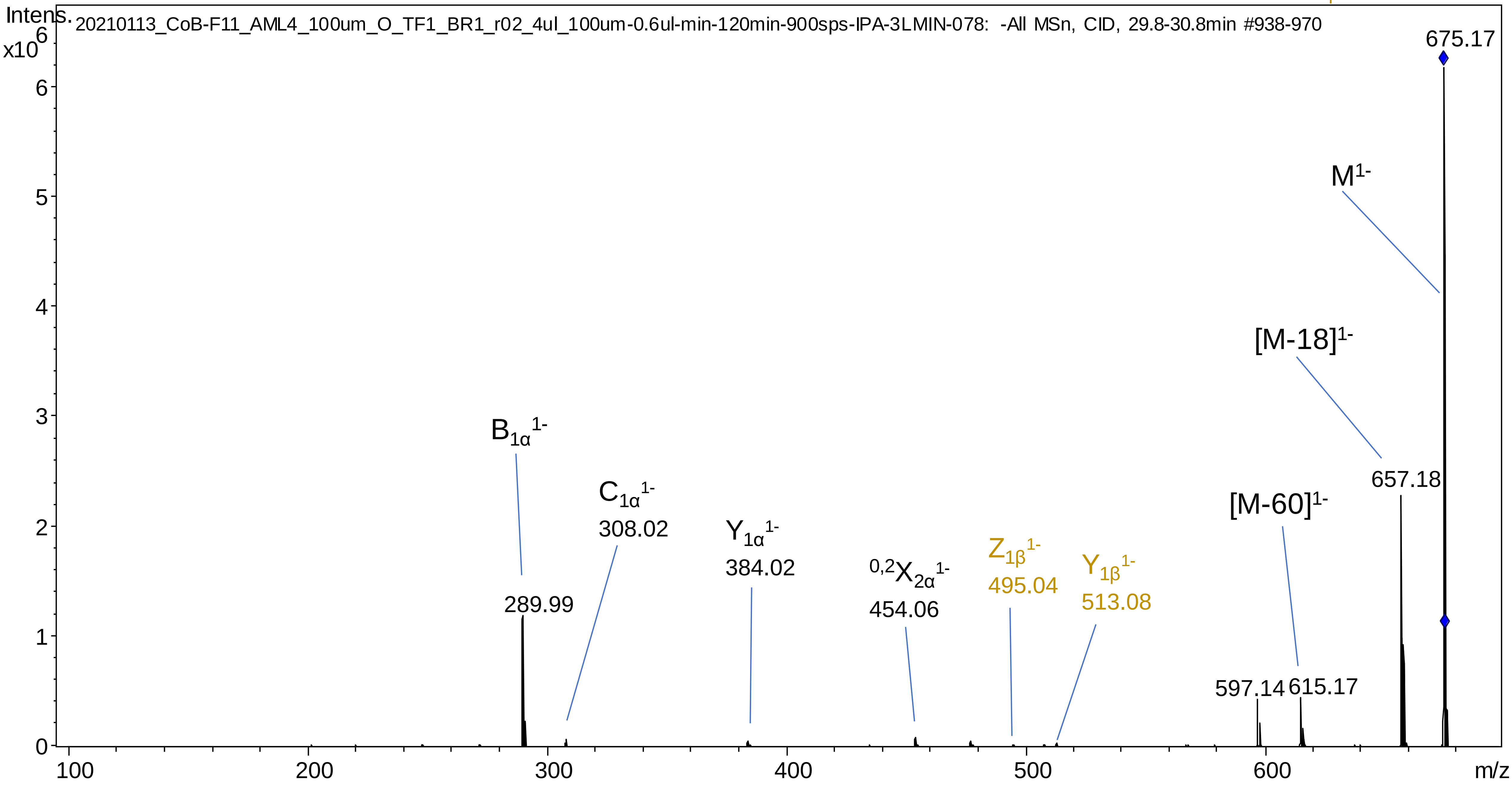

Glycan 4b

H1N1S1

Depicted MS² was obtained from analysis of cell line: TF-1

Monoisotopic mass: 676.26 Da
Charge observed: 1-
Theoretical ion: *m/z* 675.25
Observed ion: *m/z* 675.18
Mass deviation: *m/z* 0.07
Retention time: 37.1 min
Note: α-2,3 linkage of sialic acid confirmed by neuraminidase S and A treatment

UniCarb-DB: #146

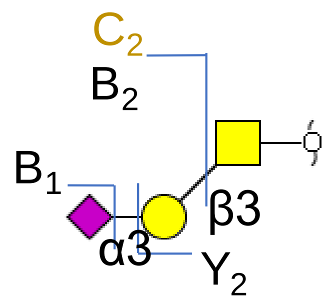

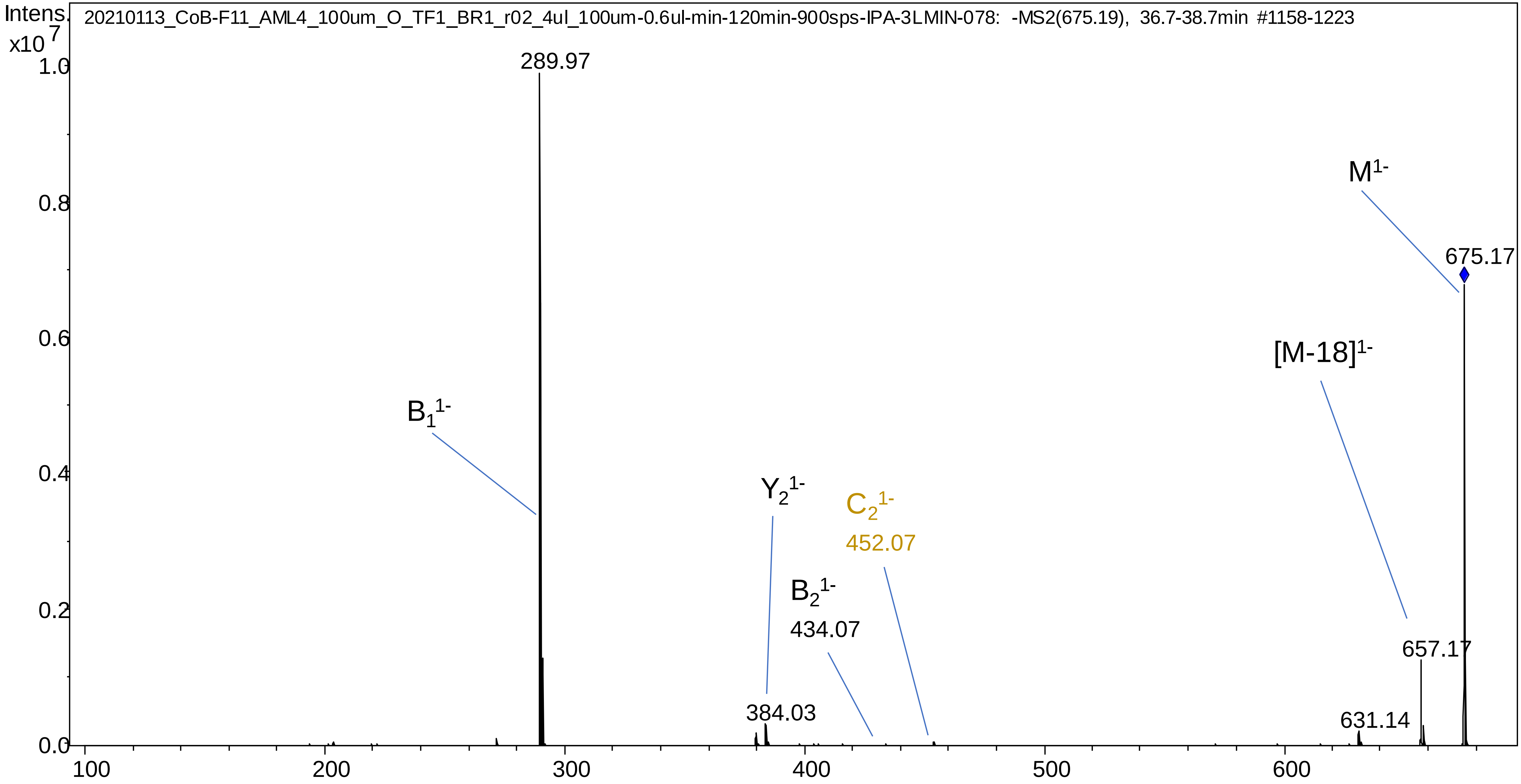

Glycan 5

H2N2

Depicted MS² was obtained from analysis of cell line: HL-60

**Monoisotopic mass:**	**750.29 Da**
**Charge observed:**	**1-**
**Theoretical ion:**	***m/z* 749.28**
**Observed ion:**	***m/z* 749.23**
**Mass deviation:**	***m/z* 0.05**
**Retention time:**	**43.9 min**

UniCarb-DB: #24

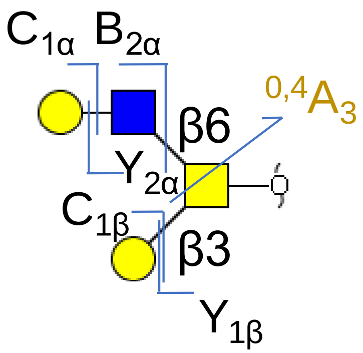

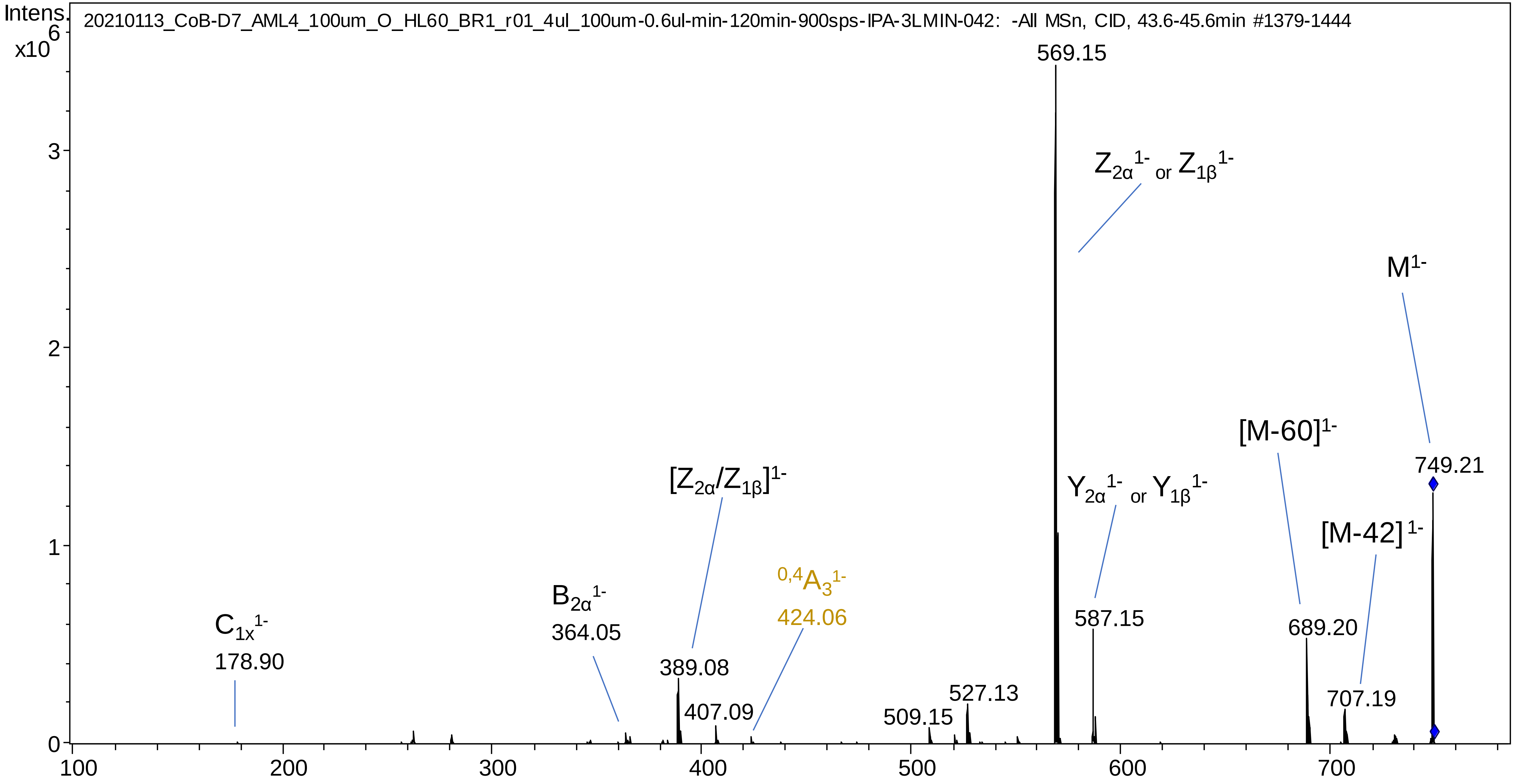

Glycan 6

H2N2Su1

Depicted MS^2 was obtained from analysis of cell line: MV4-11

**Monoisotopic mass:**	**830.25 Da**
**Charge observed:**	**1-**
**Theoretical ion:**	***m/z* 829.24**
**Observed ion:**	***m/z* 829.22**
**Mass deviation:**	***m/z* 0.02**
**Retention time:**	**43.7 min**

UniCarb-DB: #104

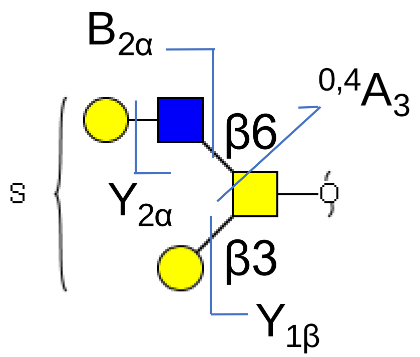

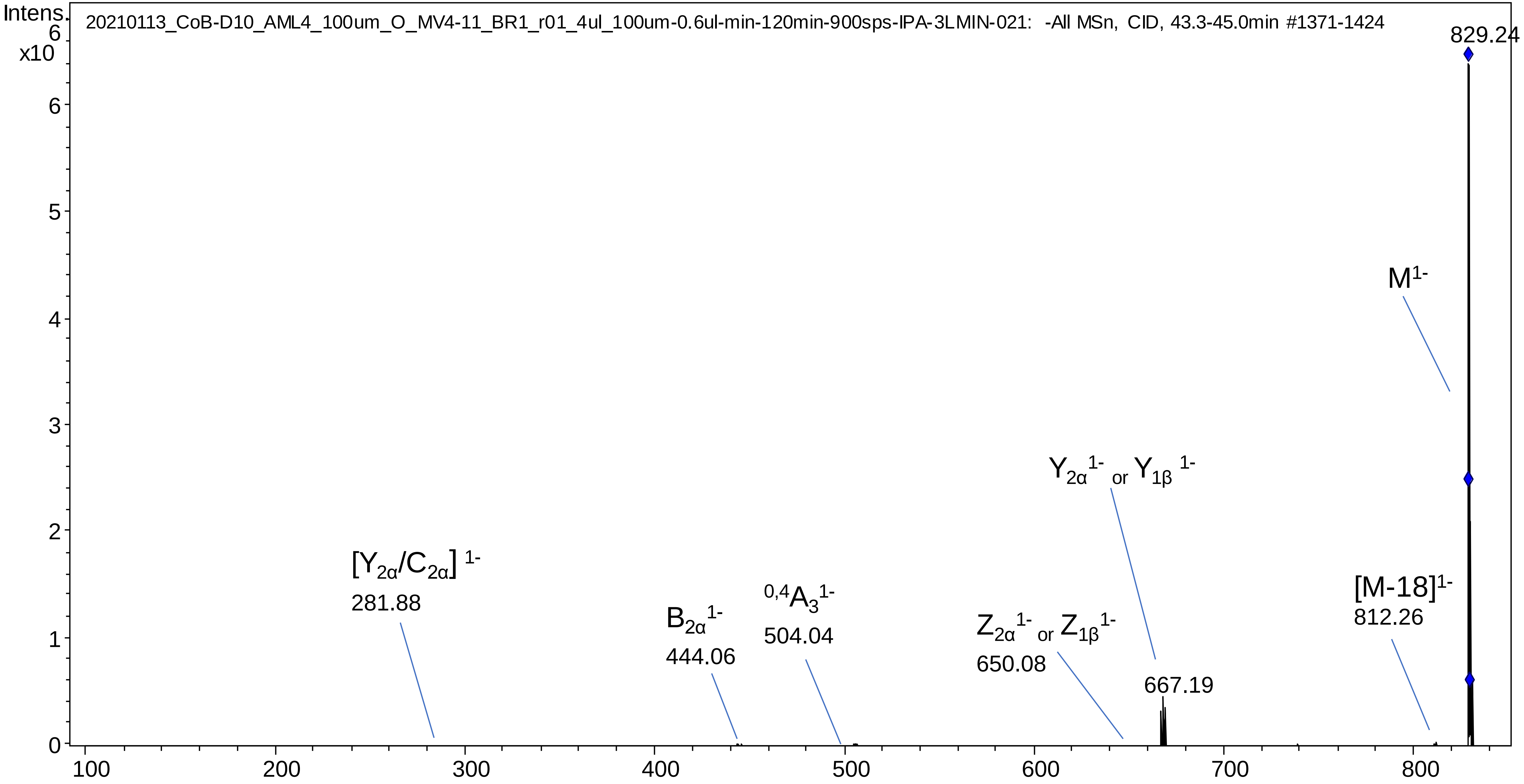

Glycan 7

H1N2S1

Depicted MS² was obtained from analysis of cell line: TF-1

**Monoisotopic mass:**	**879.34 Da**
**Charge observed:**	**1-**
**Theoretical ion:**	***m/z* 878.33**
**Observed ion:**	***m/z* 878.27**
**Mass deviation:**	***m/z* 0.05**
**Retention time:**	**46.0 min**

Note: α-2,3 linkage of sialic acid confirmed by neuraminidase S and A treatment

UniCarb-DB: #191

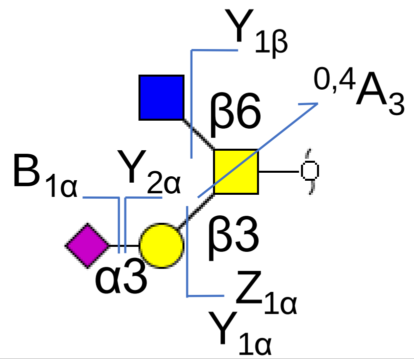

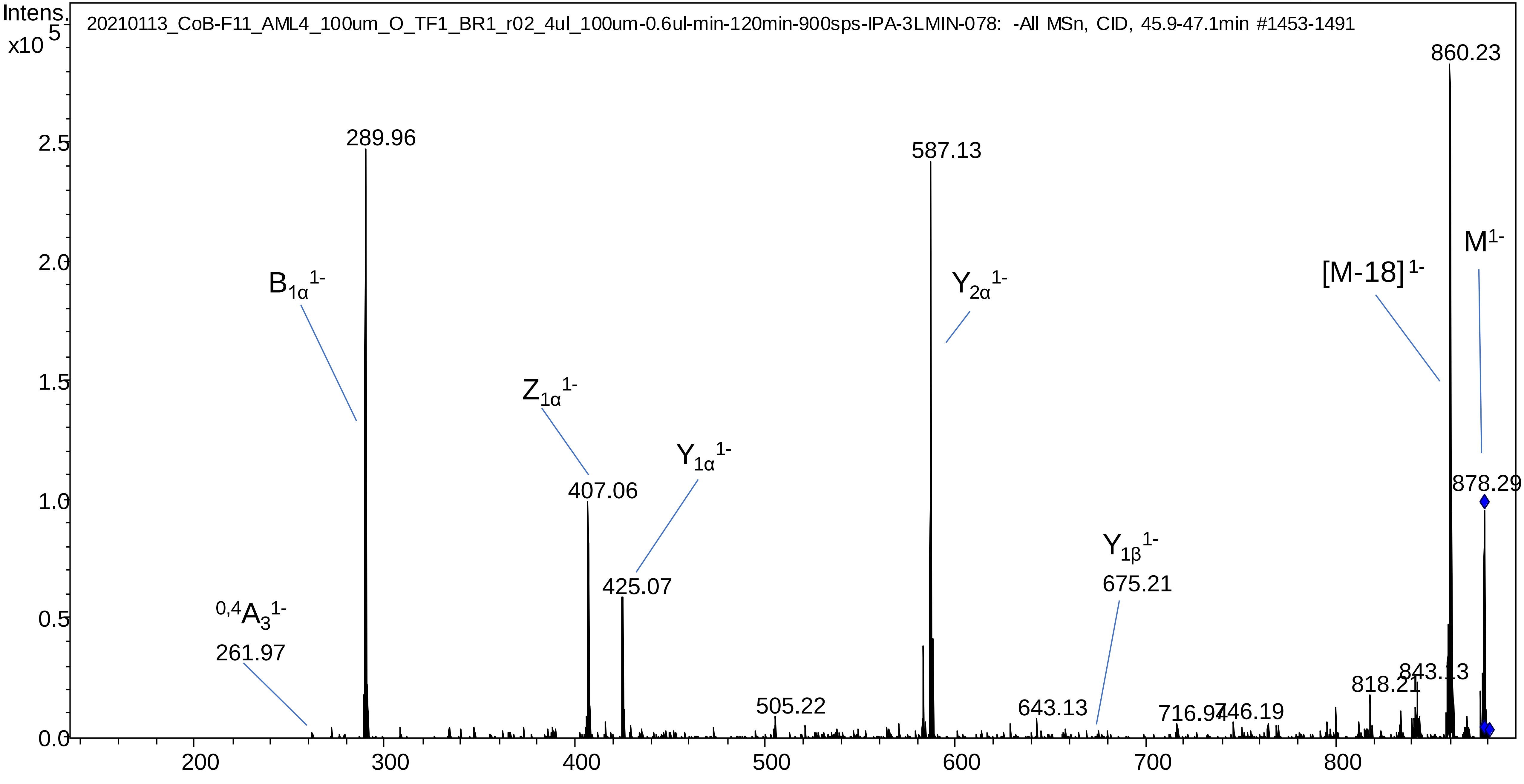

Glycan 8

H2N2F1

Depicted MS^2 was obtained from analysis of cell line: MV4-11

**Monoisotopic mass:**	**896.35 Da**
**Charge observed:**	**1-**
**Theoretical ion:**	***m/z* 895.34**
**Observed ion:**	***m/z* 895.28**
**Mass deviation:**	***m/z* 0.06**
**Retention time:**	**37.0 min**

Note: Characteristic *m/z* 348 ion for Lewis a

UniCarb-DB: #33

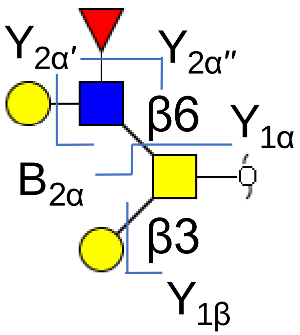

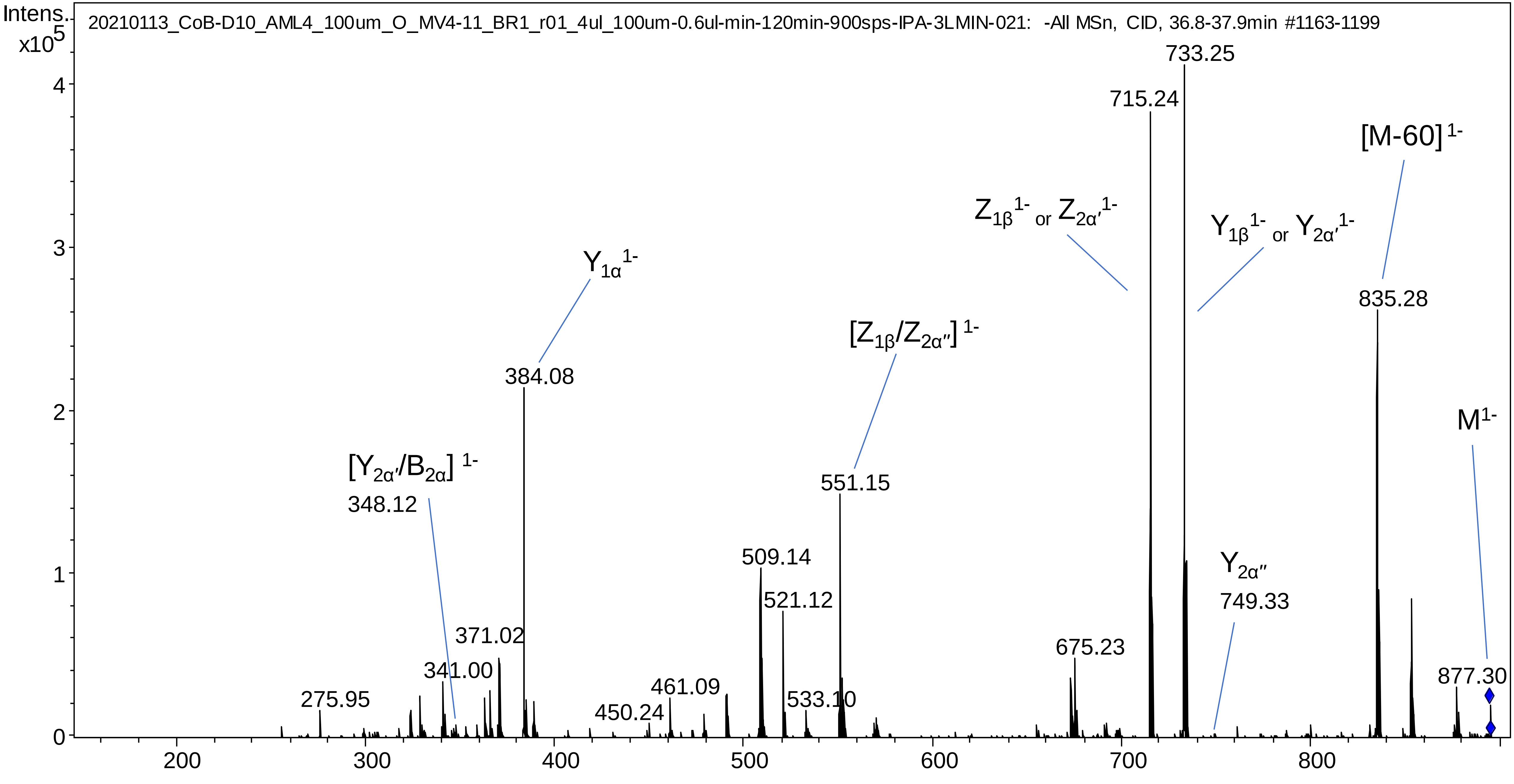

Glycan 9

H1N1S2

Depicted MS[2] was obtained from analysis of cell line: MV4-11

Monoisotopic mass: 967.35 Da
Charge observed: 1-
Theoretical ion: *m/z* 966.34 *m/z*
Observed ion: *m/z* 966.32 *m/z*
Mass deviation: *m/z* 0.02 *m/z*
Retention time: 38.1 min
Note: Linkage of sialic acids confirmed by neuraminidase S and A treatment

UniCarb-DB: #47

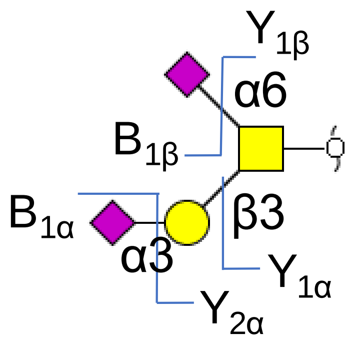

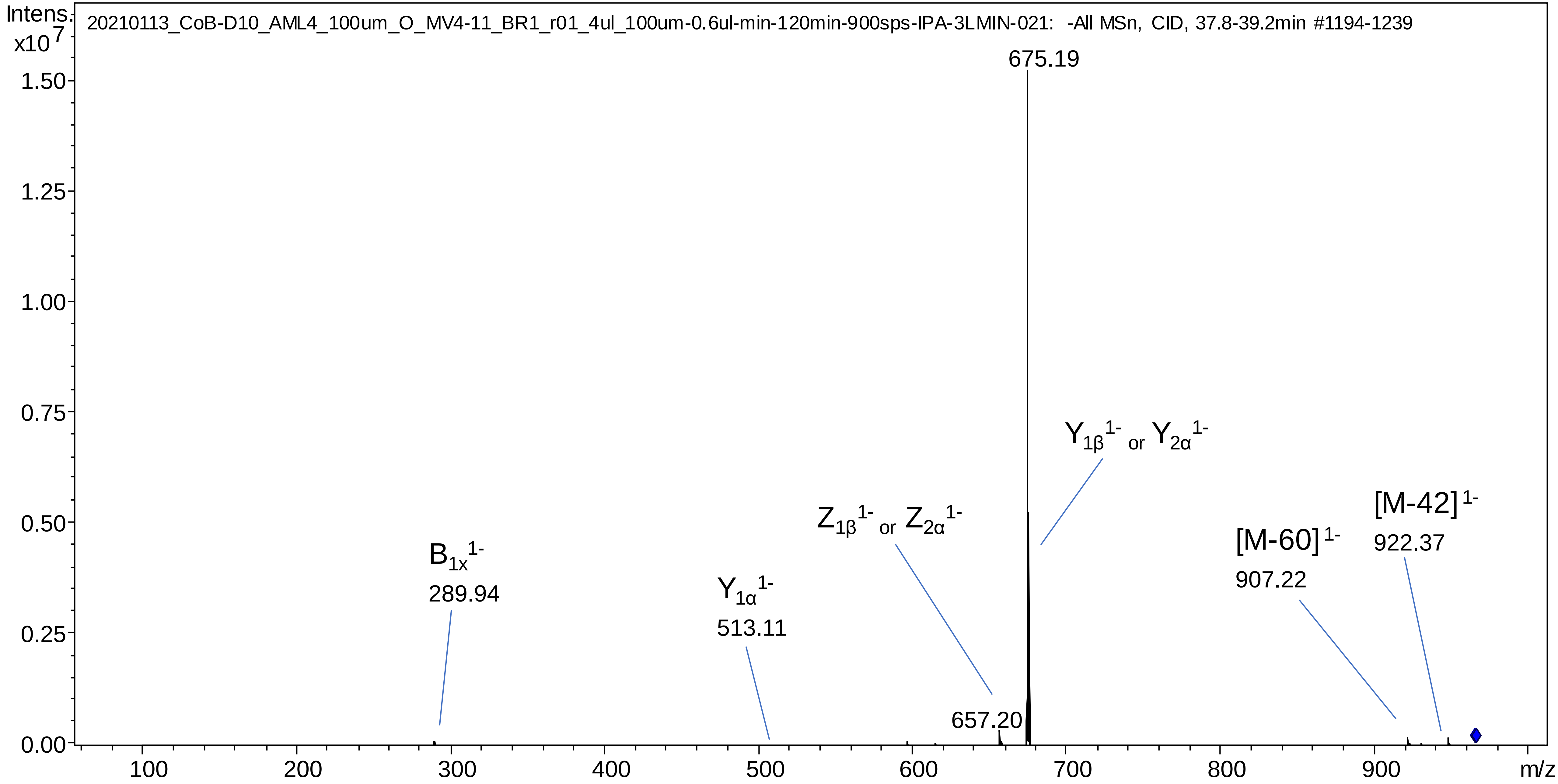

Glycan 10a

H2N2S1

Depicted MS[2] was obtained from analysis of cell line: TF-1

**Monoisotopic mass:**	**1041.40 Da**
**Charge observed:**	**1-**
**Theoretical ion:**	***m/z* 1040.39**
**Observed ion:**	***m/z* 1040.35**
**Mass deviation:**	***m/z* 0.04**
**Retention time:**	**53.5 min**

Note: α-2,3 linkage of sialic acid confirmed by neuraminidase S and A treatment

UniCarb-DB: #48

Elution order of isomers in line with Jin, Chunsheng, et al. *Mol. & Cell. Proteomics* 16.5 (2017): 743-758.

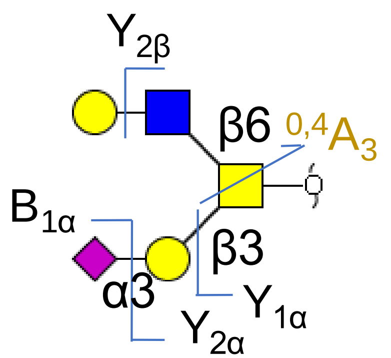

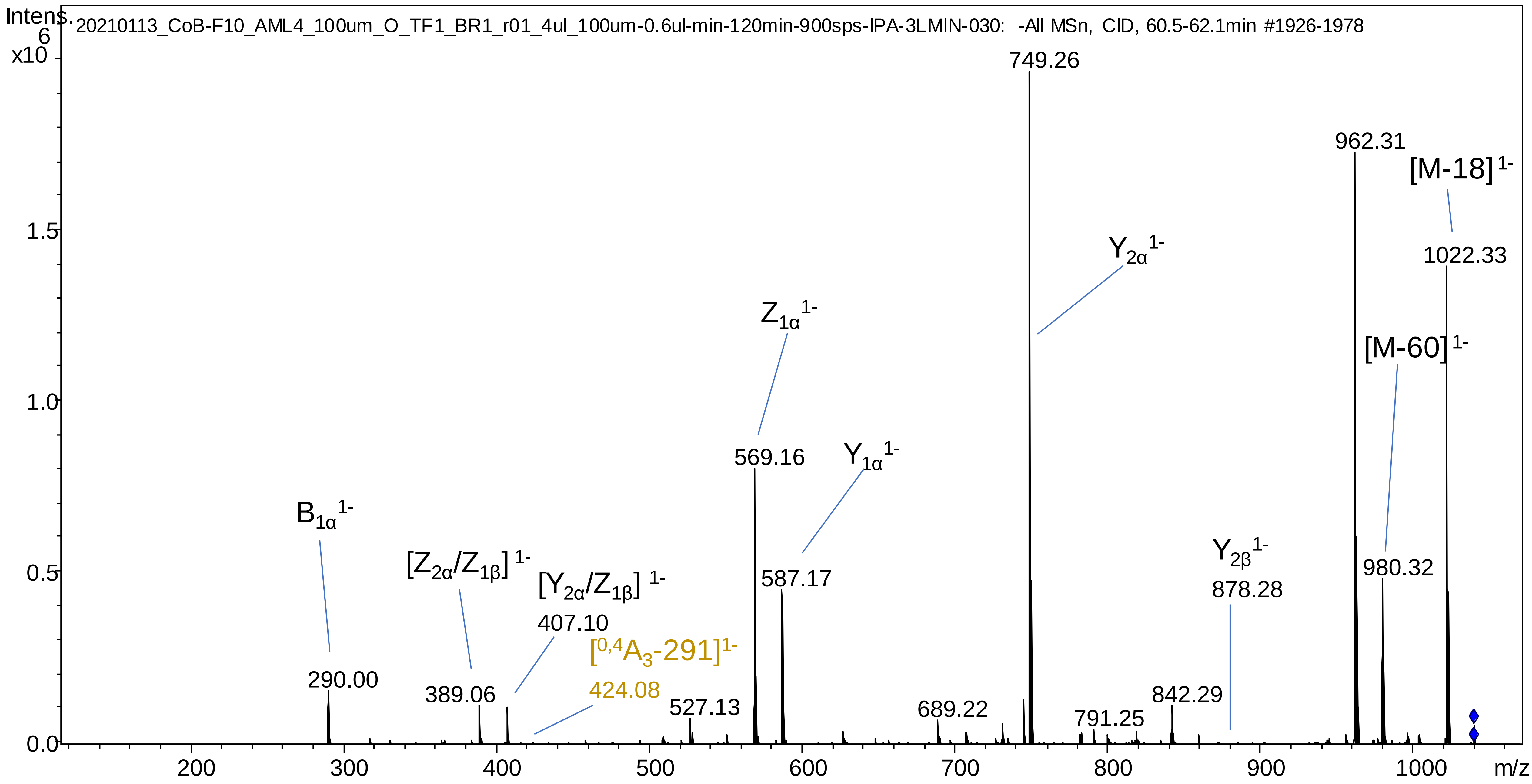

Glycan 10b

H2N2S1

Depicted MS^2 was obtained from analysis of cell line: HL-60

**Monoisotopic mass:**	**1041.40 Da**
**Charge observed:**	**1-**
**Theoretical ion:**	***m/z* 1040.39**
**Observed ion:**	***m/z* 1040.33**
**Mass deviation:**	***m/z* 0.06**
**Retention time:**	**61.3 min**

Note: α-2,3 linkage of sialic acid confirmed by neuraminidase S and A treatment

UniCarb-DB: #2643

Elution order of isomers in line with Jin, Chunsheng, et al. *Mol. & Cell. Proteomics* 16.5 (2017): 743-758.

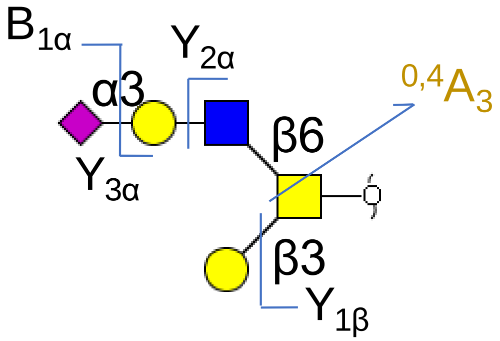

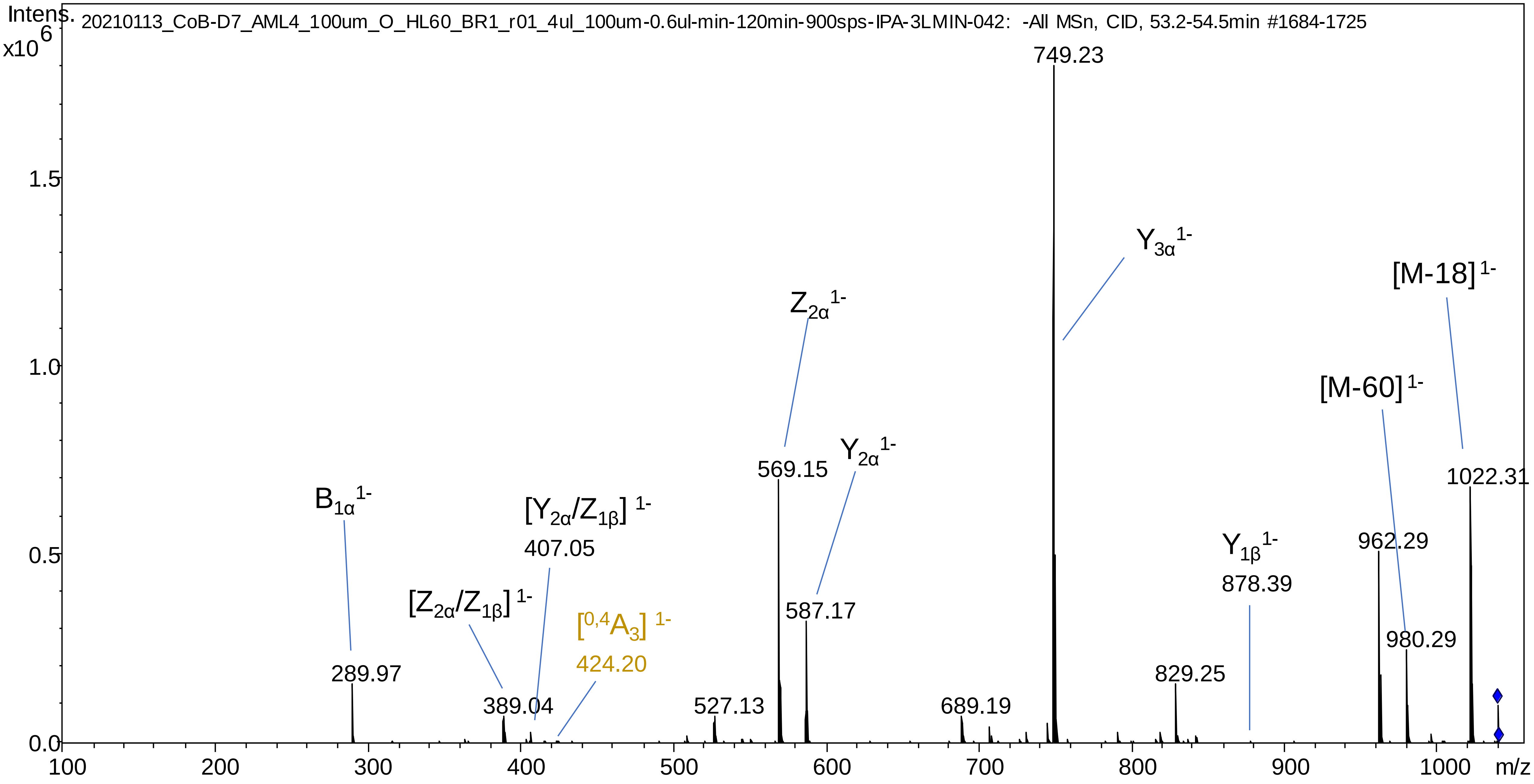

Glycan 11

H3N3

Depicted MS^2 was obtained from analysis of cell line: MV4-11

**Monoisotopic mass:**	**1115.43 Da**
**Charge observed:**	**1-**
**Theoretical ion:**	***m/z* 1114.42**
**Observed ion:**	***m/z* 1114.38**
**Mass deviation:**	***m/z* 0.04**
**Retention time:**	**65.8 min**

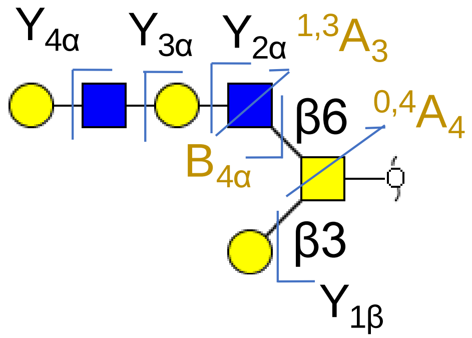

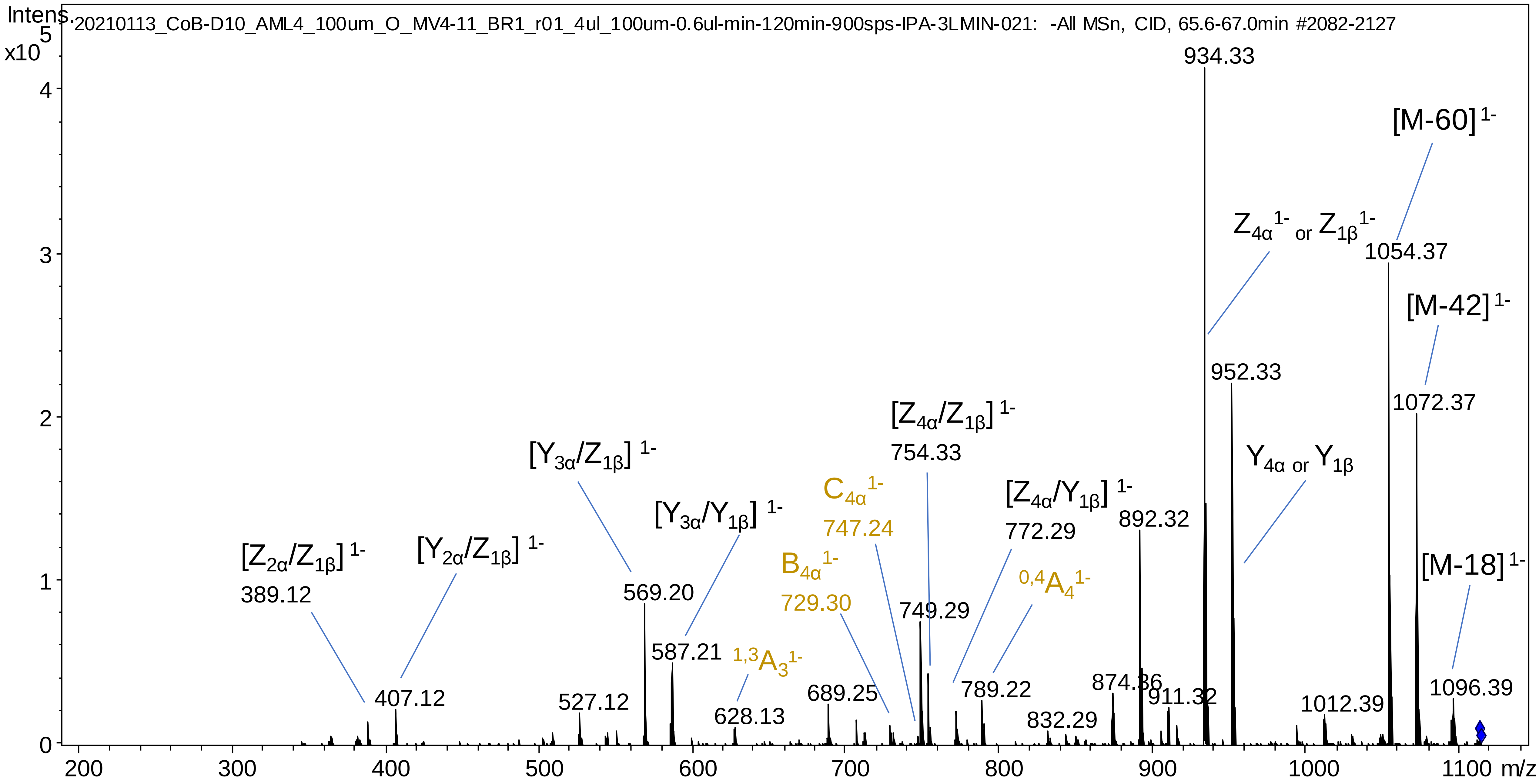

Glycan 12a

H2N2F1S1

Depicted MS^2 was obtained from analysis of cell line: HL-60

Monoisotopic mass: 1187.45 Da
Charge observed: 1-
Theoretical ion: *m/z* 1186.44
Observed ion: *m/z* 1186.37
Mass deviation: *m/z* 0.07
Retention time: 47.7 min
Note: α-2,3 linkage of sialic acids confirmed by neuraminidase S and A treatment; *m/z* 364 fragment indicates Lewis x

UniCarb-DB: #493

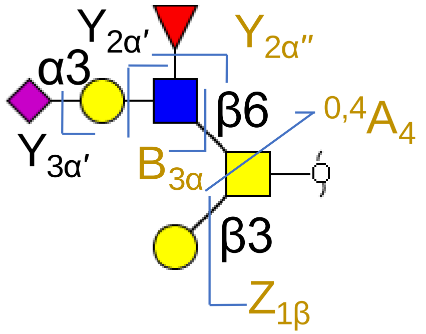

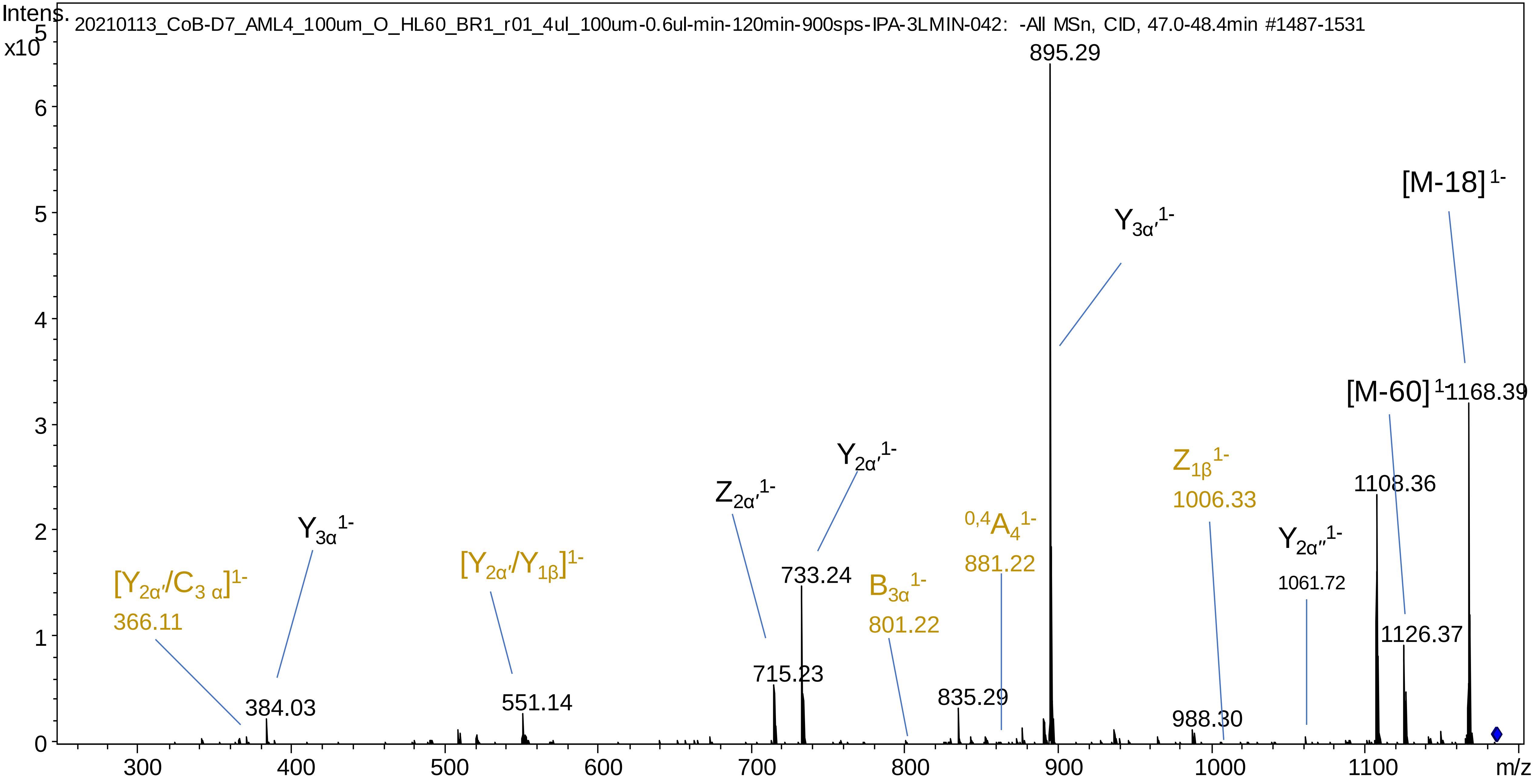

Glycan 12b

H2N2F1S1

Depicted MS² was obtained from analysis of cell line: TF-1

**Monoisotopic mass:**	**1187.45 Da**	**UniCarb-DB: #165**
**Charge observed:**	**1-**	
**Theoretical ion:**	***m/z* 1186.44**	
**Observed ion:**	***m/z* 1186.40**	
**Mass deviation:**	***m/z* 0.04**	
**Retention time:**	**62.5 min**	

Note: α-2,3 linkage of sialic acids confirmed by neuraminidase S and A treatment; *m/z* 325 and *m/z* 409 fragment indicate a H-epitope

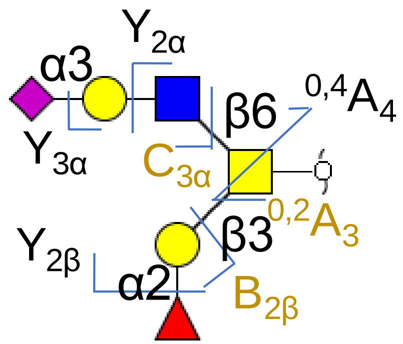

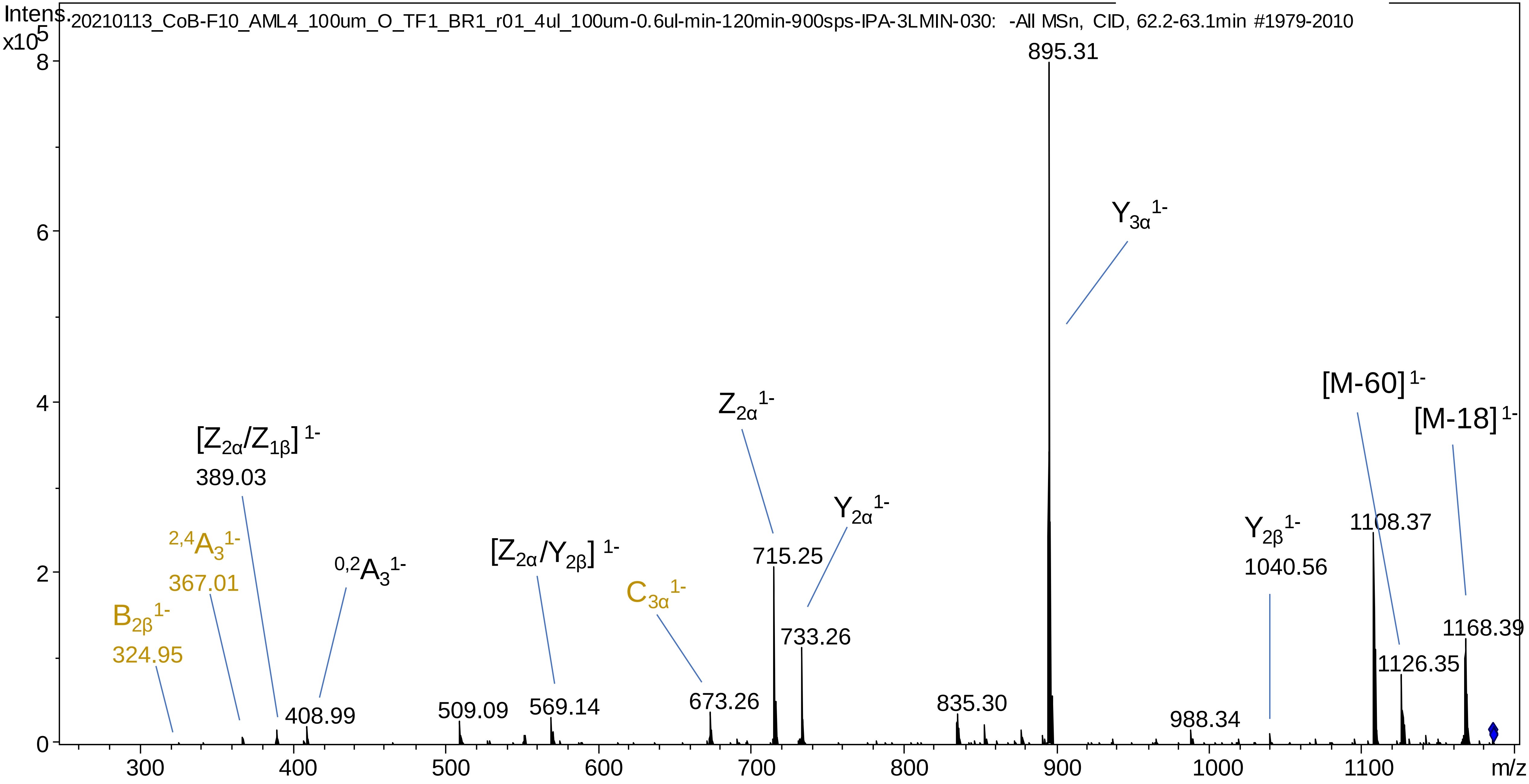

Glycan 13

H1N1S3

Depicted MS² was obtained from analysis of cell line: TF-1

**Monoisotopic mass:**	**1258.45 Da**
**Charge observed:**	**1-**
**Theoretical ion:**	***m/z* 1257.44**
**Observed ion:**	***m/z* 1257.40**
**Mass deviation:**	***m/z* 0.04**
**Retention time:**	**39.9 min**

Note: *m/z* 581 indicates two sialic acids linked to each other

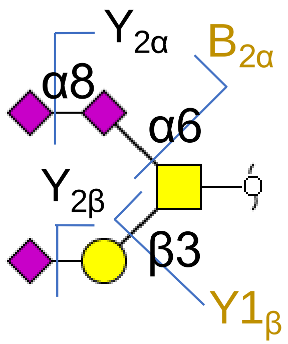

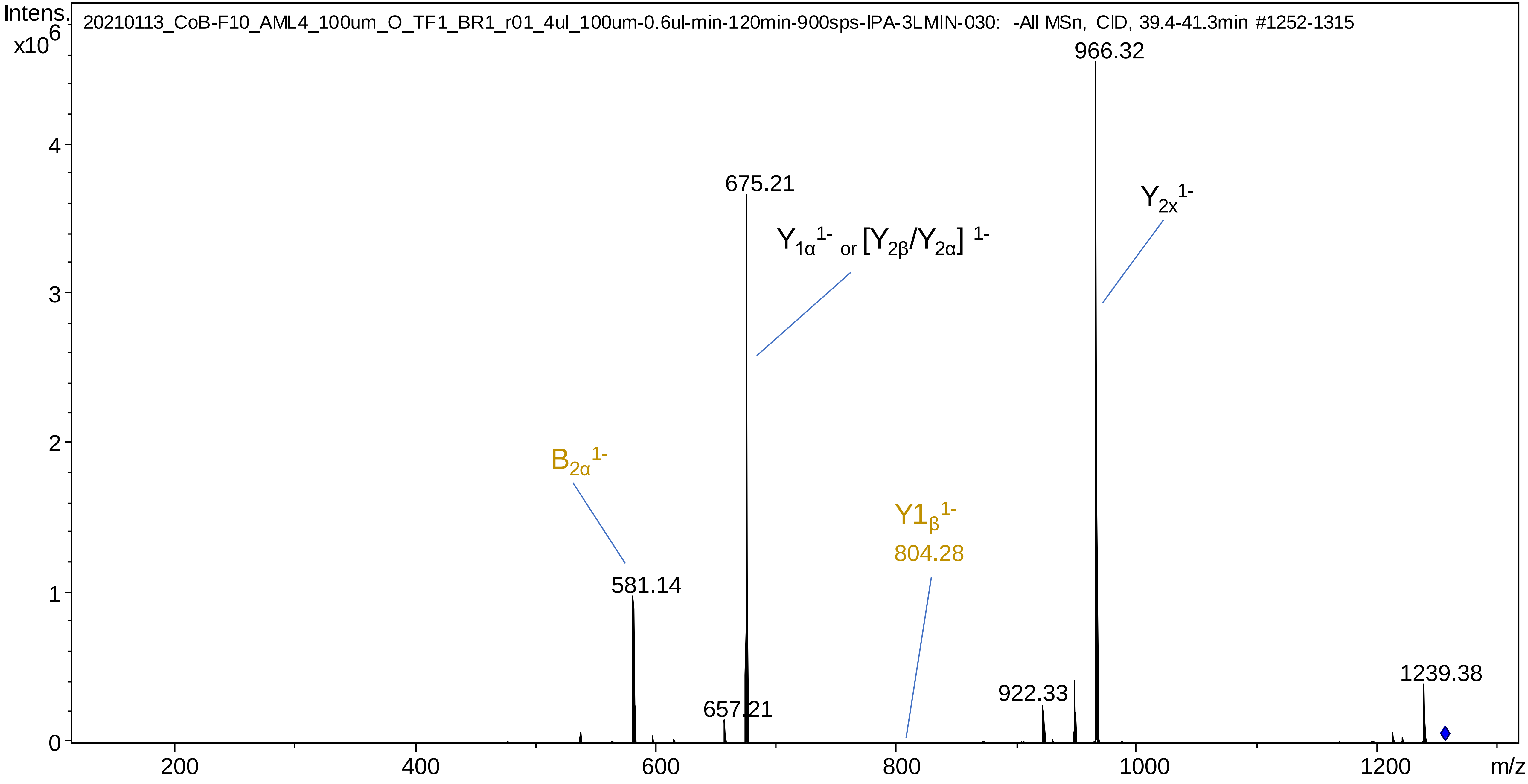

Glycan 13

H1N1S3

Depicted MS^2 was obtained from analysis of cell line: TF-1

**Monoisotopic mass:**	**1258.45 Da**
**Charge observed:**	**2-**
**Theoretical ion:**	***m/z* 628.22**
**Observed ion:**	***m/z* 628.15**
**Mass deviation:**	***m/z* 0.05**
**Retention time:**	**39.9 min**

Note: *m/z* 581 indicates two sialic acids linked to each other

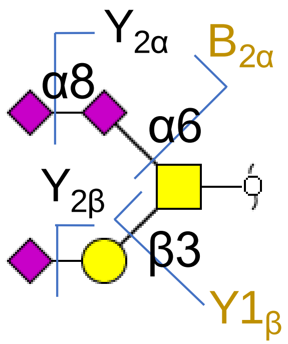

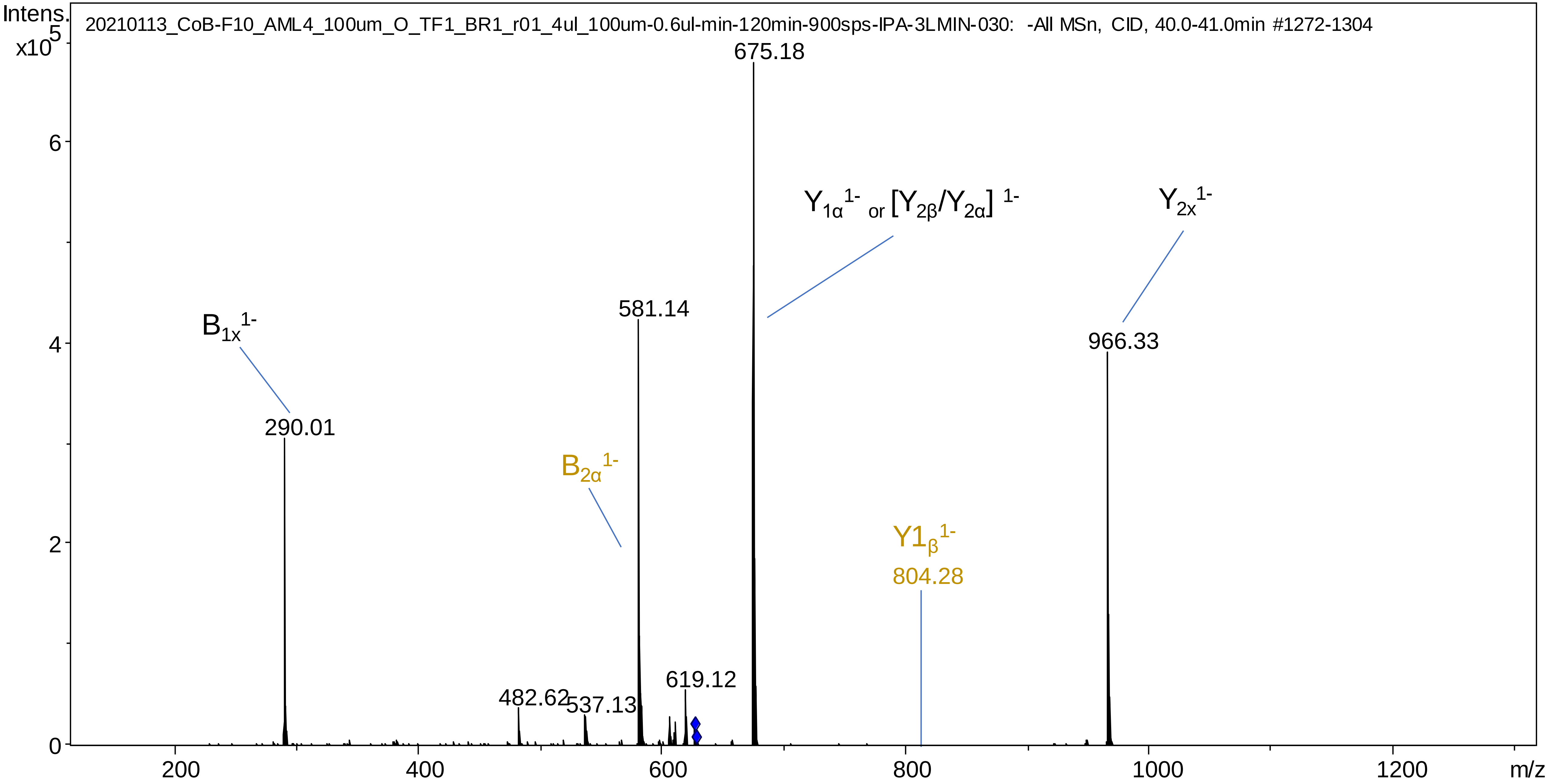

Glycan 14

H2N2S2

Depicted MS^2 was obtained from analysis of cell line: HL-60

**Monoisotopic mass:**	**1332.48 Da**
**Charge observed:**	**1-**
**Theoretical ion:**	***m/z* 1331.47**
**Observed ion:**	***m/z* 1331.44**
**Mass deviation:**	***m/z* 0.03**
**Retention time:**	**65.4 min**

UniCarb-DB: #502

Note:: α-2,3 linkage of both sialic acids confirmed by neuraminidase S and A treatment as no species were detected with α-2,6 as a digested H2N2S1

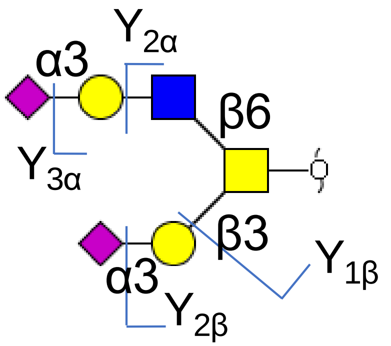

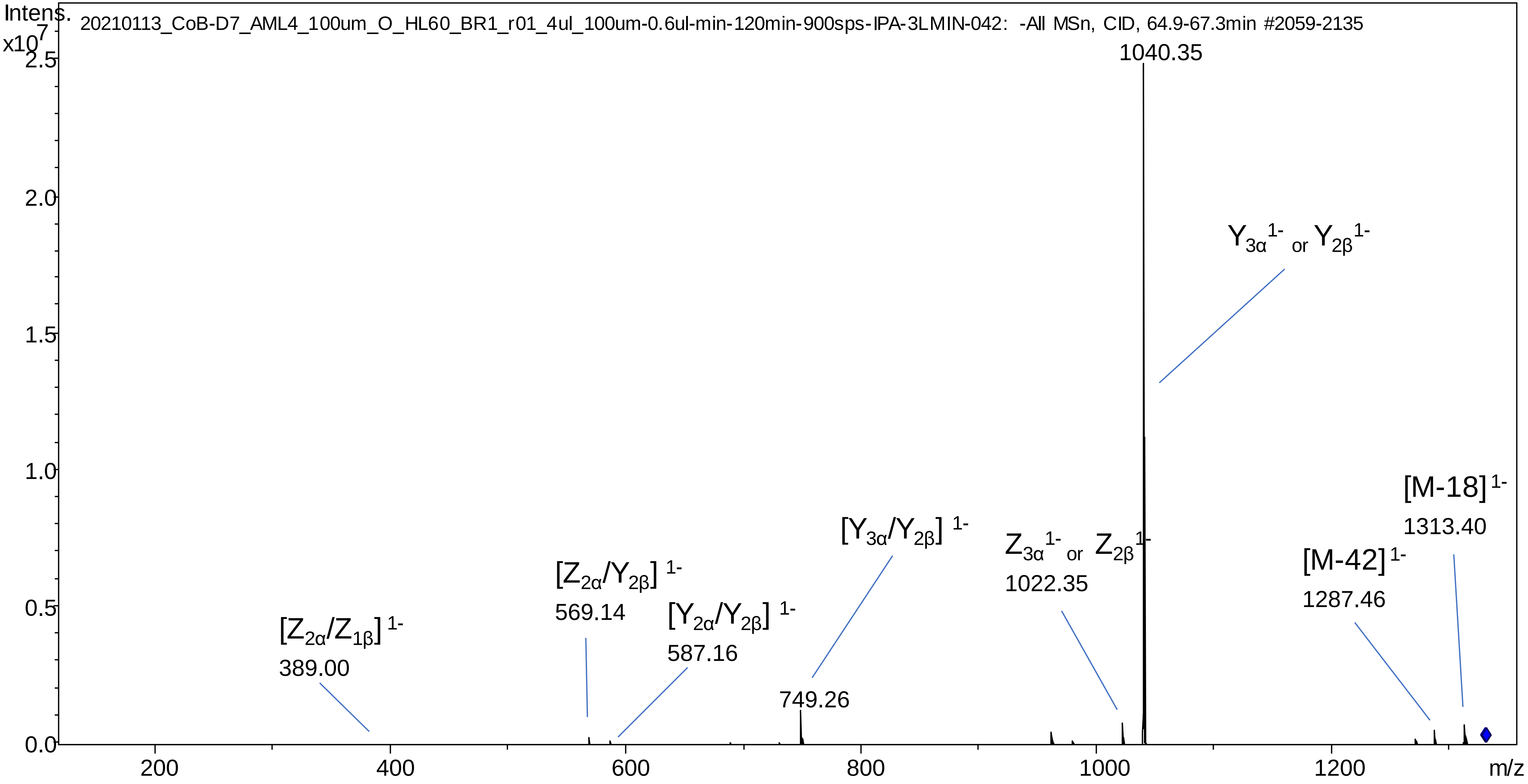

Glycan 14

H2N2S2

Depicted MS^2 was obtained from analysis of cell line: HL-60

Monoisotopic mass: 1332.48 Da
Charge observed: 2-
Theoretical ion: *m/z* 665.23
Observed ion: *m/z* 665.19
Mass deviation: *m/z* 0.04
Retention time: 65.4 min
Note:: α-2,3 linkage of both sialic acids confirmed by neuraminidase S and treatment as no species were detected with α-2,6 as a digested H2N2S1

UniCarb-DB: #169

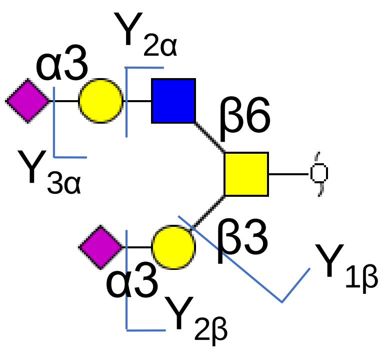

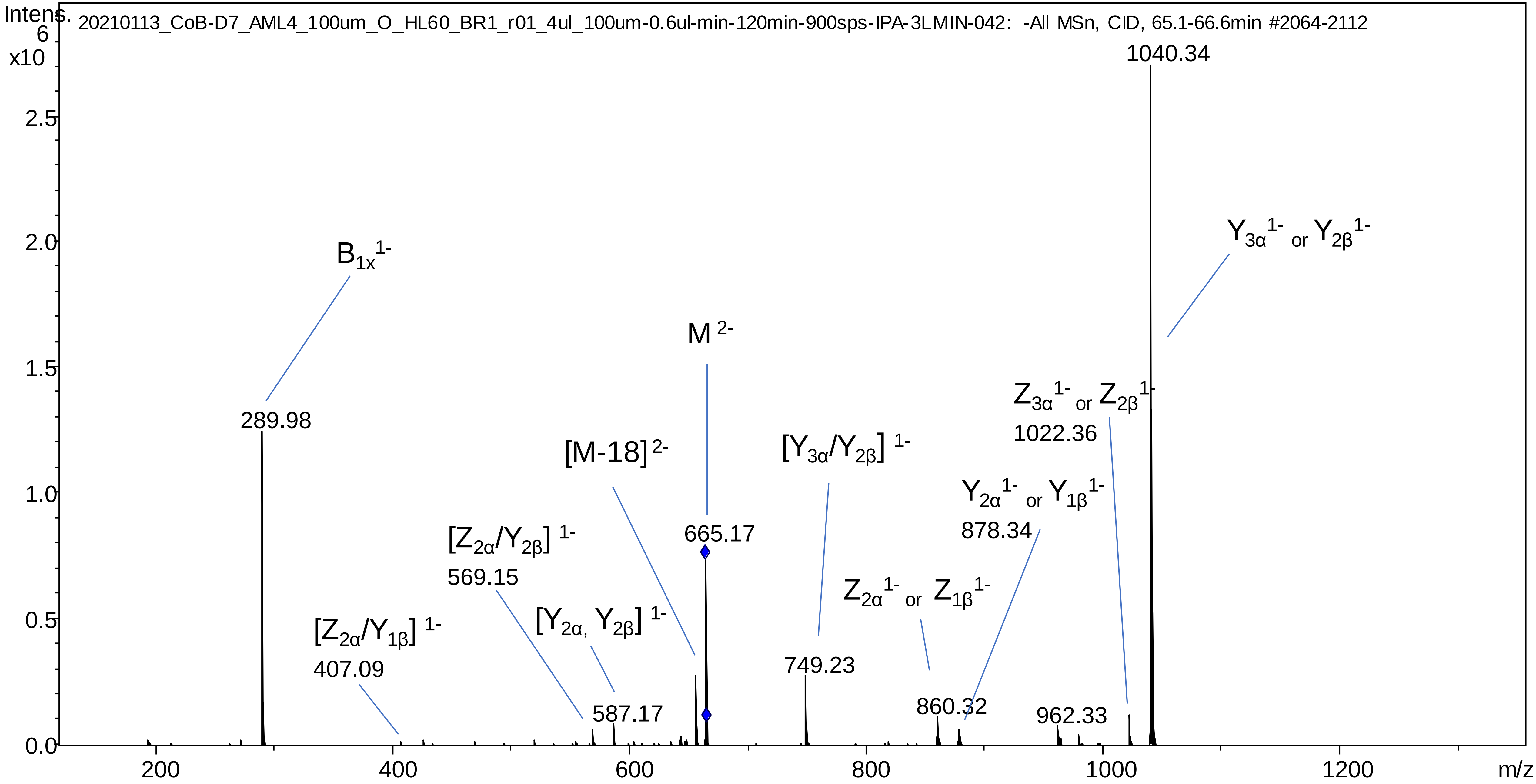

Glycan 15

H2N2S2Su1

Depicted MS^2 was obtained from analysis of cell line: TF-1

Monoisotopic mass: 1412.44 Da
Charge observed: 2-
Theoretical ion: *m/z* 705.21
Observed ion: *m/z* 705.14
Mass deviation: *m/z* 0.07
Retention time: 58.3 min

UniCarb-DB: #2650

Note: α-2,3 linkage of both sialic acids confirmed by neuraminidase S and A treatment as no species were detected with α-2,6 as a digested H2N2S1Su1

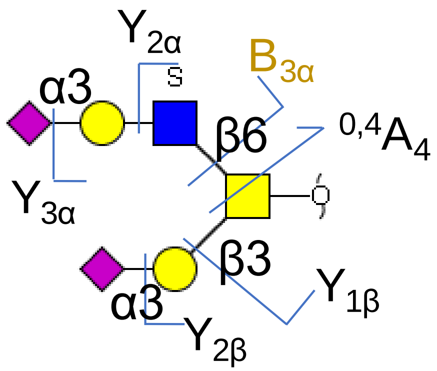

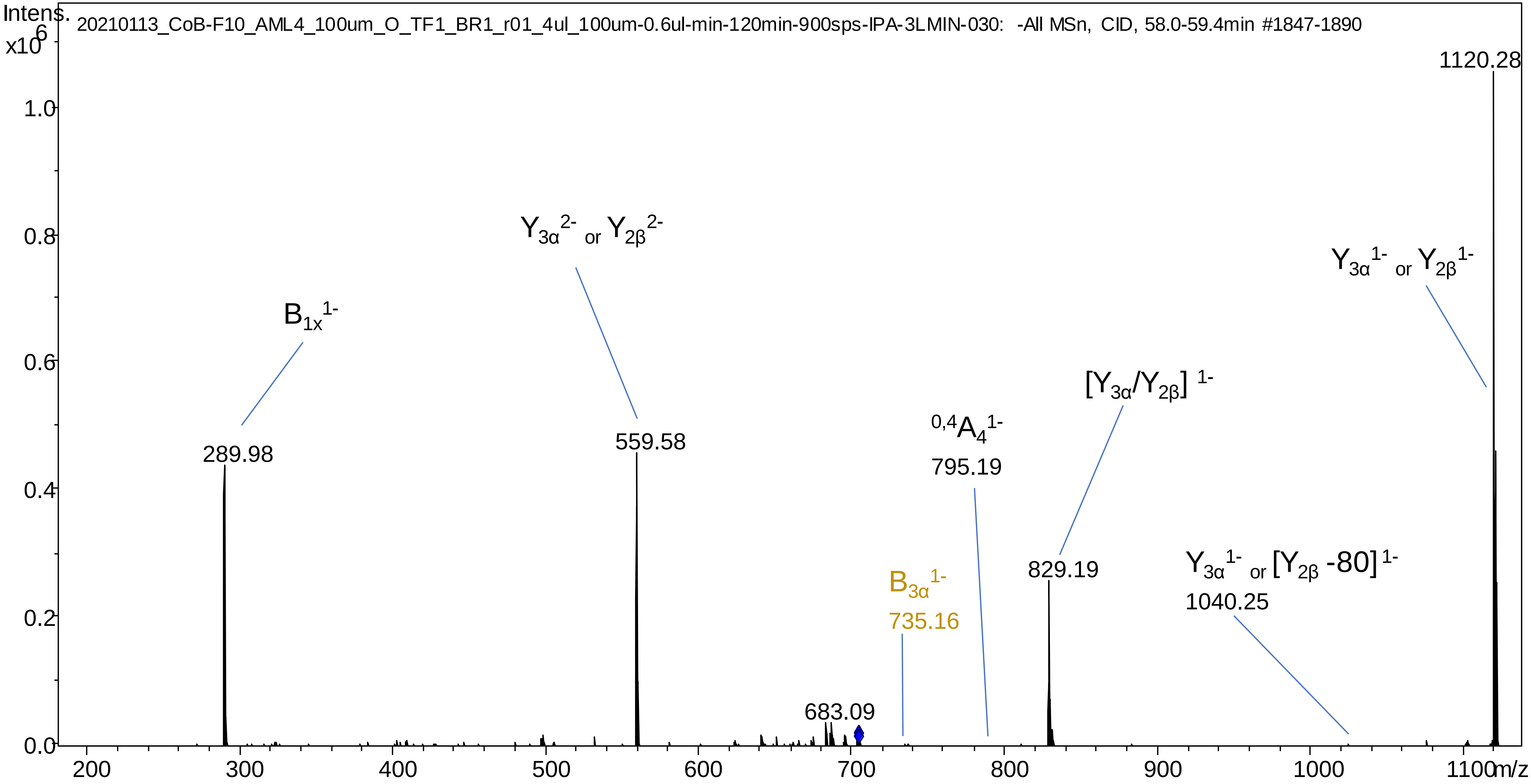

Glycan 16

H2N2F1S2

Depicted MS[2] was obtained from analysis of cell line: HL-60

**Monoisotopic mass:**	**1478.54 Da**	**UniCarb-DB: #61**
**Charge observed:**	**2-**	
**Theoretical ion:**	***m/z* 738.26**	
**Observed ion:**	***m/z* 738.21**	
**Mass deviation:**	***m/z* 0.05**	
**Retention time:**	**54.6 min**	

Note: α-2,3 linkage of both sialic acids confirmed by neuraminidase S and A treatment as no species were detected with α-2,6 as a digested H2N2F1S1; *m/z* 364 fragment indicates Lewis x

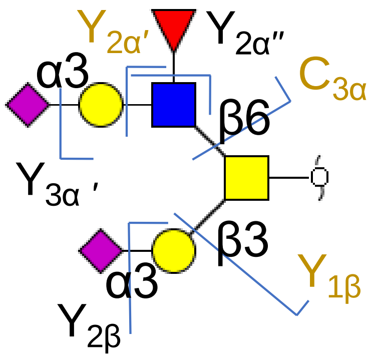

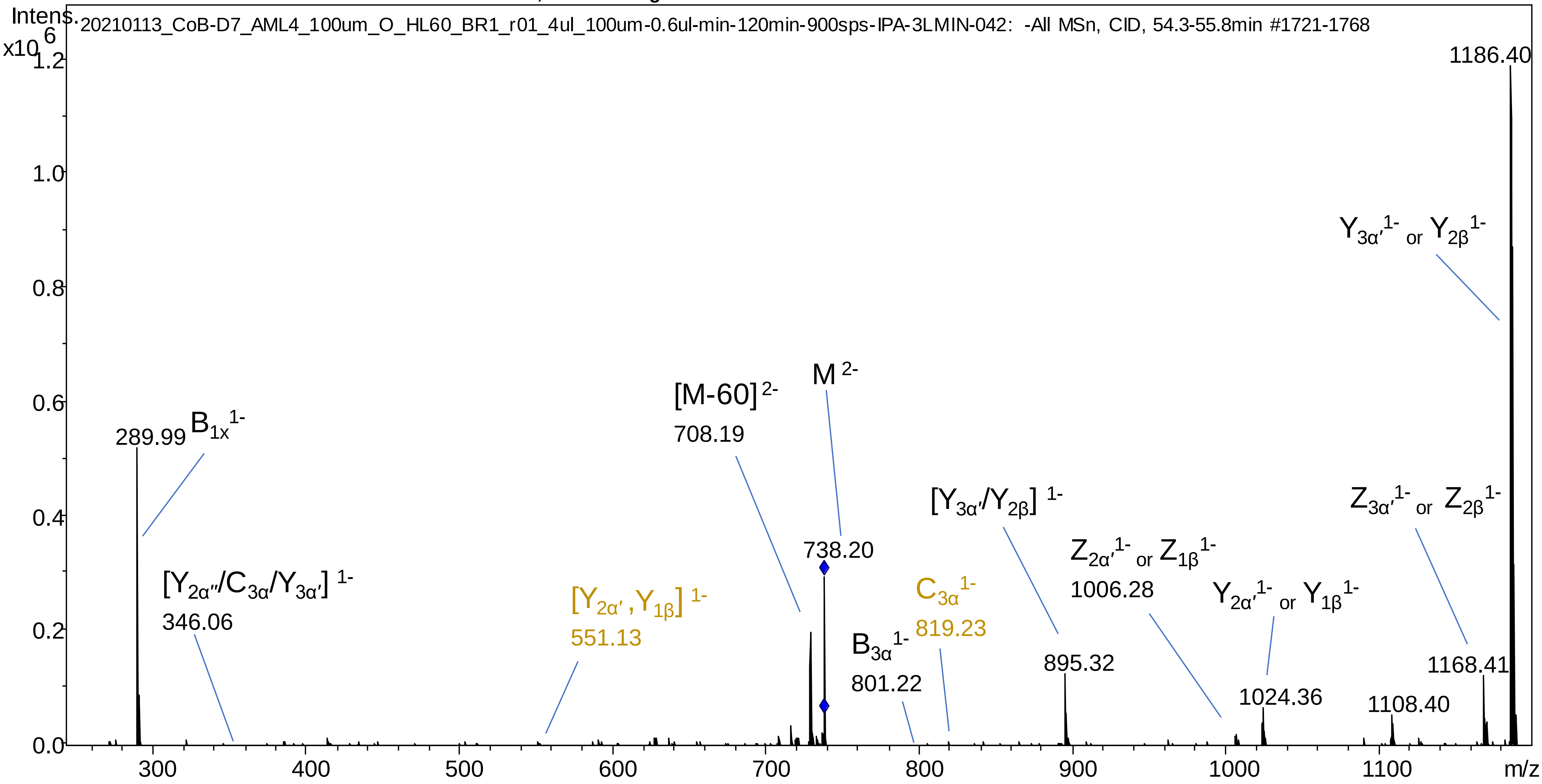

Glycan 17

H2N2F1S2Su1

Depicted MS[2] was obtained from analysis of cell line: M-07e

**Monoisotopic mass:**	**1558.50 Da**
**Charge observed:**	**2-**
**Theoretical ion:**	***m/z* 778.25**
**Observed ion:**	***m/z* 778.20**
**Mass deviation:**	***m/z* 0.05**
**Retention time:**	**51.6 min**

UniCarb-DB: #2947

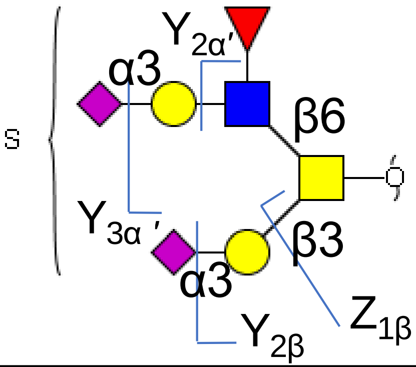

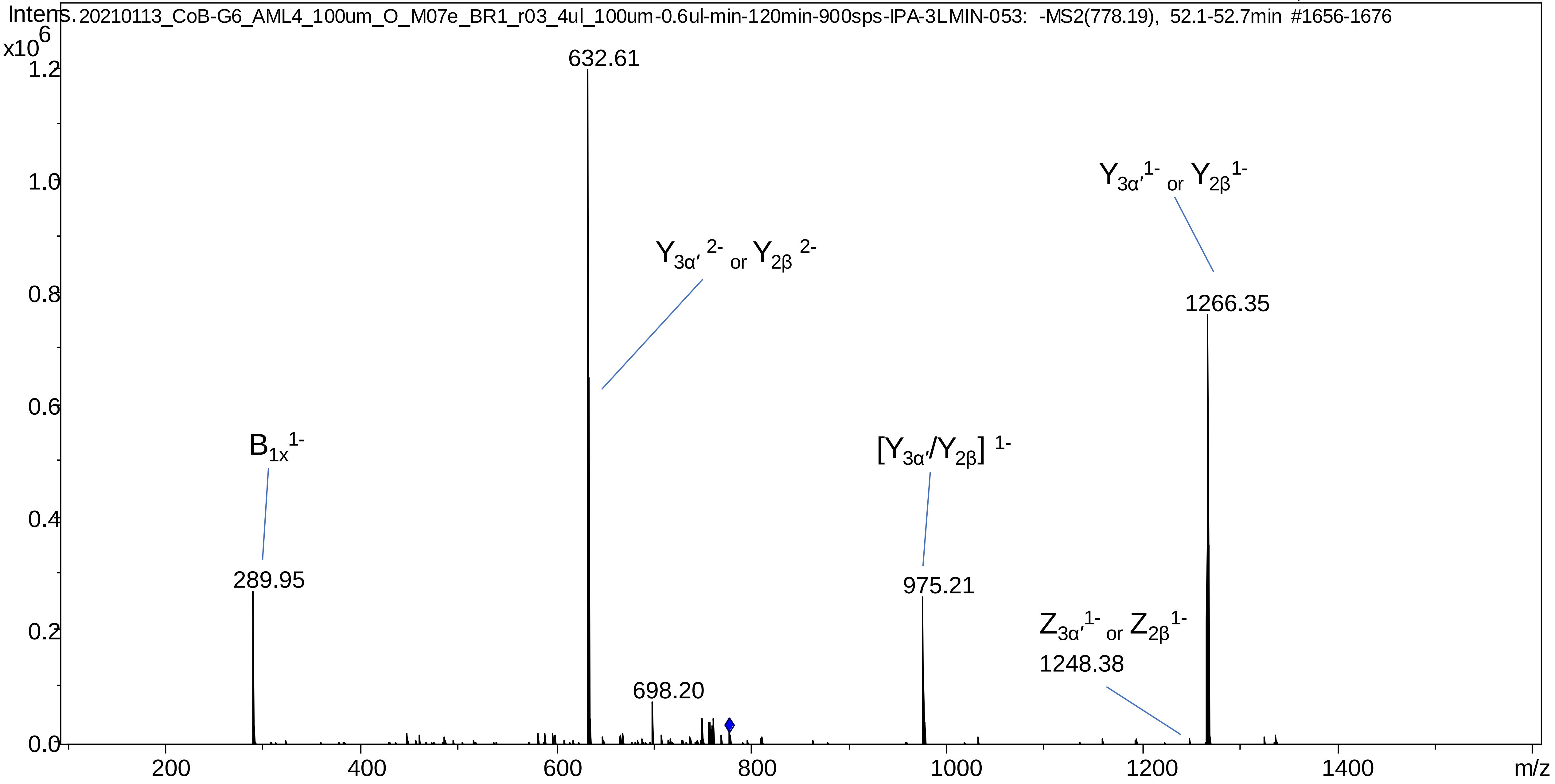

Glycan 18a

H3N3S2

Depicted MS[2] was obtained from analysis of cell line: TF-1

**Monoisotopic mass:**	**1697.61 Da**
**Charge observed:**	**2-**
**Theoretical ion:**	***m/z* 847.80**
**Observed ion:**	***m/z* 847.76**
**Mass deviation:**	***m/z* 0.04**
**Retention time:**	**77.0 min**

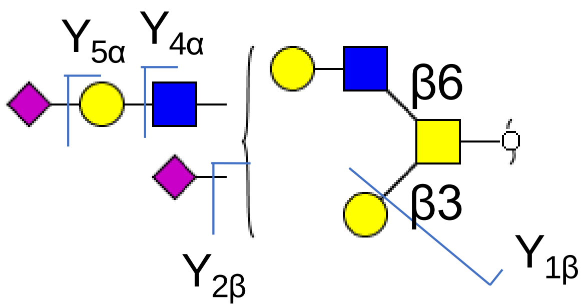

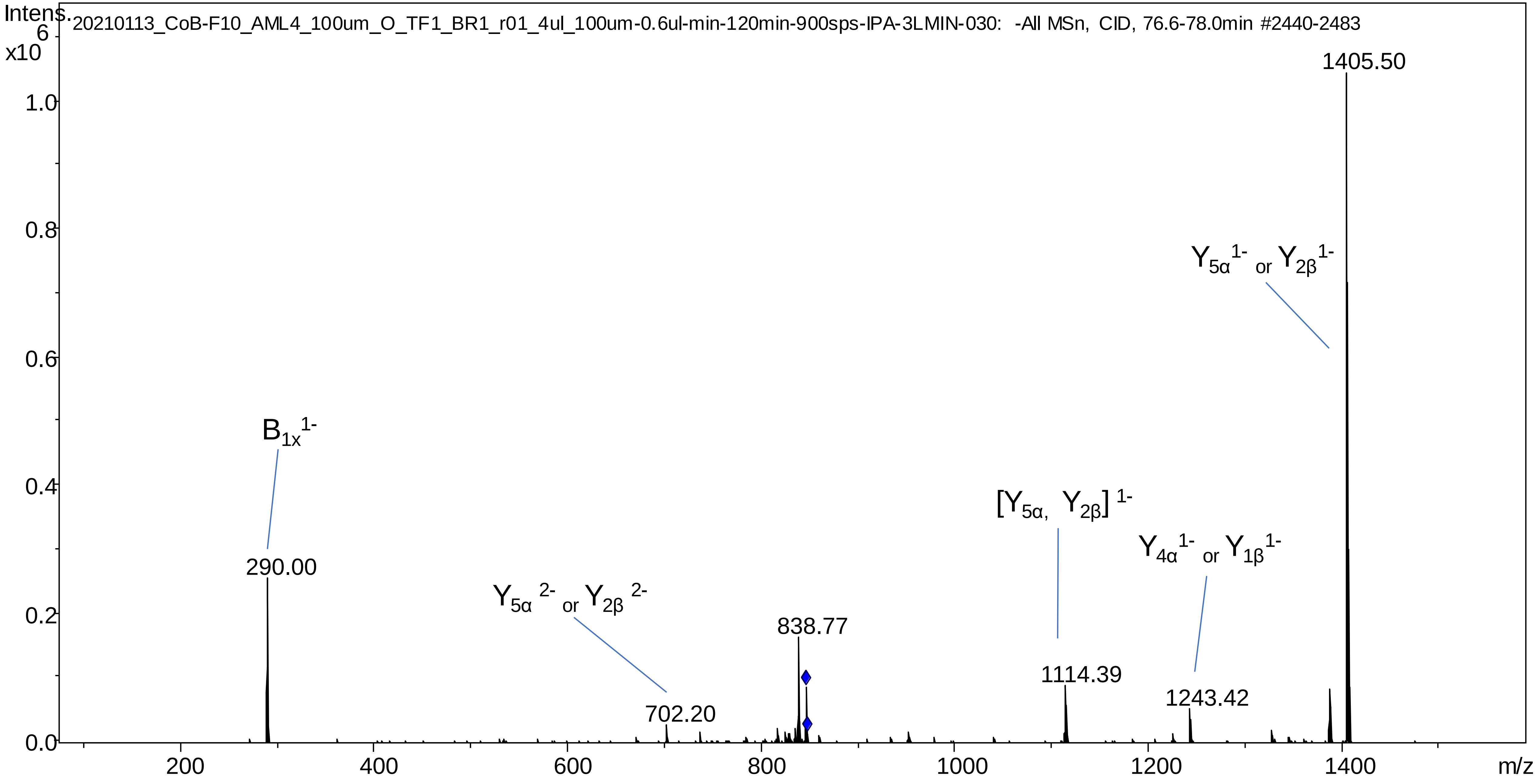

Glycan 18b

H3N3S2

Depicted MS² was obtained from analysis of cell line: HL-60

**Monoisotopic mass:**	**1697.61 Da**
**Charge observed:**	**2-**
**Theoretical ion:**	***m/z* 847.80**
**Observed ion:**	***m/z* 847.75**
**Mass deviation:**	***m/z* 0.05**
**Retention time:**	**82.1 min**

Note: α-2,3 linkage of both sialic acids confirmed by neuraminidase S and A treatment as no species were detected with α-2,6 as H3N3S1

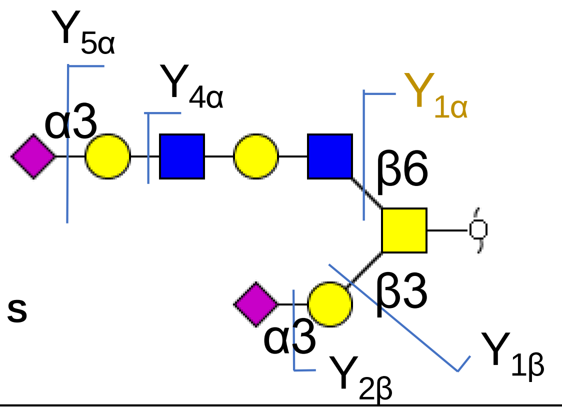

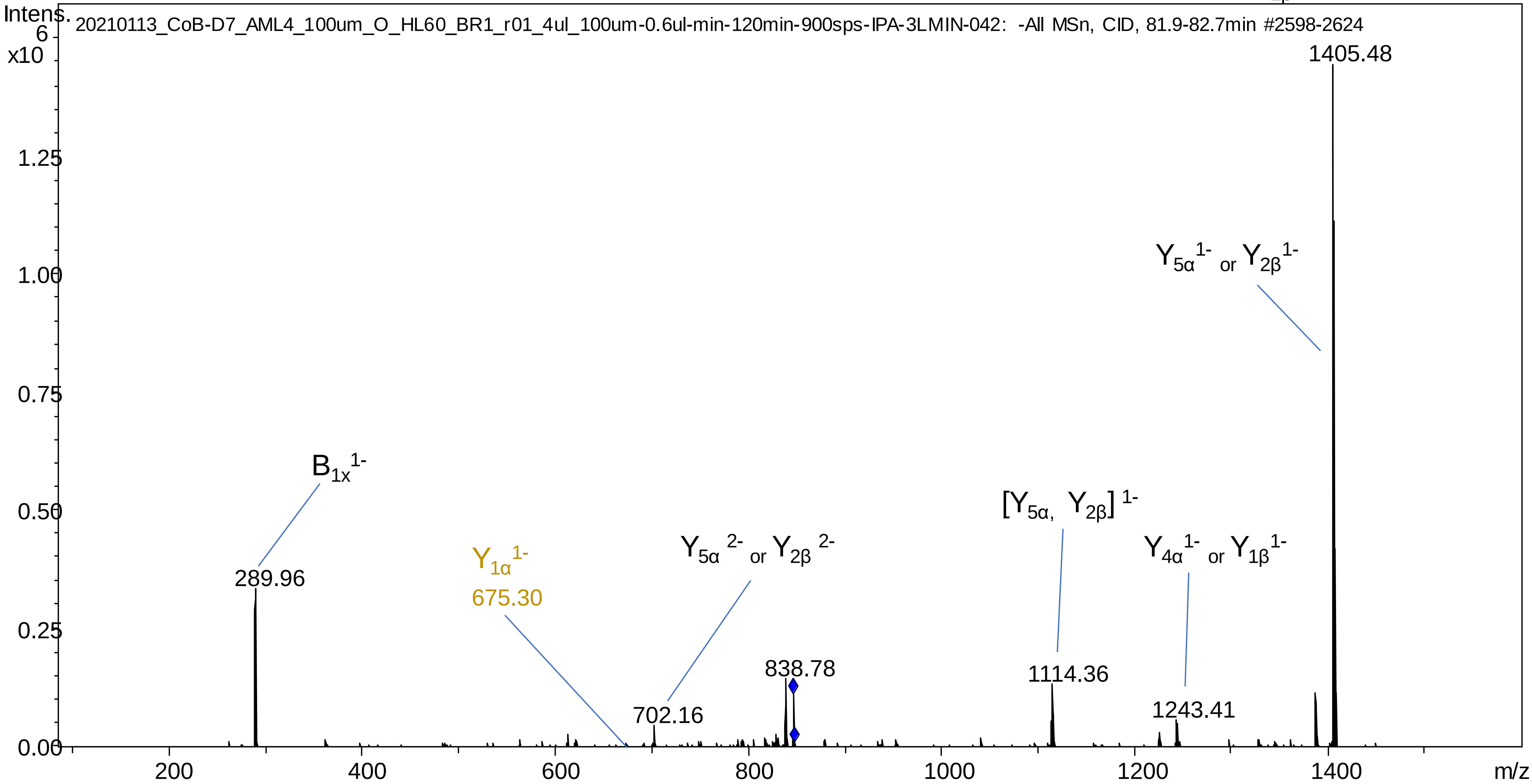

Glycan 19

H3N3F1S2

Depicted MS² was obtained from analysis of cell line: HL-60

**Monoisotopic mass:**	**1843.67 Da**
**Charge observed:**	**2-**
**Theoretical ion:**	***m/z* 920.79**
**Observed ion:**	***m/z* 920.77**
**Mass deviation:**	***m/z* 0.02**
**Retention time:**	**69.2 min**

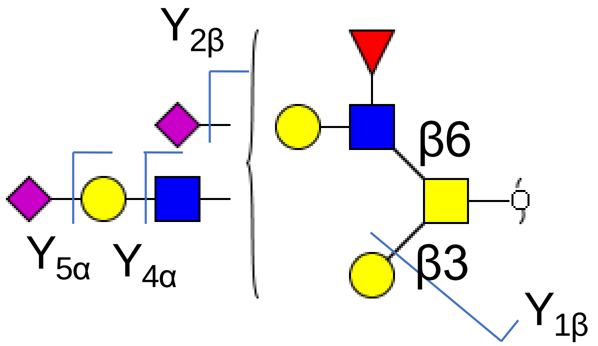

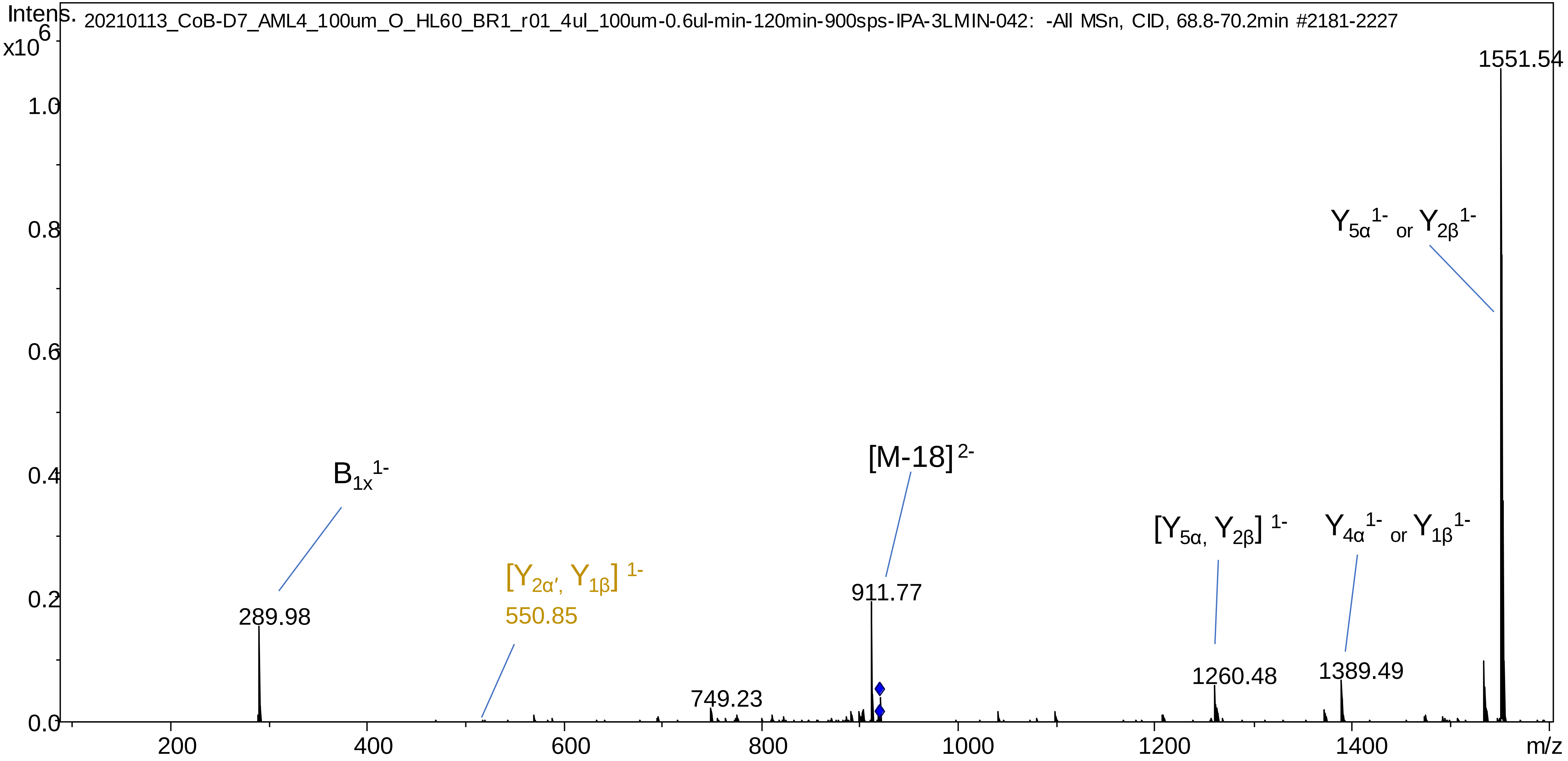

Supplement: Supplementary file 1 [file cells-10-03058-s001.zip › Supplementary_Information_2.pdf]
